# Spectroelectrochemical study of carbon structural and functionality characteristics on vanadium redox reactions for flow batteries†

**DOI:** 10.1039/d4ma00675e

**Published:** 2024-08-15

**Authors:** Ha H. Phan, Jon G. Bell, Greg A. Mutch, Alan J. McCue, Anh N. Phan, K. Mark Thomas

**Affiliations:** a Wolfson Northern Carbon Reduction Laboratories, School of Engineering, Newcastle University Newcastle upon Tyne NE1 7RU UK anh.phan@ncl.ac.uk mark.thomas@ncl.ac.uk; b School of Engineering, Newcastle University Newcastle upon Tyne NE1 7RU UK; c School of Chemistry, University of Aberdeen Aberdeen AB24 3UE UK

## Abstract

Vanadium redox flow batteries have applications for large-scale electricity storage. This paper reports the influence of carbon structural characteristics of sustainable walnut shell-derived carbons in carbon/polyvinylidene fluoride composite electrodes on vanadium redox reactions. Pyrolysis, gasification, and chemical treatment procedures were used to modify the structural characteristics of carbons. Carbon functional groups were modified by chemical treatment with HNO_3_, heat treatment with K_2_CO_3_, and high-temperature NH_3_ treatment. Carbon porous structures were characterized using gas adsorption studies. Raman spectroscopy and X-ray diffraction were used to characterize the carbon molecular structure. Functional groups were characterized using X-ray photoelectron spectroscopy, acid/base titrations, temperature-programmed desorption, and Fourier transform infrared spectroscopy. The influence of carbon structure, porosity, and surface functional groups on the redox reactions of vanadium was investigated using cyclic voltammetry and electrical impedance spectroscopy. The VO^2+^/VO_2_^+^ and V^2+^/V^3+^ couples had well-defined peaks in cyclic voltammetry, with the former being the most intense, but the V^3+^/VO^2+^ couple was not observed for samples carbonized under nitrogen. The results show that V^2+^/V^3+^ and VO^2+^/VO_2_^+^ couples observed in cyclic voltammograms were enhanced for carbonization temperatures up to 800 °C. Electrical impedance spectroscopy also showed impedance trends. The electrochemistry results are primarily related to changes in carbon structure and the catalysis of V^3+^ oxidation by surface functional groups in the carbon structure. The V^3+^/VO^2+^ couple was limited by slow kinetics, but it occurs on specific oxygen and nitrogen sites in the carbon structure. The oxidation of V(iii) to V(iv) only occurs on a limited number of surface sites, and the outer-sphere electron transfer to oxidize V(iii) takes place at much more positive potentials. The coulombic, voltage, and energy efficiency of the carbon electrodes were suitable for batteries.

## Abstract 2

The oxidation of V(iii) to V(iv) only occurs on a limited number of specific functional group sites and the outer-sphere electron transfer to oxidize V(iii) only takes place at much more positive potentials.

## Graphical abstract

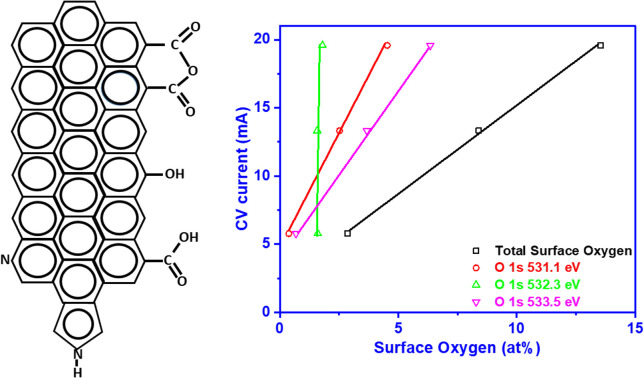

## 1. Introduction

Vanadium redox flow batteries (VRFBs) are potential commercial large-scale energy storage systems with plants constructed in recent years in several countries to store energy produced from renewable energy plants.^1–3^ The main advantages of VRFBs include high safety (nonflammable, no risk of thermal runaway), good scalability, and low contamination between cathode and anode due to only one metal being used in electrolyte solutions. Both electrodes are made of carbon materials with a polymer binder, and long life cycles (up to 20 000 cycles) are achieved compared to lithium-ion batteries (5000–15 000 cycles) and sodium-ion batteries (3000–7000 cycles).^4–6^ Graphite or graphite composite materials employed as bipolar plates/electrodes are one of the main components in VRFBs due to their low cost, high electric conductivity, wide potential window, and high chemical resistance to both acidic and oxidizing environments.^7,8^ However, graphite lacks significant heteroatom functional groups and a porous structure, which have been shown to enhance the kinetics of vanadium redox reactions and the energy efficiency of VRFBs.^9–11^ Biomass-derived activated carbons^12^ have received attention as a potential renewable carbon source for VRFBs since biomass-derived carbon is more cost-effective than its counterparts and provides high porosity and oxygen functional group contents.^13,14^ Therefore, using biomass-derived carbon can help reduce the cost of VRFBs and potentially enhance performance.

Functional groups can act as adsorption sites and modify the dispersion of surface catalytic particles.^15,16^ The impact of functional groups, porous structure, and carbon structure on the kinetics of vanadium redox reactions have been studied on many forms of carbon (carbon nanotubes, carbon blacks, graphite flakes, graphite felt, *etc.*). Nitrogen and phosphorus functional groups in pristine polyaniline graphite felt (a non-porous structure with very low surface area (<1 m^2^ g^−1^) demonstrated catalytic effects for both VO^2+^/VO_2_^+^, V^3+^/VO^2+^, and V^2+^/V^3+^ increasing the reversibility of vanadium reactions and reducing interface transfer resistance compared to the original electrodes.^9^ An increase in the surface area of graphite felt (18 m^2^ g^−1^) compared to untreated graphite felt (3 m^2^ g^−1^) resulted in a reduction in overpotential, higher energy efficiency, and electrolyte utilization in VRFBs.^17^ Density functional theory calculations on graphite electrodes also showed that nitrogen, phosphorus, and boron-doped graphite increased the wettability and electronic conductivity towards vanadium redox reactions.^10^ Multiwalled carbon nanotubes were used to ascertain the impact of phosphorus content on the electrocatalytic activity of the VO^2+^/VO_2_^+^ couple with phosphorus enhancing the electrocatalytic properties of the activity of electrodes, which led to higher energy efficiency than non-doped electrodes.^18^

Biomass-derived carbons are complex with a wide range of pore size distributions, surface areas, and functional groups, which may vary widely depending on the biomass precursor and carbonization conditions. Carbons derived from orange peel,^13^ coconut shell,^14^ and sal wood sawdust^19^ had high mesopore and micropore contents and BET surface areas up to ∼1900 m^2^ g^−1^. The high mesopore contents in orange peel-derived carbon^13^ and coconut shell-derived carbon^14^ significantly enhanced the kinetics of vanadium redox reactions and increased the energy efficiency of VRFBs compared to pristine graphite plates. However, it was shown that coffee bean-derived carbon had the highest micropore and mesopore, but the biochar did not have improved electron transfer kinetics or battery efficiency for vanadium redox flow batteries.^20^ Maharjan *et al.* showed that sal wood-derived carbon with a micropore volume of ∼0.42 cm^3^ g^−1^ and a mesopore volume of ∼0.93 cm^3^ g^−1^ improved the kinetics of vanadium redox reactions, and the energy efficiency of this activated carbon was the same as pristine graphite plate.^19^ Most studies in biomass-derived carbons^13,14,19^ attributed oxygen functional groups as catalytic sites for vanadium redox reactions, but these ACs underwent high temperature treatment (800 °C), which was sufficient to eliminate most of the oxygen functional groups out of ACs, and this can be ascertained through temperature-programmed desorption analysis.^21–23^ The surface oxygen functional groups in these biomass-derived carbons were identified using X-ray photoelectron spectroscopy (XPS) analysis,^13,14,19^ which provides the surface composition (up to ∼5 nm) rather than bulk oxygen content for organic materials.^24^ Furthermore, the oxygen functional group distribution in carbon micropores and mesopores may have different influences on the kinetics of vanadium redox reactions due to the accessibility of vanadium ions. In addition, the role of carbon structure π–π* in electron transfer is also a critical factor influencing electrical conductivity in carbons.^25^

In VRFBs energy is stored as redox pairs in the electrolyte solutions. The carbon electrodes function as catalysts but do not undergo redox reactions during discharge/charge operations.^26^ The complexity of vanadium flow battery reactions is well established, especially at highly acid conditions, and there are usually mixtures of surface functional groups present on heterogeneous carbon surfaces. Most studies of the electrochemistry of vanadium reactions and surface chemistry are for dilute electrolyte solutions, and more work is required on concentrate solutions where hydrolytic, polymerization, and ion association processes may occur.^27^ The aim of the research was to increase insight into the impact of carbonaceous structure and surface functional groups in sustainable biomass-derived carbons on the catalysis of vanadium redox reactions for concentrated electrolyte solutions. Currently, VFRBs are constrained by the electrochemical activity of carbon electrodes and a limited understanding of surface redox reactions. The kinetics of vanadium species are often slow on carbon surfaces, and hence there is interest in functionalization of carbon surfaces to improve operation. It is not clear if electrode oxidation treatment improves the performance of both electrodes to the same extent.^28^ In this paper, the carbon structure and surface functional group characteristics of biomass carbons derived from walnut shells were varied systematically using heat treatment temperature (HTT), hold time at maximum heat treatment temperature, and carbonization atmosphere (N_2_ and CO_2_). Chemical treatment methods were also used to incorporate nitrogen and oxygen functional groups into the carbons to increase understanding of their role in redox reactions in vanadium flow batteries. This multifactorial study investigates carbon structure–reactivity relationships necessary for understanding and improving electrode performance in vanadium redox flow batteries. The series of carbons used were predominantly ultramicroporous and had very similar porous structures thereby eliminating the porous structure as a factor in the electrochemistry. The fundamental issues are the mechanisms through which changes in carbon molecular structure and the functionalization of carbon surfaces affect the electrochemistry of vanadium under electrolyte and electrode conditions reasonably close to operating conditions but simplified to concentrate on the details of the electrochemistry.

## 2. Experimental

### 2.1. Material preparation

#### 2.1.1. Carbonization studies

Carbons were prepared from walnut shells (particle size ∼1 mm) by pyrolysis in either nitrogen or carbon dioxide at heat treatment temperatures (HTTs) of 600–1000 °C with a heating rate of 8.5 °C min^−1^ to vary the structures of carbon samples. The samples were labeled as X/*T*-h, in which X indicates the carbonization atmosphere (X = N_2_ or CO_2_), *T* is the final pyrolysis temperature in °C, and h is the hold time in hours after the system reached the heat treatment temperature. An example of the nomenclature is carbon CO_2_/800-1 prepared by pyrolysis of the biomass in a CO_2_ atmosphere at an HTT of 800 °C with a 1 h hold time.

#### 2.1.2. K_2_CO_3_-treated carbon

15 g of carbon CO_2_/800-1 was soaked in 50 mL of deionized (DI) water mixed with 15 g K_2_CO_3_ for 24 h. This carbon was dried overnight in an oven at 60 °C and then heat treated at 800 °C for 2 h under a CO_2_ atmosphere (100 mL min^−1^). The carbon was then washed with HCl (0.2 M) and DI water until the pH was ∼7 and then extracted using a Soxhlet apparatus with 400 mL DI water to remove residual water-soluble compounds. This carbon was denoted as CO_2_/800-1-K_2_CO_3_/800.

#### 2.1.3. HNO_3_-treated carbons

10 g of carbon CO_2_/800-1 was refluxed in 200 mL HNO_3_ (7.5 M) at 95 °C for 6 h. This carbon was washed with DI water until the pH was ∼6–7 and then extracted using a Soxhlet apparatus with 400 mL of DI water for at least 72 h to remove residual acid and water-soluble compounds in the AC. This carbon was denoted as CO_2_/800-1-HNO_3_. This carbon was treated at temperatures of 400 and 800 °C in an H_2_ atmosphere (40 mL min^−1^) for 2 h, and the final carbons were named CO_2_/800-1-HNO_3_/400 and CO_2_/800-1-HNO_3_/800, respectively.

#### 2.1.4. NH_3_-treated carbons

1 g of carbon N_2_/800-1 was treated at 600 °C and 800 °C in an NH_3_ atmosphere (40 mL min^−1^) for 2h to give carbons denoted as N_2_/800-1-NH_3_/600 and N_2_/800-1-NH_3_/800, respectively.

### 2.2. Material characterization

#### 2.2.1. Chemical analyses

Elemental analyses for C, H, N, and O were determined by Microanalytical Services, Okehampton, UK. Oxygen analysis was obtained using the Unterzaucher pyrolysis method.^29^

#### 2.2.2. Porous structure characterization

The porous structure was analyzed using CO_2_ adsorption at 0 °C and N_2_ adsorption at −196 °C. The CO_2_ adsorption/desorption isotherms were obtained using an intelligent gravimetric analyser (IGA) supplied by Hiden Isochema Ltd, Warrington, UK. The Dubinin–Radushkevich (DR) ultramicropore volume was obtained from CO_2_ adsorption at 0 °C within the relative pressure range *p*/*p*_0_ = 2.7 × 10^−7^–3 × 10^−2^. N_2_ adsorption and desorption isotherms at −196 °C were recorded using a Thermo Fisher Scientific Surface Analyzer, and the isotherms were used to determine the Brunauer–Emmett–Teller (BET) surface area using the linear region of the BET graph up to *p*/*p*_0_ = ∼0.1. The Dubinin–Radushkevich (DR) micropore volume was obtained from N_2_ adsorption at −196 °C within the relative pressure range up to *p*/*p*_0_ = 3 × 10^−2^. The amount of N_2_ adsorbed at −196 °C and *p*/*p*_0_ = 0.995 was used to calculate the total pore volume.

#### 2.2.3. X-ray photoelectron spectroscopy (XPS)

The spectra were recorded using a Thermo Fisher Scientific K-alpha^+^ spectrometer and a micro-focused monochromatic Al X-ray source (72 W) with an area of ∼400 μm^2^. Spectra were recorded at pass energies of 150 eV for survey scans with 1 eV step sizes and 40 eV for the high-resolution scans with 0.1 eV step sizes. The C 1s spectra were recorded over the binding energy ranges 275–300 or 279–300 eV. The O 1s and N 1s spectra were recorded over the binding energy ranges 525–545 eV and 392–410 eV, respectively. Charge neutralization of the sample was attained using a combination of low-energy electrons and argon ions. Curve fitting analysis was performed in CasaXPS software using a Shirley-type background and Scofield cross sections, with an energy dependence of −0.6.

#### 2.2.4. Temperature programmed desorption (TPD)

The TPD profiles were obtained using a Netzsch Jupiter STA 449C thermogravimetric analyzer interfaced with an Aeolos QMS 403 quadrupole mass spectrometer. The sample was subjected to a series of depressurization/pressurization cycles with He to remove air from the system before analysis. The sample (16.0 ± 0.5 mg) was placed in an Al_2_O_3_ crucible and covered with an aluminum perforated mesh to ensure no sample was lost during the heating procedure. The biochar sample was heated at 10 °C min^−1^ until 1250 °C with He as the carrier gas (35 mL min^−1^). The gas evolution profiles were monitored as a function of temperature for mass/charge (*m*/*z*) ratios: 12, 14, 17, 18, 28, 30, 32 and 44.

#### 2.2.5. Titration studies

The oxygen functional group contents of the carbons were investigated using the acid/base titration method developed by Boehm.^30^ 0.2 g aliquots of carbon were reacted at ambient temperature with 25 mL of NaOH (0.1 N), NaHCO_3_ (0.1 N), Na_2_CO_3_ (0.1 N), and HCl (0.1 N) for 72 h. The excess acid and base were determined by titration with NaOH (0.1 N) and the excess base by titration with HCl (0.1 N), respectively. The concentrations of acidic sites were calculated using the assumptions that NaOH reacts with carboxylic, lactonic, and phenolic groups, Na_2_CO_3_ reacts with carboxylic and lactonic groups, and NaHCO_3_ reacts only with carboxylic groups. The total content of basic carbon sites was determined from titration with HCl (0.1 N).

#### 2.2.6. Raman spectroscopy

A Horiba LabRAM HR Evolution Raman spectrometer was used to analyze the carbonaceous molecular structure (exciting line wavelength, *λ* = 532 nm, source power 455 mW). A non-dispersive filter was used to reduce the power at the sample to ∼1% of the laser power. The samples were examined using an Olympus LMPLFLN20× magnification objective. Each Raman spectrum was obtained as ten accumulations of 10 s acquisitions. OriginPro software was used to curve fit the Raman spectra in the range 800–1800 cm^−1^ (first-order region). The background was subtracted by forming a linear baseline between the spectral regions 800–950 cm^−1^ and 1750–1850 cm^−1^, where the signals were constant.

#### 2.2.7. Fourier transform infrared spectroscopy (FTIR)

The carbon spectra were measured using a PerkinElmer Spectrum 2 Fourier transform infrared spectrometer fitted with a diamond attenuated total reflectance (ATR) accessory. Samples were dried and compacted on the diamond crystal. Spectra were recorded for the 400–4000 cm^−1^ range by averaging 4 scans. The spectral resolution was 4 cm^−1^.

#### 2.2.8. Powder X-ray diffraction (PXRD)

Profiles were measured using a PANalytical X’Pert Pro MPD instrument with a Philips PW3040/60 X-ray source (Cu K_α_ radiation, *λ* = 1.540598 nm) and an X’Celerator* detector. The PXRD profiles were scanned between 5–80° with a scan step of 0.03342°. The Scherrer equation was used to determine the carbon crystallite thickness (*L*_c_) and apparent crystallite diameter (*L*_a_),^31,32^ which are described by the following equations:1
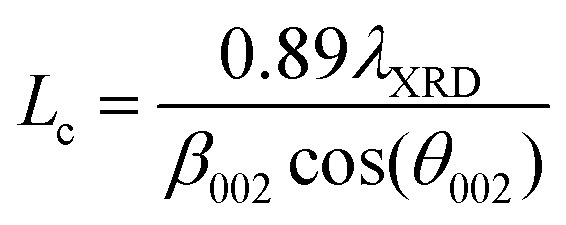
2
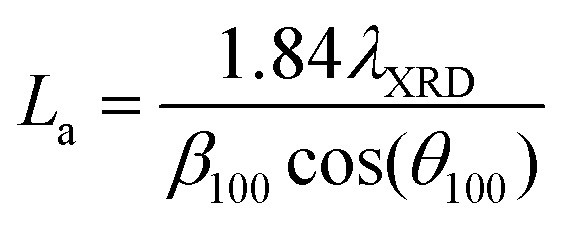
where *λ*_XRD_ is the wavelength of the incident X-ray radiation, *β*_002_ and *β*_100_ are the full-width at half peak maximum of the (002) and (100) peaks, respectively, and *θ*_002_ and *θ*_100_ are the diffraction angles of the (002) and (100) peaks. The interlayer space (*d*_002_) was determined using the Bragg equation:

3 *nλ*_XRD_ = 2*d*_002_ sin(*θ*_002_)

#### 2.2.9. Transmission electron microscopy

Transmission electron microscopy bright-field (TEM-BF), transmission electron microscopy dark-field (TEM-DF), high-resolution TEM (HR-TEM), and electron diffraction were performed on an FEI Tecnai T20 AEM microscope.

### 2.3. Electrochemical measurements

#### 2.3.1. Electrode preparation in nitrogen

A sample of carbon (90 wt%) and polyvinylidene fluoride as the binder (10 wt%) were thoroughly mixed, and a homogeneous slurry was fabricated by dispersing the mixture in *N*-methyl-2-pyrrolidone solution (100 mg mL^−1^). The electrodes were made by coating the slurry (30 μL cm^−2^) on one surface of a 0.6 mm thick expanded bipolar plate (TF6, SGL Carbon). Electrodes had a coated area of 1 cm^2^ (1 cm × 1 cm) and a mass of 0.105 g.

#### 2.3.2. Cyclic voltammetry (CV)

Cyclic voltammetry was recorded using a three-electrode cell with a PARSTAT potentiostat (PMC-1000) using 1.6 M V^3.5+^ in 4.5 M total sulfate electrolyte solution (GFE, Nuremberg, Germany). The counter electrode (CE) was a platinum wire, and the reference electrode (RE) was a saturated mercury electrode in 1 M H_2_SO_4_ (MSE). The applied voltage window was between −1.3 and 1 V *vs.* RE. The CV graphs reported in this article are steady-state CVs obtained after a full cycle scan with the voltage window mentioned above. Scan rates of 5, 10, and 20 mV s^−1^ were investigated, but studies mainly used a scan rate of 20 mV s^−1^. All measurements were carried out under a nitrogen atmosphere at 20 °C.

#### 2.3.3. Electrical impedance spectroscopy (EIS)

A V^4.5+^ solution (50% V^4+^ and 50% V^5+^) was obtained from the commercial 1.6 M V^3.5+^ in a 4.5 M total sulfate electrolyte solution (GFE, Nuremberg, Germany). This solution was electrolyzed in a two-compartment flow cell with a cation exchange membrane (Fumatech, GmbH, Bietigheim, Germany) consisting of PAN-based carbon felt (SIGRACELL GFD 4.6 EA, SGL Carbon) with thickness 4.6 mm and a bipolar plate (TF6, SGL Carbon). The electrolysis studies were carried out under an N_2_ atmosphere to prevent the oxidation of electrolyte ions. A V^4.5+^ solution was used for electrochemical impedance spectroscopy (EIS), and the electrode preparation method used for the CV experiments was also used for EIS. The EIS was also recorded in a three-electrode cell with the same experimental setup used in the CV experiments. An MGP-2 battery test station potentiostat was used to record EIS, using the frequency range from 100 mHz to 20 kHz at an alternating current signal of 10 mV.

### 2.4. Static cell configuration performance

The charge–discharge performance of the carbons was evaluated in a static cell using the procedure reported previously at 20 °C. The PAN-based carbon felt was heat treated at 700 °C (hold time 5 h) in air and allowed to cool down naturally. The treated carbon felt was cut to the precise size of the working electrode 4 cm^2^ pieces (2 × 2 cm) and soaked into 1.6 M V^3.5+^ in a 4.5 M total sulfate electrolyte solution (GFE, Nuremberg, Germany). The AC-coated bipolar plate was in contact with the treated carbon felt and the current collector, similar to the VRFB configuration. The two half-cells were separated with an ion exchange membrane. The static cell setup was assembled carefully, with the thickness of the whole cell measured in the range of 23–24 mm to ensure the forces applied during the preparation of different samples were equal. In this setup, carbon felt, ion exchange, and current collector were kept constant to measure the influence of different activated carbons. Additional details of the cell are included in ESI.†

Static cell charge–discharge was recorded using a PARSTAT potentiostat (PMC-1000) at 25 °C in an environmental chamber. The cell was stabilized for 1 hour before recording. The charge–discharge tests were performed at constant current densities of 10, 15, and 20 mA cm^−2^ in the potential cut-off window of 0.9–1.65 V. The coulombic efficiency (CE), energy efficiency (EE), and voltage efficiency (VE) were calculated using the following equations:4
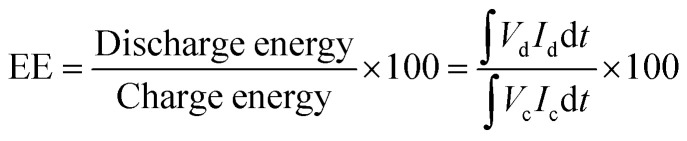
5
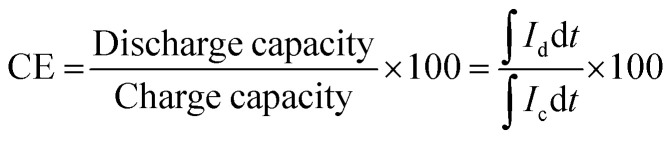
6
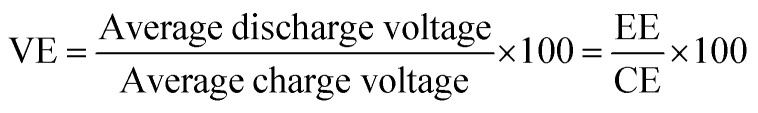
where *V*_d_ and *V*_c_ are discharge and charge voltages, respectively, and *I*_d_ and *I*_c_ are discharge and charge currents, respectively.

## 3. Results and discussion

The structure and surface properties of carbon in carbon/PVDF composite electrodes were varied systematically to study factors and process timescales that influence vanadium redox reactions. The primary factors determining carbon structure are the sustainable biomass carbon precursor and the experimental carbonization conditions, mainly heat treatment temperature (HTT), with secondary experimental factors such as hold time, heating rate, and gaseous atmosphere. This study was limited to walnut shell as the carbon precursor, and the carbonization heating rate was constant. The following carbonization variables were investigated: HTT, hold time, and gaseous atmosphere. Chemical treatment, gasification, and thermolysis processes were also used to modify the carbon surface functionality.

Three series of samples were used in this study to explore the role of carbonization conditions and chemical treatment procedures on structural carbon characteristics and the impact on carbon/PVDF composite electrode properties. These series were as follows:

(1) Series 1: carbonization in a relatively inert nitrogen atmosphere at various HTTs (600–1000 °C)

(2) Series 2: carbonization in a carbon dioxide atmosphere at various HTTs where some partial gasification occurs *via* the Boudouard reaction (700–1000 °C). This results in incorporating some functional groups and activating the porous structure.

(3) Various chemical treatment procedures incorporated functional groups into carbons with HTT 800 °C. The treatment procedures used in this study were (a) oxidation with nitric acid combined with heat treatment in H_2_ up to 800 °C to remove oxygen functional groups progressively,^33,34^ (b) treatment with K_2_CO_3_ at 800 °C, and (c) NH_3_ treatment at 600 and 800 °C.^34,35^ The chemical treatment, gasification, and thermolysis procedures were limited to a maximum of 800 °C so as not to introduce structural differences due to higher carbonization temperatures.

### 3.1. Analytical data

Extensive analytical studies of biomass decomposition in an inert atmosphere to form biochars have established a relationship between the H/C ratio and heat treatment temperature, aromatic clusters, and sorption characteristics. HTT was the main factor, with the H/C ratio decreasing from 1.45–1.8 at 150 °C to 0.2–0.45 at 600 °C. The H/C ratio and HTT relationship have a quantitative reverse sigmoidal shape up to 700 °C, independent of the biochar precursor.^36^Table 1 gives the elemental analysis for carbon, hydrogen, nitrogen, and oxygen on a dry ash-free (daf) basis for the carbons used in this study. The walnut shell used had an ash content of 0.65 wt% db. The ash contents of the carbons were in the range of 0.26–3.86 wt% db. The H/C ratios for carbons N_2_/600-1 and CO_2_/700-1 were 0.29 and 0.26, respectively. These values are within the H/C ranges obtained for the HTTs for biochars prepared from various precursors.^36^ The O/C ratios for carbon N_2_/600-1 (0.055) and carbon CO_2_/700-1 (0.088) were also similar to those of biochars with the same HTT.^37^ The H/C and O/C ratios for Series 1 (N_2_ atmosphere) carbons decrease with increasing HTT (600–1000 °C). In contrast, the H/C and O/C ratios for Series 2 (CO_2_ atmosphere) have a different trend with only small changes in the H/C ratio, and the O/C ratio increases markedly for HTT of 1000 °C. The latter is attributed to the Boudouard reaction increasing the surface oxygen groups. The effect of incorporating oxygen and nitrogen functional groups into the carbon structure was studied by combining chemical treatment, gasification, and thermolysis procedures.

**Table 1:** Elemental and ash analysis for the carbons used in this study

Carbon sample	Elemental content (wt%, dry ash free)				Ash (wt% dry basis)
	C	H	N	O	
N_2_/600-1	90.80 ± 0.77	2.22 ± 0.06	0.29 ± 0.02	6.69 ± 0.41	1.67
N_2_/800-1	88.86 ± 0.32	1.63 ± 0.05	0.32 ± 0.02	9.19 ± 0.24	1.75
N_2_/1000-1	95.53 ± 0.14	0.36 ± 0.03	0.47 ± 0.01	3.64 ± 0.06	3.86
CO_2_/700-1	87.48 ± 0.44	1.90 ± 0.04	0.34 ± 0.01	10.28 ± 0.39	1.55
CO_2_/800-1	90.52 ± 0.21	1.63 ± 0.09	0.31 ± 0.01	7.54 ± 0.12	1.56
CO_2_/1000-1	83.57 ± 0.27	1.85 ± 0.11	0.44 ± 0.01	14.14 ± 0.15	1.97
CO_2_/800-1-K_2_CO_3_	86.82 ± 0.32	1.49 ± 0.10	0.53 ± 0.02	11.16 ± 0.20	0.48
CO_2_/800-1-HNO_3_	72.97 ± 0.25	1.88 ± 0.04	1.31 ± 0.01	23.84 ± 0.21	0.52
CO_2_/800-1-HNO_3_/400	77.90 ± 0.23	2.14 ± 0.03	1.03 ± 0.01	18.93 ± 0.20	0.50
CO_2_/800-1-HNO_3_/800	88.11 ± 0.12	1.89 ± 0.12	0.43 ± 0.01	9.57 ± 0.01	0.40
N_2_/800-1-NH_3_/800	83.02 ± 0.28	1.83 ± 0.01	4.76 ± 0.01	10.39 ± 0.30	0.26

### 3.2. Gas adsorption–desorption characteristics

The gas adsorption characteristics of the carbons used in this study are summarized in Table 2. N_2_ adsorption at −196 °C was used to measure the micropore volume (<2 nm), total pore volume, and BET surface area, while CO_2_ adsorption at 0 °C was used to obtain the ultramicropore volume (<0.7 nm).^38–40^ The N_2_ adsorption at −196 °C was very low compared with CO_2_ adsorption at 0 °C for carbon samples prepared by carbonization under a nitrogen atmosphere (Series 1). Nitrogen adsorption measurements at −196 °C are sometimes unsuitable for ultramicroporous carbons due to activated diffusion effects.^41^ Therefore, CO_2_ adsorption at 0 °C was used to obtain an ultramicropore volume of porous carbons using the Dubinin–Radushkevich (DR) equation. The CO_2_ adsorption and desorption isotherms at 0 °C were reversible and Type I in the IUPAC Classification Scheme^42^ with no hysteresis for carbons N_2_/600-1, N_2_/700-1 and N_2_/800-1 (Fig. S1, ESI†). The DR model fitted the adsorption isotherms for the relative pressure range *p*/*p*_0_ 0–0.03 (Fig. S2, ESI†), indicating that the pore size distributions in the carbons were Gaussian^43^ and the ultramicropore volumes are given in Table 2. The CO_2_ adsorption kinetic profile measurements at 0 °C for the adsorption and desorption isotherms confirmed slow diffusion into the porous structures for carbons N_2_/600-1, N_2_/700-1, and N_2_/800-1. The kinetic profiles were fitted to a stretched exponential model (Fig. S3, ESI†). The kinetics are faster than those reported for CO_2_ adsorption on carbon molecular sieves used for air separation.^44^ However, the higher carbonization temperature for carbon N_2_/1000-1 annealed the carbon structure, and CO_2_ adsorption, even at 30 °C was very slow with equilibration times per isotherm point estimated to be ∼1.5 × 10^5^ s, which prevented isotherm measurements (see ESI†, Fig. S3d). This slow diffusion of CO_2_ into the porous structure confirms activated diffusion effects and has implications for liquid phase diffusion of electrochemical species into the porous structure, which are much slower.

**Table 2:** Pore structure characterization data for carbons obtained from CO_2_ (0 °C) and N_2_ (−196 °C) adsorption isotherms

Carbon sample	SA CO_2_ (m^2^ g^−1^)	DR V CO_2_ (cm^3^ g^−1^)	SA_BET_ (m^2^ g^−1^)	TPV N_2_ (cm^3^ g^−1^)	DR VN_2_ (cm^3^ g^−1^)	*V*_meso_ (cm^3^ g^−1^)
N_2_/600-1	501 ± 5	0.1885 ± 0.0017	n.d.	n.d.	n.d.	n.d.
N_2_/700-1	544 ± 5	0.2044 ± 0.0018	n.d.	n.d.	n.d.	n.d.
N_2_/800-1	591 ± 5	0.2223 ± 0.0018	n.d.	n.d.	n.d.	n.d.
CO_2_/700-1	607 ± 3	0.228 ± 0.001	502.9 ± 1.5	0.279	0.1958 ± 0.0059	0.0828
CO_2_/800-0	611 ± 4	0.2298 ± 0.0014	482.3 ± 1.1	0.254	0.1887 ± 0.0074	0.0657
CO_2_/800-1	632 ± 4	0.2376 ± 0.0014	444.8 ± 1.1	0.266	0.1806 ± 0.0012	0.0848
CO_2_/800-3	642 ± 4	0.2414 ± 0.0016	569.7 ± 1.7	0.322	0.2259 ± 0.0006	0.0963
CO_2_/1000-1	784 ± 16	0.2948 ± 0.0059	686.4 ± 2.5	0.380	0.2712 ± 0.0008	0.1087
CO_2_/800-1-K_2_CO_3_/800	839 ± 10	0.3140 ± 0.0037	673.5 ± 1.7	0.334	0.2667 ± 0.0007	0.0669
CO_2_/800-1-HNO_3_	550 ± 8	0.2066 ± 0.0031	583.3 ± 1.2	0.307	0.2229 ± 0.0010	0.0844
CO_2_/800-1-HNO_3_/400	658 ± 4	0.2475 ± 0.0015	559.6 ± 1.6	0.346	0.2172 ± 0.0007	0.1282
CO_2_/800-1-HNO_3_/800	650 ± 13	0.2444 ± 0.0050	725.7 ± 4.8	0.356	0.2884 ± 0.0032	0.0672
N_2_/800-1-NH_3_/600	567 ± 5	0.2132 ± 0.0020	n.d.	n.d.	n.d.	n.d.
N_2_/800-1-NH_3_/800	687 ± 14	0.2584 ± 0.0054	708.7 ± 1.7	0.337	0.2734 ± 0.0008	0.0634

In contrast to the series of carbons prepared under a nitrogen atmosphere (Series 1), the corresponding carbons prepared in a CO_2_ atmosphere (Series 2) adsorb nitrogen at −196 °C and have a Type I/Type II isotherm with a steep uptake at low *p*/*p*_0_ corresponding to micropore filling (Fig. S4, ESI†). The N_2_ isotherms at −196 °C have minimal adsorption/desorption hysteresis (Type H4 in the IUPAC classification scheme)^42^ consistent with small amounts of mesoporosity in the carbons. The hysteresis loops close at *p*/*p*_0_ ∼0.4–0.45, corresponding to a pore size of ∼4 nm. The mesopore size distributions are shown in Fig. S4 and S9 (ESI†). All the samples have very similar mesopore size distributions with peaks in the range 3.5–4 nm with a sharp decrease in the pore diameter range 4–5 nm. The mesopore distributions show that all carbon samples have only a minimal mesoporosity <6 nm. Both BET and DR graphs were linear (Fig. S5 and S6, ESI†), and the surface area and micropore volumes are given in Table 2. The N_2_ DR micropore volumes cover the pore size range <2 nm, whereas the CO_2_ ultramicropore volume covers <0.7 nm.^38–40^ Carbon CO_2_/1000-1 had a high DR CO_2_ micropore volume of 0.2948 ± 0.0059 cm^3^ g^−1^ due to partial gasification activating the porous structure, whereas carbon N_2_/1000-1 had very narrow porosity due to thermal annealing. The DR CO_2_ graphs are linear (Fig. S2 and S8, ESI†), and CO_2_ micropore volumes increased with increasing carbonization temperature, from 600 °C to 800 °C for carbons prepared under both nitrogen and carbon dioxide atmospheres, and this is attributed to the development of porous carbon structure with loss of volatiles in the case of N_2_ atmosphere and in addition, some gasification in a CO_2_ atmosphere, which increases with increasing temperature. The N_2_ DR micropore volumes (<2 nm) are 76–95% of the corresponding DR CO_2_ micropore volumes (<0.7 nm) for Series 2 carbons made by carbonization in a CO_2_ atmosphere. The values are closer to 1 for higher HTT and longer hold time, indicating an increasing activation level. This can be attributed to activated diffusion limitations in the ultramicroporosity.

Carbon CO_2_/800-1 was used as a starting carbon material for functionalization studies. K_2_CO_3_ was used to modify the structure of carbon CO_2_/800-1. This treatment increased the DR CO_2_ ultramicropore volume from 0.2376 to 0.3140 cm^3^ g^−1^ (Fig. S8 and S13, ESI†). The corresponding total pore volumes (TPV_N_2__) increased from 0.266 cm^3^ g^−1^ to 0.334 cm^3^ g^−1^ (Fig. S4 and S9, ESI†). The BET surface area increased from 444.8 m^2^ g^−1^ for carbon CO_2_/800-1 to 673.5 m^2^ g^−1^ for carbon CO_2_/800-1-K_2_CO_3_ (Fig. S5 and S10, ESI†). K_2_CO_3_ has been reported as an activating agent for increasing the porosity of carbons.^46,47^

Oxidation with HNO_3_ increased the DR N_2_ micropore volumes from 0.1806 cm^3^ g^−1^ for CO_2_/800-1 to 0.2229 cm^3^ g^−1^ CO_2_/800-1-HNO_3_ indicating an increase in ultramicroporosity. Heat treatment of CO_2_/800-1-HNO_3_ decomposed the labile functional groups, giving CO_2_/800-1-HNO_3_/400, which had a DR N_2_ micropore volume of 0.2172 cm^3^ g^−1^. Carbon CO_2_/800-1-HNO_3_/800 had a DR N_2_ micropore volume of 0.2884 cm^3^ g^−1^, indicating that a combination of oxidation and heat treatment increases microporosity. The CO_2_ ultramicropore volumes for these samples were similar, with no clear trend. Oxidation of carbon CO_2_/800-1-HNO_3_ gave carbons with N_2_ total pore volumes in the order.CO_2_/800-1 < CO_2_/800-1-HNO_3_ ∼ CO_2_/800-1-HNO_3_/400 < CO_2_/800-1-HNO_3_/800.

There is minimal adsorption–desorption hysteresis in the N_2_ adsorption isotherms at −196 °C (Fig. S9, ESI†). It is evident that there is a small increase in microporosity in the series, but the overall changes in porous structure are relatively small.

High-temperature gas phase NH_3_ treatment incorporates nitrogen into the carbon structure.^48–50^ Carbon N_2_/800-1 does not adsorb significant N_2_ at −196 °C due to activated diffusion in very narrow ultramicroporosity. NH_3_ treatment of carbon N_2_/800-1 at 600 °C reduced CO_2_ DR micropore volume from 0.2223 cm^3^ g^−1^ (N_2_/800-1) to 0.2132 cm^3^ g^−1^ (N_2_/800-1-NH_3_/600) (Table 2). N_2_ adsorption at −196 °C is limited by activated diffusion effects, as shown by the higher CO_2_ adsorption at 0 °C and the slow diffusion of CO_2_ into the porous structure (Fig. S3, ESI†). However, the higher treatment temperature at 800 °C led to gasification reactions, increasing the CO_2_ DR ultramicropore volume to 0.2584 cm^3^ g^−1^ and the N_2_ DR micropore volume of 0.2734 cm^3^ g^−1^. The total pore volume was 0.337 cm^3^ g^−1^, and the carbon is predominantly ultramicroporous.

BET surface areas were determined from the linear region of the BET graph that included point B, which covered adsorption data up to a maximum *p*/*p*_0_ of 0.07–0.12 (Fig. S10, ESI†). The *C* parameter in the BET equation was very high (>1000 in all cases), indicating significant filling of micropores. The BET surface areas are very strongly correlated with the DR N_2_ micropore volume (*R*^2^ = 0.99) (Fig. S14b, ESI†) but weakly with the N_2_ total pore volume (*R*^2^ = 0.74) (Fig. S14a, ESI†). The ratios of DR N_2_ micropore volume to CO_2_ ultramicropore volume were in the range of 89–114% for chemical treatment procedures. The carbons prepared in a CO_2_ atmosphere had ultramicropore volumes >75% of the corresponding total pore volume. However, activated diffusion effects for carbons prepared in a nitrogen atmosphere did not allow a total pore volume to be measured using N_2_ adsorption at −196 °C. The functionalized carbons had ultramicropore volumes that were >67% of the total pore volume. Carbons prepared in a CO_2_ atmosphere (Series 2) had ultramicropore volumes >75% of the total pore volumes. All the carbons used in this study were predominantly ultramicroporous (<0.7 nm). Therefore, differences in the porous carbon structures in both these series of carbons do not contribute significantly to the CV characteristics of vanadium redox reactions due to diffusion limitations in the liquid phase.

### 3.3. Functional group characterization

Characterization of functional groups in heterogenous amorphous carbons requires a range of techniques. XPS provides surface analysis of the carbon to a depth of ∼5 nm^51^ but requires curve resolution to quantify the components. ATR covers depths of 0.1 to 1 μm depending on wavelength,^52^ and the spectra of carbons with HTTs of 800 °C are very weak and broad. Titration measurements provide information on oxygen functional groups (carboxyl, anhydrides, lactone, lactol, and phenolic) accessible to aqueous solutions.^30^ Temperature-programmed desorption assumes that functional groups decompose to a specific product. However, this is not always the case since surface species can react. TPD and chemical analysis give information on functional groups in bulk samples. The carbons used in this study were primarily microporous, and the functional group analytical methods provided information on different sampling depths and environments. These issues must be considered when comparing characterization data from various characterization techniques.

#### 3.3.1. X-ray photoelectron spectroscopy (XPS)

The XPS surface elemental analyses (at%) obtained from the survey scan of the samples used in this study are given in Table 3. Minimal amounts of K, Ca, and Si (average <2 at% for Series 1 and 2 carbons and absent from the functionalized carbons except carbon N_2_/800-1-NH_3_/800 (0.28 at%)) originating from the walnut shell were also detected. These values are similar to the ash contents for the bulk samples reported in Table 1. Potassium was the primary metal on the surface (see Table 3). A comparison of XPS O 1s peaks with potassium-containing reference materials^53^ showed that none could be identified in the XPS spectra of the carbons. Therefore, the XPS O 1s spectra could be used without possible interference of peaks from metal oxide species. Therefore, oxygen surface groups in the carbons were analyzed using both O 1s and C 1s spectra. The C 1s spectra represent carbon functional groups, whereas, for O 1s spectra, some oxygen functional groups have two oxygen types, contributing to different XPS O 1s peaks. Binding energy ranges exist for each functional group because of the varying structural environments in heterogeneous carbons.

**Table 3:** Elemental surface analysis (at%) obtained from XPS for carbons

Carbon sample	Atomic content (at%)			
	C	N	O	Total K, Ca, and Si
N_2_/600-1	89.63	0.0	9.12	1.25
N_2_/600-3	89.53	0.0	9.19	1.28
N_2_/700-1	89.89	0.0	8.22	1.89
N_2_/800-1	90.01	0.31	8.10	1.58
N_2_/800-3	89.83	0.0	8.35	1.82
N_2_/1000-1	89.86	0.0	8.43	1.72
CO_2_/700-1	89.87	0.0	8.39	1.74
CO_2_/700-3	91.63	0.0	6.87	1.5
CO_2_/800-1	89.98	0.0	8.3	1.72
CO_2_/800-3	90.61	0.0	7.7	1.69
CO_2_/1000-1	88.53	0.0	8.96	2.51
CO_2_/800-1-K_2_CO_3_/800	92.93	0.0	7.07	0
CO_2_/800-1-HNO_3_	85.7	0.77	13.54	0
CO_2_/800-1-HNO_3_/400	90.27	1.33	8.4	0
CO_2_/800-1-HNO_3_/800	96.82	0.33	2.85	0
N_2_/800-1-NH_3_/600	94.69	2.56	2.76	0
N_2_/800-1-NH_3_/800	93.1	4.86	1.8	0.28

The C 1s, O 1s, and N 1s spectra were analyzed using curve fitting to determine the distribution of surface species as the percentage area of the total area under the peak. The quality of the curve fitting is critical to obtaining accurate distributions of surface groups.^54^ The normalized residuals (*R*_*i*_) are defined as follows.

7 *R*_*i*_ = (Calculated intensity − Experimental intensity)/(√(Experimental intensity))

Comparison of the normalized residuals, experimental data, and curve fitting of components for the XPS spectra are shown in Fig. 3–5, providing a visualization of the quality of the curve fitting. The Abbe criterion was also used to analyze the residuals. The Abbe criterion is defined by the equation below.8
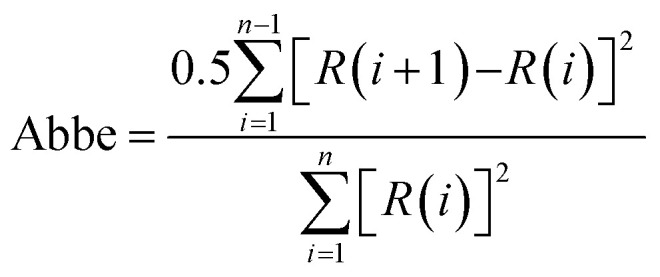


*R*(*i*) and *R*(*i* + 1) are the residuals for the *i* and *i* + 1 points, respectively, and *n* is the number of data points. Random noise and statistically distributed residuals should give Abbe = 1. The curve fitting results for the spectra are provided in ESI† (Tables S4 and S5, ESI†), which includes details of the *χ*^2^ parameters obtained from the Casi software, the Abbe criteria, and repeatability information.

Carbons are complex heterogeneous materials with a wide range of functional groups located at the edge of graphene layers in varying chemical environments, and carbon has amphoteric characteristics. The functional groups include carboxylic anhydride, phenol, hydroquinone, lactone, quinone, and pyrone.^30^

The surface oxygen functional group species are only minor components, typically 7–10% of the C 1s profiles for Series 1 and 2 carbons and are observed on the high energy side of the asymmetric C 1s peak. The C 1s XPS spectra of a wide range of carbons have peaks in the following ranges: C sp^2^ (284.2–284.6 eV), C sp^3^ (284.8–285.4 eV), C–O groups (phenols and ethers), 285.9–286.6 eV, carbonyl (ketones and quinones) and carbons bonded to two oxygens at 286.7–287.5 eV and carboxyl, carboxylic anhydrides, and esters (O–C=O) at 288.3–288.9 eV and shake-up peaks of carbon in aromatic compounds (π–π* transition) at 290–294 eV.^55^ These values are consistent with curve-fitting protocols for organic compounds.^56^

The C 1s spectra for Series 1 carbons (N_2_/700–1 to N_2_/1000-1) showed that the functional group distributions were similar: C–O/OH (58.0 ± 2.2% at 286.49 ± 0.06 eV), C=O (27.0 ± 1.7% at 287.94 ± 0.11 eV) and O–C=O (15.0 ± 1.6% at 289.34 ± 0.27 eV). Series 2 carbons (CO_2_/700-1 to CO_2_/1000-1) also had very similar oxygen functional group distributions: CO/OH (58.7 ± 2.6% at 286.51 ± 0.03 eV), C=O (26.6 ± 1.6% at 288.0 ± 0.1 eV) and O–C=O (14.7 ± 1.9% at 288.96 ± 0.30 eV). Therefore, it is apparent that there are no significant differences observed in C 1s spectra of oxygen functional group distributions between the two series of carbons in the HTT range 700–1000 °C.

Carboxylic, anhydrides, lactones, esters, and pyrone groups present in carbons have both C–O bonds and C=O bonds, and these groups will contribute to different peaks in the O 1s spectra. Two peaks were observed in the XPS spectrum of polymer polyethylene terephthalate (PET) containing carboxyl/ester groups where the O=C peak occurs at 531.6 eV and the O–C peak at 533.2 eV.^57,58^ The O 1s peaks for both Series 1 and 2 carbons were curve fitted using five peaks: (1) carbonyl, lactone, and ester C=O (531.0–531.6 eV), (2) C–O ether and hydroxyl bonded to aliphatic (532.5–532.9 eV), (3) C–O ether and OH bonded to aromatic carbon, O–C in carboxyl/ester C–O aromatic (533.0–533.6 eV), (4) chemisorbed water/oxygen (534.8–536.2 eV) and (5) a very weak peak was also observed at 537.4 ± 0.5 eV.^55,59–62^ Peaks 4 and 5 were 3–8% of the total O 1s peak area and did not show systematic changes with HTT or in functionalization studies. Therefore, these peaks are not discussed in detail.

Series 1 carbons (N_2_/700-1 to N_2_/1000-1) had very similar oxygen functional group distributions based on the O 1s spectra: C=O (35.6 ± 3.4% at 531.08 ± 0.05 eV), C–OH (36.5 ± 2.1% at 532.35 ± 0.12 eV) and O–C in carboxyl/ester groups (23.5 ± 2.1% at 533.57 ± 0.12 eV). There were also two weaker peaks, with 3.5 ± 0.2% at 535.30 ± 0.34 eV and 1.3 ± 0.3% at 537.30 ± 0.83 eV. Series 2 carbons (CO_2_/700-1 to CO_2_/1000-1) also had very similar functional group distributions based on the O 1s spectra: C=O (32.5 ± 3.6% at 531.07 ± 0.07 eV), C–O aliphatic (35.4 ± 1.3% at 532.26 ± 0.11 eV), and O–C aromatic (26.8 ± 2.9% at 533.5 ± 0.1 eV). The weaker peaks at higher energies were at 3.8 ± 0.6% at 535.22 ± 0.27 eV and 1.5 ± 0.4% at 537.04 ± 0.54 eV. Comparisons of the shapes of the O 1s XPS spectra for Series 1 and 2 carbons for HTTs 700–1000 °C are shown in ESI,† Fig. S23. These comparisons show that the functional group distributions are similar. Fig. S22 (ESI†) shows that carbonization hold time did not influence oxygen surface groups in Series 1 and 2 carbons. There is agreement from the C 1s and O 1s spectra that the Series 1 and 2 carbons with HTTs in the range 700–1000 °C have very similar oxygen functional group distributions irrespective of HTT, carbonization atmosphere, and hold time.

The oxygen functional distributions from the C 1s spectra for carbons N_2_/600-1 and N_2_/600-3 were not statistically different based on the C 1s spectra, but the O 1s spectra did show minor differences. The O 1s spectra show a decrease from 33.6 ± 0.6% for carbons N_2_/600-1 and N_2_/600-3 to 23.5 ± 2.1% in the O–C aromatic groups at 533.75 eV and an increase from 24.7 ± 0.6 for N_2_/600-1 and N_2_/600-3 to 35.5 ± 3.4% for HTT 700–1000 °C in the 531.2 eV carbonyl peak. This difference is consistent with the small loss of O–C bonded to aromatics in carboxyl, lactone, *etc.*, and the increase in various carbonyl groups over the temperature range of 600–700 °C.

**Fig. 1:**
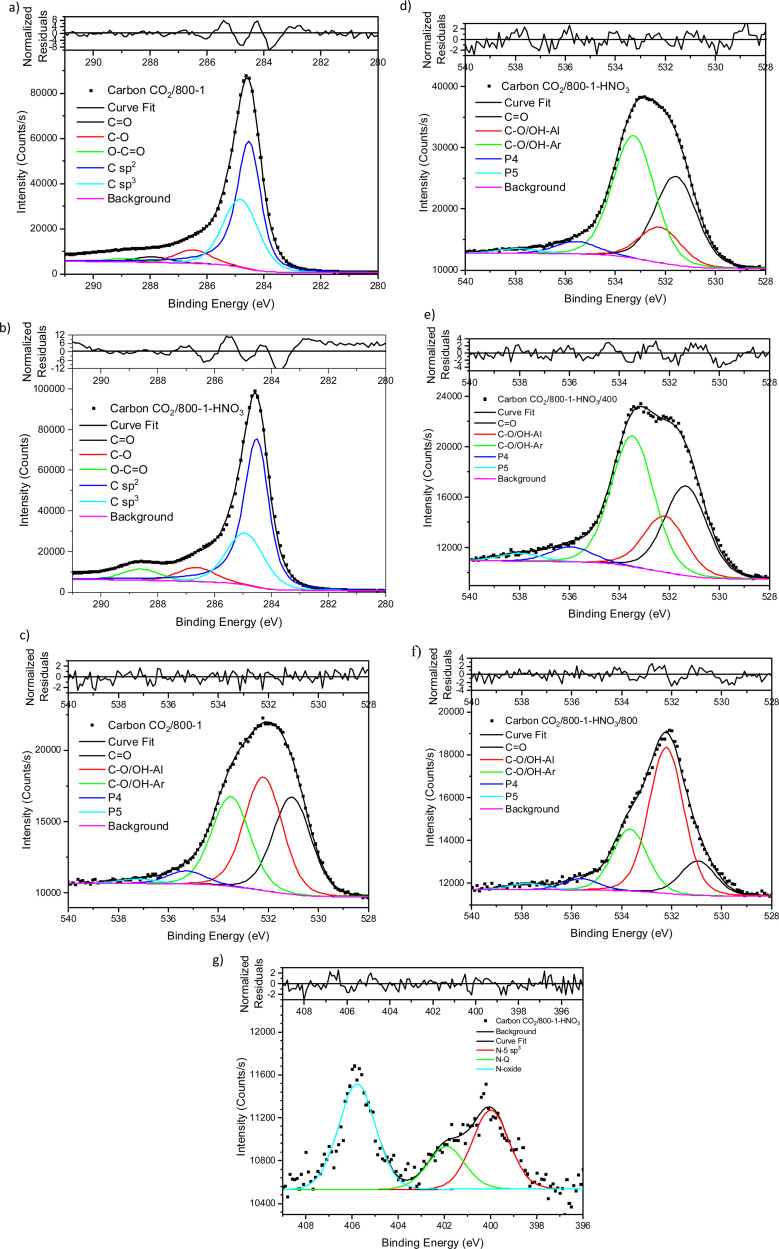
Comparison of XPS spectra, curve fitting, and residuals of carbons (a) C 1s, carbon CO_2_/800-1 (b) C 1s, carbon CO_2_/800-1-HNO_3_, (c) O 1s, carbon CO_2_/800-1, (d) O 1s, carbon CO_2_/800-1-HNO_3_, (e) O 1s, carbon CO_2_/800-1-HNO_3_/400 and (f) O 1s, carbon CO_2_/800-1-HNO_3_/800 and (g) N 1s, carbon CO_2_/800-1-HNO_3_,.

Carbons CO_2_/800-1 and N_2_/800-1 were used as precursors for treatment to incorporate functional groups into the carbon structure. HNO_3_ treatment of carbon CO_2_/800-1 mainly incorporates oxygen functional groups into the carbon structure. The total surface oxygen of carbon CO_2_/800-1 increased from 8.1 at% to 13.54 at% for CO_2_/800-1-HNO_3_ (see Table 3). In comparison, elemental analysis of the bulk samples gave 7.54 wt% for CO_2_/800-1 and 23.84 wt% in CO_2_/800-1-HNO_3_ (see Table 1). The XPS C 1s and O 1s spectra of carbons CO_2_/800-1 and CO_2_/800-1-HNO_3_ are compared in Fig. 1a and b. Comparison of the C 1s spectra shows that the functional group distribution has a marked increase in the O–C=O peak at ∼288.8 eV from ∼15.4% in carbon CO_2_/800-1 to 44.5 ± 0.3% in carbon CO_2_/800-1-HNO_3_ and a marked decrease in the C=O peak at 287.9 eV from 24.5% to <0.1% (Table S4 and S5, ESI†). The C–O peak at 286.5 eV only decreased slightly in intensity for 60 to 55.5% of the distribution. These changes are consistent with the incorporation of carboxylic groups. Furthermore, the O 1s spectra showed that HNO_3_ treatment resulted in the O–C (ether and hydroxyl groups bonded to aromatics) peak at 533.3 eV increased from 28.2% (2.3 at%) in carbon CO_2_/800-1 to 46.93 ± 0.45% (6.4 at%) in carbon CO_2_/800-1-HNO_3_, while C–O aliphatic peak at 532.2 eV decreased from 35.69 (2.96 at%) to 13.3 ± 0.66% (1.80 at%) (Table S5b, ESI†). The corresponding C=O peak distribution only increased slightly in carbon CO_2_/800-1-HNO_3_, but since the surface oxygen content is much higher in carbon CO_2_/800-1-HNO_3_, the C=O peak corresponded to 4.5 at% compared with 2.5 at% in carbon CO_2_/800-1. The XPS results show increases in carboxylic, anhydride, and lactone groups.

Heat treatment of carbon CO_2_/800-1-HNO_3_ decreased the surface oxygen content from 13.54 at% for carbon CO_2_/800-1-HNO_3_ to 8.4 at% for carbon CO_2_/800-1-HNO_3_/400, and to 2.85 at% for CO_2_/800-1-HNO_3_/800 (Table 3). The XPS survey scan showed that metal species were absent (see Table 3). Therefore, possible O 1s peaks from residual inorganic species do not contribute to the O 1s spectra. The C 1s spectra showed that heat treatment of carbon CO_2_/800-1-HNO_3_ to 800 °C to form carbon CO_2_/800-1-HNO_3_/800 increased the C–O/C–OH peak distribution from 55.5% to 93% of the oxygen functionality and decreased the O–C=O peak from 44.5% to 6.7%. These results are consistent with the decomposition of carboxylic groups and the formation of C–O/OH during heat treatment. The O 1s spectra showed that heat treatment of carbon CO_2_/800-1-HNO_3_ decreased the C=O peak at 531.5 eV and the larger peak in the O–C attached to aromatic groups at 533.3 eV and a marked increase in the C–O bonded to aliphatic carbon at 532.2 eV. This change in the distribution of XPS peaks is consistent with the decomposition of carboxylic and the formation of phenolic and ether groups. The O 1s spectra for this series show evidence for the component peaks as shoulders (Fig. 1d–f), thereby supporting the curve resolution scheme. Thermally labile carboxylic groups in CO_2_/800-1-HNO_3_ are also confirmed by the loss of CO_2_ in TPD, as discussed later.

HNO_3_ oxidation results in the incorporation of small amounts of thermally labile nitrogen species (0.8 at%) into the carbon structure (Fig. 1g) that decomposes on heat treatment (Fig. S27 and S28, ESI†). The XPS N 1s spectrum for carbon CO_2_/800-1-HNO_3_ was curve-fitted, revealing peaks at 399.9, 401.9, and 405.8 eV. The most prominent XPS peak (47.1 ± 0.7%) at 405.8 eV was tentatively assigned to *N*-oxide.^63,64^ The other N 1s peaks were at 399.9 eV (33.2%) assigned to pyrrolic or N sp^3^ nitrogen and 401.9 eV (19.7 ± 0.8%) assigned to quaternary nitrogen, but there was no evidence for significant amounts of pyridinic nitrogen. The TPD supports the *N*-oxide assignment, which showed NO desorption at HTT <400 °C (see TPD section). The N 1s peak at 398.5 eV (21.5%) in carbon CO_2_/800-1-HNO_3_/400 was assigned to N sp^2^ (pyridinic groups). The peak at 399.5 eV (49.5%) and 400.6 eV (21.1%) were assigned to pyrrolic, with the peak at 402.3 eV (8%) to quaternary nitrogen by analogy with high-temperature carbons, but other labile nitrogen species could also contribute to the peaks. The decomposition of thermally labile surface groups was evident from the absence of the 405.8 eV *N*-oxide peak in the XPS N 1s spectrum of CO_2_/800-1-HNO_3_/400 (Fig. S27b, ESI†) and the absence of nitrogen surface species in the N 1s XPS spectra of CO_2_/800-1-HNO_3_/800 (Fig. S27c, ESI†).

**Fig. 2:**
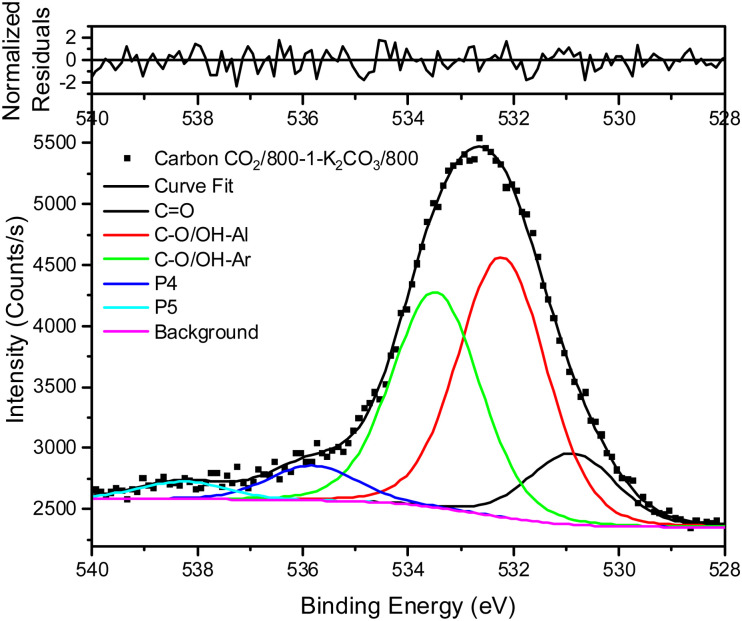
XPS O 1s spectrum, curve fitting, and residuals of carbon CO_2_/800-1-K_2_CO_3_/800.

Treatment of carbon CO_2_/800-1 with K_2_CO_3_ at 800 °C resulted in a small decrease in surface oxygen content from 8.3 to 7.1 at%. The XPS survey scan showed that metal species and residual K_2_CO_3_ were absent (see Table 3). A comparison of the XPS C 1s spectra does not show any apparent changes in the distribution of oxygen surface species. However, the O 1s spectrum showed a decrease in the C=O peak at ∼531 eV from 30.6% in carbon CO_2_/800-1 to 11.7% in carbon CO_2_/800-1-K_2_CO_3_/800. This change was accompanied by smaller increases in the intensities of C-O/C-OH bonded to aliphatic (532.2 eV) and O–C/OH bonded to aromatics (533.5 eV) (Fig. 1c and 2, and Table S5b, ESI†). The titration results showed increased acidic character in aqueous solution, attributed to increased phenolic and carboxylic groups. The TPD results show that more thermally labile oxygen groups in the carbon structure gave CO_2_ evolution starting at ∼550 °C.

**Fig. 3:**
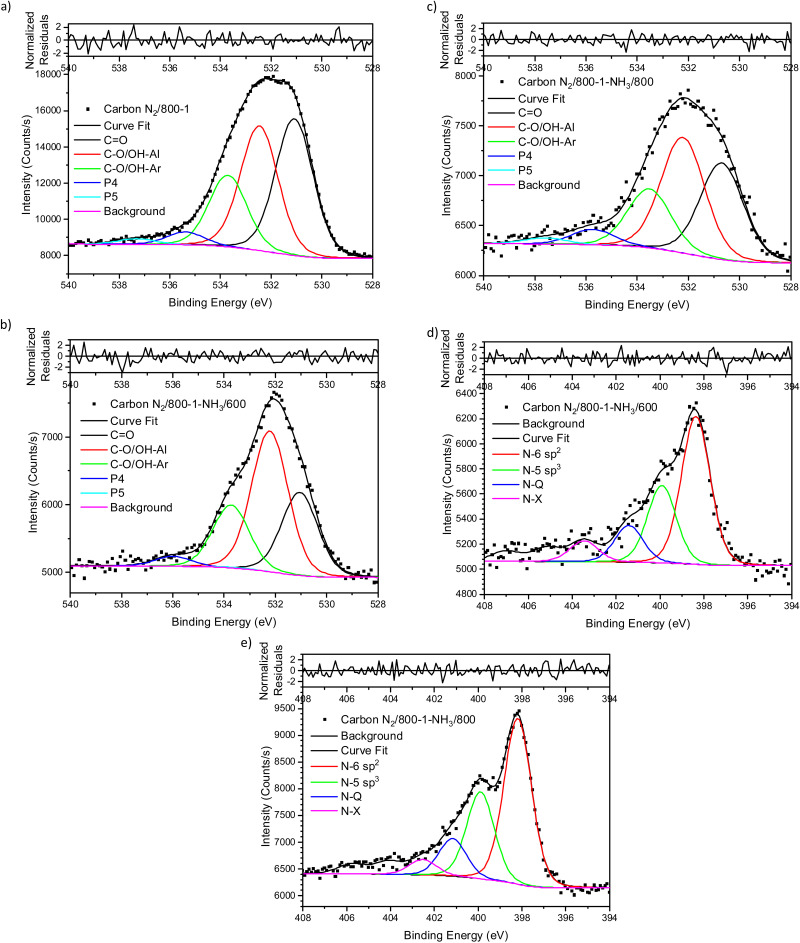
Comparison of XPS spectra, curve fitting, and residuals of untreated carbon and NH_3_ treated carbons (a) O 1s, carbon N_2_/800-1, (b) O 1s, carbon N_2_/800-1-NH_3_/600 (c) O 1s, carbon N_2_/800-1-NH_3_/800, (d) N 1s, carbon N_2_/800-1-NH_3_/600 and (e) N 1s, carbon N_2_/800-1-NH_3_/800.

The XPS spectrum of carbon N_2_/800-1 shows that surface nitrogen species are minimal (see Fig. S28b and Table 3, ESI†) and the O 1s and N 1s spectra for N_2_/800-1-NH_3_/600 and N_2_/800-1-NH_3_/800 are shown in Fig. 3. NH_3_ treatment of carbon N_2_/800-1 increased surface nitrogen content to 2.56 at% in carbon N_2_/800-1-NH_3_/600 and 4.86 at% in carbon N_2_/800-1-NH_3_/800. NH_3_ treatment also reduced the surface oxygen content from 8.1 at% for the precursor (carbon N_2_/800-1) to 2.7 for N_2_/800-1-NH_3_/600 and 1.8 at% N_2_/800-1-NH_3_/800 (Table 3). The nitrogen content obtained by chemical analysis of N_2_/800-1-NH_3_/800 was similar (4.76 wt%) to the XPS surface concentration (4.86 at%). However, the surface oxygen content was 1.8 at%, which is much lower than 10.39 wt% oxygen from chemical analysis, indicating differences between the surface and bulk of the sample. Titration studies discussed later indicated that carbon N_2_/800-1-NH_3_/800 had some weakly acidic groups.

**Fig. 4:**
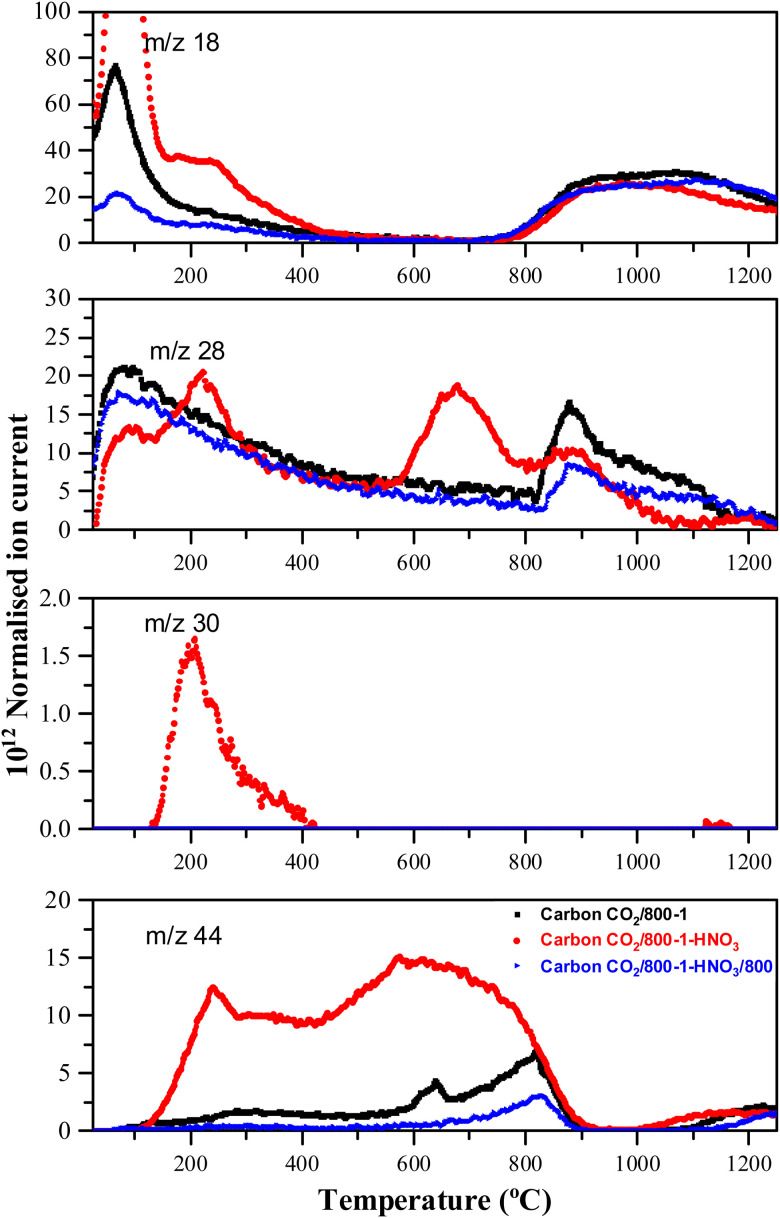
Comparison of TPD profiles of carbon CO_2_/800-1 and HNO_3_ oxidized and heat-treated carbons (CO_2_/800-1-HNO_3_ and CO_2_/800-1-HNO_3_/800) in He (35 mL min^−1^) at 10 °C min^−1^.

**Fig. 5:**
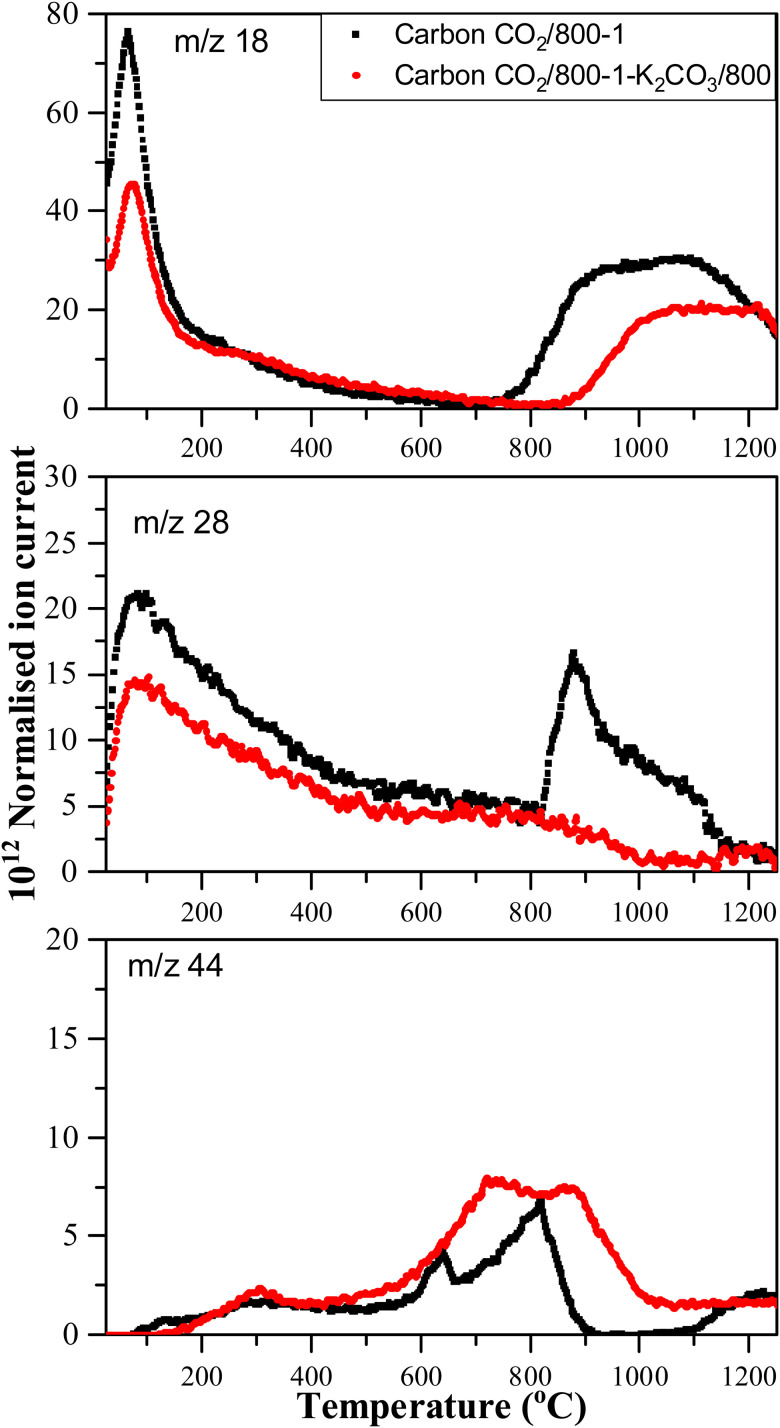
Comparison of TPD profiles of carbons CO_2_/800-1 and CO_2_/800-1-K_2_CO_3_/800 in He (35 mL min^−1^) at 10 °C min^−1^.

Curve fitting was used to analyze the C 1s, O 1s, and N 1s XPS spectra. The C 1s spectra showed that the distribution of oxygen surface groups was not changed very much by NH_3_ treatment at 600 and 800 °C (Table S5a, ESI†). However, the XPS O 1s spectra showed that NH_3_ treatment changed the distribution of surface oxygen species, as shown by the relative intensities of the C=O (531 eV) and C–O attached to aliphatic (532.2 eV) peaks (Table 4). The XPS N 1s showed that pyridinic and pyrrolic groups were the two primary forms of nitrogen, and the N-6/N-5 ratios (∼2 : 1) were similar for both carbons (Table 5). Previous studies of the high-pressure carbonization of pure organic compounds with well-defined pyridinic and pyrrolic nitrogen groups as a function of HTT have shown^63^ that the order of stability of surface groups is as follows:quaternary > pyridinic > pyrrolic

**Table 4:** Oxygen functional group surface analysis from XPS for the carbon samples used in this study

Carbon sample	Components from O 1s profiles obtained from curve resolution				
	Peak 1	Peak 2	Peak 3	Peak 4	Peak 5
	531.12 ± 0.23 eV	532.27 ± 0.11 eV	533.53 ± 0.15 eV	535.49 ± 0.31 eV	537.49 ± 0.58 eV
	C=O carbonyl carboxylic	C–O/OH-aliphatic	CO/OH-aromatic	Chemisorbed H_2_O/O_2_	π–π*
N_2_/600-1	2.29	3.35	3.10	0.26	0.12
N_2_/600-3	2.23	3.54	3.05	0.24	0.12
N_2_/700-1	2.98	3.13	1.80	0.26	0.06
N_2_/800-1	3.10	2.78	1.83	0.28	0.11
N_2_/800-3	2.74	3.16	2.03	0.30	0.12
N_2_/1000-1	2.64	3.20	2.17	0.30	0.11
CO_2_/700-1	2.46	3.03	2.46	0.28	0.15
CO_2_/700-3	2.50	2.27	1.66	0.32	0.12
CO_2_/800-1	2.54	2.96	2.34	0.34	0.12
CO_2_/800-3	2.28	2.76	2.25	0.28	0.13
CO_2_/1000-1	3.25	3.26	2.08	0.29	0.08
CO_2_/800-1-K_2_CO_3_/800	0.83	3.09	2.56	0.40	0.19
CO_2_/800-1-HNO_3_	4.54	1.80	6.35	0.60	0.24
CO_2_/800-1-HNO_3_/400	2.52	1.58	3.67	0.42	0.22
CO_2_/800-1-HNO_3_/800	0.38	1.60	0.68	0.12	0.07
N_2_/800-1-NH_3_/600	0.77	1.30	0.60	0.09	0.00
N_2_/800-1-NH_3_/800	0.58	0.69	0.35	0.09	0.04

**Table 5:** XPS N 1s profiles of carbons N_2_/800-1-NH_3_/600, N_2_/800-1-NH_3_/800, CO_2_/800-1-HNO_3_ and CO_2_/800-1-HNO_3_/400

Carbon sample	Content of each N component						
	N-6/N sp^2^ pyridinic	N-5/N sp^3^ pyrrolic	N-5/N sp^3^ pyrrolic	N–Q	N–X	*N*-Oxide	Total N content
Energy (eV)	398.3	399.9		401.2			
N_2_/800-1-NH_3_/600 (at%)	1.24	0.65		0.31	0.37		2.56
N_2_/800-1-NH_3_/800 (at%)	2.41	1.30		0.56	0.60		4.86
Energy (eV)		399.9		401.9		405.8	
CO_2_/800-1-HNO_3_ (at%)		0.26		0.15		0.36	0.77
Energy (eV)	398.6	399.6	400.6	402.3			
CO_2_/800-1-HNO_3_/400 (at%)	0.29	0.66	0.28	0.11	—	—	1.33

NH_3_ treatment of carbon N_2_/800-1 incorporated mainly pyridinic groups with smaller amounts of pyrrolic and quaternary nitrogen, changing the surface chemistry and influencing electrochemistry.

#### 3.3.2. Acid and base titrations

Activated carbons have amphoteric characteristics due to various types of functional groups. Nitrogen and oxygen functional groups exist in carbon structures, and the π-electron system of the carbon basal planes has basic characteristics by binding protons from an aqueous solution.^30^ A comparison of potentiometric titration methods with the Boehm titration methods has shown good agreement, provided the proper common metric is used.^65^ HCl neutralizes basic surface groups and the carbon basal plane π electron systems.^66^ It was proposed that the basic properties of carbon surfaces are due to a combination of redox reactions and proton transfer to/from the surface. In this study, titration with sodium ethoxide was not used to determine reactive carbonyls.^30^ Therefore, some oxygen groups accessible in aqueous solutions were not quantified.

The titration results in Table 6 showed similar trends to those observed previously for coconut-derived carbons, HNO_3_ oxidized carbon, and heat treatment studies of HNO_3_ oxidized carbons under a nitrogen atmosphere up to 800 °C.^33,34^ The inorganic contents of Series 1 and 2 carbons are low (1.5–3.9 wt%), and the functionalized carbons were much lower (∼0.5 wt%). Therefore, the inorganic material only makes a minimal contribution to the basic characteristics of the carbons. The acid and base titration results for carbons CO_2_/800-1 and CO_2_/800-1-HNO_3_ showed that HNO_3_ oxidation incorporated carboxylic, lactone/lactol, and phenolic groups into the carbon structure, which was consistent with both the O 1s and C 1s XPS data. In addition, a small amount of nitrogen (0.8 at%) was also incorporated into the carbon CO_2_/800-1-HNO_3_ structure. The XPS spectrum shows that *N*-oxide and pyrrolic functionalities are the primary forms of nitrogen in oxidized carbon CO_2_/800-1-HNO_3_. Titration results showed that carbons CO_2_/800-1, N_2_/800-1, and N_2_/1000-1 were basic carbons. CO_2_/700-1 contained small quantities of phenolic, lactone/lactol groups, carboxylic groups, and some basic groups. The titration results of CO_2_/800-1-K_2_CO_3_/800 showed that K_2_CO_3_ treatment of carbon CO_2_/800-1 at 800 °C also introduced a range of carboxyl, phenolic, and lactone/lactol functional groups and increased the acidity compared to CO_2_/800-1. These results are consistent with the XPS and TPD results discussed later. Titration studies show that N_2_/800-1-NH_3_/800 shows some weakly acidic character and basic character, whereas carbon N_2_/800-1 only has basic character. However, the surface oxygen was decreased by NH_3_ treatment at 800 °C to 1.8 at% (Table 3).

**Table 6:** Functional group concentrations from titration studies for carbons (mequiv. g^−1^)

Carbon sample	Phenolic	Lactone/lactol	Carboxylic	Basic
CO_2_/700-1	0.210	0.065	0.036	0.427
CO_2_/800-1	0.029	0	0	0.618
CO_2_/1000-1	0	0	0	0.453
N_2_/600-1	0.024	0	0	0.317
N_2_/800-1	0	0	0	0.506
N_2_/1000-1	0	0	0	0.228
CO_2_/800-1-HNO_3_	0.062	0.348	0.912	0.037
CO_2_/800-1-HNO_3_/400	0.915	0.017	0.206	0
CO_2_/800-1-HNO_3_/800	0.494	0	0	0
CO_2_/800-1-K_2_CO_3_/800	0.296	0.048	0.166	0.253
N_2_/800-1-NH_3_/800	0.287	0	0	0.541

#### 3.3.3. Temperature programmed desorption (TPD)

Temperature-programmed desorption provides information on the thermal stability of carbon functional groups. Surface oxygen species exist in various heterogeneous environments in carbons and decompose to form H_2_O, CO, and CO_2_ over a wide temperature range (150–1250 °C). CO_2_ is evolved from the decomposition of carboxyl groups, lactones, and reactions of surface oxygen species. A good correlation was observed between NaOH titration measurements and CO_2_ formation from thermal desorption studies.^67^ CO desorption results from the decomposition of carbonyl, quinones, hydroquinones, phenols, and ether functional groups. H_2_O results from the decomposition of hydroquinones and phenolic groups. Carboxylic acid groups usually decompose below 400 °C. Acid anhydrides, carbonyls, lactones, and lactones (carboxylic group derivatives) decompose between 600–700 °C, and hydroquinones/semiquinones and ethers decompose up to 1000 °C.^22,68^ H_2_O desorption results from the decomposition of carboxylic and phenolic groups and surface reactions of hydrogen species with oxygen surface groups. H_2_, N_2_, and NO are also desorbed at high temperatures and result from the decomposition of surface groups and the reaction of mobile surface species. This characterization method provides insight into the surface functional groups present in carbon samples by comparing gas evolution profiles for various species in TPD.

The TPD profiles for CO, CO_2_, and H_2_O for carbons in He (Series 1) and CO_2_ (Series 2) carbonization atmospheres are shown in Fig. S29 (ESI†). The low-temperature regions (<150 °C) of the TPD profiles show the desorption of H_2_O and N_2_ physisorbed in the porous structures with peaks at ∼80 °C. Physisorbed O_2_ was also desorbed, but this is not shown. The higher temperature peaks are due to the decomposition of functional groups. The TPD profiles for Series 1 of H_2_O, CO, and CO_2_ carbon decomposition products shift to higher temperatures with increasing HTT (Fig. S29a, ESI†). Carbon N_2_/600-1 has a significant weight loss of 4.35 wt% on heat treatment from 600 to 800 °C but much lower weight losses of 1.57 wt% between 800 and 1000 °C and 0.72 wt% between 1000 and 1200 °C (Fig. S30, ESI†). The TPD profiles of H_2_O, CO, and CO_2_ decomposition products for Series 2 carbons also shift to higher temperatures with increasing carbon HTT (Fig. S29b, ESI†). Carbon CO_2_/700-1 loses 2.32 wt% on heating from 700 to 800 °C in TPD but smaller weight loss values of 1.95 and 0.69 wt% over the temperature ranges 800–1000 °C and 1000–1200 °C, respectively. The weight loss during the carbonization process occurs above the corresponding HTT for the carbon (Fig. S29, ESI†), leading to changes in the bulk carbon structure.

HNO_3_ treatment of carbon CO_2_/800-1 incorporated additional oxygen and nitrogen functionality into the carbon structure to form oxidized carbon CO_2_/800-1-HNO_3_. The TPD CO_2_ profile of carbon CO_2_/800-1-HNO_3_ had a wide temperature range of (150–900 °C) with broad and overlapping desorption peaks (Fig. 4). The low temperature CO_2_ TPD peak at ∼239 °C overlapped with peaks at ∼231 °C for *m*/*z* 18 (H_2_O), at ∼207 °C for *m*/*z* 30 (NO) and ∼221 °C for *m*/*z* 28 (CO/N_2_). The desorption of H_2_O and NO are consistent with the decomposition of carboxylic acid and *N*-oxide surface groups, respectively. The low-temperature decomposition of *N*-oxides was reported previously by Xiao *et al*.^35^ The XPS results show that *N*-oxide functional groups (Fig. 1g and Table 5) were decomposed by heat treatment to 400 °C, but pyrrolic groups were still present (carbon CO_2_/800-1-HNO_3_/400, Fig. S27b, ESI†). Further heat treatment to 800 °C showed that all nitrogen surface groups were absent (carbon CO_2_/800-1-HNO_3_/800, Fig. S27c, ESI†). The other broad overlapping CO_2_ peaks are due to the desorption of carboxylic anhydride, lactone, and carbonyl surface sites, but H_2_O desorption is minimal over the temperature range 450–780 °C with a multicomponent peak in the temperature range (780–1250 °C), which is assigned to the decomposition of phenolic groups and surface reactions. The *m*/*z* 28 (CO) desorption peaks were observed at 675 and 878 °C, which are attributed to anhydride, lactones, *etc.* Heat treatment of oxidized carbon CO_2_/800-1-HNO_3_ to 800 °C removed oxygen functional groups, which decompose below 800 °C, forming carbon CO_2_/800-1-HNO_3_/800 (see XPS data in Table 4). The TPD profile of carbon CO_2_/800-1-HNO_3_/800 was comparable to carbon CO_2_/800-1, thereby showing the effect of HTT (Fig. 4). Previous thermolysis studies of HNO_3_ oxidized carbons showed that the order of thermal stability was:carboxylic acid < lactone/lactol < phenolic, carbonyl, semiquinone < chromene/pyronealthough decomposition temperature ranges may overlap.^33–35,69^

Comparison of the TPD profiles of carbons CO_2_/800-1-K_2_CO_3_/800 and CO_2_/800-1 shows that the reaction has modified the profiles (Fig. 5). Carbon CO_2_/800-1-K_2_CO_3_/800 showed a shift of the *m*/*z* 18 water peak to a higher temperature, and the *m*/*z* 28 CO peak was negligible. The K_2_CO_3_ treatment procedure produced strong bimodal CO_2_ desorption peaks at 725 and 875 °C. The low-temperature CO_2_ peak was attributed to lactones/carboxyl, while the high-temperature CO_2_ peak, which coincides with the start of H_2_O evolution, was assigned to the decomposition of phenolic and lactol groups and the reaction of surface species. The O 1s XPS results indicate carbon CO_2_/800-1-K_2_CO_3_/800 has lower C=O contents than CO_2_/800-1 (Table 4 and Table S5, ESI†). The titration results for carbons CO_2_/800-1-K_2_CO_3_/800 show increases in acidic carbon–oxygen surface groups compared with carbon CO_2_/800-1 with surface groups in the order: phenolic > carboxyl > lactone/lactol. The TPD results are consistent with the XPS (Table 4) and titration results (Table 6).

**Fig. 6:**
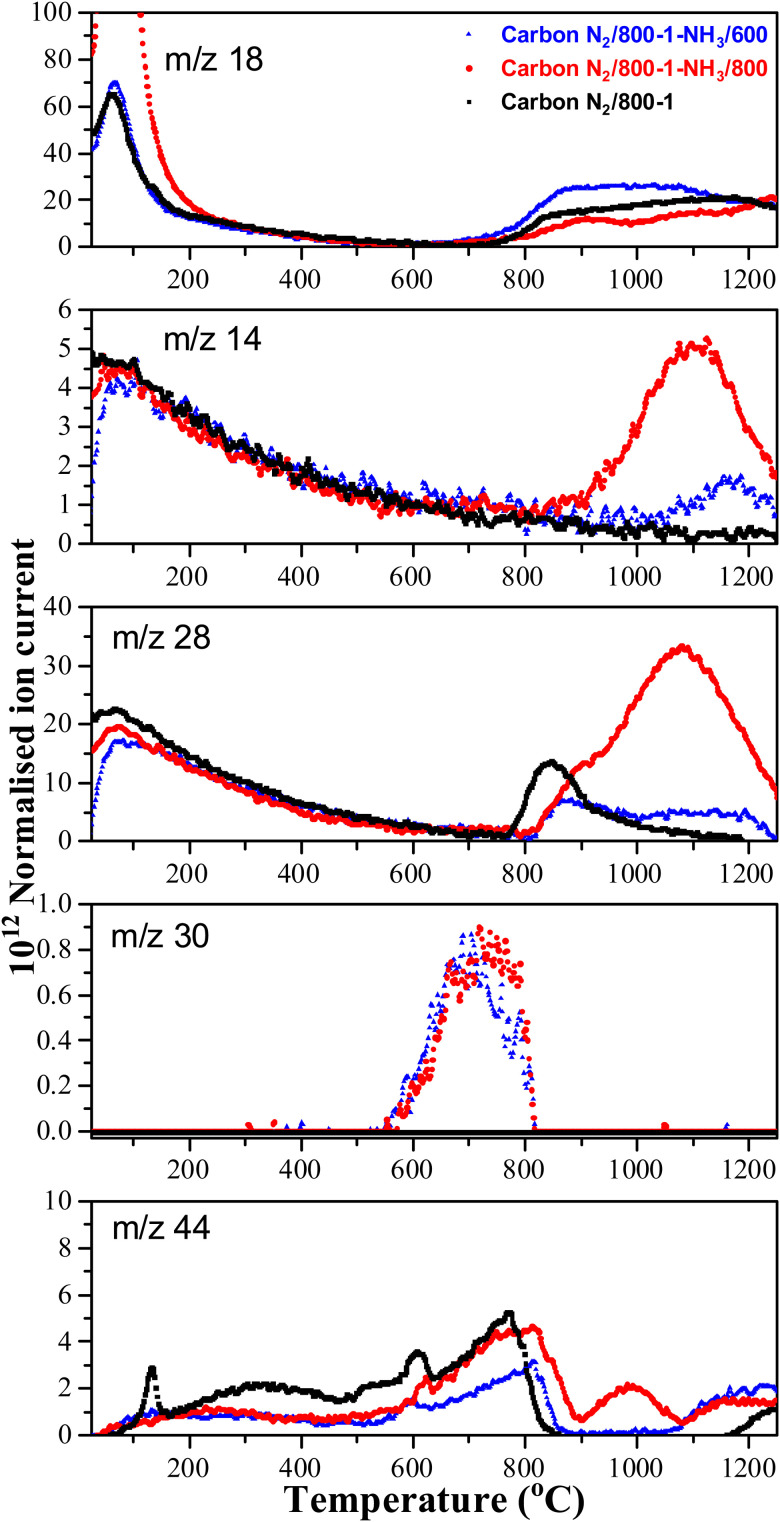
Comparison TPD profiles of carbon N_2_/800-1 and NH_3_ treated carbons N_2_/800-1-NH_3_/600 and N_2_/800-1-NH_3_/800 in He (35 mL min^−1^) at 10 °C min^−1^.

High-temperature ammonia treatment of carbons led to the incorporation of nitrogen functional groups into the carbon structure. The TPD *m*/*z* 28 (CO) peak profile for the starting carbon material N_2_/800-1 did not have a corresponding peak for *m*/*z* 14 (Fig. 6). Chemical analysis and the XPS spectrum of carbon N_2_/800-1 show minimal bulk and surface nitrogen contents. Therefore, the comparison of *m*/*z* 14 and *m*/*z* 28 TPD profiles allows the desorption of N_2_ to be distinguished from CO for carbons N_2_/800-1-NH_3_/600 and N_2_/800-1-NH_3_/800. The TPD profiles of both these samples had weak *m*/*z* 28 desorption peaks at ∼850–900 °C. The *m*/*z* 14 and 28 gas evolution profiles show N_2_ desorption and some CO desorption from 850–1200 °C (Fig. 6). N_2_ is the major nitrogen-containing desorption product. Nitrogen surface functional groups in carbon are mobile, reacting on the surface to form N_2_.^35^ Both NH_3_ treated carbons N_2_/800-1-NH_3_/600 and N_2_/800-1-NH_3_/800 showed small TPD peaks for *m*/*z* 30 (NO) at ∼740 °C (Fig. 6). The thermolysis of nitrogen functional groups shows that pyridine *N*-oxide is the least stable. Pyrrolic-N gradually transforms into pyridinic-N and subsequently into quaternary-N with increasing HTT.^35,63^ These nitrogen-containing desorption products are consistent with the nitrogen species detected in XPS (Fig. 1g and 3, Fig. S27 and Table 5, Table S5, ESI†) discussed previously.

#### 3.3.4. Fourier transform infrared spectroscopy

Surface functional groups have been identified using ATR infrared spectroscopy, but the spectra of carbons with HTTs of 800 °C and above are very weak, with broad peaks.^70–72^ In this study, the 900–1550 cm^−1^ spectral region provides a spectral fingerprint region, and the 1550–1800 cm^−1^ shows absorption bands due to aromatic C=C and C=O stretching vibrations. The ATR spectra for Series 1 carbons N_2_/600-1 and N_2_/800-1 show that the intensity of the band at 1579 cm^−1^ decreases markedly at 800 °C (Fig. S41a, ESI†). However, the infrared spectrum of carbon CO_2_/800-1 in Series 2 has a more substantial band at 1580 cm^−1^ than carbon N_2_/800-1, indicating that carbonization in a CO_2_ atmosphere has incorporated some surface oxygen groups into the carbon structure (see Fig. S41b, ESI†). Comparison of the ATR spectra of carbons CO_2_/800-1 and CO_2_/800-1-K_2_CO_3_/800 shows that the spectra are very similar, but the latter have higher intensity (see Fig. S41c, ESI†). The main ATR peaks at 1580 and 1713 cm^−1^ for CO_2_/800-1-HNO_3_, as shown in Fig. S41d (ESI†), are similar to the diffuse reflectance spectrum reported for nitric acid oxidation of a steam-activated carbon, which had bands at 1576 and 1717 cm^−1^.^35^ The analytical data for carbon CO_2_/800-1-HNO_3_ is similar to that reported for nitric acid oxidation of a steam-activated carbon.^35^ The peak at 1713 cm^−1^ is consistent with the presence of carboxylic groups. Heat treatment studies for HNO_3_ oxidation of the steam-activated carbon resulted in progressive decomposition of the surface oxygen groups, with lactone and lactol groups forming at 400–600 °C. The spectra for the carbon treated at high temperatures with ammonia are shown in Fig. S41e (ESI†). There are relatively small differences between the infrared spectra of carbons N_2_/800-1 and N_2_/800-1-NH_3_/600. However, the infrared spectrum of carbon N_2_/800-1-NH_3_/800 is markedly different with the loss of the 1007 cm^−1^ peak and increased intensity for the 1190 and 1577 cm^−1^ peaks.

### 3.4. Carbonaceous molecular structure

#### 3.4.1. Raman spectroscopy

Raman spectra of disordered carbons have two prominent peaks: the D peak, which is attributed to the A_1g_ breathing mode in disordered carbon-ring clusters (near the K-zone boundary) with more than five aromatic rings, and the G peak is due to the E_2g_ breathing mode of carbon aromatic sites.^73,74^ Various curve fitting methods using up to 9 peaks have been used to analyze the Raman spectra of carbons. The Raman spectra of carbons used in this study showed a shoulder on the D peak at 1180 cm^−1^, designated as the S peak. The S peak has been assigned to the symmetric breathing mode of small polyaromatics and rings containing seven or more carbons.^75^ A small peak at ∼1510 cm^−1^ (A peak) was also required for curve fitting, and this has been attributed to amorphous carbon linked to oxygen functional groups.^31,75,76^ The Raman spectra were curve-fitted using D, G, A, and S peaks using the method described previously.^75^ The A and S Raman peaks were fixed at 1510 and 1180 cm^−1^ in the curve fitting of all spectra. Typical examples of the peak fitting of the Raman spectra, the peak components, and residuals for the fitting and the *χ*^2^ values are shown in Fig. S33–S39 (ESI†), and the data are given in Table 7. Repeatability studies are shown in Table S7 and Fig. S32 (ESI†). It is evident from the residuals for the peak curve fitting procedure that the four peaks provide good fits to all the Raman spectra studied. The contributions of the S and A peaks to the Raman spectra were 9.5–14.5 and 12–19% of the spectra, respectively. The contribution of the A peak increases significantly over the carbonization temperature of 600–700 °C but has a constant contribution to the overall spectral profile thereafter.

**Table 7:** Raman spectroscopy parameters *I*_D_/*I*_G_ (peak intensity ratio for D and G peaks), *A*_D_/*A*_G_ (area ratio for D and G peaks), full bandwidths at half maximum of D and G peaks, and D and G peak Raman shifts of carbons from curve fitting

Carbon sample	*I*_D_/*I*_G_	*A*_D_/*A*_G_	FWHM_D_ (cm^−1^)	FWHM_G_ (cm^−1^)	D-peak (cm^−1^)	G-peak (cm^−1^)	Reduced *χ*^2^	*R* ^2^ (COD)
Carbonization under N_2_ (Series 1)								
N_2_/600-0	0.65	1.22	182.9	66.3	1345.4	1594.7	374.6	0.992
N_2_/600-1	0.67	1.26	181.6	64.9	1344.6	1595.3	212.8	0.993
N_2_/600-3	0.70	1.31	178.2	64.7	1341.6	1596.7	168.1	0.992
N_2_/700-0^a^	0.81	1.47	164.2	61.7	1337.5	1597.5	61.3, 87.2	0.993, 0.990
N_2_/700-1^a^	0.85	1.54	162.8	61.1	1336.3	1598.2	54.5, 66.0	0.990, 0.992
N_2_/700-3^a^	0.92	1.57	160.9	63.6	1337.4	1596.9	52.9, 63.0	0.985, 0.992
N_2_/800-0^a^	0.99	1.71	153.1	60.1	1337.5	1598.6	46.8, 60.5	0.976, 0.991
N_2_/800-1^a^	0.98	1.67	157.2	62.8	1338.5	1598.3	28.7, 34.8	0.992, 0.977
N_2_/800-3^a^	1.02	1.65	154.6	64.5	1340.1	1600.0	37.5, 43.8	0.987–0.991
N_2_/1000-0	1.06	1.74	150.0	61.8	1338.1	1595.3	99.0	0.996
N_2_/1000-1	1.14	1.70	142.3	64.2	1341.0	1596.1	104.3	0.993
N_2_/1000-3	1.16	1.67	137.7	64.8	1342.2	1595.7	99.2	0.992
Carbonization under CO_2_ (Series 2)								
CO_2_/700-0	0.85	1.50	164.6	63.0	1336.2	1595.0	68.3	0.994
CO_2_/700-1^a^	0.90	1.54	161.3	63.8	1337.3	1597.0	41.3–50.4	0.991–0.992
CO_2_/700-3^a^	0.95	1.62	158.6	62.8	1336.5	1596.3	39.2–55.0	0.987–0.993
CO_2_/800-0	0.96	1.58	154.2	63.8	1337.7	1597.4	54.8	0.993
CO_2_/800-1^a^	1.00	1.68	153.4	62.0	1337.8	1597.4	35.2–40.7	0.985–0.992
CO_2_/800-3^a^	1.05	1.72	142.3	58.9	1336.9	1597.3	38.6, 53.4	0.985–0.990
CO_2_/1000-0	1.06	1.74	145.2	59.9	1336.8	1597.0	33.6	0.989
CO_2_/1000-1	1.11	1.73	144.0	63.0	1340.4	1596.0	117.1	0.994
Functionalized carbons								
CO_2_/800-1-HNO_3_	0.96	1.43	141.7	64.5	1344.2	1601.9	64.9	0.994
CO_2_/800-1-HNO_3_/400	0.94	1.47	150.7	65.5	1343.7	1601.0	45.5	0.993
CO_2_/800-1-HNO_3_/800	1.04	1.80	133.5	52.3	1343.1	1605.3	94.4	0.985
CO_2_/800-1-K_2_CO_3_/800	1.08	1.68	139.4	60.9	1341.4	1602.0	46.7	0.986
N_2_/800-1-NH_3_/600	1.02	1.92	153.6	55.3	1337.8	1598.3	138.3	0.996
N_2_/800-1-NH_3_/800	1.06	1.83	145.8	57.1	1339.2	1602.1	234.6	0.994

a Average values.

The *I*_D_/*I*_G_ ratio (peak intensity ratio of the D and G peaks) is used widely as a measure of the degree of ordered carbon in carbon materials, while the peak area ratio for the D and G peaks (*A*_D_/*A*_G_) are less commonly reported.^77,78^ The Raman spectra for carbons prepared in N_2_ (Series 1) and CO_2_ (Series 2) atmospheres show markedly increased *I*_D_/*I*_G_ and *A*_D_/*A*_G_ ratios with increasing HTT from 600 °C up to 800 °C and slower increase from 800 °C to 1000 °C (Fig. S40a and b, ESI†). Previous studies of the Raman spectra of carbons derived from a wide range of biomass materials have shown that *I*_D_/*I*_G_ increases for the temperature range 800 to ∼2000 °C but decreases at higher temperatures.^79^ In contrast, changes with carbonization hold time were small, indicating that temperature is the primary experimental carbonization parameter influencing carbon structure. The full width at half maximum of the D peak (FWHM_D_) decreased gradually with increasing HTT (Fig. S40c, ESI†), and this carbon structural rearrangement resulted in a more ordered carbon structure.^80^ The full width at half maximum intensity of the G peak (FWHM_G_), D, and G peak positions are included in Table 7, but none of these parameters show marked changes with carbonization hold time (0–3 h) or carbonization gaseous atmosphere. The changes in D and G peak Raman shifts with HTT were small. Therefore, significant conclusions regarding carbon structure could not be obtained from these parameters.^81^

The incorporation of functional groups produced minor changes in the Raman spectra. HNO_3_ oxidation reduced the FWHM_D_ of the D band, *I*_D_/*I*_G_, and *A*_D_/*A*_G_ ratio in the Raman spectra of carbon CO_2_/800-1-HNO_3_ compared with carbon CO_2_/800-1. Heat treatment of carbon CO_2_/800-1-HNO_3_ resulted in substantial weight loss (Fig. S30b, ESI†) due to the decomposition of the functional groups in TPD (Fig. 4). The *A*_D_/*A*_G_ ratio in the Raman spectrum of carbon CO_2_/800-1-HNO_3_/800 was very similar to that of the starting material carbon CO_2_/800-1. The values of D bandwidth (FWHM_D_) were slightly lower for the heat-treated carbon series CO_2_/800-1-HNO_3_, CO_2_/800-1-HNO_3_/400, and CO_2_/800-1-HNO_3_/800 than for the initial carbon CO_2_/800-1. The high-temperature treatment of carbon CO_2_/800-1 with K_2_CO_3_ at 800 °C and treatment of carbon N_2_/800-1 with NH_3_ at 800 °C reduced FWHM_D_ slightly compared to the corresponding precursor materials. The changes in the Raman D/G peak intensity ratios, peak area ratios, and D bandwidths show that carbonization temperature has a much more significant effect on the carbon structure than the chemical treatment and thermolysis to incorporate functional groups.

#### 3.4.2. Powder X-ray diffraction (PXRD)

The PXRD profiles of the carbon samples had two broad peaks at ∼22–23° and ∼43° representing (002) and (100) reflections of carbon crystallites (Fig. S31, ESI†). The PXRD profiles in Series 1 and 2 carbons had very small peaks at 2*θ* ∼ 29.5° due to inorganic mineral content (Fig. S31a and b, ESI†). This crystalline phase present in the biochar was not present in the ash.^82^ Crystallite thickness (*L*_c_), apparent crystallite diameter (*L*_a_), and interlayer distance (*d*_002_) are given in Table 8. Repeatability studies are shown in Table S6 (ESI†). An increase in HTT for both Series 1 (600–1000 °C) and Series 2 (700–1000 °C) carbons increased *L*_a_ significantly (see Fig. S40d, ESI†), indicating the presence of larger apparent crystallite sizes in the carbon structure, which is attributed to the dewrinkling of graphene layer sheets. Previous studies of 5 coal chars and a carbonized pitch with HTTs 500–2800 °C showed that *L*_a_ increased over the range 18–66 Å with *L*_a_ had values in the range 18–33 Å for 600–1000 °C, which is similar to the *L*_a_ range for the carbons used in this study (Fig. S40e and f, ESI†). The Raman D bandwidth (FWHM_D_) for these chars decreased with increasing HTT (500–1900 °C), and this correlated with an increase in the apparent crystallite diameter (*L*_a_).^80^ This correlation was also observed for the carbons used in this study (Fig. S40e and f, ESI†). It has been reported that CO_2_ gasification removed more aliphatic carbon groups, leading to higher *L*_a_ values and dewrinkling of carbon graphene sheets.^83^ However, the *L*_a_ data for carbons CO_2_/800-0, CO_2_/800-1, and CO_2_/800-3 were not statistically different (Table 8). The *L*_a_ data for carbons N_2_/800-0, N_2_/800-1, and N_2_/800-3 increased from 20.09 Å for carbon N_2_/800-1 to 23.57 Å for carbon N_2_/800-3. No clear trends in stack layer height (*L*_c_) and interlayer spacing (*d*_002_) of the graphene basal planes with HTT were observed for both Series 1 and 2 carbons.

**Table 8:** Crystallite thickness *L*_c_, interlayer distance *d*_002_, and apparent crystallite diameter *L*_a_ from PXRD

Carbon sample	Crystallite thickness, *L*_c_ (Å)	Interlayer distance, *d*_002_, (Å)	Crystallite diameter, *L*_a_ (Å)
Carbonization in N_2_ (Series 1)			
N_2_/600-1	10.75	4.14	18.33
N_2_/600-3	10.67	3.99	18.61
N_2_/700-0	9.83	3.83	19.29
N_2_/700-1	10.19	4.01	21.10
N_2_/700-3	9.98	3.85	21.00
N_2_/800-0	10.02	3.83	20.09
N_2_/800-1	10.21	3.86	21.03
N_2_/800-3^a^	9.98	3.83	23.57
N_2_/1000-1	9.99	3.86	26.85
N_2_/1000-3	10.12	3.84	28.6
Carbonization in CO_2_ (Series 2)			
CO_2_/700-1	9.54	3.90	19.98
CO_2_/800-0	9.79	3.86	23.52
CO_2_/800-1^a^	10.14	3.86	23.53
CO_2_/800-3^a^	9.32	3.86	24.31
CO_2_/1000-1	10.23	4.04	28.71
Functionalized carbons			
CO_2_/800-1-HNO_3_	10.07	3.92	25.65
CO_2_/800-1-HNO_3_/400	10.32	3.66	25.98
CO_2_/800-1-HNO_3_/800	10.81	3.80	24.01
CO_2_/800-1-K_2_CO_3_/800	8.55	4.12	27.87
N_2_/800-1-NH_3_/600	10.07	4.18	23.00
N_2_/800-1-NH_3_/800	10.24	3.76	26.99

a Average values.

Carbons with the same HTT (CO_2_/800-1 and N_2_/800-1) were used for the carbon functionalization studies with maximum treatment temperatures of 800 °C to eliminate structural changes due to HTT. Carbon molecular structure changes during functionalization studies were investigated by comparison of X-ray diffraction parameters *d*_002_, *L*_c_, and *L*_a_ (see Table 8). The nitric acid oxidation of carbon CO_2_/800-1 produced carbon CO_2_/800-1-HNO_3_, and *L*_c_, *d*_002_, and *L*_a_ were increased, but the values of *L*_c_ and *d*_002_ were close to three standard deviations. HNO_3_ oxidation of carbon CO_2_/800-1 increased the crystallite size (*L*_a_) from 23.53 Å for carbon CO_2_/800-1 to 25.65 Å in carbon CO_2_/800-1-HNO_3_. This is consistent with the dewrinkling of the graphene layers. Heat treatment studies of carbon CO_2_/800-HNO_3_ to form CO_2_/800-1-HNO_3_/800 led to a reduction in *L*_a_ to 24.01 Å, similar to the starting carbon material.

Treatment of carbon CO_2_/800-1 with K_2_CO_3_ at 800 °C decreased *L*_c_ and increased *d*_002_ and *L*_a_ (see Table 8). The apparent crystallite size (*L*_a_) increased from 23.53 Å for carbon CO_2_/800-1 to 27.87 Å for carbon CO_2_/800-1-K_2_CO_3_/800. The increase in the apparent crystallite size results from the dewrinkling of the graphene layers. Treatment of carbon N_2_/800-1 with NH_3_ at 800 °C to form carbon N_2_/800-1-NH_3_/800 did not change *L*_c_, *d*_002_ was decreased, and *L*_a_ increased. The apparent crystallite size increased from 21.03 Å for carbon N_2_/800-1 to 26.99 Å for carbon N_2_/800-1-NH_3_/800. This is also consistent with the dewrinkling of the graphene layers. Therefore, all three treatment procedures increased apparent crystallite size (*L*_a_). Heat treatment of carbon CO_2_/800-1-HNO_3_ increased *L*_c_ and decreased *L*_a_, while there was no clear trend in *d*_002_.

Many carbons, including various graphite, glassy carbons, fibers, carbon black, and other carbons, have been used as electrode materials.^7^ The carbons used in this study were non-graphitizable biochars and have the following PXRD parameter ranges: crystal thickness (*L*_c_) 8.55–10.75 Å, apparent crystallite diameter (*L*_a_) 18.33–28.71 Å and interlayer spacing (*d*_002_) 3.66–4.14 Å. The ranges of values for *L*_a_ and *L*_c_ for the biomass carbons are similar to glassy carbon and Spheron 6 carbon black used previously, but the *d*_002_ values are significantly higher for the carbons used in this study than the maximum (3.55 Å) reported previously.^7^ Carbons with HTTs ≤ 1000 °C consist of units of no more than 10–12 aromatic rings with stacks of 2–3 units. The units have no general organization except for a possible local organization. This is consistent with a more significant structural disorder in the biomass carbons.

### 3.5. Electrochemical characteristics

#### 3.5.1. Carbon structure

**Fig. 7:**
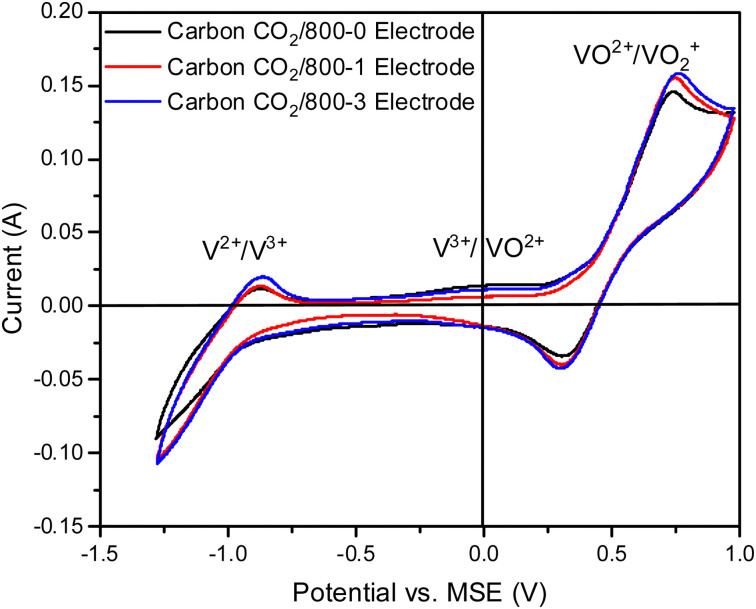
Comparison of cyclic voltammograms of electrodes prepared from carbons with HTT 800 °C and various hold times (carbons CO_2_/800-0, CO_2_/800-1, and CO_2_/1000-3) for vanadium redox reactions, Sweep rate 20 mV s^−1^.

The cell electrodes used for cyclic voltammetry and electrical impedance spectroscopy of vanadium redox reactions were identical carbon/PVDF polymer composites. The reactions occur on the carbon but might be affected by the porous structure of the composite electrode. The primary factors determining carbon structure are the biomass carbon precursor and the experimental carbonization conditions, mainly heat treatment temperature (HTT), with secondary experimental factors such as gaseous atmosphere, hold time, and heating rate. The carbonization heating rate was held constant throughout this study. The effect of hold time on carbon structural characteristics was examined for carbonization at 800 °C under a carbon dioxide atmosphere, and the cyclic voltammograms (CVs) are shown in Fig. 7. The differences between the cyclic voltammograms are minimal, and this is consistent with the similarities in the carbon structural characterization data for the porous structure characteristics from gas adsorption, surface analysis from XPS, and molecular structure from Raman and XRD (see Tables 2–5, 7, and 8). Therefore, the carbon hold time at the HTT was held constant at 1 h for all the carbons used in the detailed electrochemistry studies.

Three series of carbon samples were used in this study to explore the role of carbonization conditions and chemical treatment procedures on carbon in carbon/PVDF composite electrode properties. Cyclic voltammetry was studied at three sweep rates: 5, 10, and 20 mV s^−1^. Consistent trends were obtained for all three sweep rates for carbons prepared under nitrogen and carbon dioxide atmospheres, with the highest currents obtained for 20 mV s^−1^ (Fig. S18, ESI†). Therefore, the 20 mV sweep rate was used for cyclic voltammetry.

**Fig. 8:**
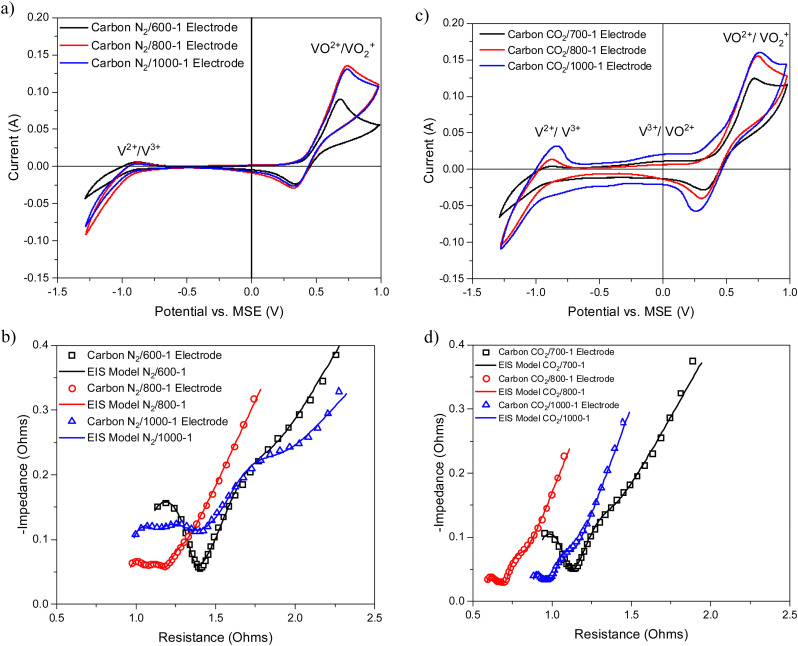
Comparison of electrochemical results for electrodes prepared from carbons prepared under nitrogen and carbon dioxide atmospheres (a) cyclic voltammograms of N_2_/600-1, N_2_/800-1, N_2_/1000-1 (b) Nyquist plots of N_2_/600-1, N_2_/800-1, N_2_/1000-1 and corresponding models; (c) cyclic voltammograms of CO_2_/700-1, CO_2_/800-1 and CO_2_/1000-1; and (d) Nyquist plots of CO_2_/700-1, CO_2_/800-1 and CO_2_/1000-1 and corresponding models.

The cyclic voltammograms with a sweep rate of 20 mV s^−1^ for electrodes made from carbons with HTTs 600, 800, and 1000 °C in a nitrogen atmosphere (Series 1) are shown in Fig. 8a. The CVs have a very weak V^2+^ → V^3+^ peak, a V^3+^/VO^2+^ was absent, and the strongest peak was the VO^2+^ → VO_2_^+^ peak. An increase in carbon HTT enhances the VO^2+^ → VO_2_^+^ peak at an HTT of 800 °C for Series 1 (N_2_ atmosphere) carbons, but an increase of HTT to 1000 °C appears to have, at most, a small effect. Similar trends were observed at CV sweep rates of 5 and 10 mV s^−1^ (Fig. S16, ESI†). A weak V^2+^ → V^3+^ peak was observed, but no V^3+^ → VO^2+^ peak was observed. Fig. 8b shows the corresponding EIS plots, showing a very distinct difference between carbons with HTTs of 600 and 800 °C. The modeling studies for the EIS are shown in Table S1 (ESI†). Overall, the electrodes with carbon HTT 800 °C were shifted to lower electrode resistance than either carbon 600 °C or carbon 1000 °C electrodes.

The CVs for electrodes made from carbons in Series 2 by carbonization at 700, 800, and 1000 °C in a carbon dioxide atmosphere are shown in Fig. 8c. The CVs for these carbon electrodes are slightly enhanced for the same temperatures compared to the carbon samples prepared in a nitrogen atmosphere (see Fig. 8a). An increase in HTT results in enhancement of the V^2+^ → V^3+^ peak in the CVs and the V^3+^/VO_2_^+^ couple is observed as an intermediate weak broad peak in carbon CO_2_/1000-1. There are only very small differences for VO^2+^ → VO_2_^+^ peak for HTTs of 800 and 1000 °C. The CVs for carbonization in N_2_ and CO_2_ at 800 and 1000 °C are shown in Fig. S18 (ESI†). The oxidation of V^3+^ to VO^2+^ was observed as a small peak at ∼0.1 V (*vs.* MSE) for carbons prepared under a CO_2_ atmosphere (Fig. 8c). This phenomenon has been reported previously.^13,14,84^ The corresponding Nyquist plots are shown in Fig. 8d. Comparison of the Nyquist plots for electrodes made from carbons prepared under N_2_ (Series 1) and CO_2_ (Series 2) at 800 and 1000 °C shows that the carbons with HTT 1000 °C are shifted to higher resistance (see Fig. S15b and d, ESI†). The Nyquist graphs show the same trend, with plots shifted to the lowest resistance for HTT 800 °C for carbonization in N_2_ and CO_2_ atmospheres. The electrical resistivity properties of high-density polyethylene/carbon black composite materials depend on carbon structure and surface functional groups. The resistivity properties of the composites can be modified using gasification and liquid phase chemical treatment of the carbon black.^85,86^ It is evident that the carbonization temperature for the carbon component of the carbon/PVDF electrodes has a major effect on cyclic voltammograms and Nyquist plots for vanadium redox reactions.

A weak shoulder on the low-frequency side of the Nyquist graph was observed at ∼15 Hz for the CO_2_ carbonization Series 2 and lower (∼2 Hz) for the corresponding N_2_ carbonization Series 1. The peak was very weak in carbon N_2_/800-1. The frequency shift for the N_2_ Series 1 compared with the CO_2_ Series 2 carbons is possibly due to differences in diffusion for these electrodes. The EIS of carbon N_2_/600-1 has a well-defined high-frequency peak, indicating a single time constant. However, electrodes prepared from carbons N_2_/800-1 and N_2_/1000-1 have two weak peaks (∼750 and 10 000 Hz) indicative of the presence of two time constants. The carbons in Series 2 prepared under the CO_2_ atmosphere show a similar trend with HTT for electrodes prepared from carbons CO_2_/800-1 and CO_2_/1000-1 with very weak peaks in the high-frequency region.

The effect of HTT on CV and EIS is similar for carbonization in both N_2_ (Series 1) and CO_2_ (Series 2). Gas adsorption studies showed that both series of carbons were predominantly ultramicroporous (<0.7 nm), and therefore, there are liquid phase diffusion limitations into the porous structures (Section 3.2). Hence, changes in CV and EIS with HTT are not directly related to the carbon pore structure. However, porosity may be formed in the carbon/PVDF composite electrode, which may be influenced by differences in carbon surface chemistry. XPS shows that the surface oxygen functional groups are very similar for both carbon series for HTTs in the range of 700–1000 °C. However, minor differences were observed for Series 1 (N_2_ atmosphere) with HTTs 600 °C (Section 3.3.1.). Titration studies show that carbon CO_2_/700-1 has amphoteric properties, while at an HTT of 800 °C, there were virtually only basic characteristics (Table 6). The Raman spectra and PXRD data show that the amorphous carbon molecular structures change with increasing HTT (see Tables 7 and 8). The change in PXRD shows that crystallite size *L*_a_ increases with increasing HTT (Section 3.4.2.) The changes in Raman parameters (*I*_D_/*I*_G_, *A*_D_/*A*_G_, and FWHM_D_) change markedly over the HTT range 600–800 °C (Section 3.4.1), but only to a smaller extent up to 1000 °C. The cyclic voltammograms change markedly in the HTT range of 600–800 °C but only slightly over the temperature range (800–1000 °C). This transition temperature coincides with the minimum carbon electrode resistance measured by EIS and the change in carbon surface characteristics from acidic to basic. Therefore, the change in CV and EIS for carbons in Series 1 and 2 are attributed to changes in carbon molecular structure, giving rise to changes in electrode resistance.

#### 3.5.2. Carbon functionalization

**Fig. 9:**
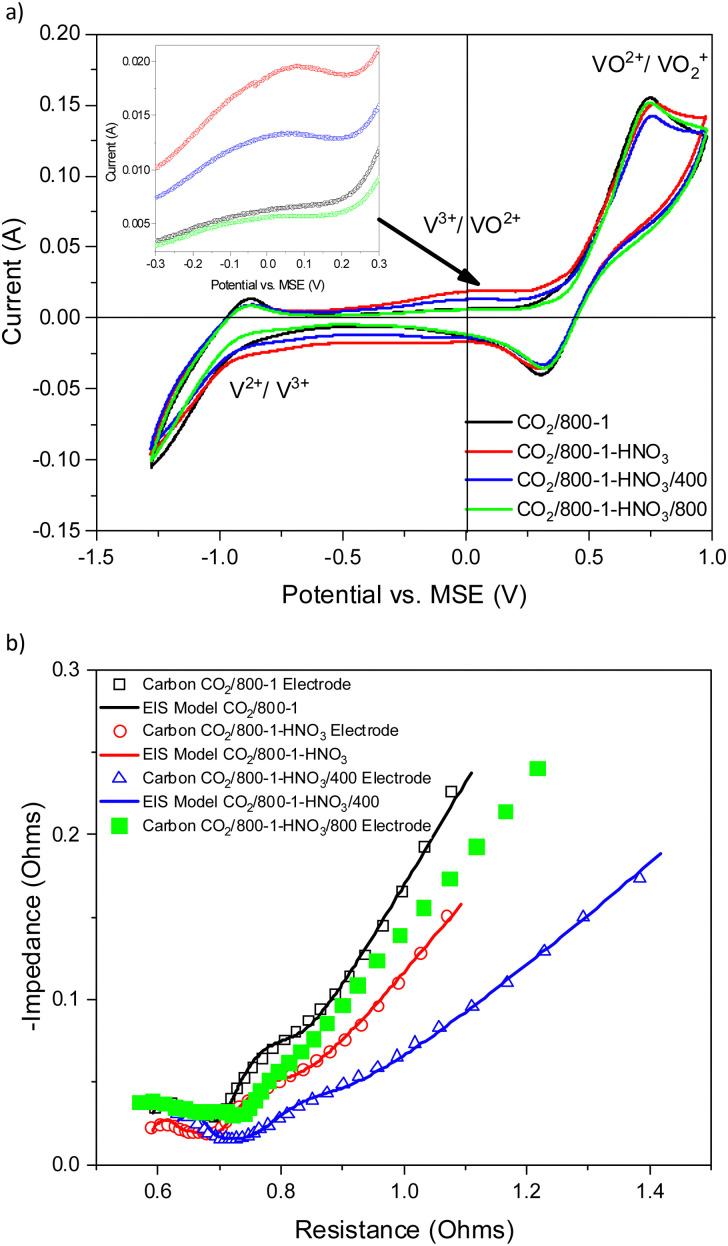
Comparison of electrochemical results for electrodes prepared from carbons CO_2_/800-1, CO_2_/800-1-HNO_3_, CO_2_/800-1-HNO_3_/400, and CO_2_/800-1-HNO_3_/800 prepared by oxidation in nitric acid and heat treatment (a) cyclic voltammograms with inset figure for potential range −0.3 to 0.3 V, and (b) Nyquist plots and corresponding models.

A comparison of the cyclic voltammograms and Nyquist plots for electrodes prepared from carbons CO_2_/800-1, CO_2_/800-1-HNO_3_, CO_2_/800-1-HNO_3_/400, and CO_2_/800-1-HNO_3_/800 are shown in Fig. 9a and b, respectively. The CVs for the electrodes prepared from these four carbons are similar. However, a systematic weak trend was observed where the broad weak feature assigned to the V^3+^/VO^2+^ couple increased with HNO_3_ oxidation, and this was reversed by heat treatment. Significant differences were observed in the Nyquist plot for carbon CO_2_/800-1-HNO_3_, which shifted to higher resistance compared with carbon CO_2_/800-1. Heat treatment also reversed this change, with the Nyquist plots carbon CO_2_/800-1-HNO_3_/800 being similar to carbon CO_2_/800-1. The differences in EIS profiles for electrodes prepared from CO_2_/800-1, CO_2_/800-1-HNO_3_, and CO_2_/800-1-HNO_3_/400 (see Fig. 9) were smaller than for the HTT series (CO_2_/700-1, CO_2_/800-1 and CO_2_/1000-1, see Fig. 8d). A weak low frequency (15–20 Hz) peak was observed in the EIS of electrodes prepared from these carbons.

Carbon CO_2_/800-1-HNO_3_ has a range of surface functional groups, including carboxylic, anhydride, lactone/lactol, and phenolic groups. Heat treatment in H_2_ to form carbon CO_2_/800-1-HNO_3_/400 resulted in the removal of mainly carboxylic and *N*-oxide groups, as shown by XPS, TPD, and titration studies. Heat treatment in H_2_ to form carbon CO_2_/800-1-HNO_3_/800 progressively removes labile oxygen functional groups. The pore structures were predominantly ultramicroporous (Table 2), limiting liquid phase diffusion. Only minor differences in Raman and XRD characterization data for this series of carbons were observed for the PXRD and Raman spectra of this series of carbons (Tables 7 and 8). Therefore, the differences in electrochemistry could not be accounted for by significant changes in either porous or molecular structures. This series of carbons allows an investigation of the role of oxygen surface functional groups in the vanadium redox reactions.

Therefore, reversible changes in CV for the V^3+^ → VO^2+^ couple were established by combining HNO_3_ oxidation and heat treatment in hydrogen. These changes follow the trend with oxygen functional group concentrations since the ultramicroporous structures limit diffusion, and changes in molecular structures are minimal. The progressive reversible changes in surface oxygen functional groups explain the differences in the CVs and Nyquist plots.

**Fig. 10:**
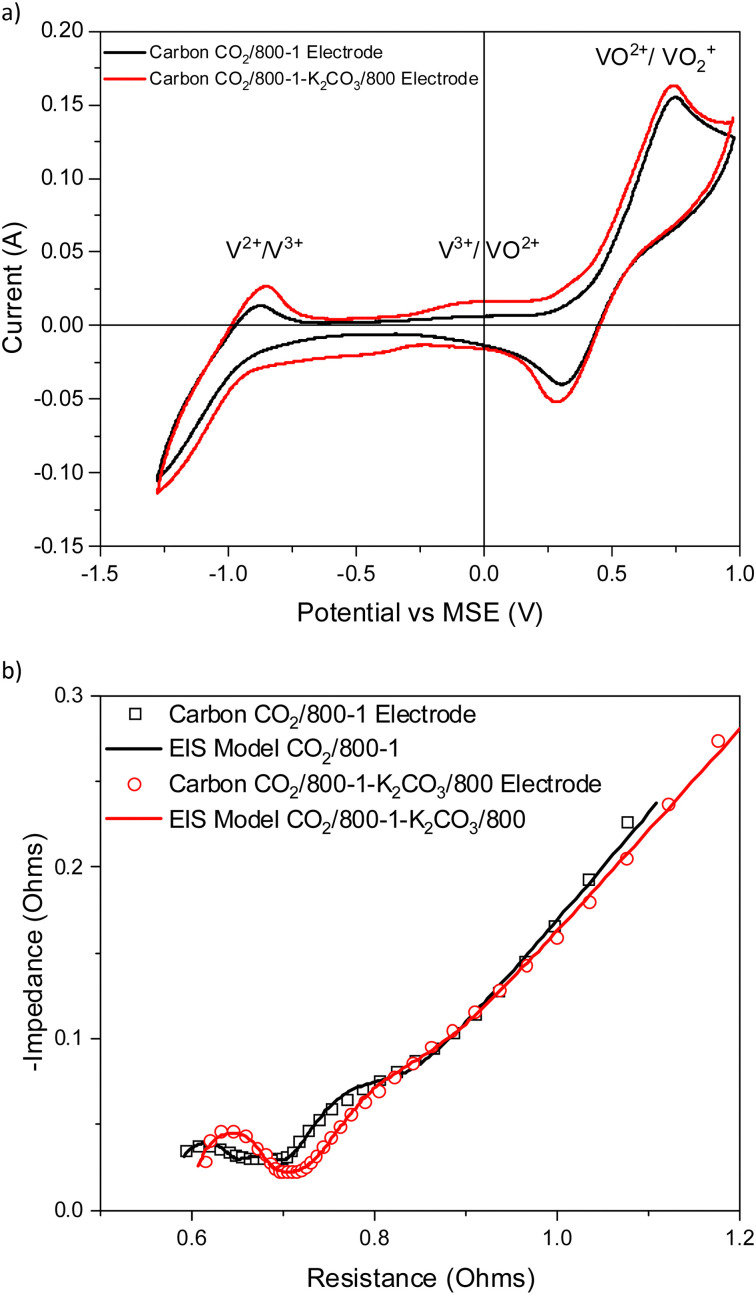
Comparison of electrochemical results for electrodes prepared from carbons CO_2_/800-1 and CO_2_/800-1-K_2_CO_3_/800 (a) cyclic voltammograms and (b) Nyquist plots  and corresponding models.

Carbon CO_2_/800-1 was treated with K_2_CO_3_ at 800 °C to form carbon CO_2_/800-1-K_2_CO_3_/800, and the cyclic voltammograms and Nyquist plots for the electrodes prepared for these carbons are shown in Fig. 10a and b. The modeling studies showed that the electrode resistances of carbons CO_2_/800-1 and CO_2_/800-1-K_2_CO_3_/800 were similar (Table S1, ESI†). The two weak high frequency Nyquist peaks at 750 and 12000 Hz observed for CO_2_/800-1 have been replaced by a single peak at 23000 Hz in CO_2_/800-1-K_2_CO_3_/800. The weak low frequency peak mentioned earlier at 15 Hz corresponds to a shoulder at ∼10 Hz in CO_2_/800-1-K_2_CO_3_/800. The increased surface homogeneity is a possible explanation of the change in the high frequency region of the Nyquist plots of CO_2_/800-1-K_2_CO_3_/800 compared with CO_2_/800-1. Carbon CO_2_/800-1-K_2_CO_3_/800 had a more pronounced CV peak for V^3+^/VO^2+^ than carbon CO_2_/800-1.

K_2_CO_3_ treatment at 800 °C only leads to small changes in the porous structure, and both samples are predominantly ultramicroporous (Table 2). The PXRD and Raman results for the carbon do not show significant differences (Tables 7 and 8). These results indicate minimal changes in the porous and molecular structures. The titration results showed that carbon CO_2_/800-1-K_2_CO_3_/800 has amphoteric surface characteristics containing both acidic (phenolic, lactone/lactol, and carboxyl) groups and basic groups (Table 6), whereas carbon CO_2_/800-1 had predominantly basic surface characteristics.

**Fig. 11:**
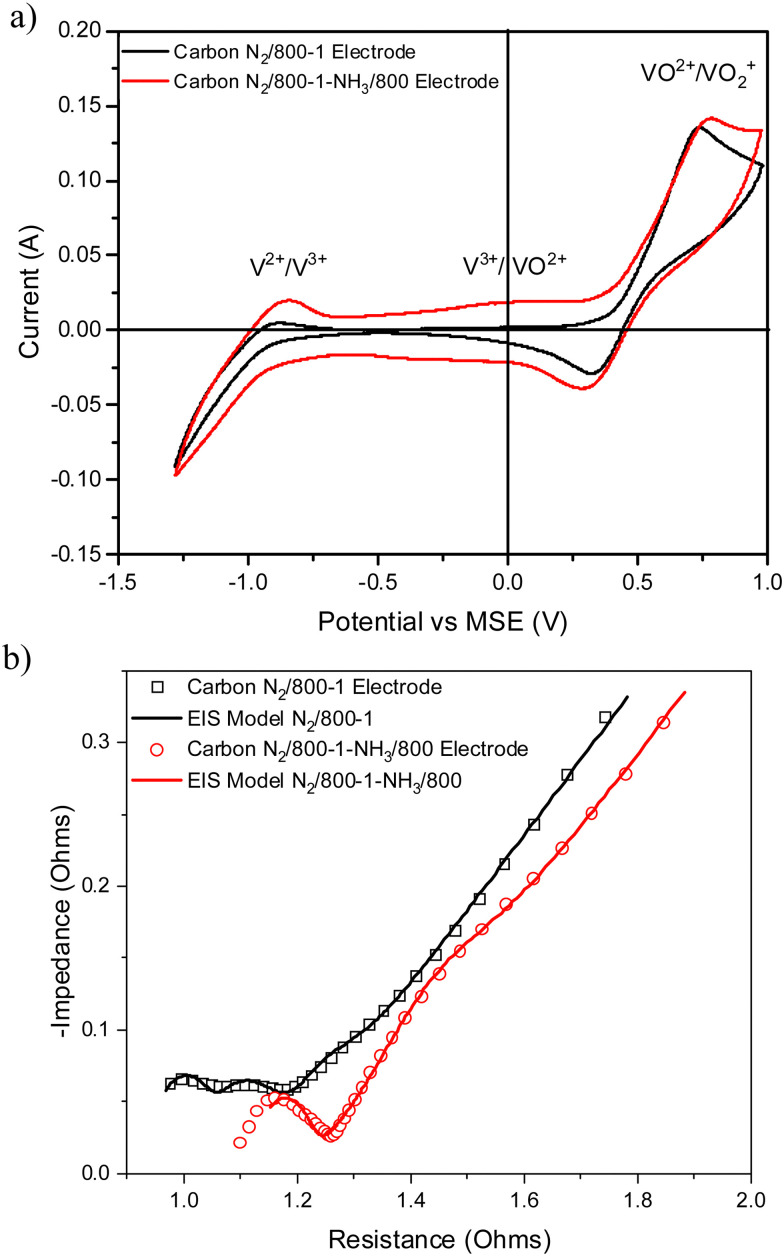
Comparison of electrochemical results for electrodes prepared from carbons N_2_/800-1 and N_2_/800-1-NH_3_/800 (a) cyclic voltammograms and (b) Nyquist plots and corresponding models.

Carbon N_2_/800-1 was treated with NH_3_ to form carbon N_2_/800-1-NH_3_/800, and the cyclic voltammograms and Nyquist plots for the corresponding electrodes for vanadium redox reactions are shown in Fig. 11a and b, respectively. The V^3+^ → VO^2+^ transition is absent for the CV profile of carbon N_2_/800-1 but was observed as a very broad peak at ∼0.1 V in N_2_/800-1-NH_3_/800. Also, the V^2+^ → V^3+^ CV peak increased more than the VO^2+^ →VO_2_^+^ peak. The EIS profile of the N_2_/800-1-NH_3_/800 electrode was shifted to a higher resistance compared to the N_2_/800-1 electrode (Table S1, ESI†). The two high-frequency weak peaks in the EIS of the N_2_/800-1 electrode coincide with a peak for the N_2_/800-1-NH_3_/800 electrode, consistent with a more homogeneous surface in the NH_3_ treated carbon. Also, a weak low frequency peak at ∼2 Hz EIS associated with diffusion/kinetics was present as a clear shoulder.

Ammonia treatment had a marked effect on carbon functional groups but a minimal effect on the predominantly ultramicroporous and molecular structures compared with carbon N_2_/800-1. The original carbon N_2_/800-1 had very low surface and bulk nitrogen contents, while the surface oxygen and bulk contents were much higher. The NH_3_ treatment of carbon N_2_/800-1 at 600 and 800 °C incorporates mainly pyridinic and pyrrolic nitrogen groups (ratio ∼2 : 1) into the carbon structure, with the surface analysis giving 2.56 and 4.86 at% for carbons N_2_/800-1-NH_3_/600 and N_2_/800-1-NH_3_/800, respectively (Table 5).

### 3.6. Electrode performance

**Fig. 12:**
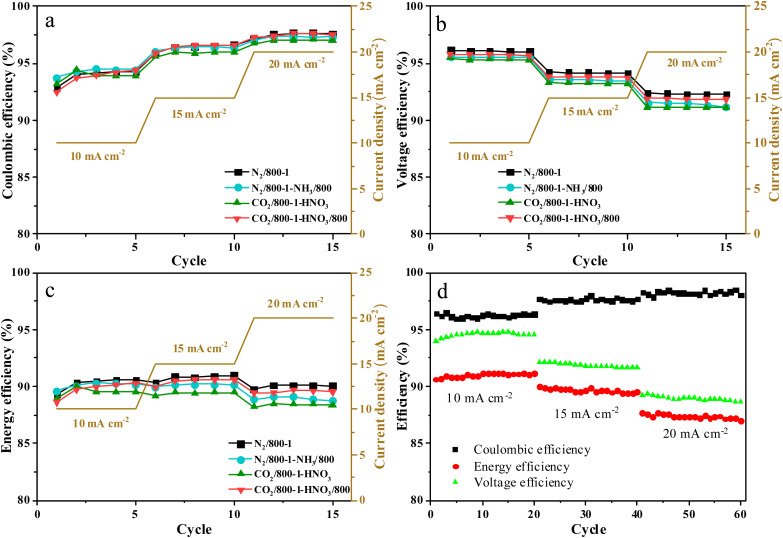
Comparison of cell performance for carbons N_2_/800-1, N_2_/800-1-NH_3_/800, CO_2_/800-1-HNO_3_ and CO_2_/800-1-HNO_3_/800. (a) Coulombic, (b) voltage, (c) energy efficiency, and (d) stability test of N_2_/800-1 for 20 cycles at each current density of 10, 15, and 20 mA cm^−2^.

VRFB static cell charge–discharge analysis was performed at current densities of 10, 15, and 20 mA cm^−2^ to evaluate the electrochemical performance of the carbons (see Fig. 12) Repeatability studies are shown in ESI,† Tables S2 and S3. The coulombic efficiency increases with increasing current density whereas the voltage efficiency decreases with increasing current density. It is apparent that the four carbon samples (N_2_/800-1, N_2_/800-1-NH_3_/800, CO_2_/800-1-HNO_3_, and CO_2_/800-1-HNO_3_/800), which had a range of functional groups, had similar voltage, coulombic and overall energy efficiencies. Stable energy efficiencies of all carbon samples were observed in the range of 89.5–90.9% at current densities of 10–15 mA cm^−2^ before slightly decreasing to 88.4–90.1% at 20 mA cm^−2^, but the overall efficiency differences are comparable to the experimental uncertainties. The slight decrease in energy efficiencies with increasing current densities can be ascribed to ohmic polarization,^87^ dynamic voltage (IR) drop at higher current densities,^13,19^ or electrolyte ions passing through the membrane.^88^ It also explains the decline of voltage efficiencies with increasing current densities. The CV and EIS results for electrodes prepared from carbons N_2_/800-1-NH_3_/800 and CO_2_/800-1-HNO_3_ demonstrated the influences of nitrogen and oxygen carbon functionality on the V^2+^/V^3+^ and VO^2+^/VO_2_^+^ couples. The microporosity in these carbons (ultra-micropores account for >75% of total pore volume) restricts the liquid phase diffusion of vanadium ions into the porous structure of the carbons, which explains the negligible differences in charge–discharge performance in these samples. The performance of walnut shell carbons prepared in this study is compared with that of other carbon materials using VRFB static cells in Table 9. The slightly lower energy efficiency of walnut shell carbons compared to orange peel carbon^13^ and spent coffee bean carbon^20^ is attributed to the high ultramicropore content limiting diffusion of vanadium ions in the carbon structure. The energy efficiency for walnut shell carbon demonstrated that it is suitable for VRFB electrodes. Carbon N_2_/800-1 had the highest efficiency and was subjected to extended tests for 20 cycles at each current density of 10, 15, and 20 mA cm^−2^, which are presented in Fig. 12d. The coulombic efficiency increases whereas the voltage efficiency decreases with increasing current density. The overall energy efficiency decreases with increasing current density, possibly due to the side reactions at each half-cell.^89^ The stable performance of walnut shell carbons suggests their suitability for use in VRFBs. The coulombic, voltage, and overall energy efficiencies of all the carbons (N_2_/800-1, N_2_/800-1-NH_3_/800, CO_2_/800-1-HNO_3_ and CO_2_/800-1-HNO_3_/800) studied were not significantly different within experimental error, and therefore longer-term stability tests were not undertaken.

**Table 9:** Comparison of coulombic, voltage efficiency, and energy efficiency for walnut shell carbon with literature studies^13,19,90–94^

Activated carbon material	Coulombic efficiency (%)	Energy efficiency (%)	Voltage efficiency (%)	Ref.
Spent tea leaf AC	98	90	92	90
Sal wood sawdust AC	95.6	91.2	95.4	19
Orange peel AC	99.8	95.5	95.6	13
Spent coffee beans based – reduced graphene oxide	98	93	94	91
Nitrogen-doped reduced graphene oxide – graphite felt	92.09	76.08	82.62	92
Nitrogen-doped graphite felt	94.20	86.47	91.79	93
Oxygen-functionalized carbon nanofiber	—	75.5	—	94
Walnut shell carbon N_2_/800-1	96.5	90.9	94.2	This study

### 3.7. The influence of carbon structural characteristics on vanadium redox reactions

Developing relationships between carbon surface chemistry, molecular structure, and porosity, with electrochemical characteristics, is essential for understanding the mechanism. Several investigations have focused on the relationship between electrochemistry and electrode properties for vanadium Redox reactions.^9–11,13,14,17–20,27,94–115^ In concentrated VRFB-relevant electrolyte solutions, polymerization, hydrolytic, and ion association processes are often extensive. Also, the capacities of VFBRs decay with use, and surface reactions are thought to be involved.^26,116^ The role of surface carbon groups on the electrode is unclear.^27^ The problem with investigating the roles of surface chemistry and carbon structure for vanadium redox electrochemical characteristics is that there is often a mixture of oxygen surface functional groups and a variety of carbon sites in an amorphous porous structure. Oxygen functional groups could enhance the wettability of carbons and act as electro-catalytic sites for redox reactions,^100–102,105,114^ but the influence of oxygen functional groups was only observed for carbon materials with low surface area or well-defined structure. Studies of the vanadium redox electrochemistry of polyacrylonitrile electrospun fibers led to the proposal that functional groups were the dominant factor for carbonization temperatures in the range 1000–1300 °C, but carbon edge sites were more critical over the temperature range 1300–1500 °C.^100^ Nitrogen and oxygen functional groups were incorporated into graphite felt by wet impregnation of urea followed by heat treatment in nitrogen to 500–700 °C. Electrochemical results suggested that pyrrolic and pyridinic functional groups were active centers for vanadium redox reactions.^95^ Li *et al*. attributed enhanced kinetics for V^2+^/V^3+^ to C–OH groups incorporated on the surface of carbon felt following thermal activation.^101^ This commercial carbon felt had a low surface area and was thermally resilient, and the improved kinetics of V^2+^/V^3+^ were attributed to the increase of oxygen functional groups. The enhanced kinetics of vanadium redox reactions have been ascribed to carboxyl groups present in multi-walled carbon nanotubes.^102^

Simulation studies showed that nitrogen incorporated in the carbon structure had a positive impact on vanadium redox reactions.^10^ Shao *et al*. attributed the enhanced VO^2+^/VO_2_^+^ couple (the positive side of VRFB) to nitrogen doping in the mesoporous carbon. However, the BET surface area could have contributed significantly to their performance as it increased from 500 m^2^ g^−1^ for the untreated material to 1100 m^2^ g^−1^ for the NH_3_ treated carbon.^104^ The improvement in CV profiles of biomass-derived carbon was attributed to the mesopore structure.^13,14^ Costa de Oliveira *et al*. used voltammetry and EIS methods to reveal that N-functionalities significantly enhance the intrinsic activity of carbon electrode surfaces.^117^ Pyridinic/pyrrolic groups improved charge transfer rates and reversibility of the vanadyl oxidation reaction. These factors are crucial for important applications such as vanadium redox flow batteries. In comparison, oxygen functional groups may negatively impact vanadyl redox reactions.^118^

Steimecke *et al*. used scanning electrochemical microscopy (SECM) to investigate the VO^2+^/VO_2_^+^ reaction for various oxidized carbon materials.^119^ Electron transfer coefficients of glassy carbon (GC) and graphite substrates and their oxidized derivatives were comparable to cyclic voltammetry. In the case of oxidized samples, the results depended on the material. A small improvement was observed for oxidized GC whereas no effect was observed for graphite where the oxygen content increases from 4.0 to 13.0 at%. A combined *in situ*-Raman-SECM was also performed for a graphene layer. The graphene edge sites had increased activity at relatively low overpotentials for VO^2+^ oxidation and at high overpotentials for VO_2_^+^ reduction compared with basal plane carbon sites.

The relationship between vanadium redox reactions and carbon properties, the active electrode component of the carbon/PVDF electrode, is a multifactorial problem and is not well understood. In this study, the effects of carbonization or heat treatment temperature, porous structure, and functional groups have been considered in detail. The carbons in Series 1 and 2 used in this study were predominantly ultramicroporous (<0.7 nm), and, therefore, carbon porosity was not a factor influencing the CV measurements of vanadium redox reactions due to diffusion limitations. However, the porosity in the composite electrode formed between the polymer and the carbon is difficult to quantify. Carbon surface chemistry may influence porosity formation in the carbon/PVDF electrodes. There is no evidence that differences in the porous structure of the carbons used in this study are significant in determining EIS characteristics. Low frequency peaks in EIS may be associated with porosity in the carbon/PVDF electrodes. Carbon surface chemistry is known to alter the resistivity in carbon black/polymer composites.^85,86^

Cyclic voltammograms reported in the literature show that the relative peaks for V^2+^ → V^3+^, V^3+^ → VO^2+^, and VO^2+^ → VO_2_^+^ vary markedly depending on the structure of the carbon electrodes and the CV sweep rates, indicating kinetic effects.^13,14,110^ The vanadium V^3+^/VO^2+^ couple has potentiometric reversibility, but there is a large activation energy, and therefore, the kinetics are slow. Sufficiently negative potentials are required to bring the reduction of V(iv) to V(iii), but in most cases, the negative potential is low enough to cause the reduction all the way to V(ii). The V^3+^/VO_2_^+^ couple influences the initial pre-charging of the electrolyte.^109^ Previous studies of pre-oxidized glassy carbon electrodes showed that increased surface roughness was beneficial, while functional groups such as C=O and C–O–C tend to reduce the activity of glassy carbon.^114^ Three concurrent processes occur, (1) slow V(iii) → V(iv), (2) oxidation V(iv)→ V(v), and (3) reaction of V(iii) and V(v) to form V(iv) species.^109^ The reversibility of the CV for V^3+^/VO_2_^+^ was improved by increased vanadium concentrations or oxygen functional groups.^109^ Various hydrolytic and ion association processes may occur in concentrated vanadium electrolyte solutions, and chemical stability is a factor.^27,111,112^ The connection between vanadium speciation in the electrolyte and surface groups in carbon electrodes is unclear. The relationship between vanadium species present in the electrolyte, electrode surface groups, and electrochemical kinetics needs systematic investigation. It has been proposed that the anodic reaction limits kinetics even at low current densities, and the cathodic reaction has mass transport effects at high current densities.^27,113^

This study investigated the use of sustainable biochar electrodes for vanadium redox reactions. Biomass pyrolysis involves the overlapping decomposition of lignocellulosic materials to form a carbonaceous char by an HTT of 600 °C. As the temperature increases, structural change leads to carbons with heterogeneous surfaces, which contain a wide range of oxygen and nitrogen surface sites and graphene layer sites, including zig-zag and armchair sites with unsatisfied valences at the edges of these layers. The heterogeneous amorphous structure of carbon leads to difficulties in structural characterization, and the techniques available have limitations. Therefore, it is very challenging to quantify the carbon structure of biochars.

The carbons used in this study were sustainable, amorphous, and non-graphitizable biochars, and quantifying the carbon structure is complicated. All carbons in Series 1 and 2 had a high proportion of ultramicroporosity (<0.7 nm), indicating diffusion limitations for liquid phases. XPS C 1s and O 1s studies indicate that surface oxygen functional groups are similar within experimental error for heat-treated carbons in Series 1 and Series 2 (Table S5, ESI†), although some differences are observed in the characterization data using titration methods (Table 6). Therefore, differences in porous structure characteristics and surface oxygen functional groups are not responsible for the significant changes in CV and EIS with HTT. The effect of HTT on carbon structure involves the loss of volatile decomposition products, as shown in the thermogravimetric and TPD data. The Raman characterization parameters show more significant changes in peak intensity (*I*_D_/*I*_G_) and area (*A*_D_/*A*_G_) ratios and D bandwidths for the HTT range 600–800 °C than for 800–1000 °C. The apparent crystallite size (*L*_a_) changes progressively with HTT. The most significant changes in the CVs for vanadium redox reactions were observed for both Series 1 and Series 2 carbons for HTTs in the 600–800 °C temperature range with minor changes for HTT 800–1000 °C, and this change was independent of CV sweep rate. The electrical impedance measurements show that the electrode resistance reaches a minimum for carbons in Series 1 and 2 with a HTT of 800 °C (see Fig. 8b and d). These changes in carbon electrochemical properties coincide with the carbon HTT for maximum CVs. The electrodes used are carbon/PVDF polymer composites, and the resistivity of these materials depends on carbon structure, polymer matrix, and the volume fraction of carbon. Resistivity is related to the extent of conductive pathways between carbon particles, and carbon composite electrodes must be defect-free. The volume resistivity of the composite may be affected by both the surface groups and the carbon structure. Previous studies have reported carbon/polymer composite volume resistivity measurements, which change with the extent of carbon black gasification and surface functional groups incorporated by HNO_3_ treatment.^85,86^ The CV reaches a maximum, and the electrode resistance from EIS measurements a minimum at ∼800 °C. Therefore, it is proposed that the electrical conductivity of the carbon particles in the electrode is responsible for the major changes in CV with HTT in Series 1 and 2 carbons.

A requirement for assessing the impact of oxygen and nitrogen functional groups incorporated into carbon structure on electrochemistry was that the HTTs should not be exceeded by the treatment temperature since this could cause further carbon molecular structural change, which could obscure the impact of functional groups. Carbons N_2_/800-1 and CO_2_/800-1 were chosen because the cyclic voltammograms do not change markedly for HTT > 800 °C. All the carbons used were predominantly ultramicroporous, and the NH_3_ and K_2_CO_3_ functionalization treatment conditions were also 800 °C. The maximum heat treatment of carbon CO_2_/800-1-HNO_3_ was also limited to 800 °C. All the treatment procedures had minimal effects on the porous and molecular structures. The main difference observed in the CV for the functionalized carbons was the enhancement of the V^3+^/VO^2+^ couple, and this was ascribed to the interaction of the nitrogen and oxygen surface groups with V(H_2_O)_6_^3+^ and VO(H_2_O)_5_^2+^.

**Fig. 13:**
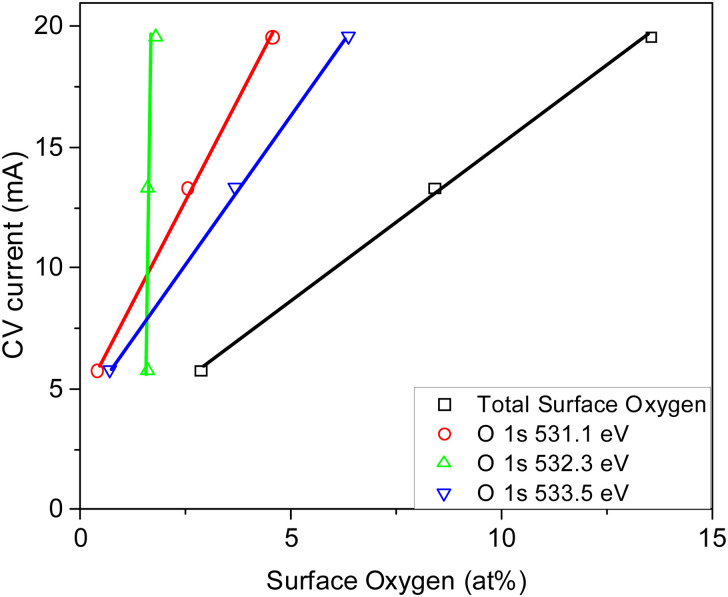
Comparison of cyclic voltammetry for the HNO_3_ functionalized carbon series and XPS oxygen surface analysis and functional groups obtained from O 1s spectra.

Oxidation of carbon CO_2_/800-1 with HNO_3_ to form carbon CO_2_/800-1-HNO_3_ increased the CV V^3+^/VO^2+^ couple, and this was reversed by heat treatment in H_2_ at 400 and 800 °C. The EIS showed that the electrode resistances were only changed slightly by functionalization. In this series of carbons, surface oxygen groups were incorporated by HNO_3_ oxidation, and labile oxygen surface oxygen groups with different thermal stabilities were removed by heat treatment in H_2_. The changes in CV followed corresponding changes in surface oxygen functional groups, with only minor molecular structure changes observed. The series had a wide range of bulk oxygen contents from chemical analysis (7.54–23.84 wt%, see Table 1) and surface oxygen contents from XPS (2.85–13.54 at%, see Table 3), and the surface and bulk oxygen contents have a linear correlation (*R*^2^ = 0.976) (Fig. S43, ESI†). Fig. 13 shows that the CV changes linearly with surface oxygen (*R*^2^ = 0.999) and the oxygen groups with peaks at 531.1 eV (C=O) (*R*^2^ = 0.999) and 533.5 eV (O–C attached to aromatic groups) (*R*^2^ = 0.999). However, the peak at 532.3 eV, ascribed to O–C aliphatic groups, does not change markedly (*R*^2^= 0.624), which suggests that O–C aliphatic groups are not responsible while carboxylic groups are involved. Various functional groups, for example, carboxylic, lactone, *etc.*, have these types of oxygen. The XPS peak at ∼535.6 eV also showed a linear correlation (*R*^2^ = 0.992) with CV current, but this only represented ∼5% of the total O 1s peak area. The XPS C 1s peak (289 eV) shows that the carboxylic acid groups were 6 at% in carbon CO_2_/800-1-HNO_3_, 1.3 at% in CO_2_/800-1-HNO_3_/400, and 0.19 at% in CO_2_/800-1-HNO_3_/800 (Fig. S43, ESI†) and this is due to thermal decomposition. The intensity of this XPS C 1s peak has a linear correlation with carboxylic groups determined by titration methods (*R*^2^ = 0.999, See Section 3.3.2 and Fig. S43, ESI†). The decomposition of the COOH groups is supported by the loss of CO_2_ in TPD (see Fig. 4) and the loss of an FTIR C=O carboxylic peak observed at 1713 cm^−1^ for carbon CO_2_/800-1-HNO_3_ (Fig. S41d, ESI†). The titration measurements showed that the weakly acidic phenolic groups had little or no effect on the CV for the carbon CO_2_/800-1-HNO_3_ oxidation series. The TPD results show the decomposition of *N*-oxide groups, carboxylic, and other oxygen groups with CO, CO_2_, and NO loss. Comparison of the XPS N 1s of carbons CO_2_/800-1-HNO_3_ and CO_2_/800-1-HNO_3_/400 showed that the *N*-oxide peak had decomposed at 400 °C. (Fig. S27 and Table 5, ESI†). Previous studies have shown that the adsorption of metal ions on carbon was dramatically increased by HNO_3_ oxidation, and carboxylic acid groups were shown to strongly adsorb various M^2+^ ions, where M^2+^ = Cd, Pb, Hg, and Ca through ion exchange.^33,120^ The adsorption of metal ions was decreased significantly by heat treatment of the carbon. This established a relation between carboxylic acid functional groups and metal ion adsorption.^33^ Adsorption of vanadium species on surface carboxylic oxygen groups is a possible interaction mechanism that explains trends in the CVs and Nyquist plots. The underlying process at high potential is most likely the kinetically limited V(iii) to V(iv) oxidation and then proceeding to V(v). The oxidation of V(iii) to V(iv) only occurs on a limited number of oxygen surface sites, and the outer-sphere electron transfer to oxidize V(iii) takes place at much more positive potentials.

K_2_CO_3_ treatment of carbon CO_2_/800-1 at 800 °C enhanced the CV peaks, particularly the V^3+^/VO^2+^ couple, and EIS showed that the electrode resistance increased slightly. Since both carbons CO_2_/800-1 and CO_2_/800-1-K_2_CO_3_/800 have similar ultramicroporous (Table 2) and molecular structures (Tables 7 and 8), the changes in CV are attributed to differences in functional groups. The XPS showed a small decrease in surface oxygen groups from 8.3 at% for carbon CO_2_/800-1 to 7.07% for CO_2_/800-1-K_2_CO_3_/800. Comparison of XPS C 1s spectra of carbons CO_2_/800-1 and CO_2_/800-1-K_2_CO_3_/800 did not show significant differences in the distribution of surface functional groups. The XPS O 1s scan showed a decreased C=O intensity (531 eV), while the CO/OH attached aliphatic (532.2 eV) and C-O attached to aromatic (533.5 eV) increased significantly with the K_2_CO_3_ treatment. In comparison, the HNO_3_ oxidation series showed that the 531.1 and 533.5 eV peaks correlated with a change in CV, whereas the 532.3 eV peaks did not. However, this must be considered in the context that some of the functional groups incorporated by chemical oxidation are thermally labile, whereas the K_2_CO_3_ treatment was carried out at 800 °C. A comparison of carbons with the same HTT shows that carbons CO_2_/800-1-HNO_3_/800 and CO_2_/800-1 have main TPD CO_2_ peaks at 825 and 818 °C. The TPD for carbon CO_2_/800-1-K_2_CO_3_/800 shows CO_2_ desorption starting at ∼550 °C with peaks at 725 and 875 °C. The TPD peak at 725 °C does not coincide with the desorption of H_2_O or CO. The titration results showed carbon CO_2_/800-1-K_2_CO_3_/800 had acidic (mainly phenolic and carboxylic with a small amount of lactone/lactol) and basic groups, whereas carbons CO_2_/800-1 only had basic groups and CO_2_/800-1-HNO_3_/800 had phenolic and some basic amphoteric characteristics. This suggests that the difference is due to the CO_2_ peak at 725 °C being associated with the strongly acidic groups as characterized by titration are responsible for changes in CV. The FTIR results do not show strong C=O stretching bands in the 1600–1800 cm^−1^ region, indicating the absence of carboxylic and anhydrides, but the spectra are weak. The characterization techniques show that the TPD CO_2_ desorption peak at 725 °C, the presence of acidic functional groups, and the increased intensity XPS O 1s 533.5 eV peak are related to changes in CV. The oxygen functional groups act as sites in carbon CO_2_/800-1-K_2_CO_3_/800 for oxidizing V(iii) to V(iv).

Treatment of carbon N_2_/800-1 with ammonia at 800 °C to form carbon N_2_/800-1-NH_3_/800 has a significant effect in increasing the V^3+^ → VO^2+^ CV peak, which was not observed for the starting carbon N_2_/800-1 and increases the V^2+^ → V^3+^ CV peak more than the VO^2+^ → VO_2_^+^peak. The EIS showed that NH_3_ treatment increased electrode resistance. NH_3_ treatments decreased the surface oxygen from 8.1 at% in carbon N_2_/800-1 to 2.7 and 1.8 at% in carbons N_2_/800-1-NH_3_/600 and N_2_/800-1-NH_3_/800, respectively. Curve resolution of the XPS C 1s and O 1s peaks show that the distribution of the oxygen functional groups for carbons N_2_/800-1, N_2_/800-1-NH_3_/600, and N_2_/800-1-NH_3_/800 were similar. XPS N 1s studies show that the NH_3_ reaction incorporates mainly pyridinic and pyrrolic nitrogen groups into the carbon structure with a ratio ∼2 : 1 with total nitrogen 2.56 at% for 600 °C and 4.86 at% for 800 °C. The titration measurements show that the surface of carbon N_2_/800-1-NH_3_/800 had amphoteric characteristics with weakly acidic phenolic groups and basic pyridinic groups on the surface of carbon N_2_/800-1-NH_3_/800. The basic characteristics were slightly higher for N_2_/800-1-NH_3_/800 carbon than N_2_/800-1 due to the pyridinic groups.

Surface nitrogen functional groups in activated carbons incorporated by high-temperature ammonia treatment increased the adsorption of capacities of M^2+^ metal ion species (M = Cd, Ni, and Cu), and it was proposed that pyridinic groups were responsible.^34^ A similar interaction of vanadium species with pyridinic groups may explain the differences between the CVs and Nyquist plots before and after NH_3_ treatment. The changes in electrochemistry can be attributed to incorporating nitrogen as pyridinic and pyrrolic groups into the carbon structure while reducing surface oxygen groups markedly, which catalyzes V^3+^ oxidation on the surface of electrodes. Pyridinic groups are the major nitrogen functional groups in carbon N_2_/800-1-NH_3_/800.

## 4. Conclusions

The influence of the structural and surface chemical characteristics of sustainable biomass-derived carbons on vanadium redox reactions has been explored in this study. The two series of walnut shell-derived carbons made in N_2_ and CO_2_ atmospheres were predominantly ultramicroporous, and therefore, the porous structures had little or no effect on the vanadium redox electrochemical characteristics because of diffusion limitations. The XPS C 1s and O 1s showed minimal changes in surface oxygen functional groups. The effect of carbonization temperature on carbon molecular structure was shown by changes in Raman and X-ray diffraction parameters. The most intense couple in the CV for the vanadium redox electrochemistry was VO^2+^/VO_2_^+^, where the current increased markedly for carbonization under N_2_ and CO_2_ in the temperature range of 600–800 °C. However, only minimal changes were observed when increasing the HTT from 800 °C to 1000 °C. These changes are the most significant in cyclic voltammetry. Electrical impedance measurements showed that the electrode resistance reached a minimum at HTT 800 °C. Biochar carbonization temperature has the primary effect on electrochemical properties. This effect is attributed to the modification of the carbon molecular structure, increasing electrical conductivity over the HTT range of 600–800 °C.

The role of surface groups in carbon structures was investigated by chemical oxidation and high-temperature treatment methods. Carbons with an HTT of 800 °C were used for functionalization studies since the cyclic voltammograms are close to the maximum CV current and avoid the effects of molecular structural change due to HTT. Oxidation of carbon CO_2_/800-1 with nitric acid resulted in incorporating a mixture of labile acidic carboxylic, lactone, lactol, and anhydride functional groups into the carbon structure, and the CV current of the V^3+^/VO^2+^ couple also increased significantly. Minor changes in CV were also observed for the V^2+^/V^3+^ and VO^2+^/VO_2_^+^ couples. These changes in CV were partly reversed when the oxidized carbon sample was heat treated in H_2_ to 400 °C, removing carboxylic groups. Thermolysis at 800 °C in H_2_ removed further oxygen functional groups, with the CV and Nyquist graphs being similar to the original carbon CO_2_/800-1, indicating some reversibility. The changes in the CV for the V^3+^/VO^2+^ couple are related to changes in surface oxygen in the carbons. The relationship between surface carboxylic groups and CV changes, as shown by XPS, is supported by titration, TPD, and FTIR measurements. The underlying process at high potential is most likely the kinetically limited V(iii) to V(iv) oxidation and then to V(v). The oxidation of V(iii) to V(iv) only occurs on a limited number of oxygen surface sites, and the outer-sphere electron transfer to oxidize V(iii) takes place only at much more positive potentials.

Similarly, electrodes prepared with carbon CO_2_/800-1-K_2_CO_3_/800 had small increases in the CV currents for V^2+^/V^3+^, V^3+^/VO^2+^, and VO^2+^/VO_2_^+^ couples compared with electrodes made from carbon CO_2_/800-1. XPS, titration, and TPD studies showed that K_2_CO_3_ treatment incorporated thermally stable acidic surface oxygen functional groups in the carbon structure. The changes in cyclic voltammetry were attributed to changes in oxygen functional groups.

NH_3_ treatment of carbon N_2_/800-1 at 600 and 800 °C resulted in the incorporation of nitrogen mainly as surface pyridinic and pyrrolic groups in the carbon structure and the removal of a large proportion of surface oxygen groups. The CV for carbon N_2_/800-1-NH_3_/800 showed the presence of a broad V^3+^/VO^2+^ peak absent in the starting material carbon N_2_/800-1. Incorporating nitrogen functional groups into the carbon structure has a similar effect to incorporating oxygen functional groups into the carbon structure and involves catalysis of V^3+^/VO^2+^ oxidation on the nitrogen surface sites of the electrodes.

The coulombic, voltage, efficiency, and energy efficiency for walnut shell carbons were suitable for VFRBs, and good stability was observed. The functionalization of the carbons only had minor effects on voltage, efficiency, and energy efficiency for both the HNO_3_ oxidized and NH_3_ treated carbons. The study of carbon structure and functional group relationships with vanadium redox electrochemistry supports the development of improved electrode materials for vanadium redox flow batteries.

## Author contributions

Ha H. Phan: investigation, methodology, data curation and analysis, original draft. Jon G. Bell: supervision (Porosity Characterization). Greg A. Mutch: investigation (Raman measurements). Alan J. McCue: investigation (FTIR measurements). Anh N. Phan: conceptualization, supervision, discussion, analysis, writing, reviewing, editing. K. Mark Thomas: conceptualization, supervision, discussion, formal analysis, modelling, writing, reviewing, editing.

## Data availability

ESI† includes all the significant data for the paper in graphical or table format. The data that support the findings of this study are available from the corresponding author upon request.

## Conflicts of interest

The authors declare no competing financial interest.

## Supplementary Material

MA-005-D4MA00675E-s001
