# Supplementary material for: Spectroelectrochemical study of carbon structural and functionality characteristics on vanadium redox reactions for flow batteries
Source: Mater Adv. 2024 Aug 15;5(18):7170–98. doi: 10.1039/d4ma00675e (PMC11325216; doi:10.1039/d4ma00675e)
Supplement: MA-005-D4MA00675E-s001 [file MA-005-D4MA00675E-s001.pdf]

Supplementary Information (SI) for Materials Advances.
This journal is © The Royal Society of Chemistry 2024

Spectroelectrochemical Study of Carbon Structural and Functionality Characteristics on Vanadium Redox Reactions for Flow Batteries

Ha H. Phan,[1] Jon G. Bell,[1] Greg A. Mutch,[2] Alan J McCue[3], Anh N. Phan,[1]* and K. Mark Thomas[1]*

[1] Wolfson Northern Carbon Reduction Laboratories, School of Engineering, Newcastle University, Newcastle upon Tyne NE1 7RU, United Kingdom

[2] School of Engineering, Newcastle University, Newcastle upon Tyne NE1 7RU, United Kingdom

School of Chemistry, University of Aberdeen, Aberdeen AB24 3UE, United Kingdom

*Corresponding authors

Contents

Page

1. **Gas Adsorption Characteristics** 5

1.1 **Carbonization in a Nitrogen Atmosphere (Series 1)** 5

1.1.1 Carbon Dioxide Adsorption and Desorption Isotherms at 0°C for the Series of Carbons prepared under a Nitrogen Atmosphere 5

1.1.2 Dubinin-Radushkevich Graphs for Carbon Dioxide Adsorption at 0°C for the Series of Carbons prepared under a Nitrogen Atmosphere 5

1.1.3 Carbon Dioxide Adsorption Kinetic Profiles for the Series of Carbons Prepared under a Nitrogen Atmosphere. 7

1.2 **Carbonization in a Carbon Dioxide Atmosphere (Series 2)** 9

1.2.1 Nitrogen Adsorption and Desorption Isotherms at -196°C for the Series of Carbons prepared under Carbon Dioxide Atmosphere 9

1.2.2 BET graphs and Mesopore Pore Size Distributions for Nitrogen Adsorption and Desorption at -196°C for the series of Carbons prepared under a Carbon Dioxide Atmosphere. 11

1.2.3 Dubinin-Radushkevich graphs for Nitrogen Adsorption at -196°C for the series of Carbons prepared in a Carbon Dioxide Atmosphere. 17

1.2.4 CO_2 Adsorption and Desorption Isotherms at 0°C for the Series of Carbons prepared in a Carbon Dioxide Atmosphere. 19

1.2.5 Dubinin-Radushkevich graphs for Carbon Dioxide adsorption at 0°C for the Series Carbons prepared in a Carbon Dioxide Atmosphere. 20

1.3 Chemical Treatment of Carbon for Functional Group Modification 23

1.3.1 Nitrogen Adsorption at -196°C for Functionalized Carbons. 23

1.3.2 BET Graphs and Mesopore Pore Size Distributions for Nitrogen Adsorption at -196°C on Functionalized Carbons. 25

1.3.3 Dubinin-Radushkevich graphs for Nitrogen Adsorption at -196°C on Functionalized Carbons. 31

1.3.4 Carbon Dioxide Adsorption Isotherms at 0°C on Functionalized Carbons. 33

1.3.5 Dubinin-Radushkevich Graphs for Carbon Dioxide Adsorption at 0°C for Functionalized Carbons. 35

1.4 Comparison of Gas Adsorption Parameters 38

2. Electrochemistry 40

2.1 Cyclic Voltammetry 40

2.1.1 Comparison of Cyclic Voltammograms with a Sweep Rate of 20mV s^{-1} for Carbon/PVDF Electrodes made from Carbons prepared in Nitrogen and Carbon Dioxide Atmospheres. 40

2.1.2 Comparison of Cyclic Voltammograms for Carbon Electrodes as a function of Sweep Rates for Carbons with a range of Heat Treatment Temperatures. 42

2.1.3 Cyclic Voltammograms as a Function of Sweep Rate. 44

2.1.4 Comparison of Cyclic Voltammograms (Sweep Rate 20 mV s^{-1}) for Electrodes made from Carbons prepared under Nitrogen and Carbon Dioxide Atmospheres with corresponding functionalized Carbons 47

2.2 Modeling of Electrical Impedance Spectra 48

2.3 Cell Performance Repeatability Measurements 54

3. X-ray Photoelectron Spectroscopy 58

3.1 Repeatability Studies 58

3.2 Comparison of X-ray Photoelectron O 1s Spectra for various Hold Times 61

3.3 Comparison of X-ray Photoelectron O 1s Spectra for Series 1 (N_2 Atmosphere) and Series 2 (CO_2 Atmosphere) as a Function of Heat Treatment Temperature 63

3.4 Comparison of X-ray Photoelectron Spectra for Various Treatments Methods with Original Materials 65

3.5 XPS N 1s Spectra for chemically treated and original carbons. 67

3.6 Curve Resolution Data for X-ray Photoelectron Spectra 69

4. Temperature Programmed Desorption 80

5. Thermogravimetric Analysis 82

6. Powder X-ray Diffraction 83

7. Raman Spectroscopy **85**

7.1 Repeatability of Curve Fitting 85

7.2 Raman Spectra, Curve Fitting, and Residuals 90

7.3 Comparisons of Raman, X-ray Diffraction, and Carbonization Temperature Data 103

8. Fourier Transform Infrared Spectroscopy 106

9. Electron Microscopy 109

10. Comparison of Characterization Data for the HNO_3 Functionalized Carbons 110

1. Gas Adsorption Characteristics

1.1 Carbonization in a Nitrogen Atmosphere (Series 1)

1.1.1 Carbon Dioxide Adsorption and Desorption Isotherms at 0°C for the Series of Carbons Prepared under Nitrogen Atmosphere

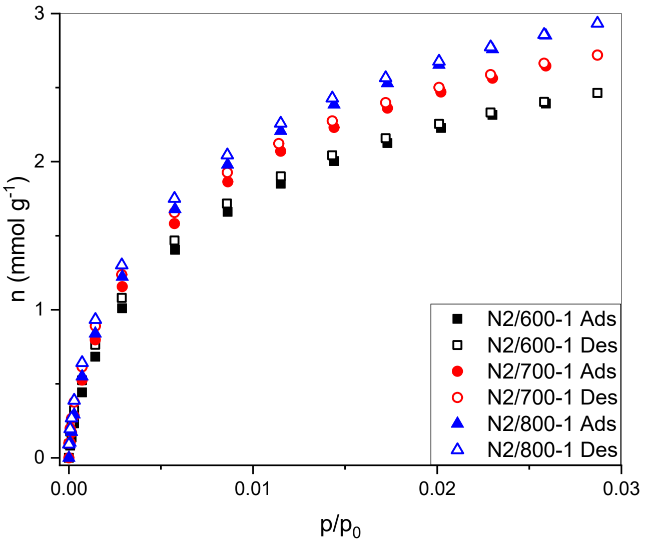

Figure S1 Carbon dioxide adsorption and desorption isotherms for carbons N_2/600-1, N_2/700-1 and N_2/800-1 at 0°C

1.1.2 Dubinin-Radushkevich Graphs for Carbon Dioxide Adsorption at 0°C for the Series of Carbons Prepared under Nitrogen Atmosphere

a)

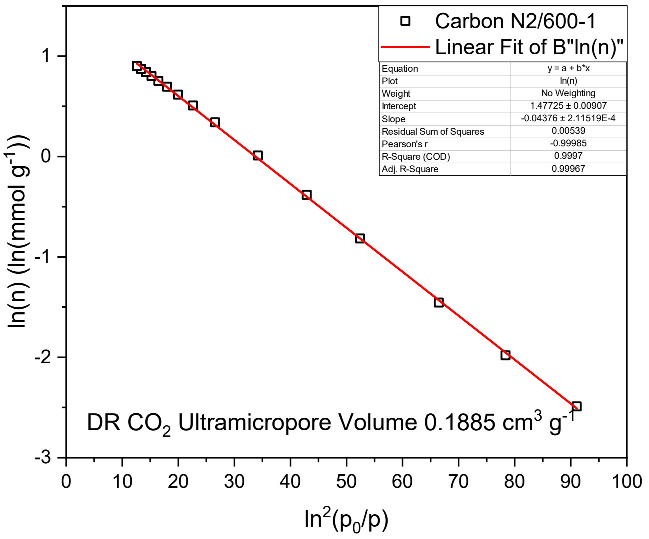

b)

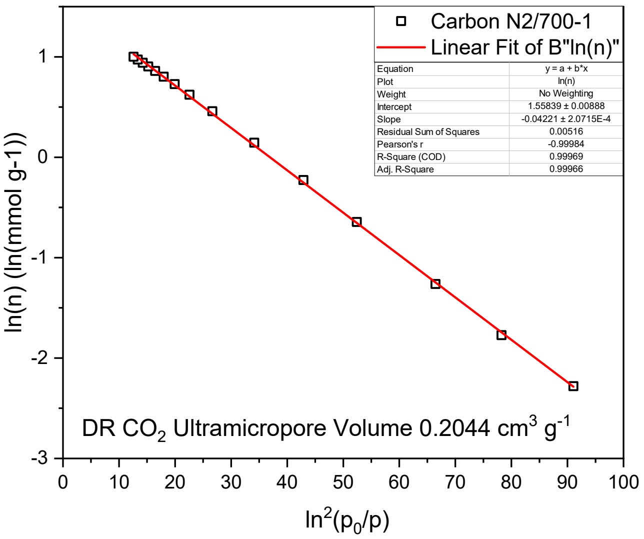

c)

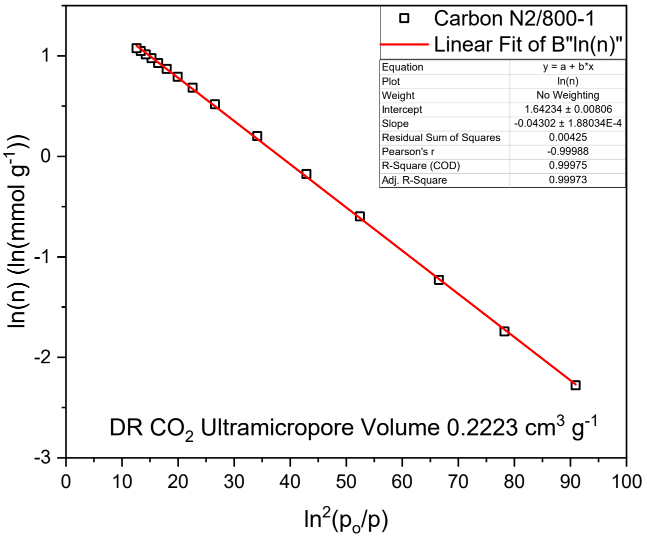

Figure S2. Dubinin-Radushkevich graphs for carbon dioxide adsorption at 0°C on carbons prepared in a nitrogen atmosphere a) Carbon N_2/600-1, b) Carbon N_2/700-1 and c) Carbon N_2/800-1

1.1.3 Carbon Dioxide Adsorption Kinetic Profiles for the Series of Carbons Prepared under a Nitrogen Atmosphere

a)

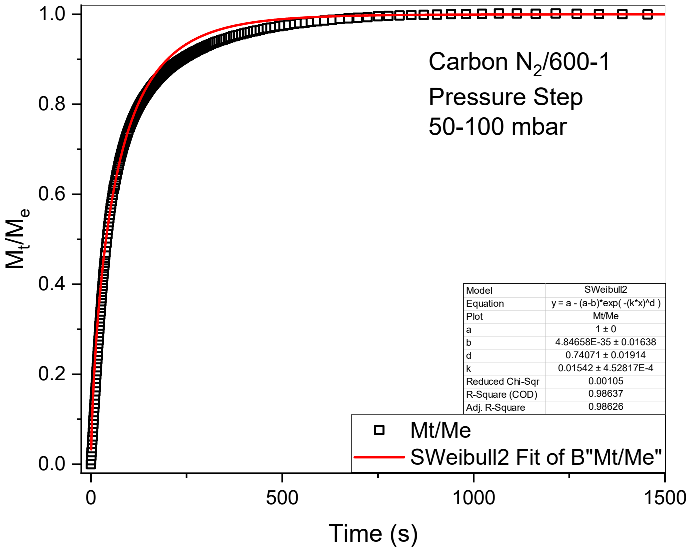

b)

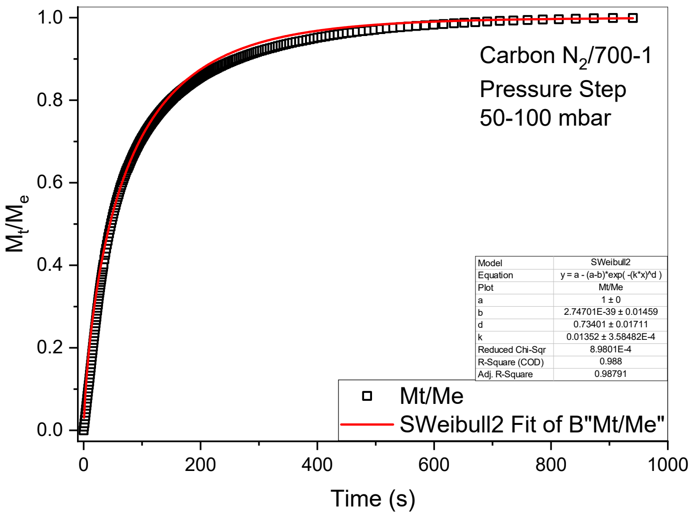

c)

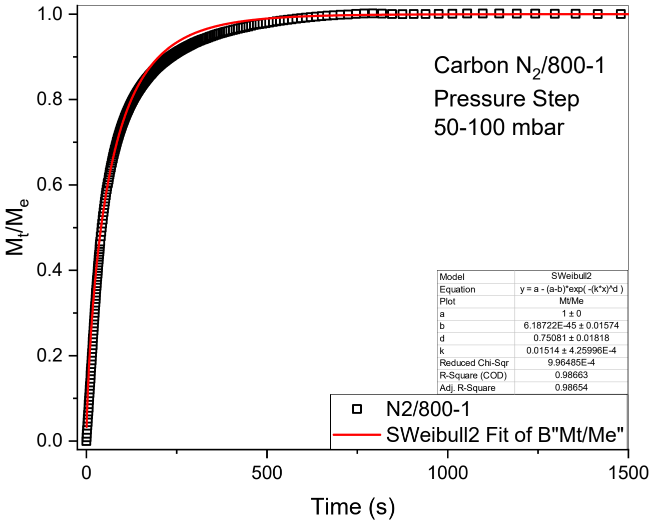

d)

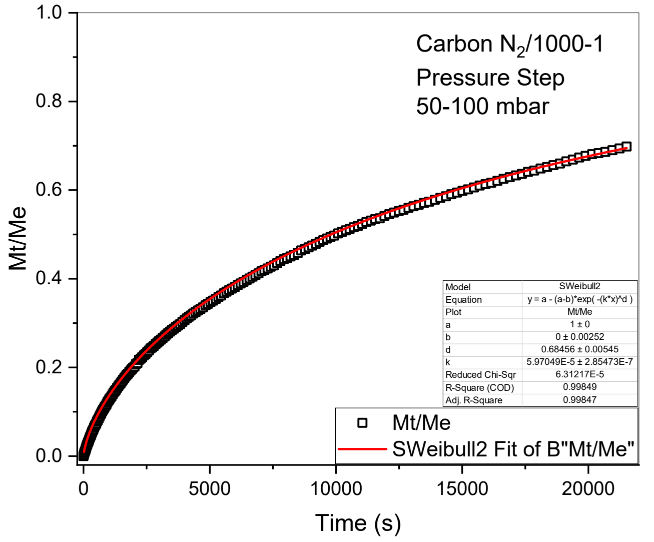

Figure S3 Adsorption kinetics for pressure step 50-100 mbar for CO_2 adsorption on carbons a) Carbon N_2/600-1 (0°C), b) Carbon N_2/700-1 (0°C), c) Carbon N_2/800-1 (0°C) and d) Carbon N_2/1000-1 (30°C).

1.2 Carbonization in a Carbon Dioxide Atmosphere (Series 2)

1.2.1 Nitrogen Adsorption and Desorption Isotherms at -196°C for the Series of Carbons prepared in a Carbon Dioxide Atmosphere

a)

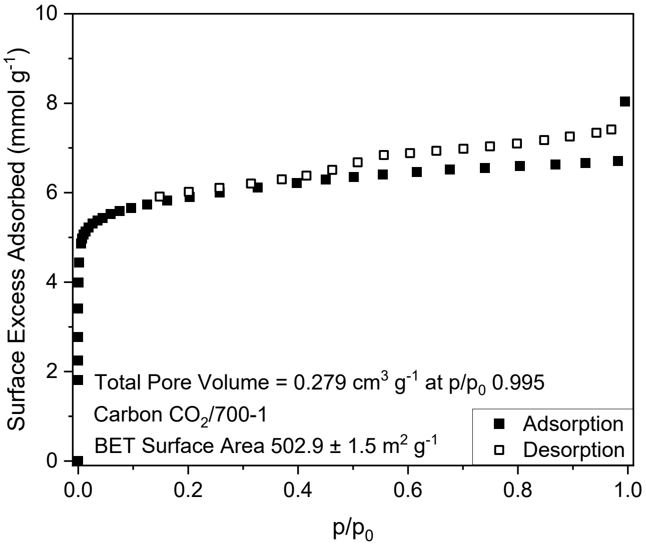

b)

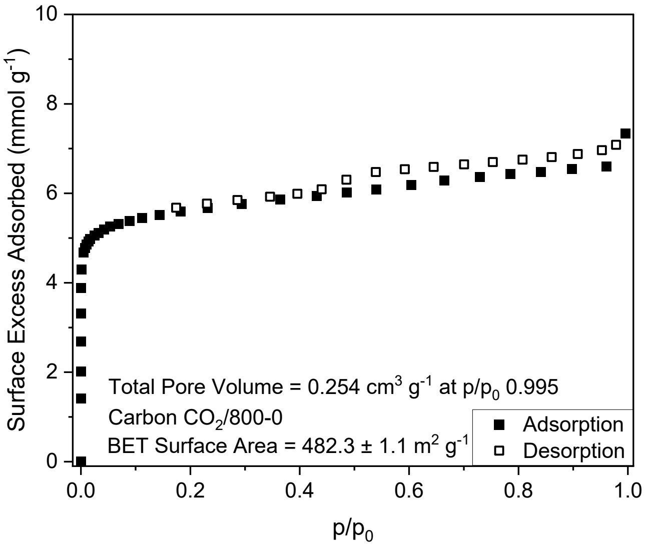

c)

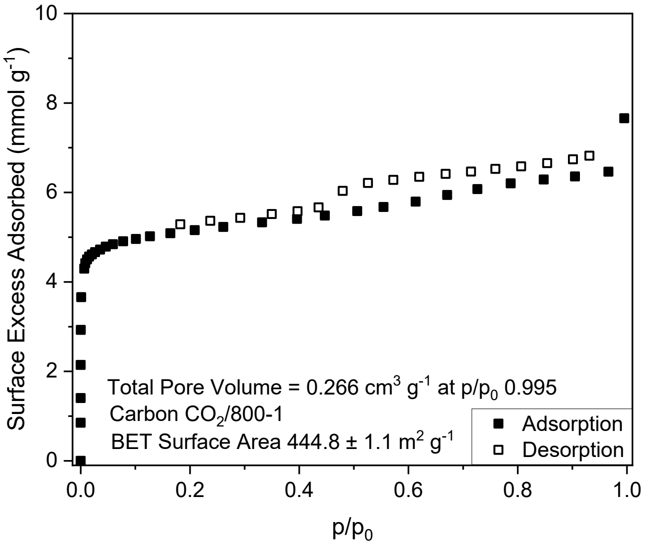
Surface Excess Adsorbed (mmol g^{-1})
p/p$_0$
Total Pore Volume = 0.266 cm^3 g^{-1} at p/p$_0$ 0.995
Carbon CO_2/800-1
BET Surface Area 444.8 ± 1.1 m^2 g^{-1}
Adsorption
Desorption
0
2
4
6
8
10
0.0
0.2
0.4
0.6
0.8
1.0

d)

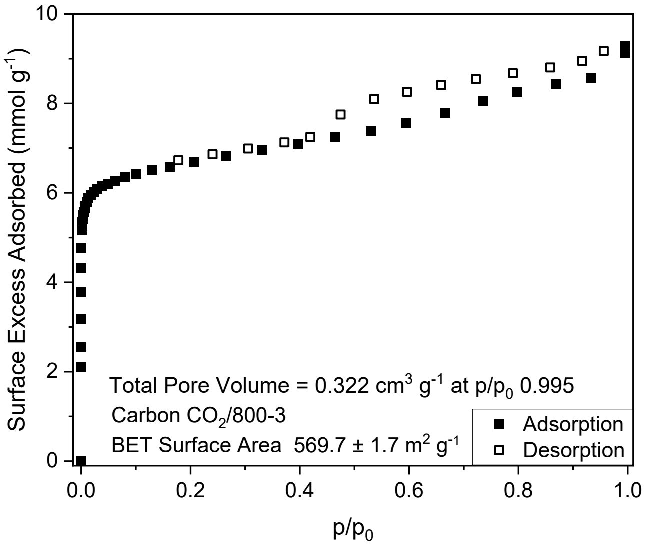
Surface Excess Adsorbed (mmol g^{-1})
p/p$_0$
Total Pore Volume = 0.322 cm^3 g^{-1} at p/p$_0$ 0.995
Carbon CO_2/800-3
BET Surface Area 569.7 ± 1.7 m^2 g^{-1}
Adsorption
Desorption
0
2
4
6
8
10
0.0
0.2
0.4
0.6
0.8
1.0

e)

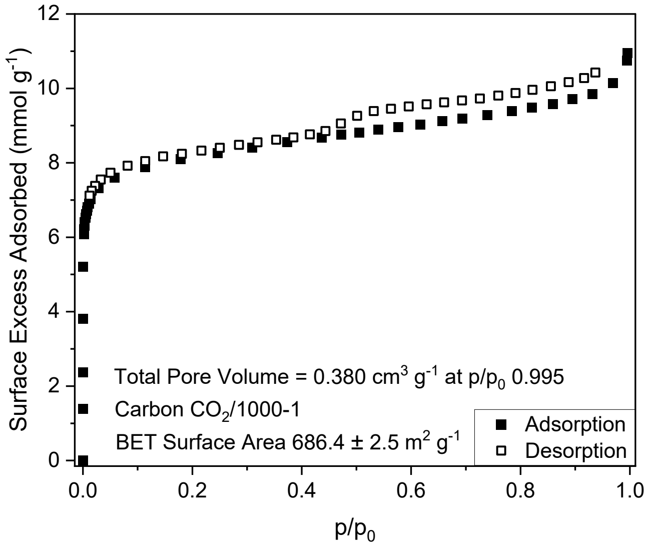

.

Figure S4. Nitrogen adsorption and desorption isotherms for carbons prepared in a carbon dioxide atmosphere at -196°C a) Carbon CO_2/700-1 b) Carbon CO_2/800-0 c) Carbon CO_2/800-1, d) Carbon CO_2/800-3 and e) Carbon CO_2/1000-1

1.2.2. BET graphs and Mesopore Pore Size Distributions for Nitrogen Adsorption and Desorption at -196°C for the Series of Carbons prepared in a Carbon Dioxide Atmosphere

a)

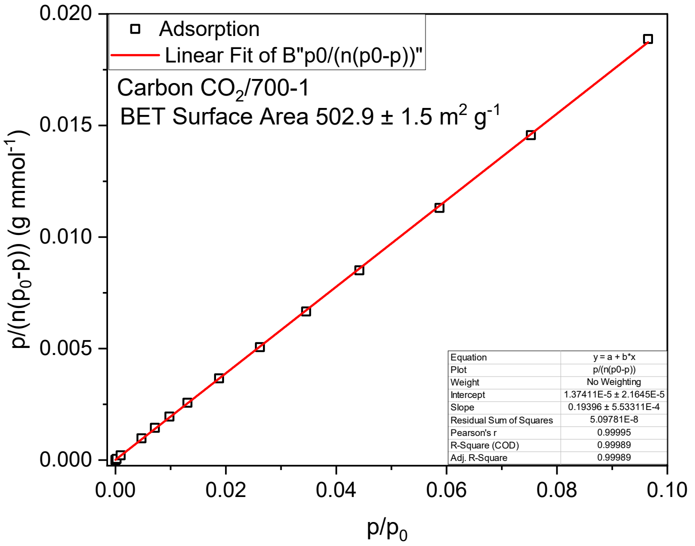

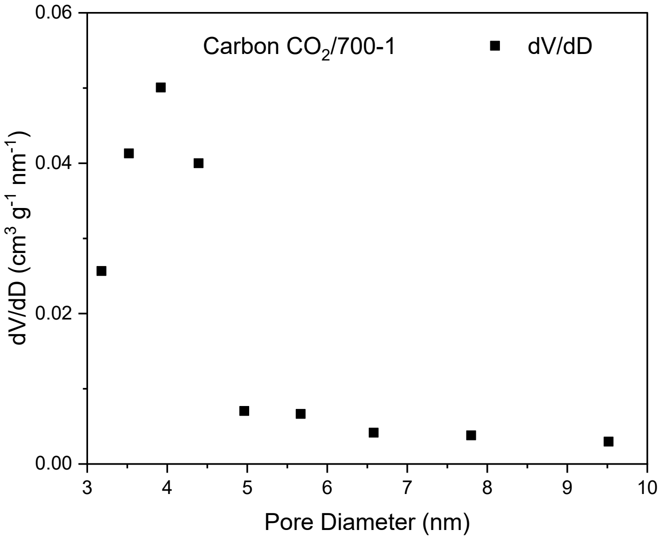
Carbon CO2/700-1
dV/dD
dV/dD (cm3 g-1 nm-1)
0.06
0.04
0.02
0.00
3
4
5
6
7
8
9
10
Pore Diameter (nm)

b)

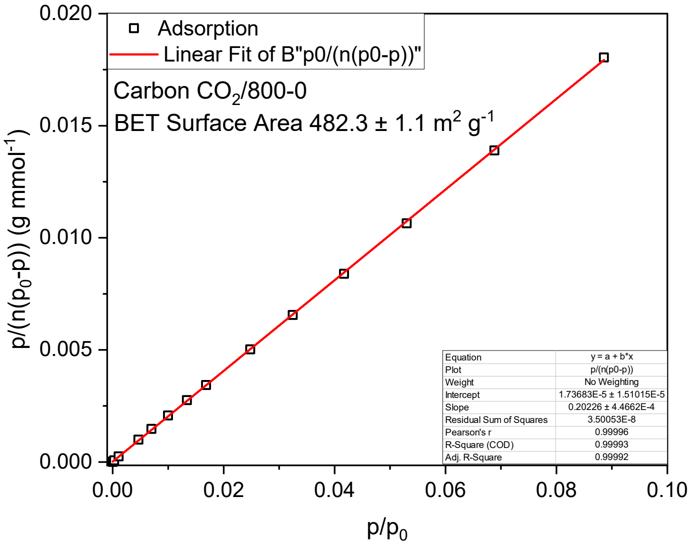
Adsorption
Linear Fit of B"p0/(n(p0-p))"
Carbon CO2/800-0
BET Surface Area 482.3 ± 1.1 m2 g-1
p/(n(p0-p)) (g mmol-1)
0.020
0.015
0.010
0.005
0.000
0.00
0.02
0.04
0.06
0.08
0.10
p/p0
Equation
y = a + b*x
Plot
p/(n(p0-p))
Weight
No Weighting
Intercept
1.73683E-5 ± 1.51015E-5
Slope
0.20226 ± 4.4662E-4
Residual Sum of Squares
3.50053E-8
Pearson's r
0.99996
R-Square (COD)
0.99993
Adj. R-Square
0.99992

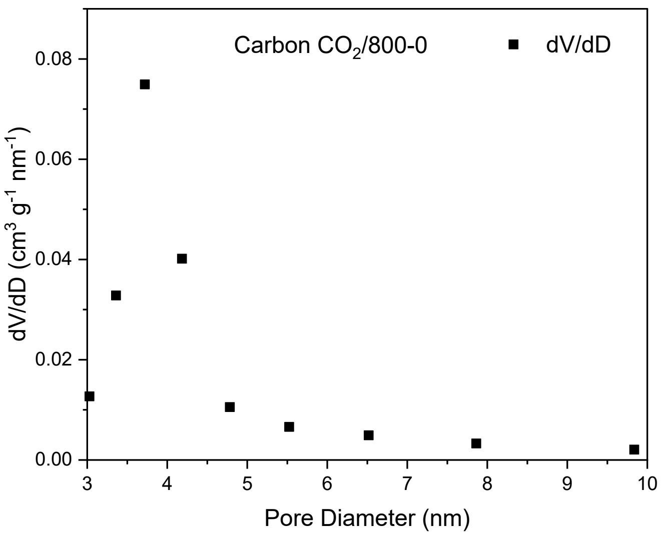

Carbon CO2/800-0
dV/dD
dV/dD (cm3 g-1 nm-1)
0.00
0.02
0.04
0.06
0.08
3
4
5
6
7
8
9
10
Pore Diameter (nm)

c)

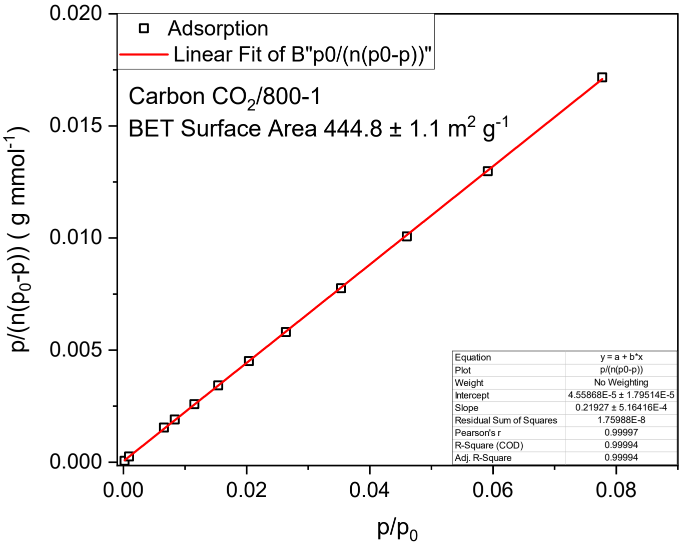

Adsorption
Linear Fit of B"p0/(n(p0-p))"
Carbon CO2/800-1
BET Surface Area 444.8 ± 1.1 m2 g-1
p/(n(p0-p)) (g mmol-1)
0.000
0.005
0.010
0.015
0.020
0.00
0.02
0.04
0.06
0.08
p/p0
Equation | y = a + b*x
Plot | p/(n(p0-p))
Weight | No Weighting
Intercept | 4.55868E-5 ± 1.79514E-5
Slope | 0.21927 ± 5.16416E-4
Residual Sum of Squares | 1.75988E-8
Pearson's r | 0.99997
R-Square (COD) | 0.99994
Adj. R-Square | 0.99994

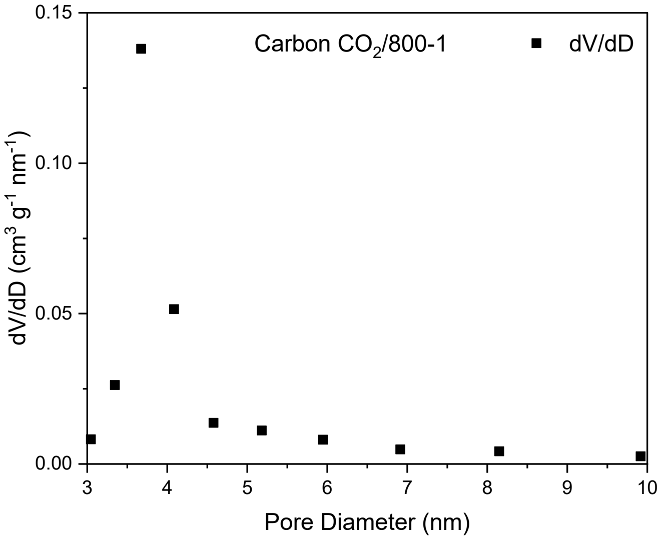
Carbon CO2/800-1
dV/dD
dV/dD (cm3 g-1 nm-1)
0.15
0.10
0.05
0.00
3
4
5
6
7
8
9
10
Pore Diameter (nm)

d)

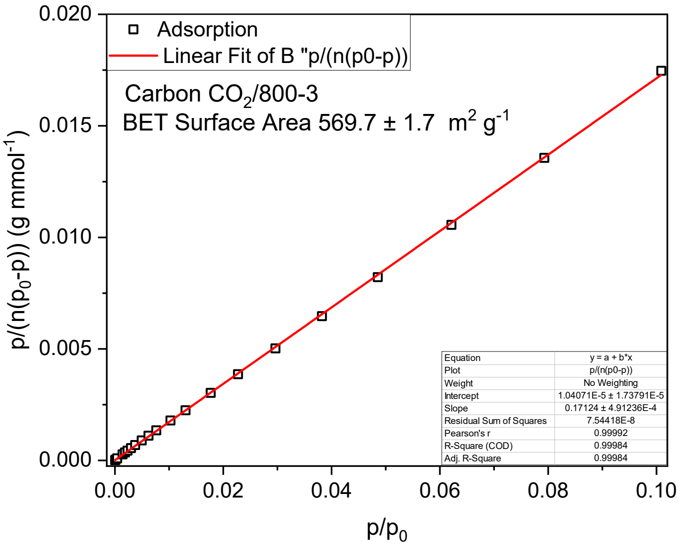
Adsorption
Linear Fit of B "p/(n(p0-p))
Carbon CO2/800-3
BET Surface Area 569.7 ± 1.7 m2 g-1
p/(n(p0-p)) (g mmol-1)
0.020
0.015
0.010
0.005
0.000
0.00
0.02
0.04
0.06
0.08
0.10
p/p0
Equation
y = a + b*x
Plot
p/(n(p0-p))
Weight
No Weighting
Intercept
1.04071E-5 ± 1.73791E-5
Slope
0.17124 ± 4.91236E-4
Residual Sum of Squares
7.54418E-8
Pearson's r
0.99992
R-Square (COD)
0.99984
Adj. R-Square
0.99984

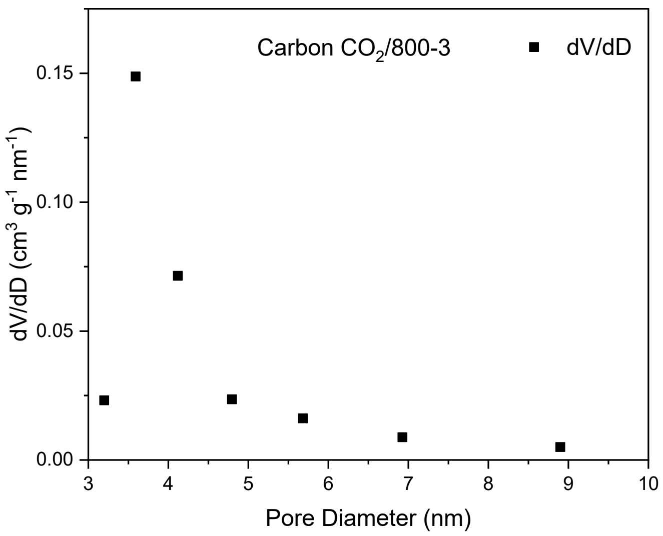
Carbon CO2/800-3
dV/dD
dV/dD (cm3 g-1 nm-1)
0.00
0.05
0.10
0.15
3
4
5
6
7
8
9
10
Pore Diameter (nm)

e)

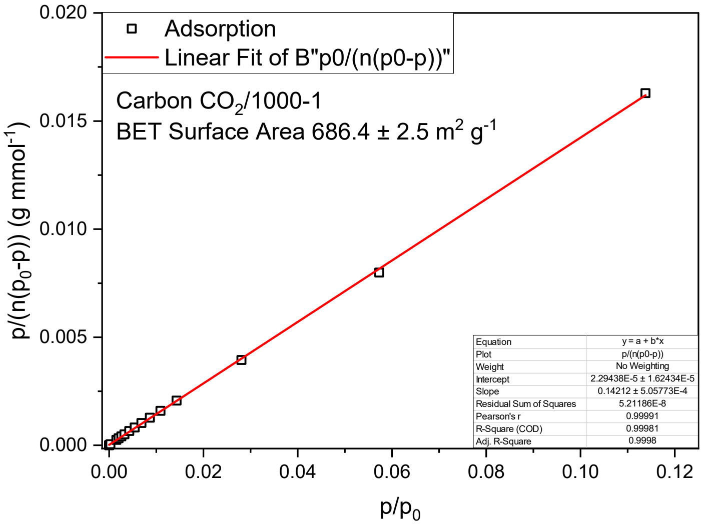
Adsorption
Linear Fit of B"p0/(n(p0-p))"
Carbon CO2/1000-1
BET Surface Area 686.4 ± 2.5 m2 g-1
p/(n(p0-p)) (g mmol-1)
0.000
0.005
0.010
0.015
0.020
0.00
0.02
0.04
0.06
0.08
0.10
0.12
p/p0
Equation | y = a + b*x
Plot | p/(n(p0-p))
Weight | No Weighting
Intercept | 2.29438E-5 ± 1.62434E-5
Slope | 0.14212 ± 5.05773E-4
Residual Sum of Squares | 5.21186E-8
Pearson's r | 0.99991
R-Square (COD) | 0.99981
Adj. R-Square | 0.9998

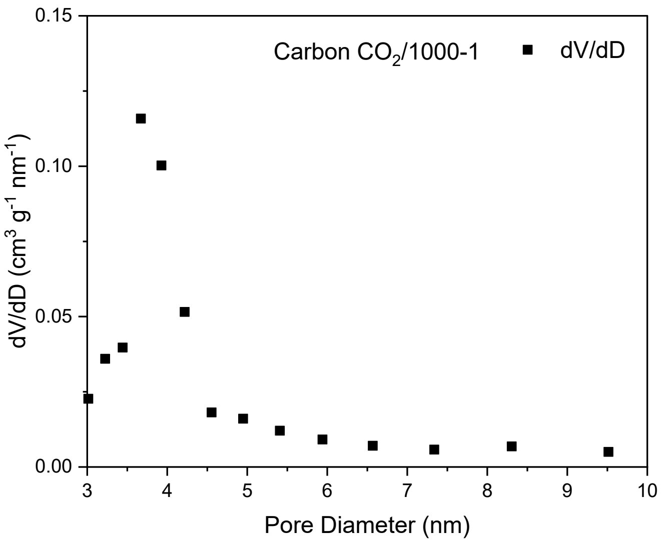

Figure S5. BET graphs and mesopore pore size distributions for nitrogen adsorption and desorption at -196°C for carbons prepared in a carbon dioxide atmosphere a) Carbon CO_2/700-1, b) Carbon CO_2/800-0 c) Carbon CO_2/800-1, d) Carbon CO_2/800-3 and e) Carbon CO_2/1000-1

1.2.3 Dubinin-Radushkevich graphs for nitrogen adsorption at -196°C for the Series of Carbons prepared in a Carbon Dioxide Atmosphere

a)

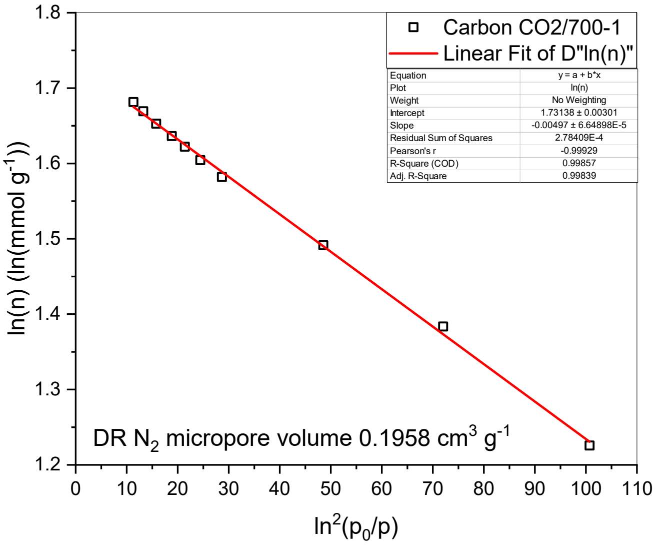

b)

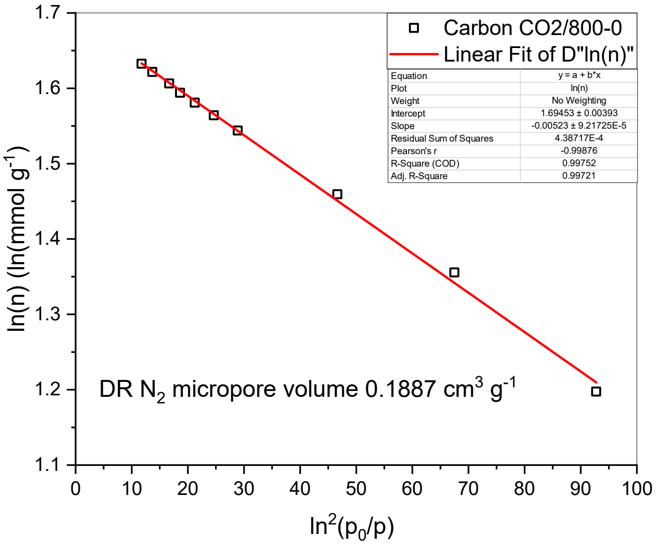

c)

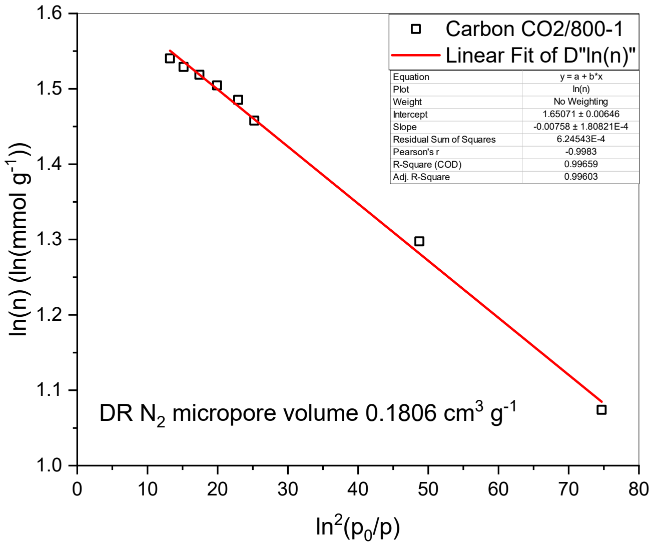

Carbon CO2/800-1
Linear Fit of D"ln(n)"
Equation y = a + b*x
Plot ln(n)
Weight No Weighting
Intercept 1.65071 ± 0.00646
Slope -0.00758 ± 1.80821E-4
Residual Sum of Squares 6.24543E-4
Pearson's r -0.9983
R-Square (COD) 0.99659
Adj. R-Square 0.99603
DR N_2 micropore volume 0.1806 $cm^3 g^{-1}$
ln(n) (ln(mmol g^{-1}))
$ln^2(p_0/p)$
1.0
1.1
1.2
1.3
1.4
1.5
1.6
0
10
20
30
40
50
60
70
80

d)

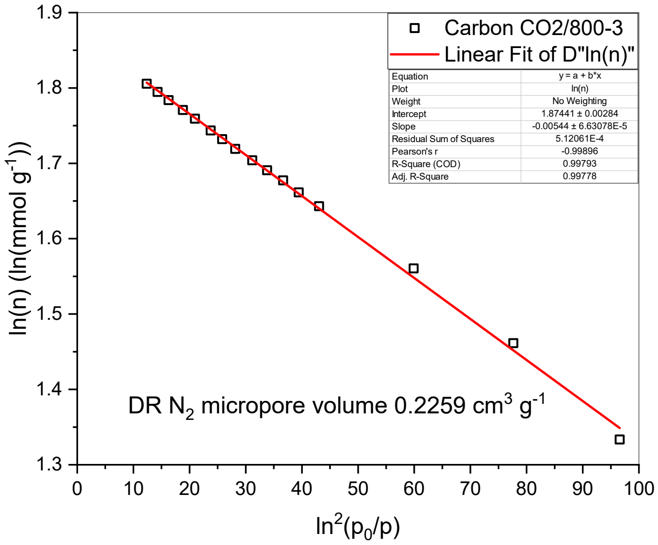

Carbon CO2/800-3
Linear Fit of D"ln(n)"
Equation y = a + b*x
Plot ln(n)
Weight No Weighting
Intercept 1.87441 ± 0.00284
Slope -0.00544 ± 6.63078E-5
Residual Sum of Squares 5.12061E-4
Pearson's r -0.99896
R-Square (COD) 0.99793
Adj. R-Square 0.99778
DR N_2 micropore volume 0.2259 $cm^3 g^{-1}$
ln(n) (ln(mmol g^{-1}))
$ln^2(p_0/p)$
1.3
1.4
1.5
1.6
1.7
1.8
1.9
0
10
20
30
40
50
60
70
80
90
100

e)

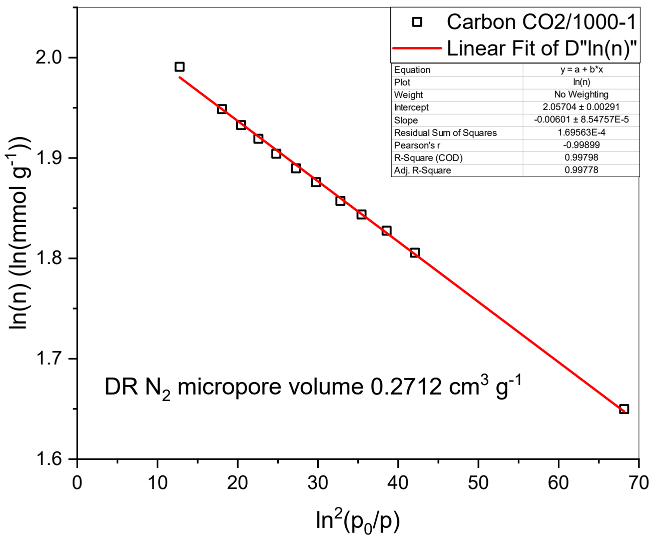

Figure S6. Dubinin-Radushkevich graphs for nitrogen adsorption at -196°C on carbons prepared in a carbon dioxide atmosphere a) Carbon CO_2/700-1, b) Carbon CO_2/800-0, c) Carbon CO_2/800-1, d) Carbon CO_2/800-3 and e) Carbon CO_2/1000-1

1.2.4 CO_2 Adsorption and Desorption Isotherms at 0°C for the Carbons prepared in a Carbon Dioxide Atmosphere

a)

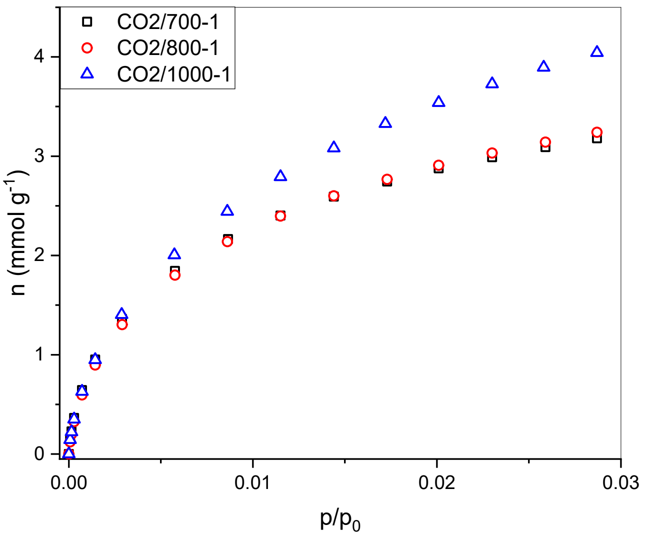

b)

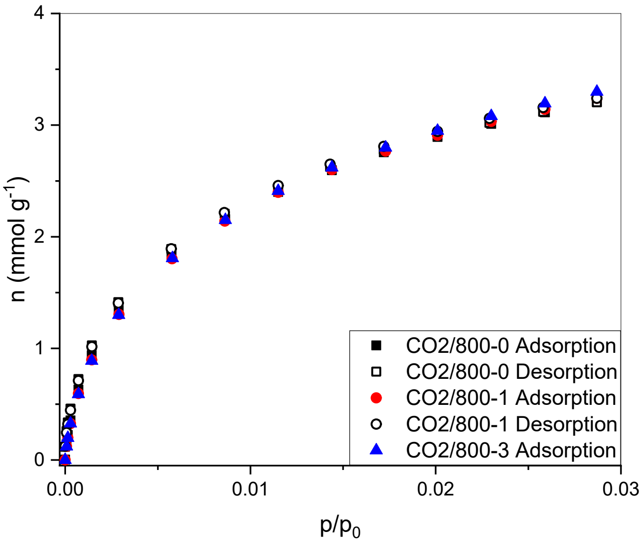

Figure S7. Carbon dioxide adsorption and desorption isotherms at 0°C for carbons prepared in a CO_2 atmosphere a) The effect of HTT and b) The effect of hold time at HTT 800°C.

1.2.5 Dubinin-Radushkevich graphs for Carbon Dioxide Adsorption at 0°C for the Series of Carbons prepared in a Carbon Dioxide Atmosphere

a)

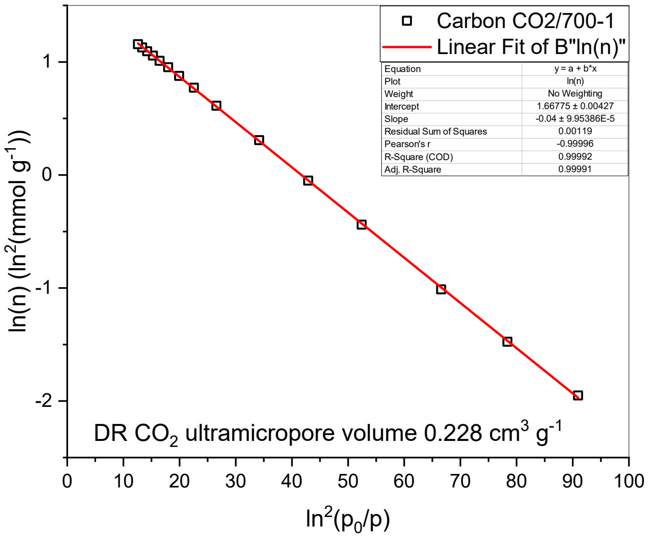

b)

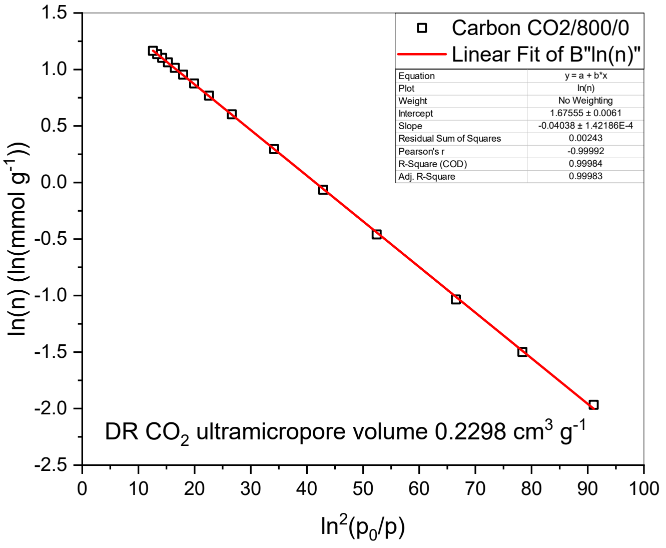
Carbon CO2/800/0
Linear Fit of B"ln(n)"
Equation | y = a + b*x
Plot | ln(n)
Weight | No Weighting
Intercept | 1.67555 ± 0.0061
Slope | -0.04038 ± 1.42186E-4
Residual Sum of Squares | 0.00243
Pearson's r | -0.99992
R-Square (COD) | 0.99984
Adj. R-Square | 0.99983
DR CO2 ultramicropore volume 0.2298 cm3 g-1
ln(n) (ln(mmol g-1))
ln2(p0/p)

c)

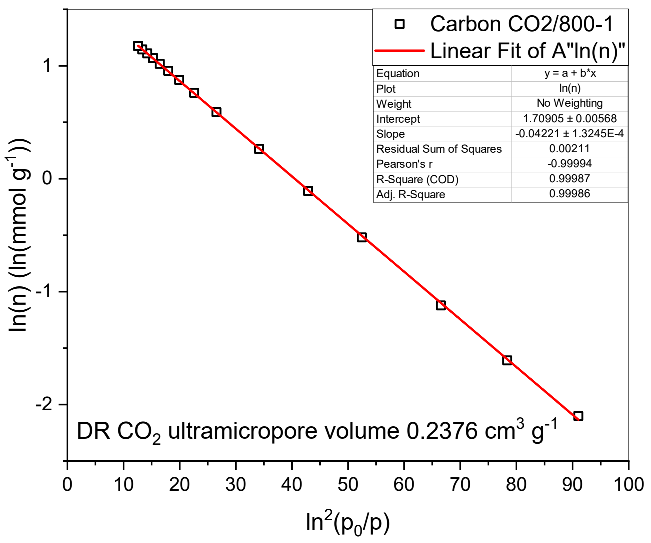
Carbon CO2/800-1
Linear Fit of A"ln(n)"
Equation | y = a + b*x
Plot | ln(n)
Weight | No Weighting
Intercept | 1.70905 ± 0.00568
Slope | -0.04221 ± 1.3245E-4
Residual Sum of Squares | 0.00211
Pearson's r | -0.99994
R-Square (COD) | 0.99987
Adj. R-Square | 0.99986
DR CO2 ultramicropore volume 0.2376 cm3 g-1
ln(n) (ln(mmol g-1))
ln2(p0/p)

d)

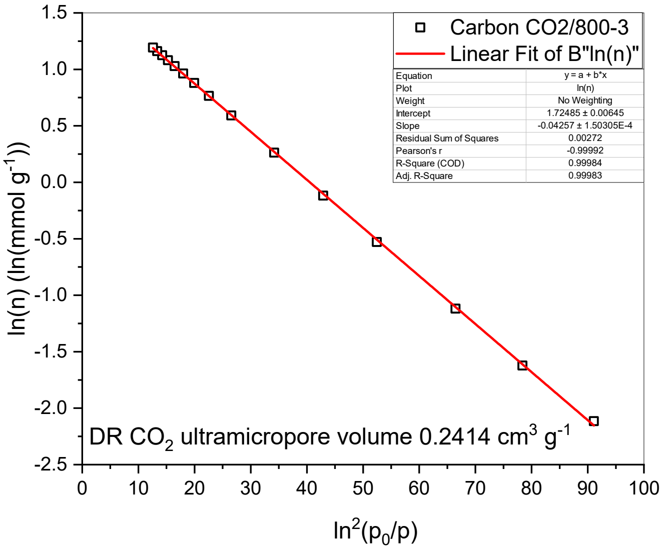

e)

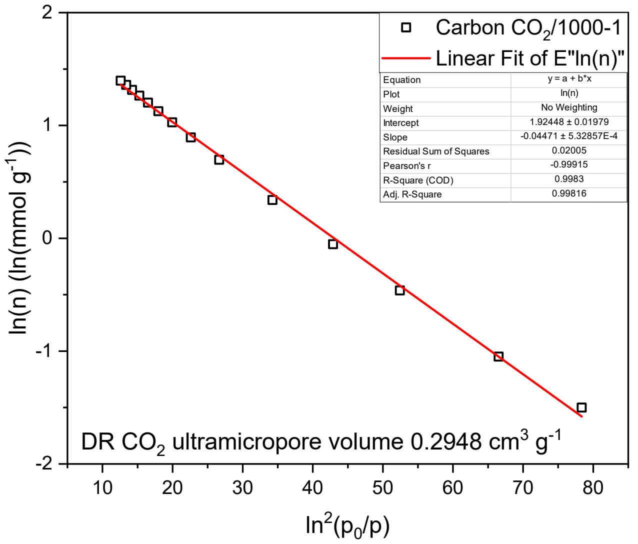

Figure S8. Dubinin-Radushkevich graphs for CO_2 adsorption at 0°C on carbons prepared in a carbon dioxide atmosphere a) Carbon CO_2/700-1, b) Carbon CO_2/800-0, c) Carbon CO_2/800-1, d) Carbon CO_2/800-3 and e) Carbon CO_2/1000-1

1.3 Chemical Treatment of Carbon for Functional Group Modification

1.3.1 Nitrogen Adsorption at -196°C for Functionalized Carbons

a)

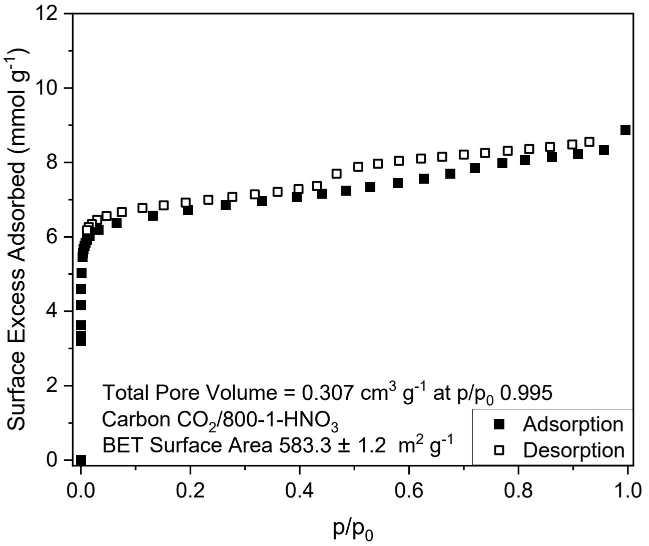

b)

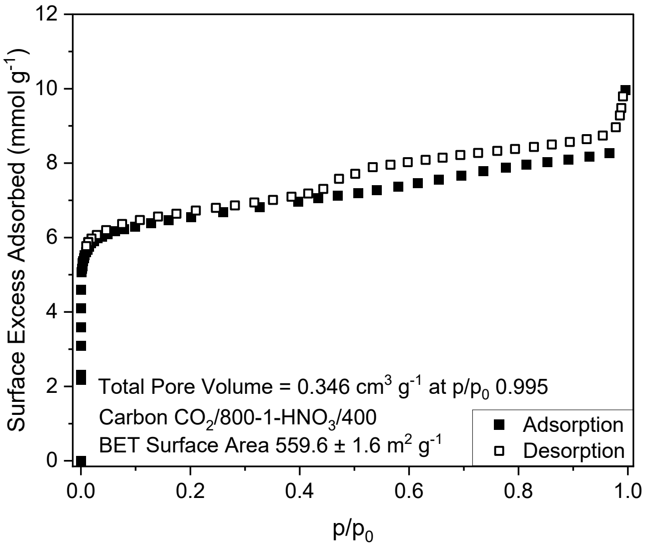

c)

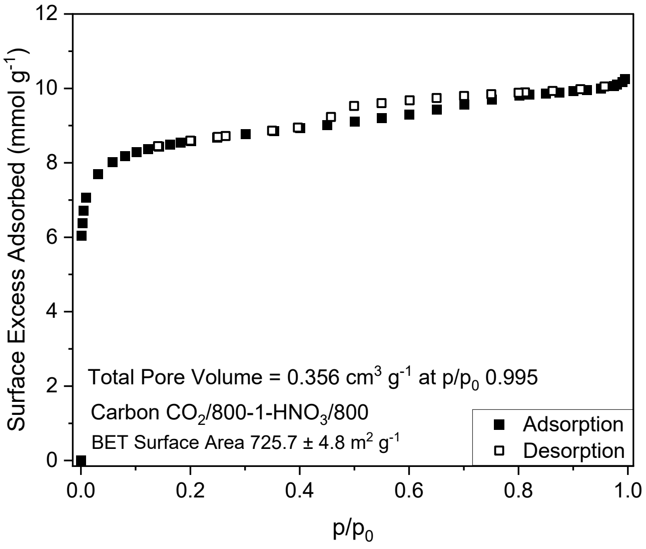

Surface Excess Adsorbed (mmol g-1)
p/p0
Total Pore Volume = 0.356 cm3 g-1 at p/p0 0.995
Carbon CO2/800-1-HNO3/800
BET Surface Area 725.7 ± 4.8 m2 g-1
Adsorption
Desorption

d)

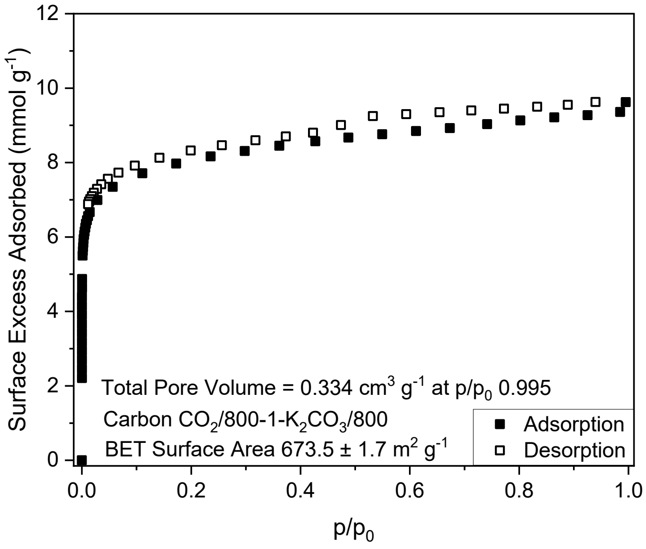

Surface Excess Adsorbed (mmol g-1)
p/p0
Total Pore Volume = 0.334 cm3 g-1 at p/p0 0.995
Carbon CO2/800-1-K2CO3/800
BET Surface Area 673.5 ± 1.7 m2 g-1
Adsorption
Desorption

e)

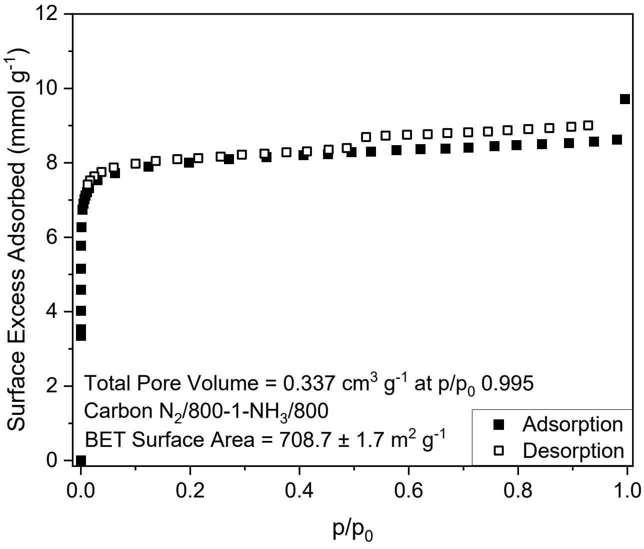

Figure Nitrogen adsorption and desorption isotherms at -196°C for functionalized carbons a) Carbon CO_2/800-1-HNO_3 b) Carbon CO_2/800-1-HNO_3/400 c) Carbon CO_2/800-1-HNO_3/800, d) Carbon CO_2/800-1-K_2CO_3/800 and e) Carbon N_2/800-1-NH_3/800

1.3.2. BET graphs and Mesopore Pore Size Distributions for Nitrogen Adsorption at -196°C on Functionalized Carbons

a)

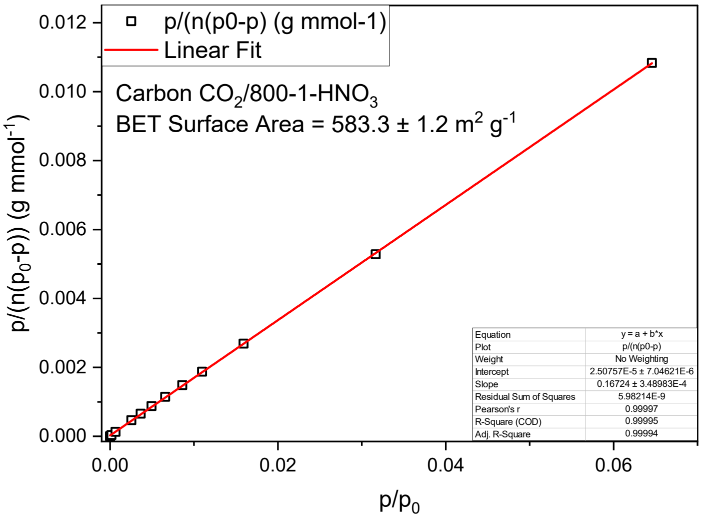

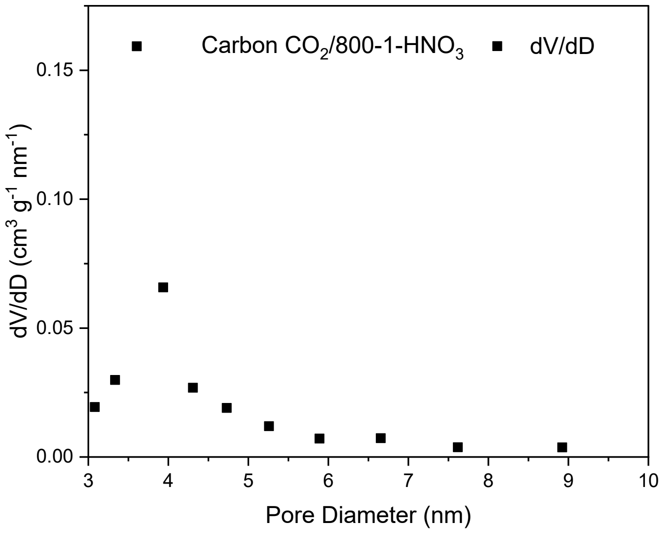

Carbon CO2/800-1-HNO3
dV/dD
dV/dD (cm3 g-1 nm-1)
0.15
0.10
0.05
0.00
3
4
5
6
7
8
9
10
Pore Diameter (nm)

b)

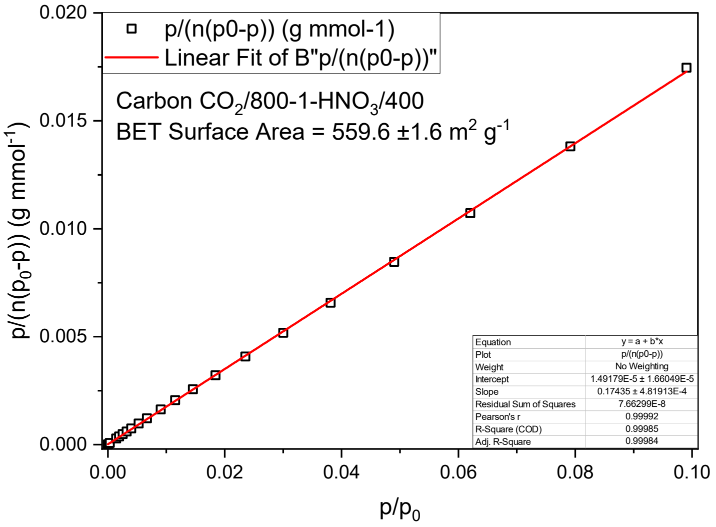

p/(n(p0-p)) (g mmol-1)
Linear Fit of B"p/(n(p0-p))"
Carbon CO2/800-1-HNO3/400
BET Surface Area = 559.6 ±1.6 m2 g-1
p/(n(p0-p)) (g mmol-1)
0.020
0.015
0.010
0.005
0.000
0.00
0.02
0.04
0.06
0.08
0.10
p/p0
Equation
y = a + b*x
Plot
p/(n(p0-p))
Weight
No Weighting
Intercept
1.49179E-5 ± 1.66049E-5
Slope
0.17435 ± 4.81913E-4
Residual Sum of Squares
7.66299E-8
Pearson's r
0.99992
R-Square (COD)
0.99985
Adj. R-Square
0.99984

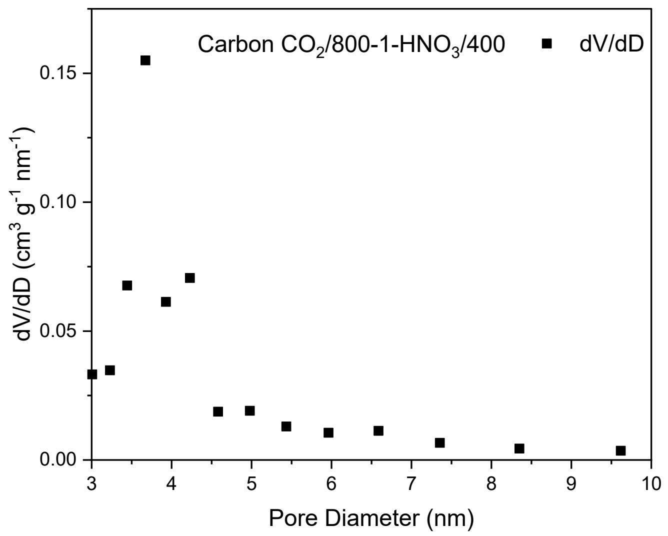
Carbon CO2/800-1-HNO3/400
dV/dD
dV/dD (cm3 g-1 nm-1)
0.00
0.05
0.10
0.15
3
4
5
6
7
8
9
10
Pore Diameter (nm)

c)

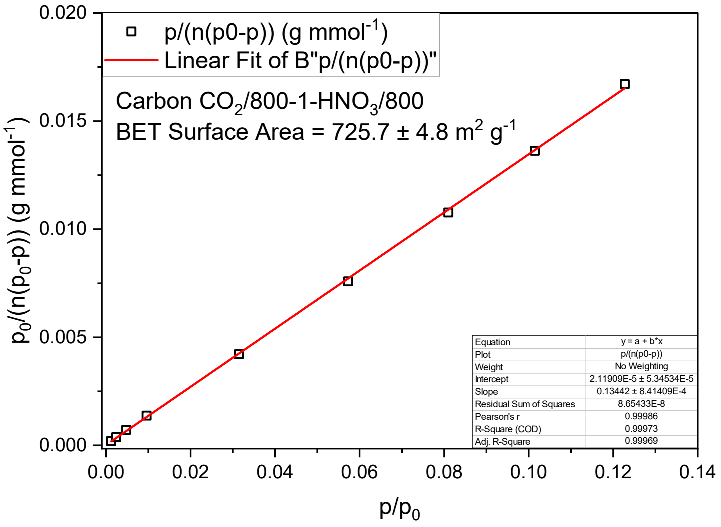
p/(n(p0-p)) (g mmol-1)
Linear Fit of B"p/(n(p0-p))"
Carbon CO2/800-1-HNO3/800
BET Surface Area = 725.7 ± 4.8 m2 g-1
p0/(n(p0-p)) (g mmol-1)
0.000
0.005
0.010
0.015
0.020
0.00
0.02
0.04
0.06
0.08
0.10
0.12
0.14
p/p0
Equation y = a + b*x
Plot p/(n(p0-p))
Weight No Weighting
Intercept 2.11909E-5 ± 5.34534E-5
Slope 0.13442 ± 8.41409E-4
Residual Sum of Squares 8.65433E-8
Pearson's r 0.99986
R-Square (COD) 0.99973
Adj. R-Square 0.99969

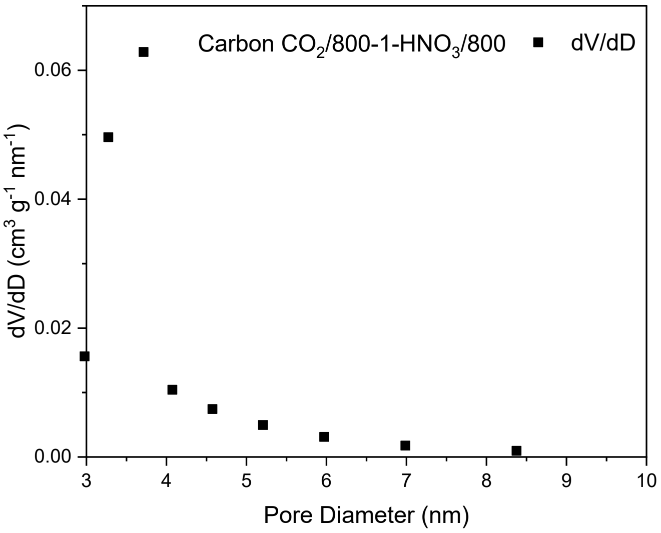
Carbon CO2/800-1-HNO3/800
dV/dD
dV/dD (cm3 g-1 nm-1)
0.00
0.02
0.04
0.06
3
4
5
6
7
8
9
10
Pore Diameter (nm)

d)

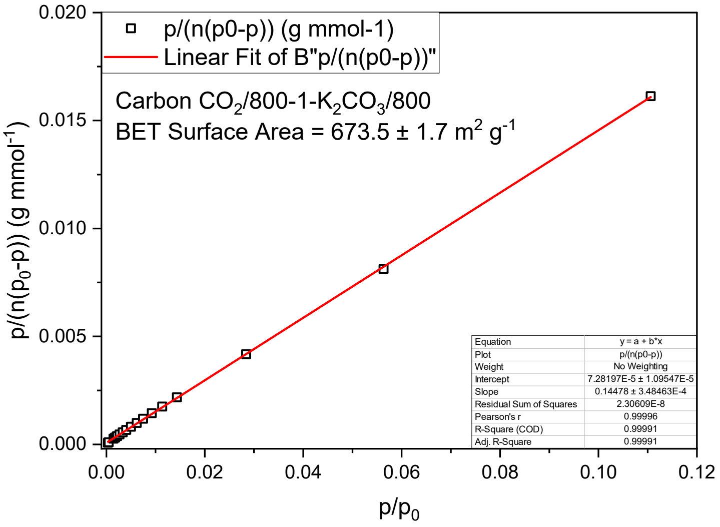
p/(n(p0-p)) (g mmol-1)
Linear Fit of B"p/(n(p0-p))"
Carbon CO2/800-1-K2CO3/800
BET Surface Area = 673.5 ± 1.7 m2 g-1
p/(n(p0-p)) (g mmol-1)
0.000
0.005
0.010
0.015
0.020
0.00
0.02
0.04
0.06
0.08
0.10
0.12
p/p0
Equation
y = a + b*x
Plot
p/(n(p0-p))
Weight
No Weighting
Intercept
7.28197E-5 ± 1.09547E-5
Slope
0.14478 ± 3.48463E-4
Residual Sum of Squares
2.30609E-8
Pearson's r
0.99996
R-Square (COD)
0.99991
Adj. R-Square
0.99991

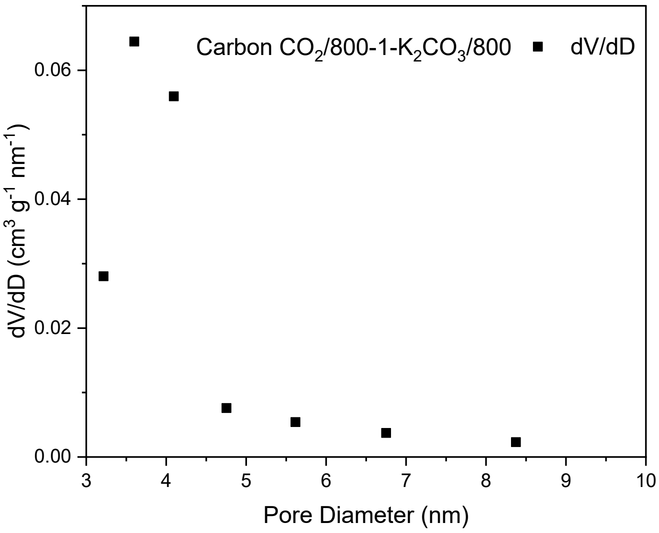

Carbon CO_2/800-1-K_2CO_3/800
dV/dD
dV/dD (cm^3 g^{-1} nm^{-1})
0.06
0.04
0.02
0.00
3
4
5
6
7
8
9
10
Pore Diameter (nm)

e)

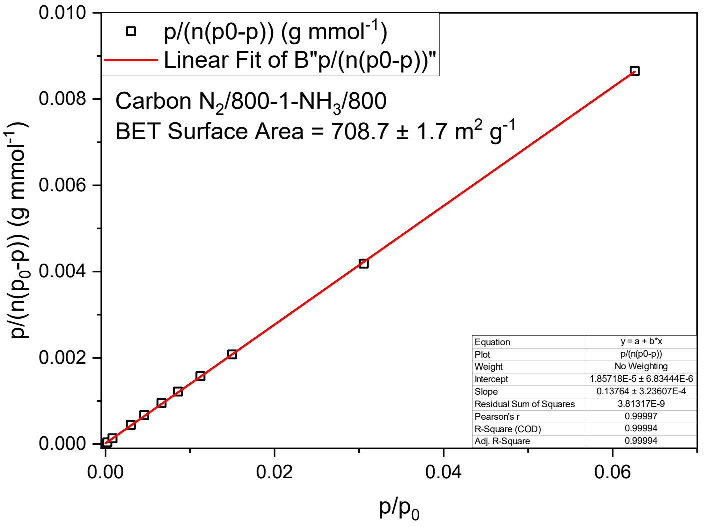

p/(n(p0-p)) (g $mmol^{-1}$)
Linear Fit of B"p/(n(p0-p))"
Carbon N_2/800-1-NH_3/800
BET Surface Area = 708.7 ± 1.7 m^2 g^{-1}
p/(n(p_0-p)) (g $mmol^{-1}$)
0.010
0.008
0.006
0.004
0.002
0.000
0.00
0.02
0.04
0.06
p/p_0
Equation y = a + b*x
Plot p/(n(p0-p))
Weight No Weighting
Intercept 1.85718E-5 ± 6.83444E-6
Slope 0.13764 ± 3.23607E-4
Residual Sum of Squares 3.81317E-9
Pearson's r 0.99997
R-Square (COD) 0.99994
Adj. R-Square 0.99994

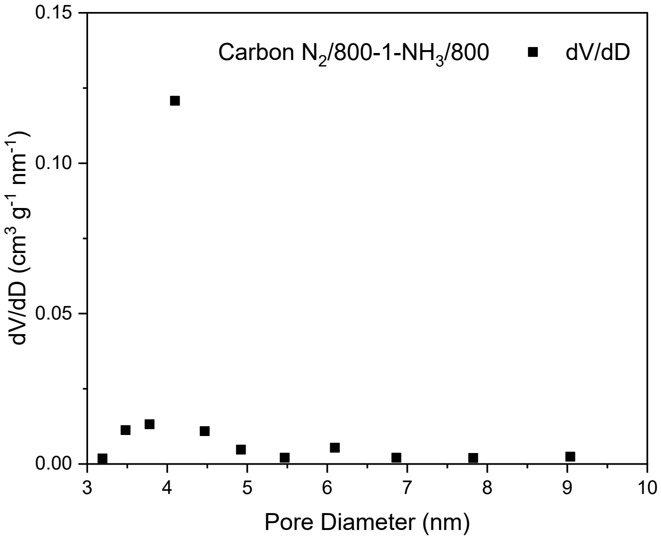

Figure S10. BET and mesopore pore size distribution graphs for nitrogen adsorption isotherms at -196°C for functionalized carbons a) Carbon CO_2/800-1-HNO_3 b) Carbon CO_2/800-1-HNO_3/400 c) Carbon CO_2/800-1-HNO_3/800, d) Carbon CO_2/800-1-K_2CO_3/800 and e) Carbon N_2/800-1-NH_3/800

1.3.3. Dubinin-Radushkevich graphs for Nitrogen Adsorption at -196°C on Functionalized Carbons

a)

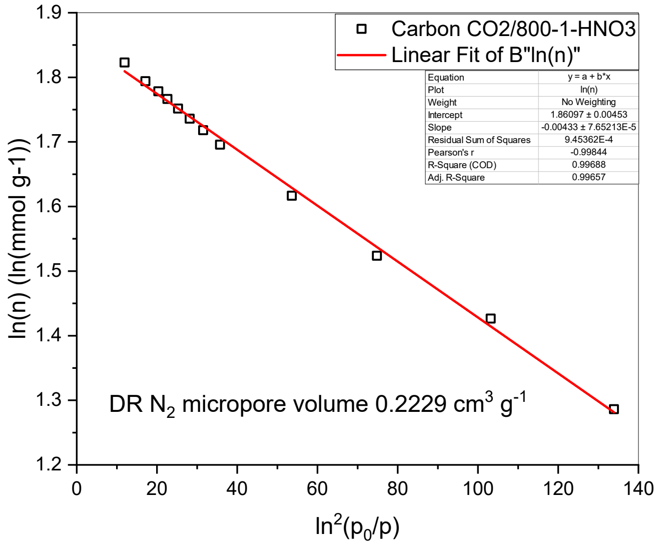

b)

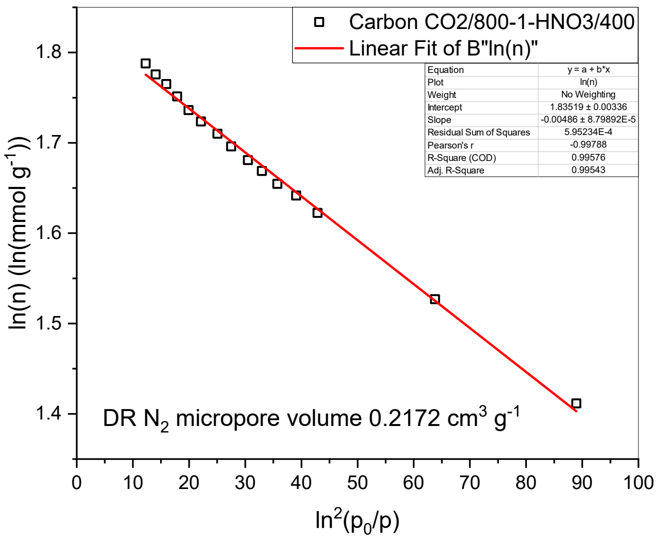

c)

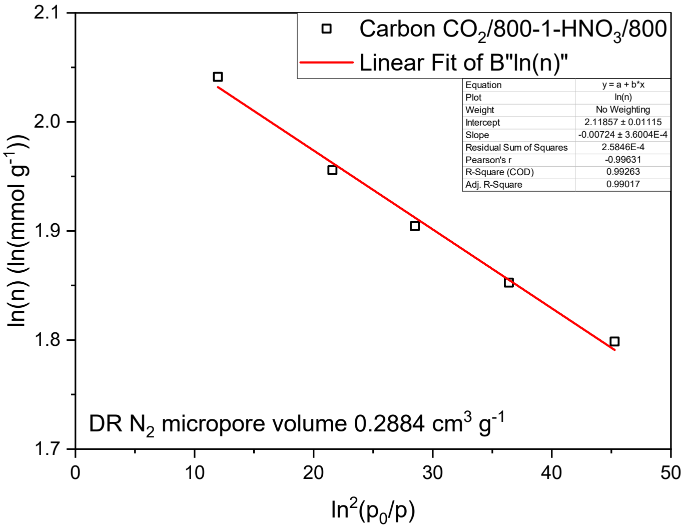
Carbon CO2/800-1-HNO3/800
Linear Fit of B"ln(n)"
Equation | y = a + b*x
Plot | ln(n)
Weight | No Weighting
Intercept | 2.11857 ± 0.01115
Slope | -0.00724 ± 3.6004E-4
Residual Sum of Squares | 2.5846E-4
Pearson's r | -0.99631
R-Square (COD) | 0.99263
Adj. R-Square | 0.99017
DR N2 micropore volume 0.2884 cm3 g-1
ln(n) (ln(mmol g-1))
ln2(p0/p)
2.1
2.0
1.9
1.8
1.7
0
10
20
30
40
50

d)

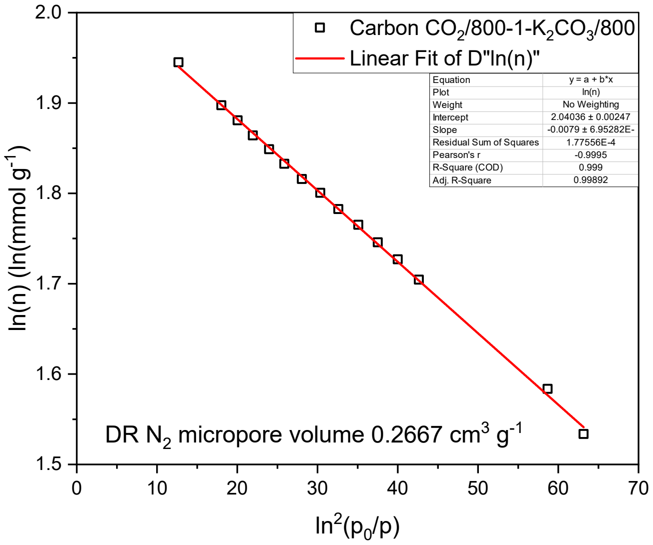
Carbon CO2/800-1-K2CO3/800
Linear Fit of D"ln(n)"
Equation | y = a + b*x
Plot | ln(n)
Weight | No Weighting
Intercept | 2.04036 ± 0.00247
Slope | -0.0079 ± 6.95282E-
Residual Sum of Squares | 1.77556E-4
Pearson's r | -0.9995
R-Square (COD) | 0.999
Adj. R-Square | 0.99892
DR N2 micropore volume 0.2667 cm3 g-1
ln(n) (ln(mmol g-1))
ln2(p0/p)
2.0
1.9
1.8
1.7
1.6
1.5
0
10
20
30
40
50
60
70

e)

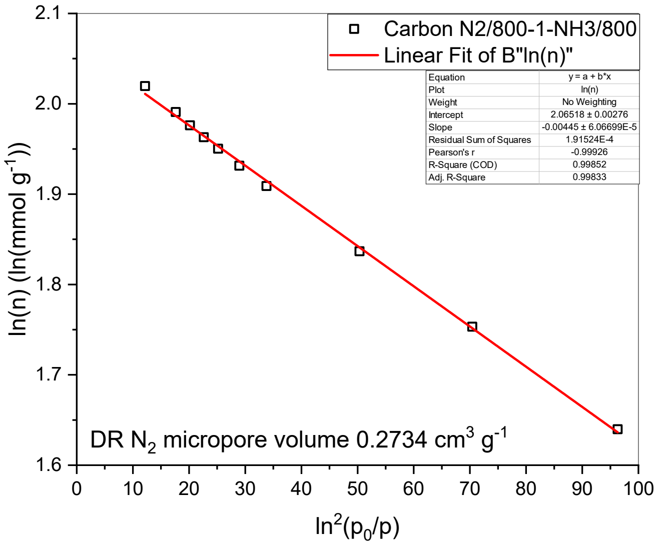

Figure S11. Dubinin-Radushkevich graphs for nitrogen adsorption at -196°C for functionalized carbons a) Carbon CO_2/800-1-HNO_3 b) Carbon CO_2/800-1-HNO_3/400 c) Carbon CO_2/800-1-HNO_3/800, d) Carbon CO_2/800-1-K_2CO_3/800 and e) Carbon N_2/800-1-NH_3/800

1.3.4 Carbon Dioxide Adsorption Isotherms at 0°C for Functionalized Carbons

a)

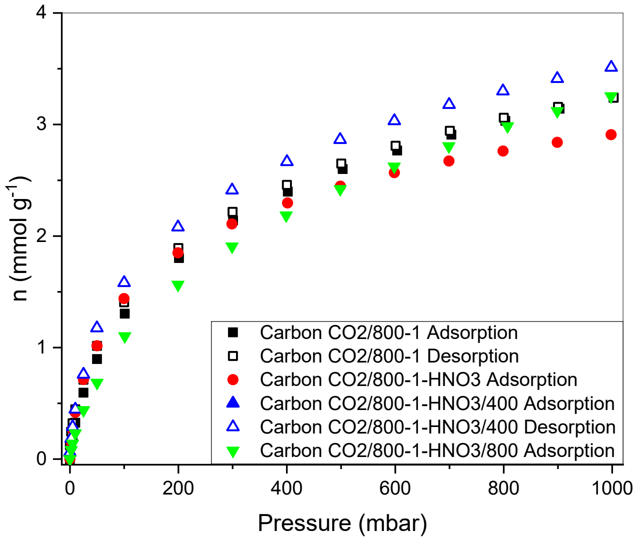

b)

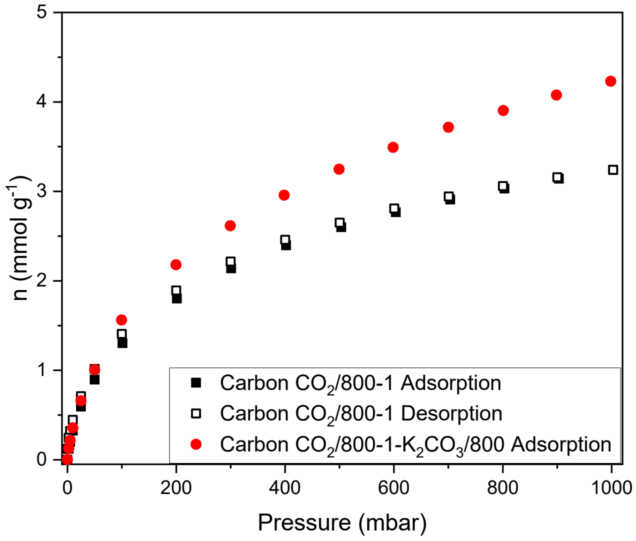

c)

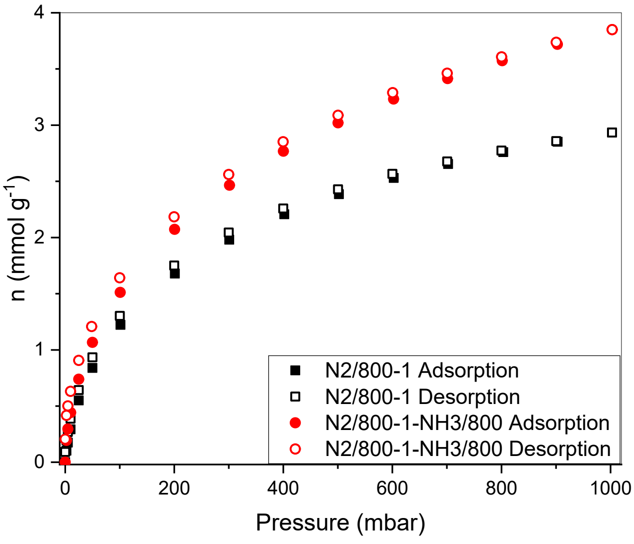

Figure S12. Carbon dioxide adsorption isotherms for functionalized carbons at 0°C a) Nitric acid oxidized carbon CO_2/800-1 and heat-treated carbon series, b) Comparison of Carbons CO_2/800-1 and CO_2/800-1-K_2CO_3/800 and c) Comparison of Carbons N_2/800-1 and N_2/800-1-NH_3/800

1.3.5 Dubinin-Radushkevich graphs for Carbon Dioxide Adsorption at 0°C for Functionalized Carbons

a)

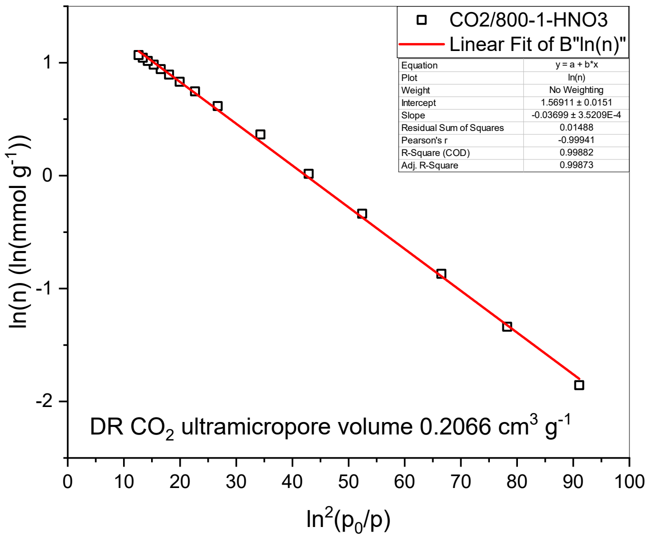

b)

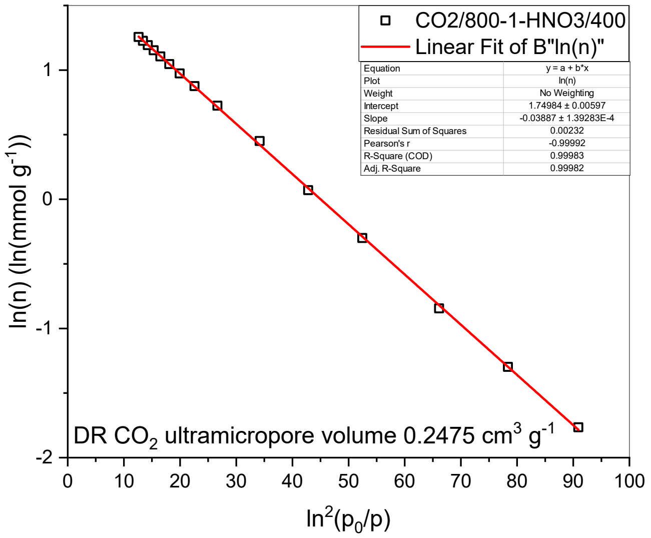

c)

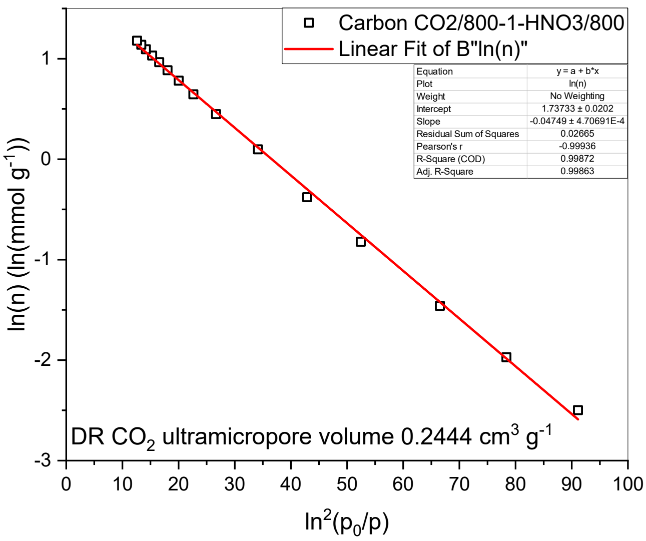
Carbon CO2/800-1-HNO3/800
Linear Fit of B"ln(n)"
Equation y = a + b*x
Plot ln(n)
Weight No Weighting
Intercept 1.73733 ± 0.0202
Slope -0.04749 ± 4.70691E-4
Residual Sum of Squares 0.02665
Pearson's r -0.99936
R-Square (COD) 0.99872
Adj. R-Square 0.99863
DR CO_2 ultramicropore volume 0.2444 cm^3 g^{-1}
ln(n) (ln(mmol g^{-1}))
$ln^2(p_0/p)$

d)

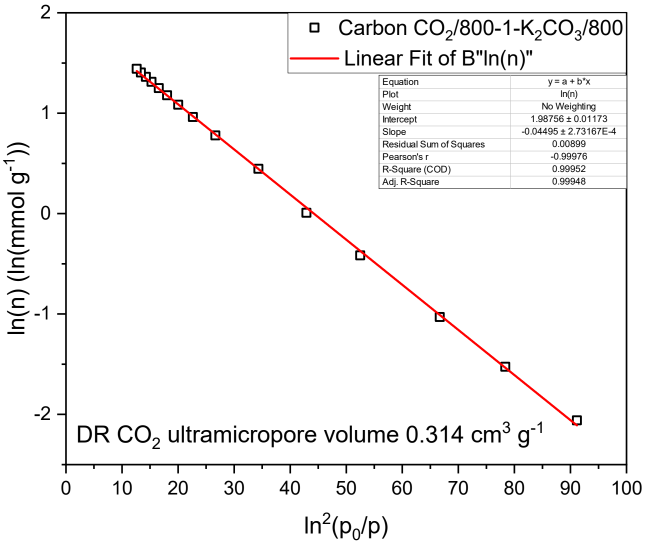
Carbon CO_2/800-1-K_2CO_3/800
Linear Fit of B"ln(n)"
Equation y = a + b*x
Plot ln(n)
Weight No Weighting
Intercept 1.98756 ± 0.01173
Slope -0.04495 ± 2.73167E-4
Residual Sum of Squares 0.00899
Pearson's r -0.99976
R-Square (COD) 0.99952
Adj. R-Square 0.99948
DR CO_2 ultramicropore volume 0.314 cm^3 g^{-1}
ln(n) (ln(mmol g^{-1}))
$ln^2(p_0/p)$

e)

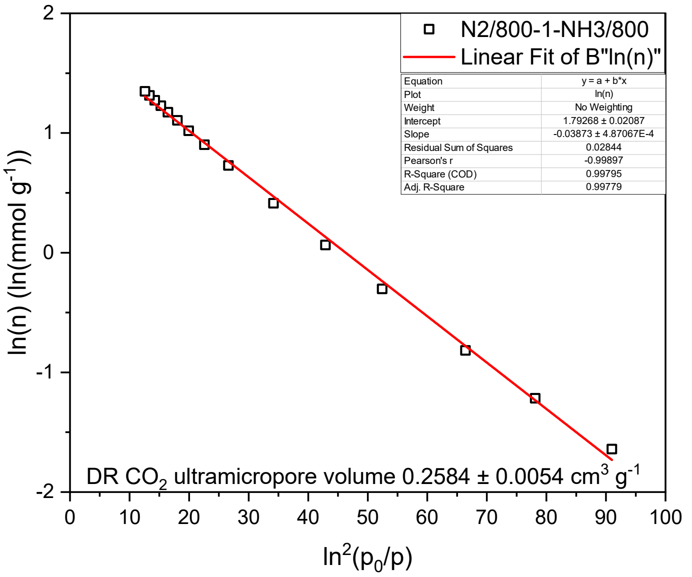

f)

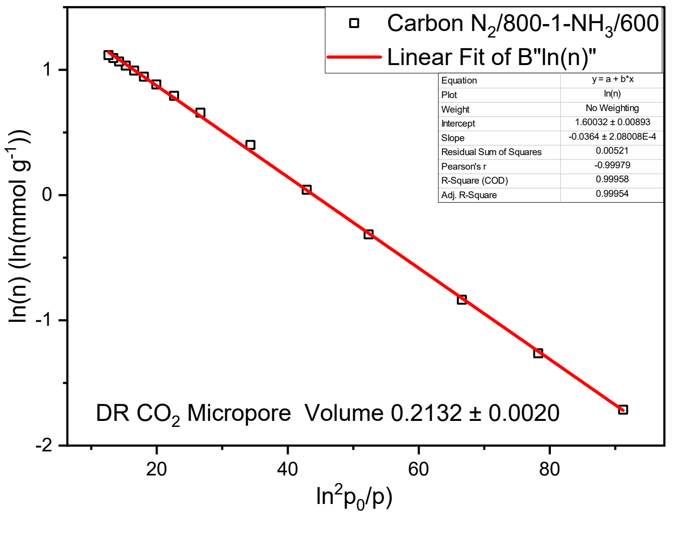

Figure S13 Dubinin-Radushkevich graphs for carbon dioxide adsorption at 0°C for functionalized carbons a) Carbon CO_2/800-1-HNO_3 b) Carbon CO_2/800-1-HNO_3/400 c) Carbon CO_2/800-1-HNO_3/800, d) Carbon CO_2/800-1-K_2CO_3/800 and e) Carbon N_2/800-1-NH_3/800 and f) Carbon N_2/800-1-NH_3/600

1.4 Comparison of Gas Adsorption Parameters

a)

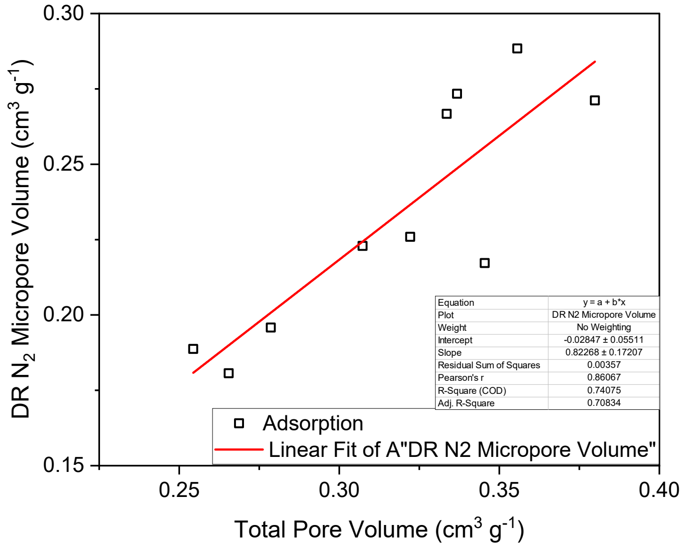

b)

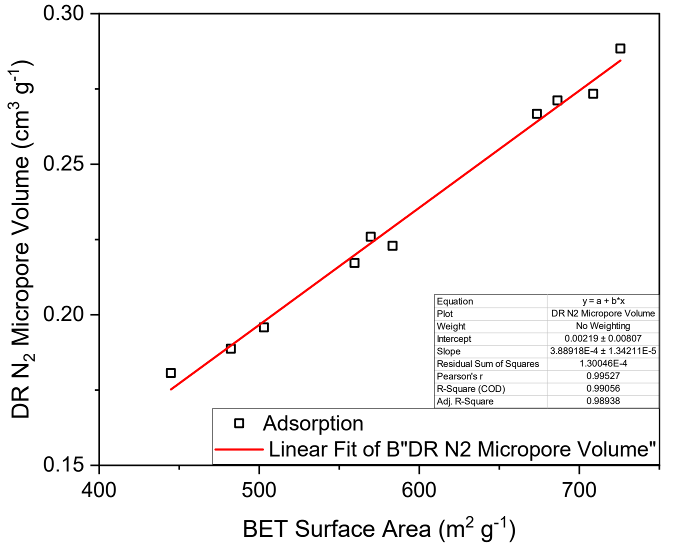

c)

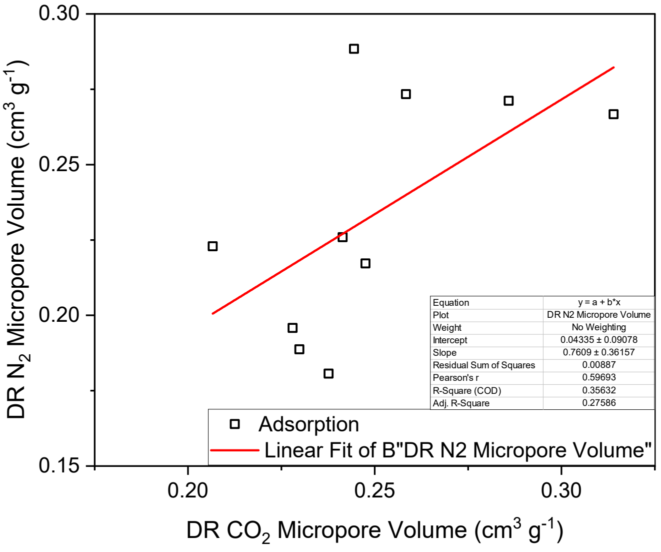

Figure S14 Comparison of adsorption parameters a) DR N_2 Micropore Volume versus Total Pore Volume; b) DR N_2 Micropore Volume versus BET Surface Area and c) DR N_2 Micropore Volume versus DR CO_2 Micropore Volume

2 Electrochemistry

2.1 Cyclic Voltammetry

2.1.1 Comparison of Cyclic Voltammograms with a Sweep Rate of 20mV s^{-1} for Carbon/PVDF Electrodes made from Carbons prepared in Nitrogen and Carbon Dioxide Atmospheres

a)

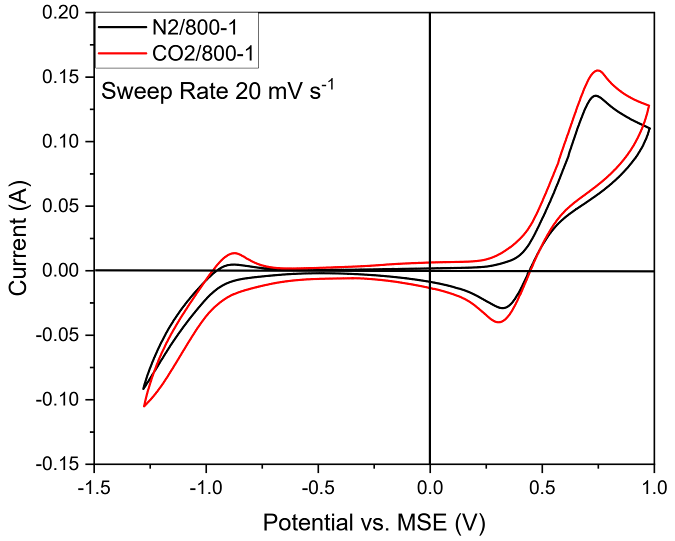

b)

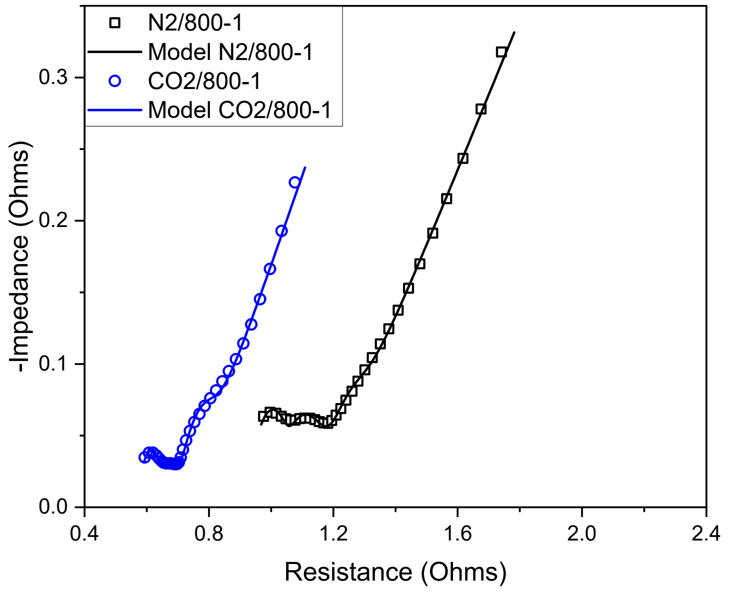

c)

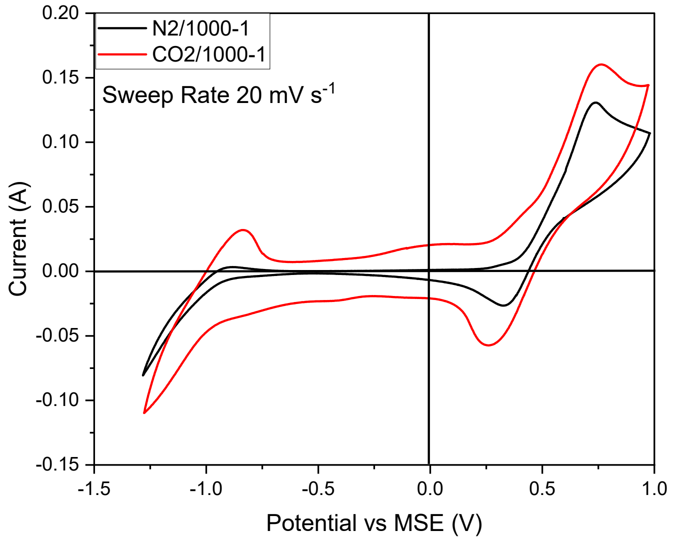

d)

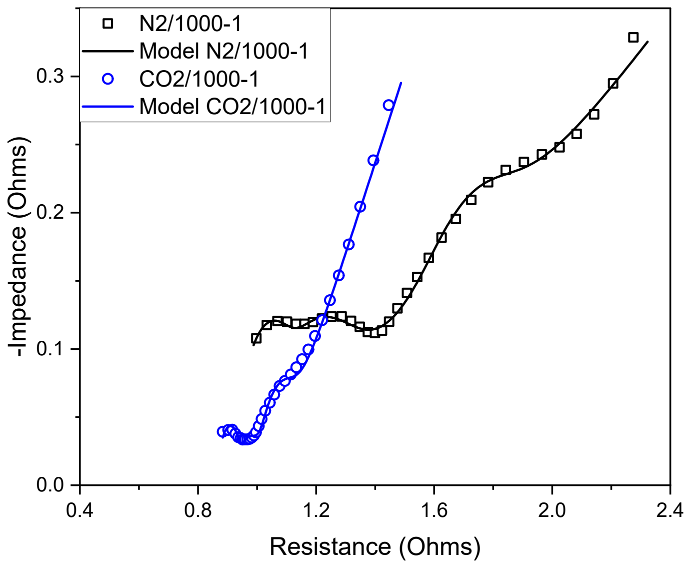

Figure S15 Comparison of cyclic voltammograms (CVs) and Nyquist graphs for vanadium redox reactions for electrodes fabricated from PVDF and carbons prepared under both nitrogen and carbon dioxide atmospheres at various temperatures a) CVs for electrodes with Carbons (HTT 800°C), b) Nyquist graphs for electrodes with Carbons (HTT 800°C), c) CVs for electrodes with Carbons (HTT 1000°C) and d) Nyquist graphs for electrodes with Carbons (HTT 1000°C)

2.1.2 Comparison of Cyclic Voltammograms for Carbon Electrodes as a Function of Sweep Rates for Carbons with a Range of Heat Treatment Temperatures

a)

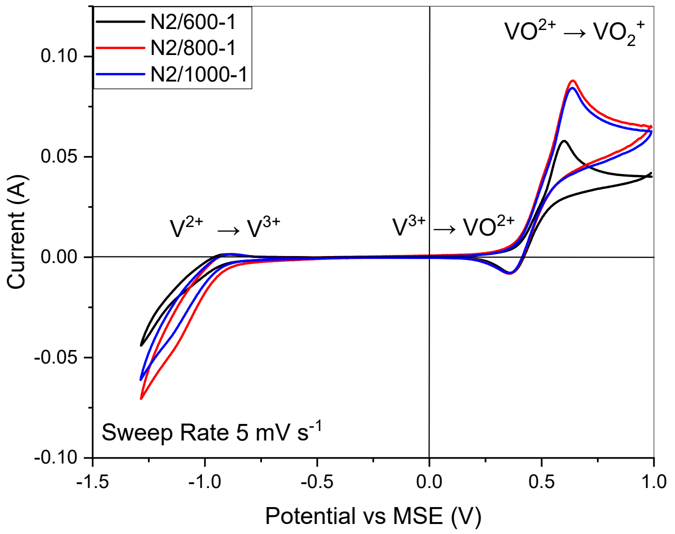

b)

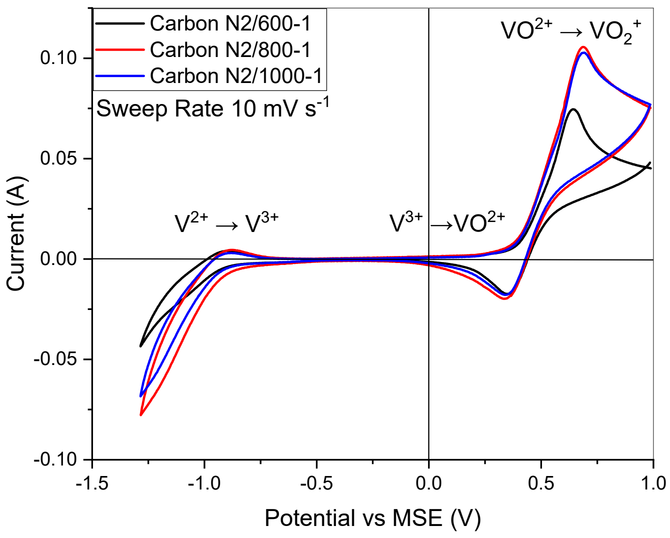

Figure S16. Comparison of cyclic voltammograms for vanadium redox reactions with carbon composite electrodes from carbonization in Nitrogen a) Sweep Rate 5mV s^{-1} and b) Sweep rate 10 mV s^{-1}

a)

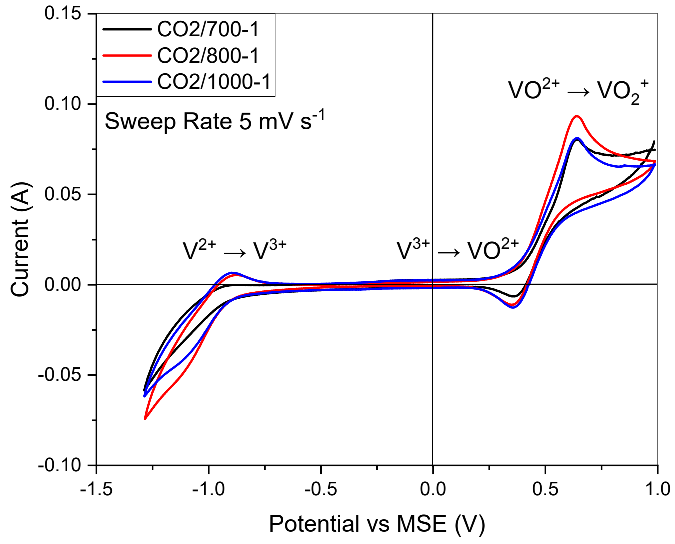

b)

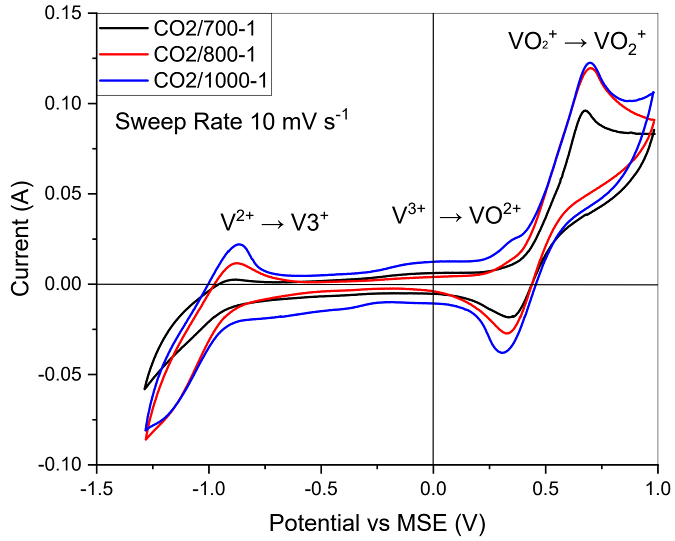

Figure S17. Comparison of cyclic voltammograms for vanadium redox reactions with carbon/PVDF composite electrodes made from carbons prepared in carbon dioxide atmosphere at various heat treatment temperatures a) Sweep Rate 5 mV s^{-1} and b) Sweep Rate 10 mV s^{-1}.

2.1.3 Cyclic Voltammograms as a Function of Sweep Rate

a)

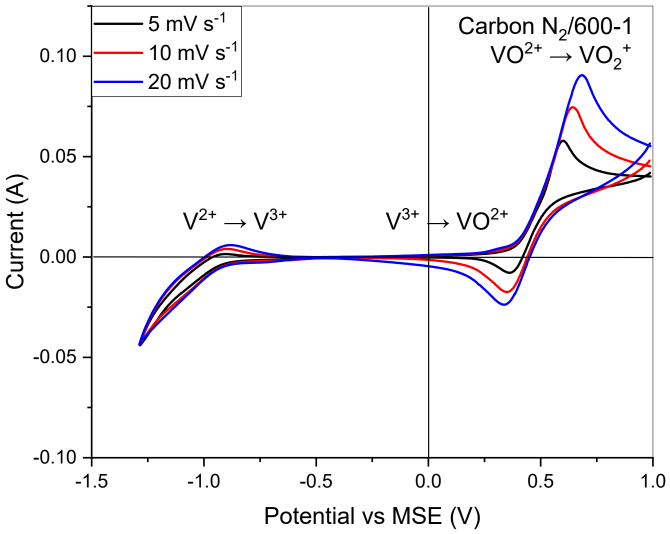

b)

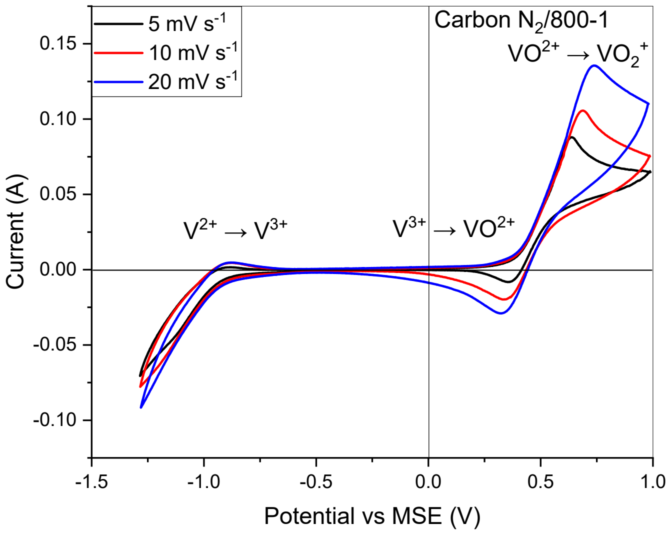

c)

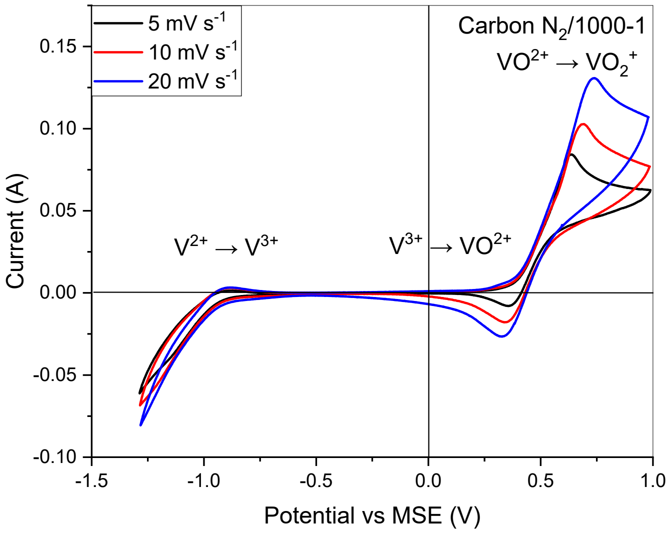
5 mV s-1
10 mV s-1
20 mV s-1
Carbon N2/1000-1
VO2+ → VO2+
V2+ → V3+
V3+ → VO2+
Current (A)
Potential vs MSE (V)
0.15
0.10
0.05
0.00
-0.05
-0.10
-1.5
-1.0
-0.5
0.0
0.5
1.0

d)

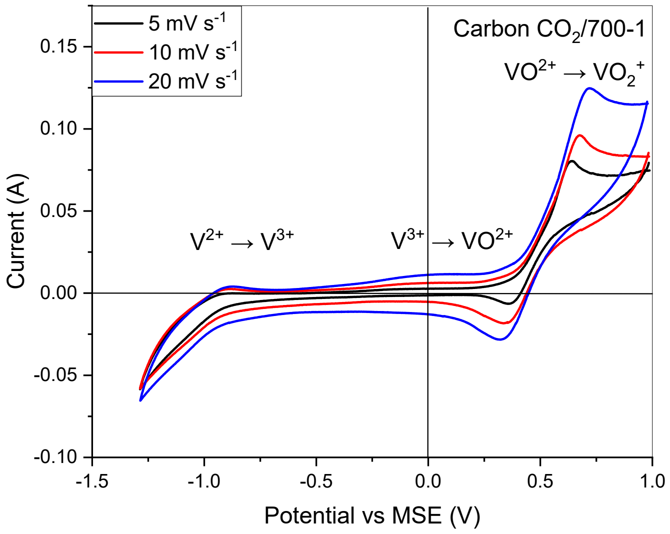
5 mV s-1
10 mV s-1
20 mV s-1
Carbon CO2/700-1
VO2+ → VO2+
V2+ → V3+
V3+ → VO2+
Current (A)
Potential vs MSE (V)
0.15
0.10
0.05
0.00
-0.05
-0.10
-1.5
-1.0
-0.5
0.0
0.5
1.0

e)

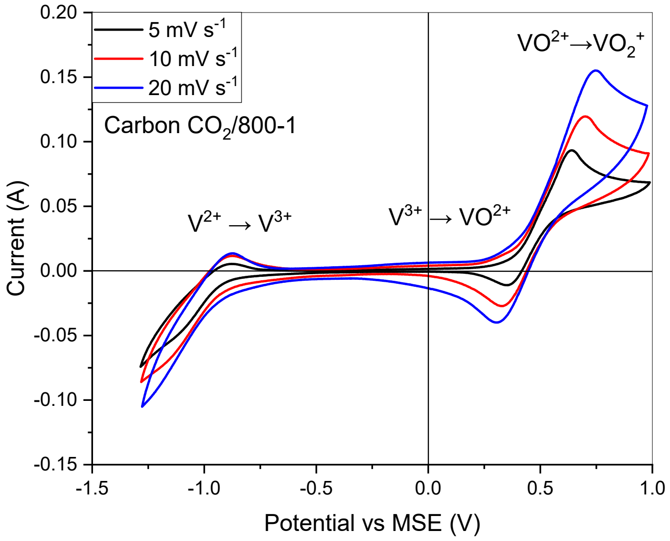

f)

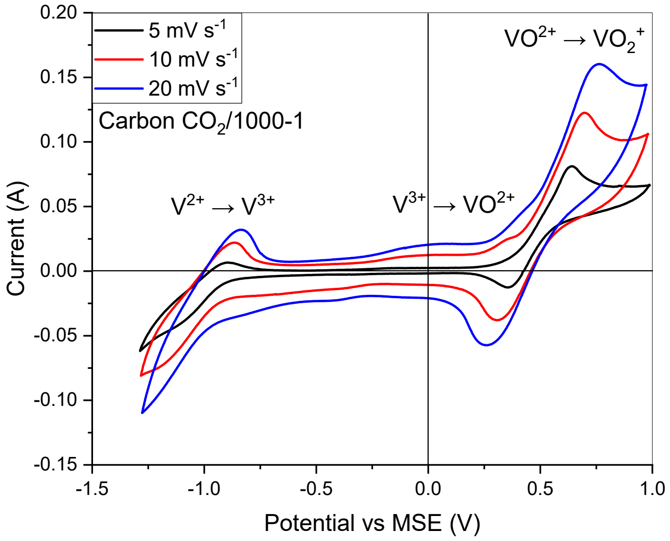

Figure S18 Comparison of cyclic voltammograms for vanadium redox reactions with carbon/PVDF composite electrodes made from carbons prepared in nitrogen and carbon dioxide atmospheres for sweep rates of 5, 10, and 20 mV s^{-1} a) Carbon N_2/600-1, b) Carbon N_2/800-1, c) Carbon N_2/1000-1, d) Carbon CO_2/700-1, e) Carbon CO_2/800-1, f) Carbon CO_2/1000-1

2.1.4 Comparison of Cyclic Voltammograms (Sweep Rate 20 mV s^{-1}) for Electrodes made from Carbons prepared under Nitrogen and Carbon Dioxide Atmospheres with corresponding Functionalized Carbons

a)

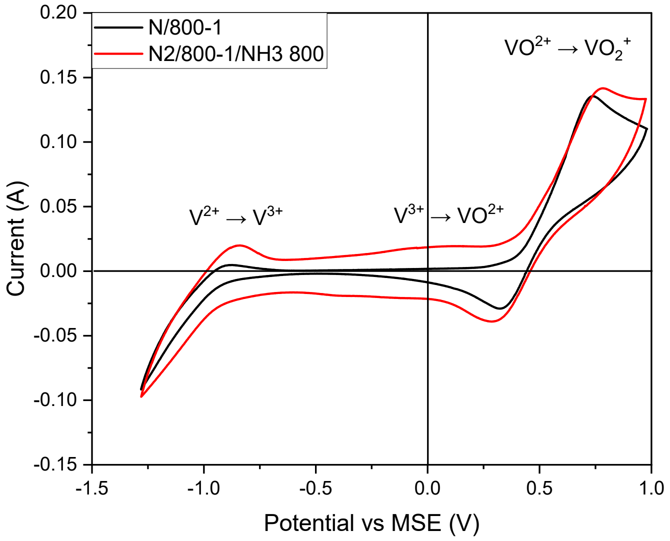

b)

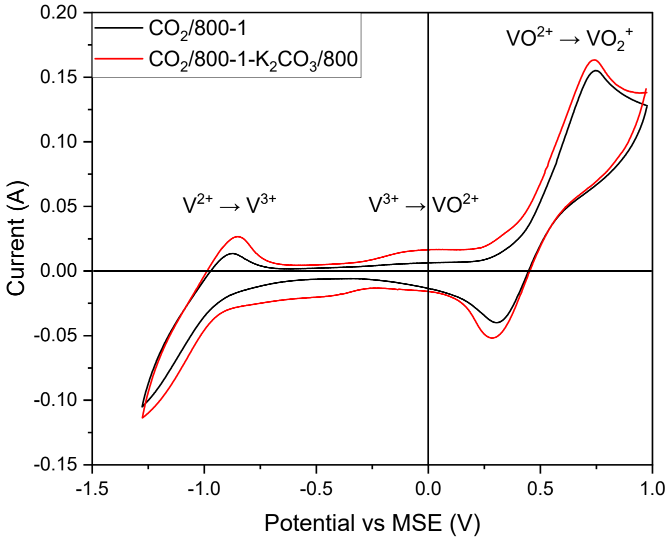

c)

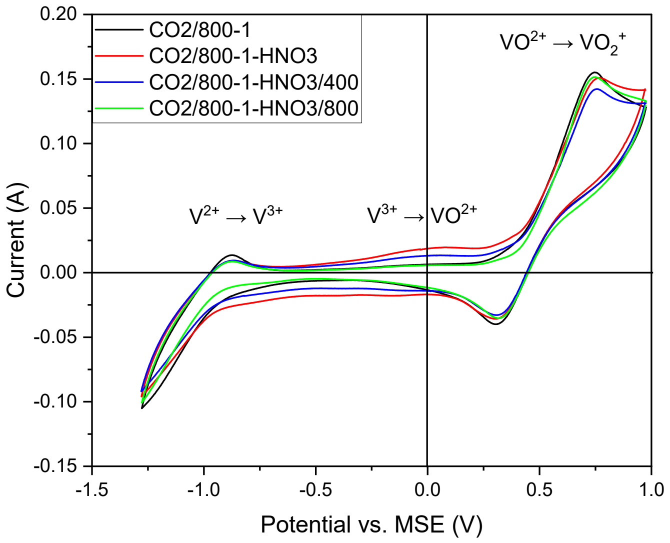

Figure S19. Comparison of cyclic voltammograms (Sweep Rate 20 mV s^{-1}) for vanadium redox reactions with carbon/PVDF composite electrodes made from functionalized carbons a) Carbons N_2/800-1 and N_2/800-1-NH_3/800; b) Carbons CO_2/800-1 and CO_2/800-1-K_2CO_3 and c) Carbons CO_2/800-1, CO_2/800-1-HNO_3, CO_2/800-1-HNO_3/400 and CO_2/800-1-HNO_3/800

2.2 Modeling of Electrical Impedance Spectra

EIS measurements probe the distribution of process timescales that the electrodes exhibit as the frequency of the probe signal in the range of 0.1 MHz to 100 kHz. The physical effects that may be observed include capacitance effects due to films, diffusion in bulk electrolytes, and the electrode structure, for example, porous structure, and distribution of timescales within heterogeneous samples. Many of the timescales are associated with specific length scales related to the thickness of electrode films, faradaic reactions at interfaces, diffusion over characteristic distances, etc.

The range of different carbons used to fabricate the electrodes suggests that modeling the EIS characteristics with a simple model is not anticipated. The model (see Figure S20a) used was applicable to all the samples investigated, but some components were not necessary for some electrodes (See Figure S20).

a)

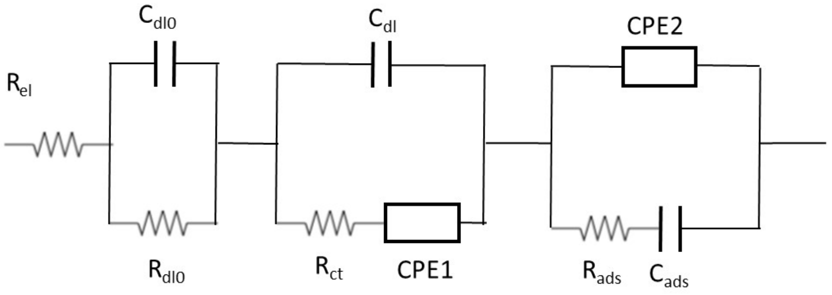

b)

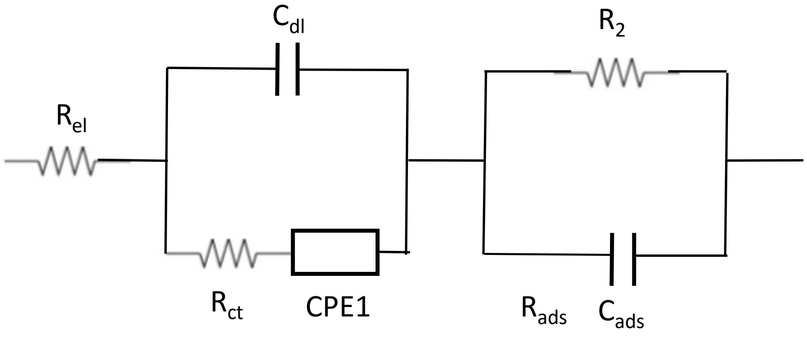

Figure S20. Electrical circuit models for electric impedance spectroscopy a) Full model b) Simplified model.

C_{dl0}, $R_{dl0,}$ and R_{dl} are the resistances and capacitance of the double layer. R_{ads} and C_{ads} are adsorption resistance and capacitance, respectively. R_{ct} is the charge transfer resistance. CPE1 and CPE2 are constant phase elements, which have Warburg impedances of the form:

$$Z = \frac{Yinv}{(j\omega)^{\varphi}} \quad \text{(S1)}$$

The angular frequency is ω , and j is √(−1). When the exponent φ approaches 1, the impedance *Z* becomes a capacitance with *Yinv* equal to the inverse of the capacitance, whereas when φ approaches zero, it becomes a resistor with *Yinv* equal to the resistance. The parameters are given in Supporting Information Table S1. However, in some cases, not all the parameters were required to reproduce the EIS graph (Table S1).

The carbon samples oxidized with HNO_3 and thermally treated gave low |ImZ| values, and the capacitance behavior of the samples was different from the other samples in the series. The only fit obtained for the samples involved taking the complex conjugate of the normal expression for Z given above.

$$Z = Yinv2\ ((i\omega)^{-\varphi}) \quad \text{(S2)}$$

$$= \frac{Yinv2}{\omega^{\varphi}}\left(\exp\left(\frac{-i\pi\varphi}{2}\right)\right) \quad \text{(S3)}$$

$$= \frac{Yinv2}{\omega^{\varphi}}\left(\cos\left(\frac{\pi\varphi}{2}\right) - i\,sin\left(\frac{\pi\varphi}{2}\right)\right) \quad \text{(S4)}$$

The imaginary part of this equation is negative. However, where a negative capacitance might be expected, it is usually where more complex chemical reactions or adsorption effects are observed. It is possible for the imaginary part of the expression above to become positive, allowing the capacitance component to become negative. In this case, the equation for impedance is below.

$$Z = \frac{Yinv2}{\omega^{\varphi}}\left(exp\left(\frac{i\pi\varphi}{2}\right)\right) \quad \text{(S5)}$$

This expression for the impedance of *Yinv2* in the circuit above allowed the CO_2/800-1-HNO_3 and CO_2/800-1-HNO_3/400 samples to be fitted; no other circuit was found to fit these data.

In the case of the electrode made from the NH_3 treated carbon (N_2/800-1-NH_3/800), a simplified model based on the above model provided a satisfactory description of the electrical impedance characteristics (See Figure 12b). The low frequency circuit is replaced by a simple parallel resistance/capacitance in which R_{ads} and $\phi 2$ in the circuit above are zero. CPE2 reverts to a resistor (R_2) rather than *Yinv2*.

In (R,C) circuits, the physical significance of the capacitance is not reflected directly in either the height or width of a feature; the response is represented by a semicircle whose diameter is equal to the value of *R* and whose height is *R/2*. The capacitance reflects the frequency at which the maximum in the semicircle is observed. At this maximum $\omega_{max}CR = 1$, particularly for lower frequency structures, there is often a relationship between ω_{max} and some characteristic distance δ. The relationship is of the form

$$\delta^2 = D/\omega_{max}^2 \qquad (S6)$$

where *D* is the diffusion coefficient. Given the values of R_2 and C_{ads}, ω_{max} = 11:8, and assuming that the diffusion coefficient is approximately 10^{-5} $cm^2 sec^{-1}$, a length of 9 μm is obtained. This is possibly a pore length. The value of $\phi 1$ is significantly lower than 0.45, which is expected for a Warburg component, reflecting the porous nature of the composite electrode.

Table S1. EIS model parameters a) Carbonization under nitrogen; b) Carbonization under carbon dioxide; c) CO_2/800-1-HNO_3 and CO_2/800-1-HNO_3/400; d) NH_3 treated carbon N_2/800-1-NH_3/800 e) K_2CO_3 treated carbon CO_2/800-1-K_2CO_3/800

a) Carbonization under Nitrogen

**Electrode Carbon**	**N_2/600-1**	**N_2/800-1**	**N_2/1000-1**
**Parameter**			
R_{el}	1.021 ± 0.006	0.9359 ± 0.0028	0.9825 ± 0.0055
C_{dl0}		9.984 ± 0.535 x10^{-5}	5.812 ± 0.379 x 10^{-5}
R_{dl0}		0.1186 ± 0.0026	0.1858 ± 0.0056
R_{ct}	0.2729 ± 0.0051	0.0749 ± 0.0023	
C_{dl}	3.282 ± 0.136 x10^{-5}	1.438 ± 0.007 x 10^{-3}	4.050 ± 0.324x 10^{-4}
ϕ1	0.2649 ± 0.0023	0.3105 ± 0.0031	0.1793 ± 0.0015
Yinv1	0.9352 ± 0.0071	0.5751 ± 0.0043	1.031 ± 0.0085
R_{ads}			
C_{ads}	0.4972 ± 0.0397	0.3344 ± 0.0399	0.5276 ± 0.0278
ϕ2			
Yinv2	0.08759 ± 0.00442	0.02911 ± 0.00265	0.9332 ± 0.00346

b)Carbonization under Carbon dioxide

**Electrode Carbon**	**CO_2/700-1**	**CO_2/800-1**	**CO_2/1000-1**
**Parameter**			
R_{el}	7.992 ± 0.073 x 10^{-1}	5.767 ± 0.014 x 10^{-1}	8.661 ± 0.038 x10^{-1}
C_{dl0}		1.951 ± 0.084 x10^{-4}	1.697 ± 0.184 x10^{-4}
R_{dl0}		7.271 ± 0.128 x10^{-2}	7.962 ± 0.352x10^{-2}
R_{ct}	1.603 ± 0.033 x10^{-1}	4.683 ± 0.115 x 10^{-2}	4.276 ± 0.284 x10^{-2}
Cdl	6.268 ± 0.266 x10^{-5}	4.644 ± 0.257 x 10^{-3}	4.018 ± 0.536 x10^{-3}
ϕ1	8.111 ± 0.718 x 10^{-1}	8.448 ± 0.598 x10^{-1}	3.778 ± 0.060 x10^{-1}
Yinv1	7.348 ± 1.412 x 10^{-2}	4.558 ± 0.718 x 10^{-2}	4.103 ± 0.047x10^{-1}
R_{ads}	1.982 ± 0.106	Constrained to zero	
C_{ads}	3.761 ± 0.254 x 10^{-3}	4.714 ± 0.223 x 10^{-2}	1.510 ± 0.13 x10^{-1}
ϕ2	1.859 ± 0.070 x 10^{-1}	2.586 ± 0.052 x10^{-1}	
Yinv2	8.537 ± 0.080 x 10^{-1}	3.528 ± 0.082 x 10^{-1}	6.308 ± 0.365x10^{-2}

c) HNO_3 treated CO_2/800-1 series.

**Electrode Carbon**	**CO_2/800-1-HNO_3**	**CO_2/800-1-HNO_3/400**
**Parameter**		
R_{el}	5.469 ± 0.043 x 10^{-1}	6.047 ± 0.045 x10^{-1}
C_{dl0}	2.476 ± 0.21 x 10^{-4}	1.840 ± 0.248x10^{-4}
R_{dl0}	5.305 ± 0.225 x 10^{-2}	6.443 ± 0.389x10^{-2}
R_{ct}	2.136 ± 0.209 x 10^{-2}	1.492 ± 0.339 x10^{-2}
C_{dl}	2.848 ± 0.471 x 10^{-3}	3.035 ± 0.956 x10^{-3}
ϕ1	3.175 ± 0.057 x 10^{-3}	3.364 ± 0.069 x10^{-1}
Yinv1	3.044 ± 0.040 x 10^{-1}	4.519 ± 0.061x10^{-1}
R_{ads}	1.534 ± 0.183 x 10^{-1}	2.298 ± 0.396x10^{-1}
C_{ads}	6.998 ± 0.697 x 10^{-2}	3.731 ± 0.668x10^{-2}
ϕ2	1.025 ± 0.056 x10^{-1}	2.413 ± 0.064x10^{-1}
Yinv2	1.337 ± 0.048 x 10^{-1}	2.247 ± 050x 10^{-1}

Note: The complex conjugate was used to calculate Yinv2.

d) NH_3 treated N_2/800-1 at 800°C

**Electrode Carbon**	**N_2/800-1-NH_3/800**
**Parameter**	
R_{el}	1.123 ± 0.003
R_{ct}	8.892 ± 0.28 x 10^{-3}
C_{dl}	1.057 ± 0.071 x 10^{-4}
ϕ1	3.134 ± 0.022 x 10^{-1}
Yinv1	5.719 ± 0.038 x 10^{-1}
R_2	4.913 ± 0.278 x 10^{-2}
C_{ads}	1.726 ± 0.149

e) K_2CO_3 treated Carbon CO_2/800-1-K_2CO_3/800

**Electrode Carbon**	**CO_2/800-1-K_2CO_3/800**
**Parameter**	
Rel	5.986 ± 0.016x10^{-1}
Cdl0	7.657 ± 0.351x10^{-5}
Rdl0	8.477 ± 0.209x10^{-2}
Rct	1.518 ± 0.192x10^{-2}
Cdl	2.227 ± 0.421x10^{-3}
ϕ1	3.425 ± 0.040x10^{-1}
Yinv1	4.466 ± 0.041x10^{-1}
Rads	
Cads	5.852 ± 0.563x10^{-1}
ϕ2	
Yinv2	3.253 ± 0.260 x10^{-2}

Note: The additional (Rdl0, Cdl0) circuit was needed to get a good fit for the high-frequency data.

2.3 Cell Performance Repeatability Measurements

Cell Construction Details

The order of assembly from outside to inside (for 1 side):

Cell frame – Insulator – Current collector – Bipolar plate – Felt holder and carbon felt.

Table S2 Cell Performance and repeatability studies

a) Cell parameters

Component	Mass (g)	Percentage (wt%)	Dimensions
Cell frame	260.712	62.36	
Bolts	36.5398	8.74	
Insulator	24.58	5.88	
Current collector	81.24	19.43	
Bipolar plate,	1.89	0.45	Active area 2 x 2 cm^2
PAN Felt holder/ case	7.368	1.76	
Carbon felt	0.3312	0.08	2 x 2 cm^2 Thickness 4.6 mm
Electrolyte	5.4 (4 mL)	1.29	
Carbon material and PVDF on bipolar plate	0.024	0.01	
Fumatech Fap-450 Anion Exchange membrane			

Data for both sides

Table S3a. Repeatability studies for average overall, coulombic, and voltage efficiencies for various carbons in cell performance cycle measurements a) average values for various current densities and b) individual cycle measurements

**Carbon**	**N_2/800-1**		**N_2/800-1-NH_3/800**		**CO_2/800-1-HNO_3**	
**Current Density mA cm^{-2}**	**Efficiency** %	**Error** %	**Efficiency** %	**Error** %	**Efficiency** %	**Error** %
	**Overall efficiency**					
10	90.48	0.12	90.23	0.93	89.60	0.46
15	90.90	0.09	90.17	0.99	89.47	0.33
20	90.10	0.13	88.98	1.17	88.43	0.26
	**Coulombic Efficiency**					
10	94.18	0.16	94.42	0.66	93.99	0.50
15	96.51	0.21	96.41	0.42	95.96	0.35
20	97.61	0.12	97.35	0.37	97.06	0.26
	**Voltage Efficiency**					
10	96.07	0.10	95.56	0.55	95.33	0.03
15	94.18	0.21	93.52	0.69	93.23	0.04
20	92.31	0.22	91.40	0.91	91.11	0.03

Table S3b. Repeatability studies for overall efficiencies for various current densities in cell performance cycle measurements for various carbons.

**Carbon sample**		**N_2/800-1**		**N_2/800-1-NH_3/800**		**CO_2/800-1-HNO_3**	
**Runs**		2		5		2	
**Cycle number**	**Current Density** mA cm^{-2}	**Efficiency** %	**Error** %	**Efficiency** %	**Error** %	**Efficiency** %	**Error** %
1	10	89.31	0.05	89.63	0.63	88.94	1.26
2	10	90.34	0.13	90.11	0.59	90.02	0.59
3	10	90.43	0.04	90.33	0.64	89.58	0.56
4	10	90.55	0.02	90.28	0.59	89.51	0.60
5	10	90.59	0.00	90.22	0.59	89.51	0.60
6	15	90.38	0.28	89.95	0.77	89.17	0.62
7	15	90.85	0.13	90.16	0.75	89.49	0.44
8	15	90.84	0.09	90.20	0.74	89.42	0.50
9	15	90.91	0.05	90.19	0.76	89.48	0.41
10	15	90.98	0.02	90.12	0.75	89.47	0.41
11	20	89.75	0.23	88.86	0.93	88.20	0.38
12	20	90.09	0.16	89.13	0.95	88.45	0.33
13	20	90.13	0.20	89.11	0.93	88.43	0.40
14	20	90.12	0.08	88.91	1.11	88.44	0.31
15	20	90.06	0.19	88.76	0.92	88.39	0.32

Figure S21 Cell Performance characteristics a) Repeatability of charge -Discharge data for carbon N_2/800-1-NH_3/800 as a function of number b) Charge-Discharge data for the four carbons used in the study

3. X-ray Photoelectron Spectroscopy

3.1 Repeatability studies

a)

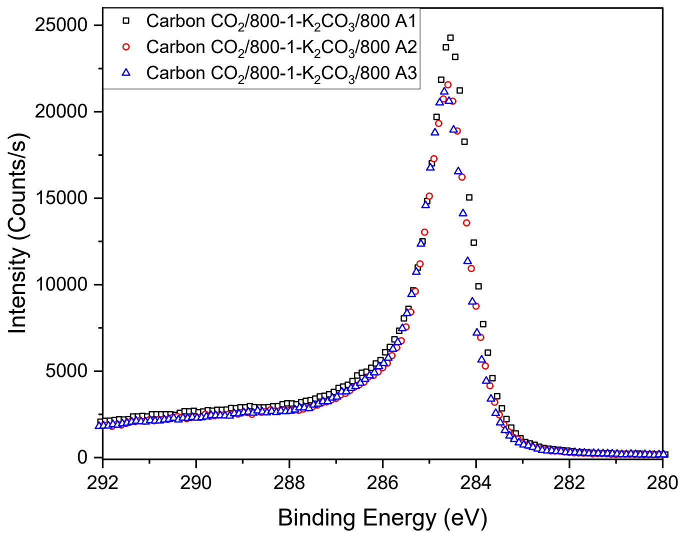

b)

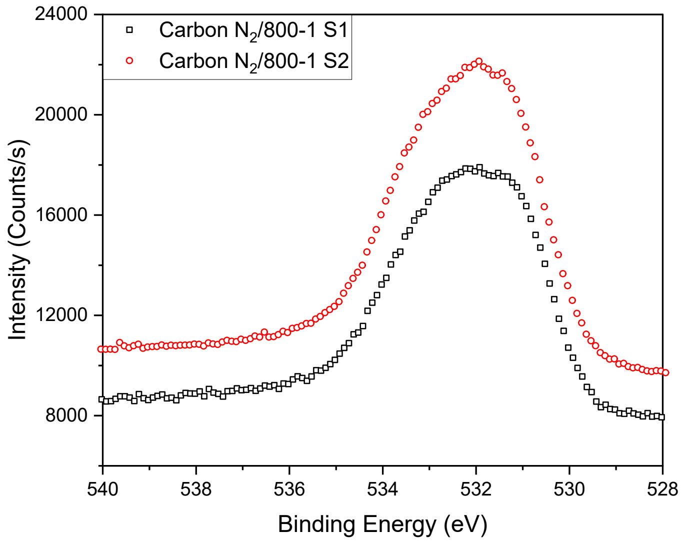

c)

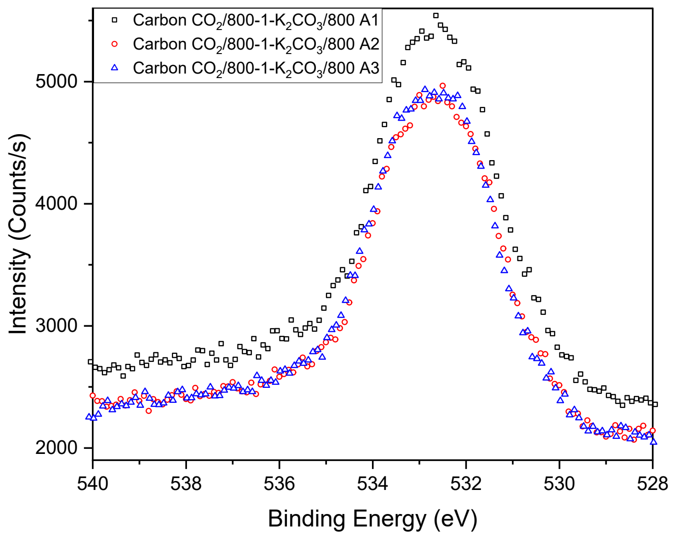
Carbon CO2/800-1-K2CO3/800 A1
Carbon CO2/800-1-K2CO3/800 A2
Carbon CO2/800-1-K2CO3/800 A3
Intensity (Counts/s)
Binding Energy (eV)
2000
3000
4000
5000
540
538
536
534
532
530
528

d)

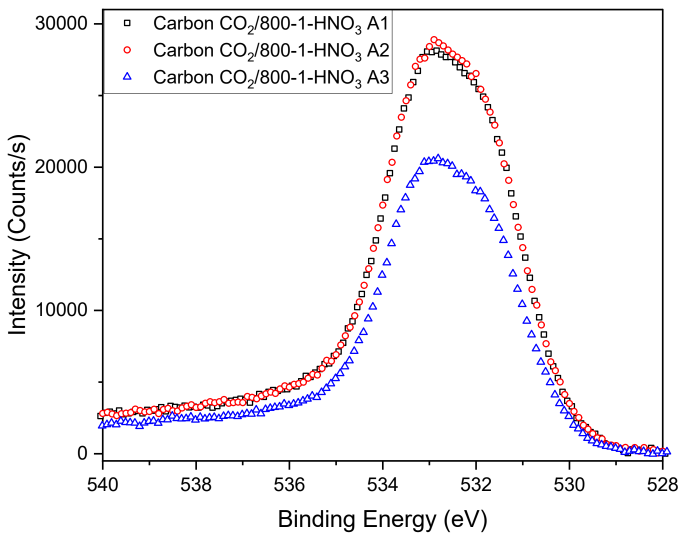
Carbon CO2/800-1-HNO3 A1
Carbon CO2/800-1-HNO3 A2
Carbon CO2/800-1-HNO3 A3
Intensity (Counts/s)
Binding Energy (eV)
0
10000
20000
30000
540
538
536
534
532
530
528

e)

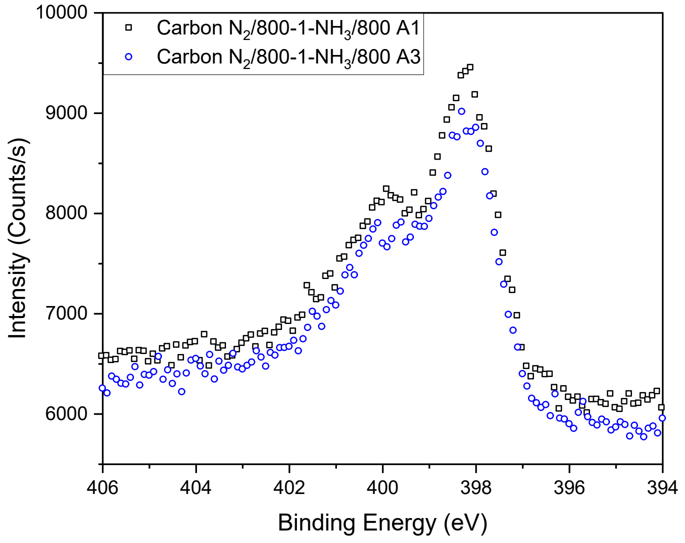

f)

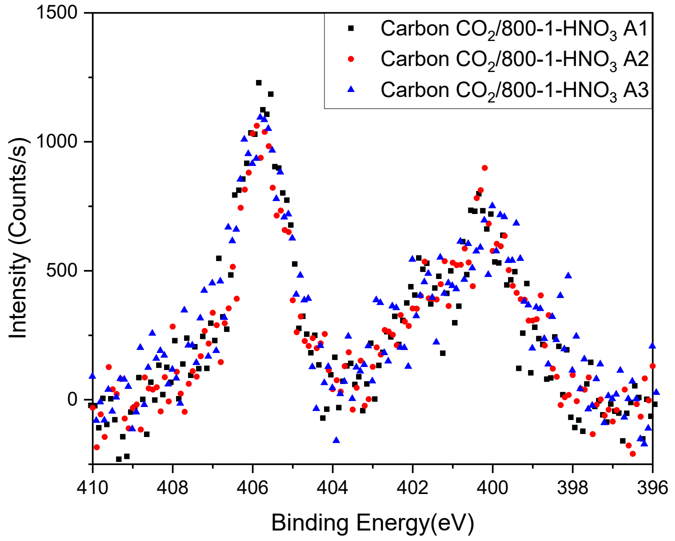

Figure S21. Repeatability comparisons for XPS spectra for the following samples a) C 1s Carbon CO_2/800-1-K_2CO_3/800, b) O 1s Carbon N_2/800-1, c) O 1s Carbon CO_2/800-1-K_2CO_3/800, d) O 1s Carbon CO_2/800-1-HNO_3, e) N 1s Carbon N_2/800-1-NH_3/800 and f) N 1s Carbon CO_2/800-1-HNO_3

3.2 Comparison of X-ray Photoelectron O 1s Spectra for various Hold Times

a)

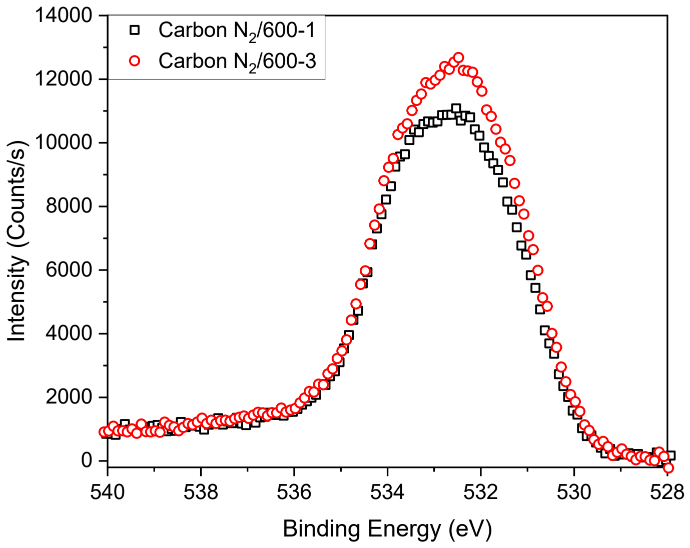

b)

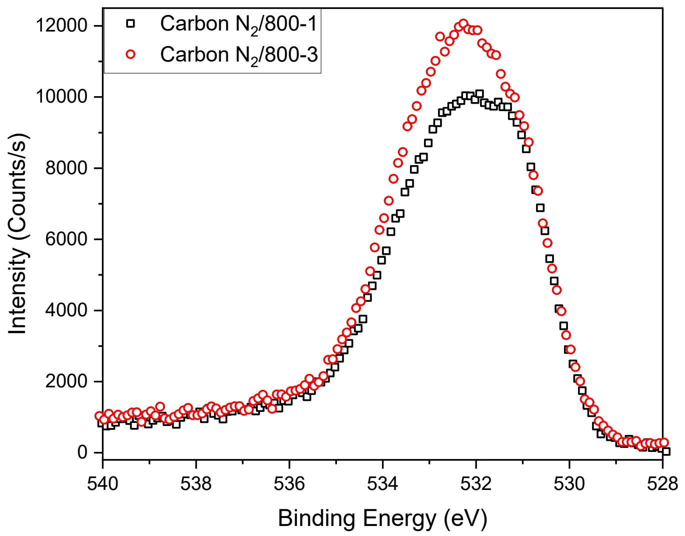

c)

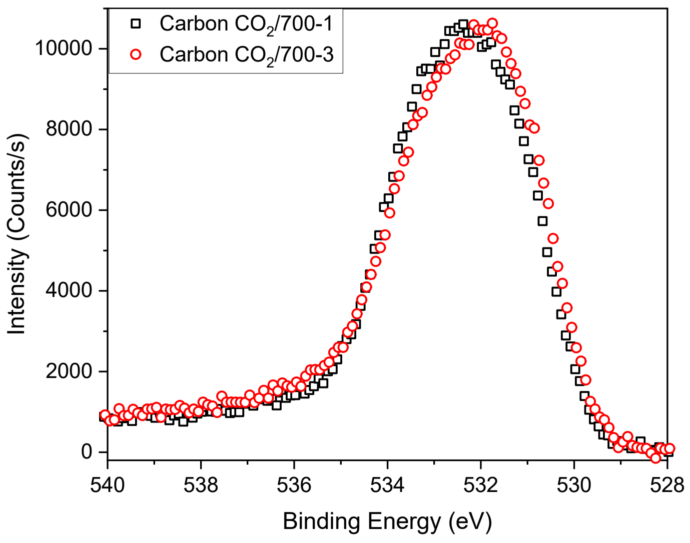

d)

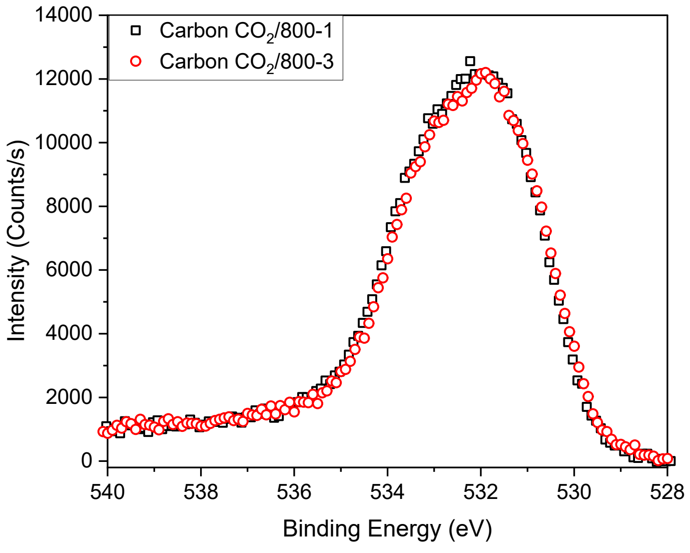

Figure S22. Comparison of XPS O1s spectra for samples with various hold times a) Carbons N_2/600-1 and N_2/600-3, b) Carbons N_2/800-1 and N_2/800-3, c) Carbons CO_2/700-1 and CO_2/700-3, and d) Carbons CO_2/800-1 and CO_2/800-3. Spectra have been amended by background subtraction using average intensity at 525 - 525.5 eV in order to compare peak shapes.

3.3 Comparison of X-ray Photoelectron O 1s Spectra for Series 1 (N_2 Atmosphere) and Series 2 (CO_2 Atmosphere) as a function of Heat Treatment Temperature

a)

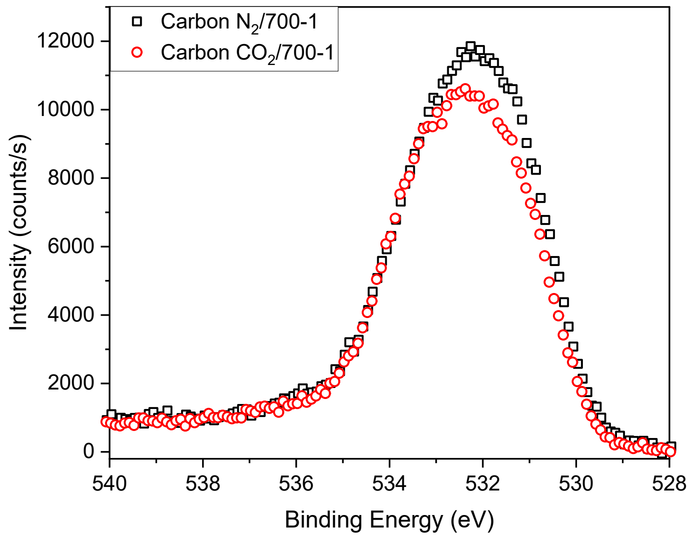

b)

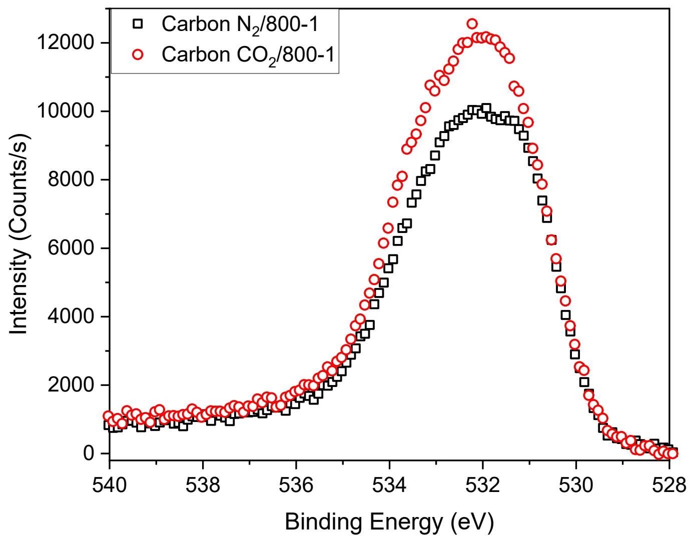

c)

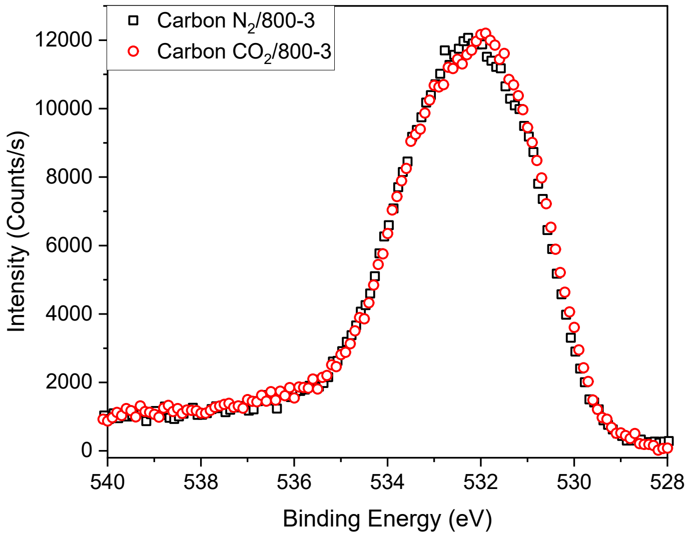

d)

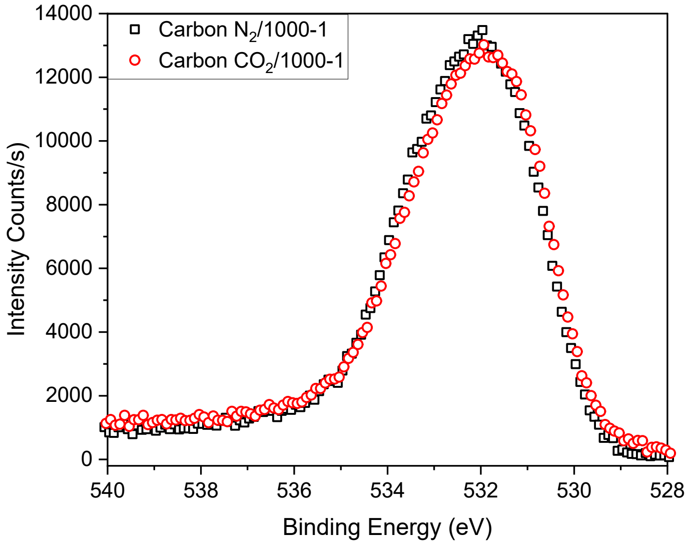

Figure S23. Comparison of X-ray Photoelectron O1s Spectra for Series1 (N_2 Atmosphere) and Series 2 (CO_2 Atmosphere) as a function of Heat Treatment Temperature a) Carbons N_2/700-1 and CO_2/700-1, b) Carbons N_2/800-1 and CO_2/800-1, c) Carbons N_2/800-3 and CO_2/800-3, and d) Carbons N_2/1000-1 and CO_2/1000-1, Spectra have been amended by background subtraction using average intensity at 525 - 525.5 eV in order to compare peak shapes.

3.4 Comparison of X-ray Photoelectron Spectra for Various Treatment Methods with Original Materials

a)

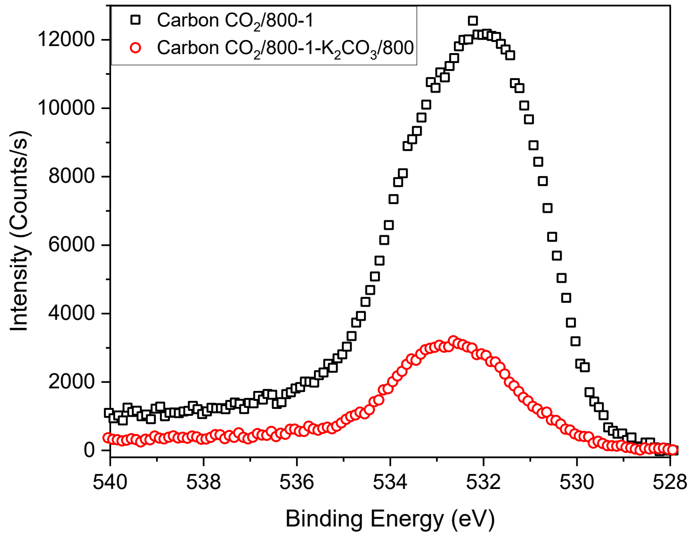

b)

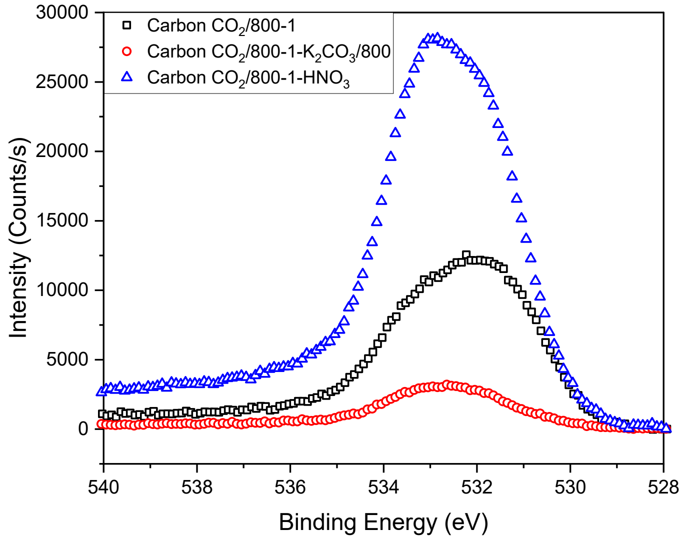

v

Figure S24. Comparison of X-ray Photoelectron O 1s Spectra for a) Carbons CO_2/800-1 and CO_2/800-1-K_2CO_3/800 and b) Carbons CO_2/800-1, CO_2/800-1-K_2CO_3/800 and CO_2/800-1-HNO_3. Spectra have been displayed after subtraction of the background, which was taken as the average intensity at 525 - 525.5 eV to compare peak shapes.

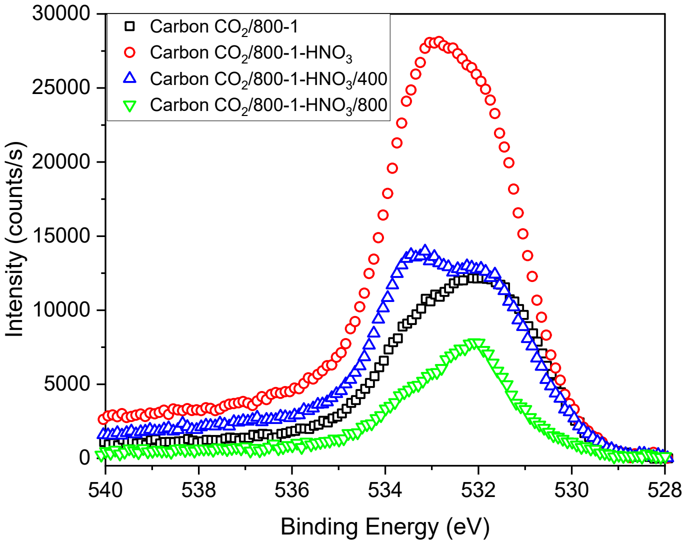

Figure S25. Comparison of X-ray Photoelectron O 1s Spectra for the HNO_3 oxidized and Heat-Treated Series of Carbons CO_2/800-1, CO_2/800-1-HNO_3, CO_2/800-1-HNO_3/400 and CO_2/800-1-HNO_3/800. Spectra have been amended by background subtraction using average intensity at 525 - 525.5 eV to allow comparison of peak shapes.

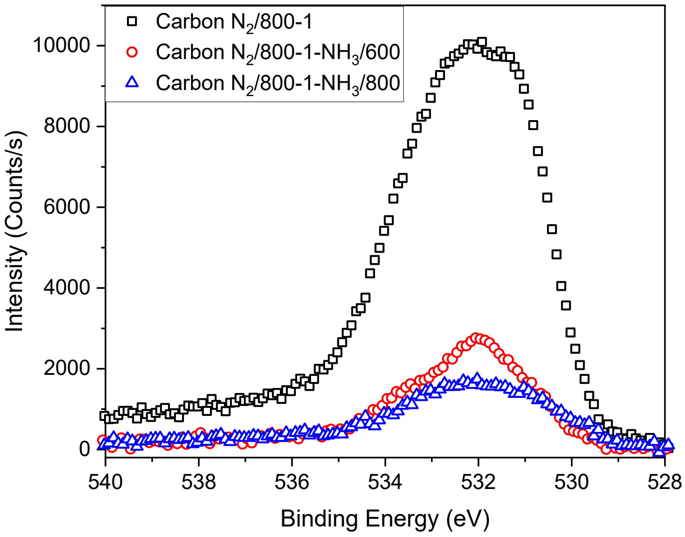

Figure S26. Comparison of X-ray Photoelectron O 1s Spectra for NH_3 Treated series Carbons N_2/800-1, N_2/800-1-NH_3/600 and N_2/800-1-NH_3/800. Spectra have been subjected to background subtraction using average intensity at 525 - 525.5 eV to allow comparison of peak shapes.

3.5 XPS N 1s Spectra for Chemically Treated and Original Carbons.

a)

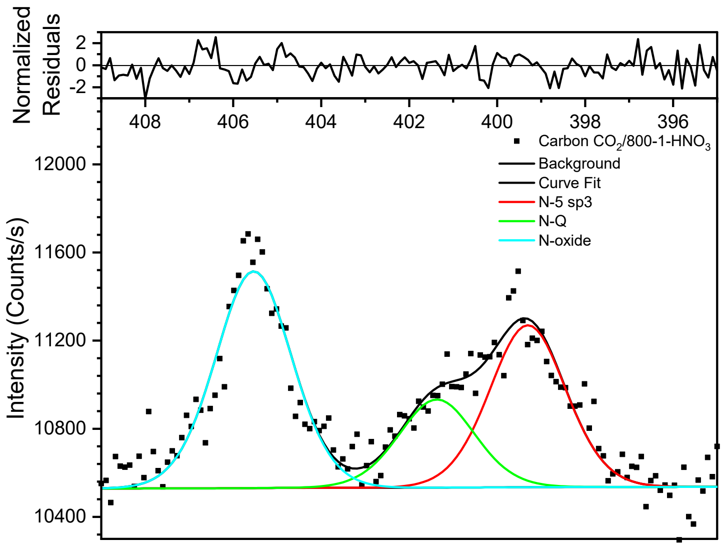

b)

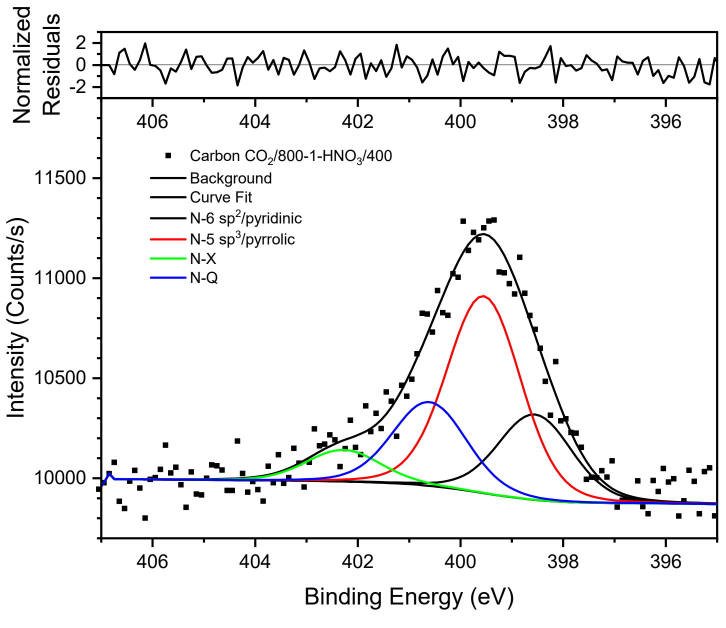

c)

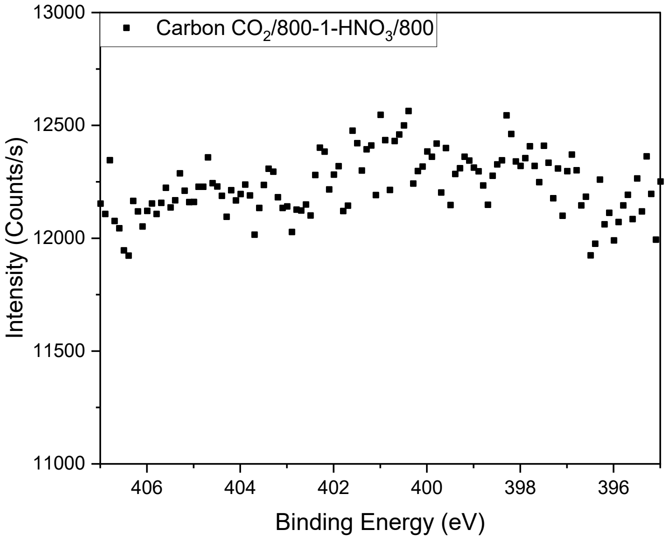

Figure S27. XPS N 1s spectra of a) Carbon CO_2/800-1-HNO_3, b) Carbon CO_2/800-1-HNO_3/400 and c) Carbon CO_2/800-1-HNO_3/800

a)

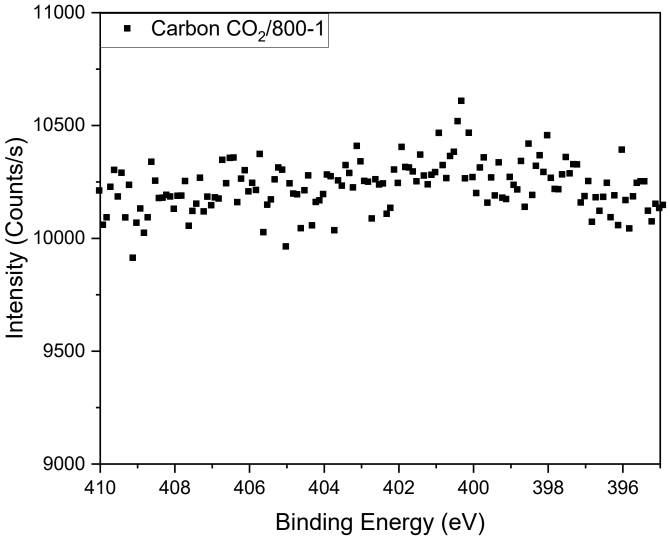

b)

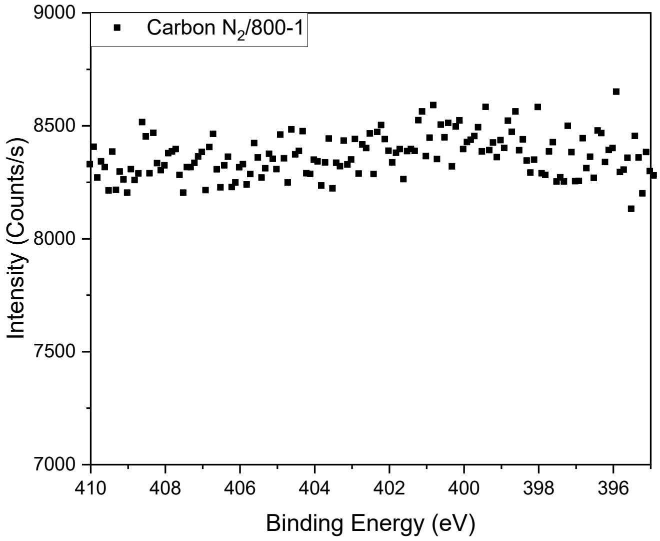

Figure S28. XPS N 1s spectra of a) Carbon CO_2/800-1 and b) Carbon N_2/800-1

3.6 Curve Resolution Data for X-ray Photoelectron Spectra

Table S4 Repeatability Studies for Curve Fitting XPS data a) C 1s, b) O 1s, c) N 1s and d) N 1s NH_3 treatment

a)

**C 1s spectra** Carbon Sample	Distribution of Peak Areas (%)			Peak Binding Energies (eV)			FWHM (eV)	Casi χ^2
	Peak I C-O/OH	Peak 2 C=O	Peak 3 O-C=O	Peak 1 C-O/OH	Peak 2 C=O	Peak 3 O-C=O		
**N_2/800-1**								
S 1	53.53	30.27	16.20	286.52	287.97	289.27	1.44	3.15
S 2	60.81	28.14	11.05	286.28	287.58	290.08	1.37	3.07
Average	57.17	29.21	13.62	286.40	287.78	289.68	1.41	
Standard Deviation	5.15	1.50	3.64	0.17	0.28	0.57	0.05	
**CO_2/800-1-K_2CO_3/800**								
A 1	57.04	24.04	18.92	286.30	287.90	289.40	1.61	2.07
A 2	56.16	24.84	18.99	286.35	287.90	289.25	1.38	2.08
A 3	54.96	25.01	20.03	286.33	287.68	289.03	1.23	2.76
Average	56.05	24.63	19.31	286.32	287.82	289.22	1.40	
Standard Deviation	1.05	0.52	0.62	0.03	0.12	0.18	0.19	
**CO_2/800-1-HNO_3**								
A 1	55.49	0.02	44.49	286.65	287.15	288.65	1.53	8.32
A 2	55.76	0.02	44.22	286.65	287.10	288.60	1.56	8.13
A 3	55.15	0.00	44.85	286.71	-	288.61	1.57	7.11
Average	55.47	0.019	44.52	286.67	287.12	288.62	1.55	
Standard Deviation	0.31	0.001	0.32	0.04	0.03	0.02	0.02	
**N_2/800-1-NH_3/800**								
A 1	63.01	22.326	14.66	286.28	287.98	289.78	1.61	3.27
A 2	63.02	23.294	13.69	286.21	287.96	289.91	1.61	2.67
A 3	62.62	24.426	12.95	286.16	287.71	289.11	1.57	2.67
Average	62.88	23.35	13.77	286.21	287.88	289.60	1.53	
Standard Deviation	0.23	1.05	0.86	0.06	0.15	0.43	0.04	

b)

**O 1s spectra**<br>Peak 1<br>Sample	Distribution of Peak Areas (%)<br>Peak 1<br>C=O	 Peak 2<br>CO/OH	 Peak 3<br>C-O	 Peak 4	 Peak 5	Peak Binding Energies (eV)<br>Peak 1<br>C=O	 Peak 2<br>C-O/OH	 Peak 3<br>O-C	 Peak 4	 Peak 5	FW HM (eV)	Casi χ^2	Abbe
**N_2/800-1**													
S 1	38.91	35.48	20.40	3.62	1.59	531.07	532.47	533.72	535.37	537.32	1.75	0.849	0.912
S 2	37.62	33.16	24.73	3.35	1.14	531.04	532.24	533.44	535.09	536.79	1.82	0.646	0.800
Average	38.27	34.32	22.57	3.49	1.36	531.06	532.36	533.58	535.23	537.06	1.79		
Standard Deviation	0.91	1.64	3.06	0.19	0.32	0.03	0.17	0.20	0.20	0.38	0.05		
**CO_2/800-1-K_2CO_3/800**													
A 1	11.89	43.39	35.98	5.84	2.90	530.90	532.20	533.45	535.80	538.30	1.96	0.890	0.934
A 2	13.68	42.08	36.48	5.46	2.30	531.05	532.20	533.50	535.60	537.65	1.83	0.855	0.791
A 3	9.66	45.80	36.08	5.67	2.79	530.83	532.23	533.58	535.73	537.93	1.87	0.840	0.850
Average	11.74	43.76	36.18	5.66	2.66	530.93	532.21	533.51	535.71	537.96	1.89		
Standard Deviation	2.01	1.89	0.26	0.19	0.32	0.11	0.02	0.07	0.10	0.32	0.07		
**CO_2/800-1-HNO_3**													
A 1	33.64	13.48	46.43	4.68	1.78	531.55	532.25	533.25	535.60	537.85	1.97	1.248	0.523
A 2	33.15	13.88	47.26	4.12	1.58	531.60	532.20	533.35	535.55	537.65	2.00	1.145	0.762
A 3	33.83	12.60	47.12	4.60	1.86	531.56	532.21	533.26	535.56	537.91	1.99	1.086	0.583
Average	33.54	13.32	46.93	4.47	1.74	531.57	532.22	533.29	535.57	537.80	1.99		
Standard Deviation	0.35	0.66	0.45	0.30	0.14	0.03	0.02	0.06	0.02	0.14	0.02		

c)

N 1s spectra Carbon Sample	Distribution of Peak Areas(%) N-sp^3	N-C-O	Nitro	Peak Binding Energies (eV) N-sp^3	N-C-O	Nitro	FWHM (eV)	Casi χ^2	Abbe
CO_2/800-1-HNO_3									
A 1	32.50	19.67	405.75	400.00	401.95	405.75	1.75	1.038	0.712
A 2	34.70	18.87	405.80	400.00	401.90	405.80	1.89	0.969	0.788
A 3	32.63	20.42	405.81	399.61	401.76	405.81	2.17	1.021	0.758
Average	33.28	19.65	405.79	399.87	401.87	405.79	1.94		
Standard Deviation	1.23	0.78	0.03	0.22	0.10	0.03	0.22		

d)

N 1s spectra Carbon Sample	Distribution of Peak Areas (%)						Peak Binding Energies (eV)						FWHM (eV)	Casi χ^2	Abbe
	N-sp^2	N-sp^3	N-Q	N-X	N?	NO	N-sp^2	N-sp^3	N-Q	N-X	N?	NO			
N_2/800-1-NH_3/800															
A 1	50.56	26.56	11.47	4.73	3.75	2.93	398.18	399.88	401.18	402.53	404.13	405.78	1.48	1.156	0.999
A 2	48.52	26.81	11.39	6.16	4.48	2.64	398.18	399.96	401.26	402.96	404.61	406.26	1.56	1.346	0.865
Average	49.54	26.69	11.43	5.44	4.12	2.78	398.18	399.92	401.22	402.74	404.37	406.02	1.52		
Standard Deviation	1.44	0.18	0.06	1.01	0.52	0.20	0.01	0.06	0.06	0.31	0.34	0.34	0.06		

Table S5 Curve Fitting Results for XPS data a) C 1s, b) O 1s, c) N 1s HNO_3 Oxidised series d) NH_3 treatment series

a)

C 1s spectra	Distribution of Peak Areas (%)			Peak Binding Energies (eV)				
Carbon Sample	C-O/OH	C=O	O-C=O	C-O/OH	C=O	O-C=O	FWHM (eV)	Casi χ^2
**Series 1**								
N_2/600-1	53.3	27.4	19.2	286.38	287.43	288.58	1.25	1.40
N_2/600-3	57.0	27.2	15.7	286.58	287.98	289.08	1.28	1.96
700-1	56.2	27.2	16.5	286.51	288.01	289.36	1.43	2.52
N_2/800-1	57.2 ± 5.2	29.2 ± 1.5	13.6 ± 3.6	286.40 ± 0.17	287.78 ± 0.28	289.68 ± 0.57	1.41 ± 0.05	
N_2/800-3	57.3	26.3	16.4	286.52	288.02	289.32	1.53	2.45
N_2/1000-1	61.2	25.2	13.6	286.52	287.97	289.02	1.58	2.41
**HTT 700-1000°C Average**	58.0 ± 2.2	27.0 ± 1.7	15.0 ± 1.6	286.49 ± 0.06	287.94 ± 0.11	289.34 ± 0.27		
**Series 2**								
CO_2/700-1	60.7	25.4	13.9	286.48	287.88	288.78	1.34	2.25
CO_2/700-3	55.3	28.4	16.3	286.55	288.00	288.75	1.47	2.77
CO_2/800-1	60.1	24.5	15.4	286.53	287.93	288.83	1.46	2.82
CO_2/800-3	61.0	27.3	11.7	286.50	288.10	288.95	1.53	2.81
CO_2/1000-1	56.5	27.2	16.3	286.48	288.08	289.48	1.62	2.04
**HTT 700-1000°C Average**	58.7 ± 2.6	26.6 ± 1.6	14.7 ± 1.9	286.51 ± 0.03	288.00 ± 0.10	288.96 ± 0.30		
**Series 3 Chemical Treatment**								
CO_2/800-1-K_2CO_3	56.1 ± 1.0	24.6 ± 0.5	19.3 ± 0.6	286.32 ± 0.03	287.82 ± 0.12	289.22 ± 0.18	1.40 ± 0.19	
CO_2/800-1-HNO_3	55.5 ± 0.3	0.02 ± 0.001	44.5 ± 0.3	286.67 ± 0.04	287.12 ± 0.03	288.62 ± 0.02	1.55 ± 0.02	
CO_2/800-1-HNO_3/400	64.7	19.7	15.6	286.60	288.40	288.80	1.56	6.96

CO_2/800-1-HNO_3/800	93.1	0.2	6.7	287.14	288.29	288.74	1.93	10.04
N_2/800-1-NH_3/600	57.6	24.7	17.6	286.51	288.01	289.46	1.67	2.84
N_2/800-1-NH_3/800	62.9 ± 0.2	23.3 ± 1.1	13.8 ± 0.9	286.21 ± 0.06	287.88 ± 0.15	289.60 ± 0.43	1.57 ± 0.04	

b)

O 1s spectra	Distribution of Peak Area (%)					Binding Energies (eV)					FWHM	Casi χ^2	Abbe
Carbon Sample	C=O	CO/OH-Al	CO/OH-Ar	Chem H_2O/O_2	$\pi-\pi*$	C=O	CO/OH Al	CO/OH-Ar	Chem H_2O/O_2	$\pi-\pi*$	(eV)		
**Series 1**													
N_2/600-1	25.1	36.7	34.0	2.8	1.3	531.28	532.48	533.78	535.63	537.63	1.77	0.887	1.031
N_2/600-3	24.2	38.6	33.2	2.7	1.4	531.18	532.38	533.73	535.48	537.28	1.79	0.903	0.951
N_2/700-1	36.3	38.0	21.8	3.1	0.8	531.16	532.46	533.66	535.86	538.71	1.89	0.978	0.917
N_2/800-1	38.27	34.32	22.57	3.49	1.36	531.06	532.36	533.58	535.23	537.06	1.79	0.849	0.912
	0.91	1.64	3.06	0.19	0.32	± 0.03	± 0.17	± 0.20	± 0.2	± 0.38	± 0.05	0.646	0.800
N_2/800-3	32.8	37.8	24.3	3.6	1.5	531.07	532.37	533.57	535.17	537.02	1.77	0.998	0.923
N_2/1000-1	31.3	38.0	25.8	3.6	1.3	531.07	532.22	533.47	535.02	536.67	1.77	0.887	1.096
**HTT 700-1000C**													
Average	35.5	36.6	23.5	3.5	1.3	531.08	532.35	533.57	535.30	537.30	1.80		
St Dev	3.4	2.1	2.1	0.2	0.3	0.05	0.12	0.12	0.34	0.83	0.06		
**Series 2**													
CO_2/700-1	29.3	36.1	29.4	3.4	1.8	531.13	532.33	533.53	535.08	536.78	1.73	0.923	0.911
CO_2/700-3	36.4	33.1	24.1	4.6	1.8	531.15	532.40	533.60	535.65	537.85	1.87	1.002	0.730
CO_2/800-1	30.6	35.7	28.2	4.1	1.4	531.03	532.23	533.53	535.28	537.23	1.80	0.975	1.015
CO_2/800-3	29.7	35.9	29.2	3.6	1.7	531.00	532.10	533.40	535.10	536.90	1.82	0.993	0.903
CO_2/1000-1	36.3	36.4	23.2	3.2	0.8	531.03	532.23	533.43	534.98	536.43	1.80	0.901	1.051
HTT 700-1000C													
Average	32.5	35.4	26.8	3.8	1.5	531.07	532.26	533.50	535.22	537.04	1.80		
St Dev	3.6	1.3	2.9	0.6	0.4	0.07	0.11	0.08	0.27	0.54	0.05		
**Series 3**													
CO_2/800-1-K_2CO_3	11.74	43.76	36.18	5.66	2.66	530.93	532.21	533.51	535.71	537.96	1.89	0.840-	0.791-
	± 2.01	± 1.89	± 0.26	± 0.19	± 0.32	± 0.11	± 0.02	± 0.07	± 0.10	± 0.32	± 0.07	0.890	0.934

CO_2/800-1-HNO_3	33.54 ± 0.35	13.32 ± 0.66	46.93 ± 0.45	4.47 ± 0.30	1.74 ± 0.14	531.57 ± 0.03	532.22 ± 0.02	533.29 ± 0.06	535.57 ± 0.02	537.80 ± 0.14	1.99 ± 0.02	1.086-1.249	0.523-0.762
CO_2/800-1-HNO_3/400	29.96	18.78	43.68	5.01	2.57	531.35	532.20	533.50	535.95	537.95	2.00	1.494	0.418
CO_2/800-1-HNO_3/800	13.32	56.19	23.75	4.32	2.42	530.95	532.21	533.67	535.63	537.80	1.63	1.501	0.571
N_2/800-1-NH_3/600	27.84	47.21	21.61	3.34	-	531.06	532.21	533.71	535.96	-	1.67	0.875	0.868
N_2/800-1-NH_3/800	32.91	39.58	20.12	5.28	2.11	530.68	532.23	533.53	535.73	537.58	2.00	0.830	0.924

c)

**Carbon Sample**	Distribution of Peak Areas (%)					Binding Energies (eV)					FWHM (eV)	Casi χ^2	Abbe
	$N\text{-}sp^2$	$N\text{-} sp^3$	N-5 pyrrolic	NCO	Nitro	$N\text{-}sp^2$	$N\text{-}sp^3$	N-5 pyrrolic	N-Q	Nitro			
CO_2/800-1-HNO_3		33.28 ±1.23		19.65 ± 0.78	47.07 ± 0.71		399.87 ±0.22		401.87 ± 0.10	405.79 ± 0.03	1.94 ± 0.22	0.968-1.038	0.712-0.789
CO_2/800-1-HNO_3/400	21.46	49.51	21.08	7.95		398.55	399.55	400.60	402.30		1.69	0.892	0.975

d)

**N 1s spectra**	Distribution of Peak Areas (%)						Binding Energies (eV)						FWHM	Casi χ^2	Abbe
**Carbon Sample**	N-sp^2	N-sp^3, pyrrolic	N-Q	N-X	N?	N-oxide	N-sp^2	N-sp^3, pyrrolic	N-Q	N-X	N?	N-oxide	(eV)		
N_2/800-1-NH_3/600	48.31	25.27	11.96	6.78	4.40	3.27	398.36	399.91	401.18	402.53	404.13	405.78	1.54	1.010	0.944
N_2/800-1-NH_3/800	49.54	26.69	11.43	5.44	4.12	2.78	398.18	399.92	401.22	402.74	404.37	406.02	1.52	1.156-	0.865-
	±1.44	±0.18	±0.06	±1.01	±0.52	±0.20	±0.02	±0.06	±0.06	±0.31	±0.34	±0.34	±0.06	1.443	0.994

Some weak curve resolution peaks in the high energy tail of the spectra are within the noise level.

4. Temperature Programmed Desorption

a)

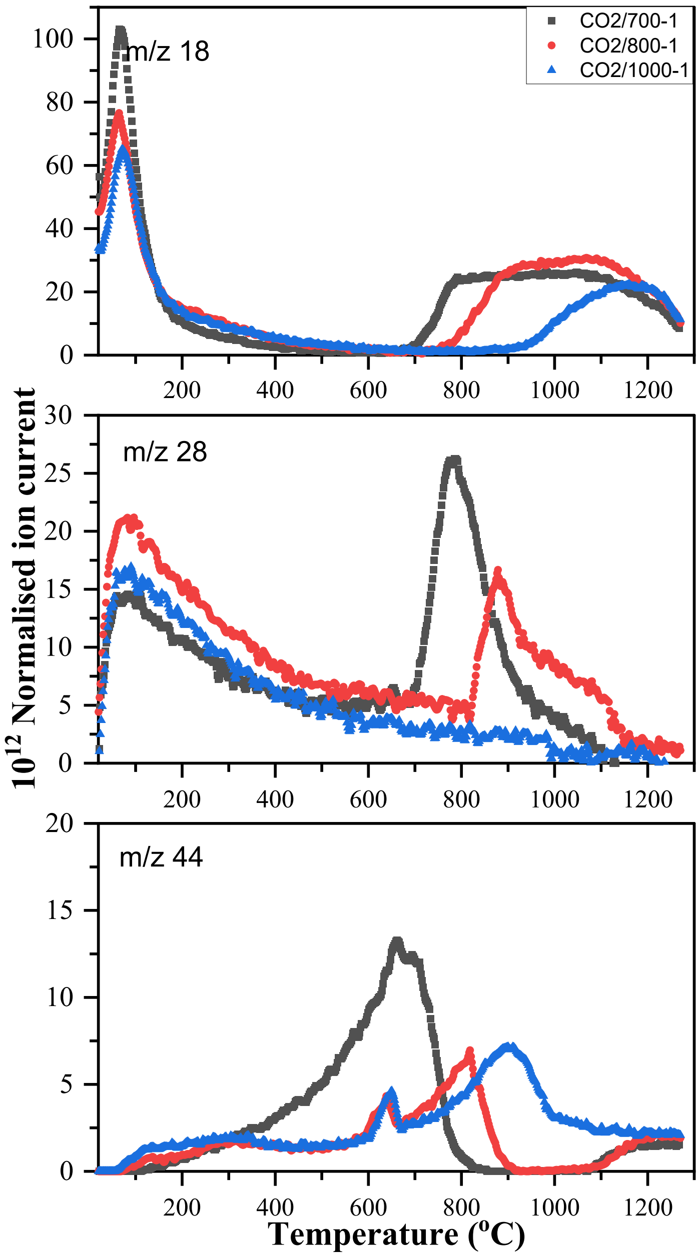

b)

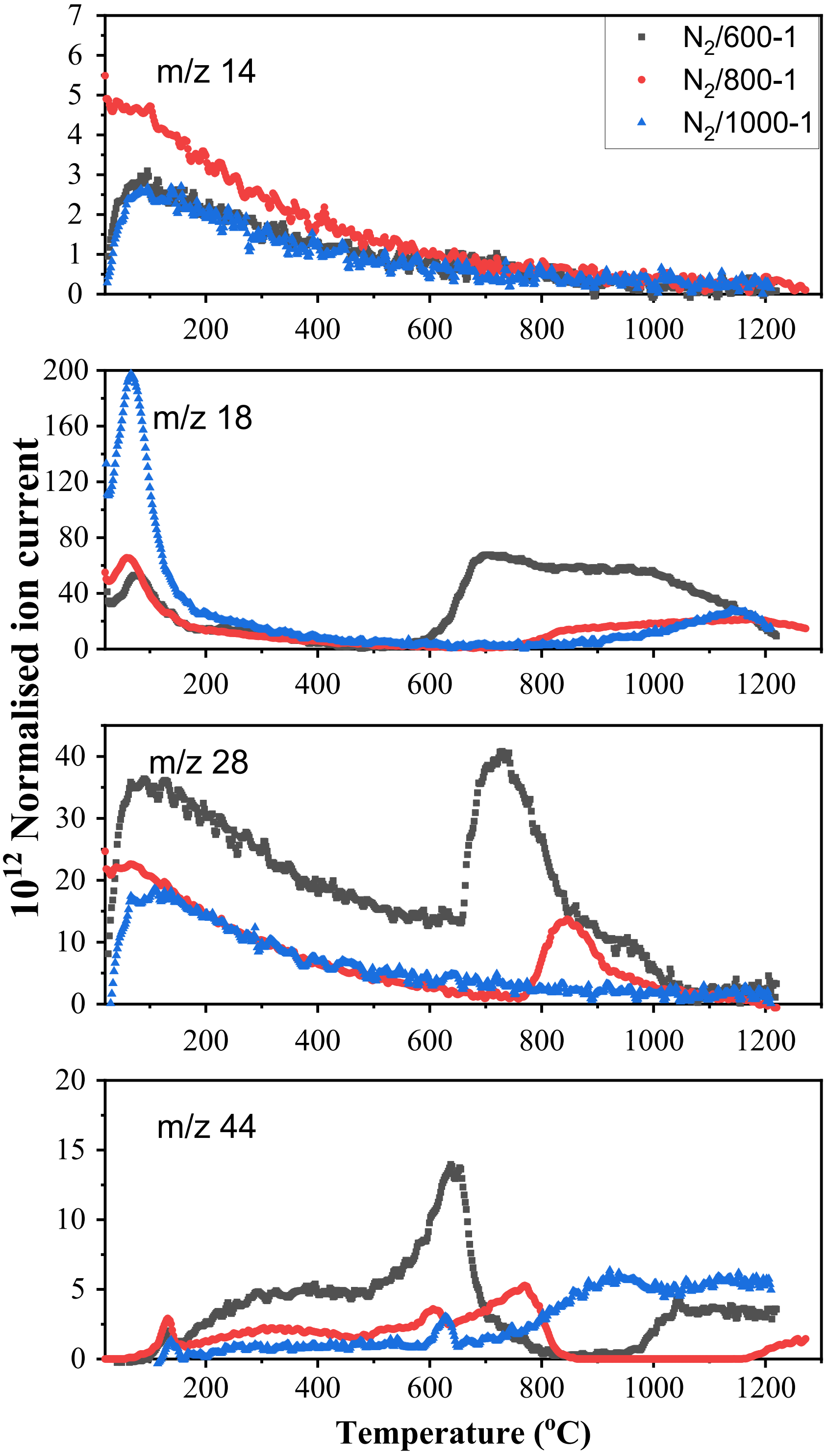

Figure S29 Comparison of TPD profiles for a) CO_2 series carbonization series 2, carbon CO_2/700-1, CO_2/800-1 and CO_2/1000-1, and b) nitrogen carbonization series 1: carbons N_2/600-1, N_2/800-1 and N_2/1000-1

5. Thermogravimetric Analysis

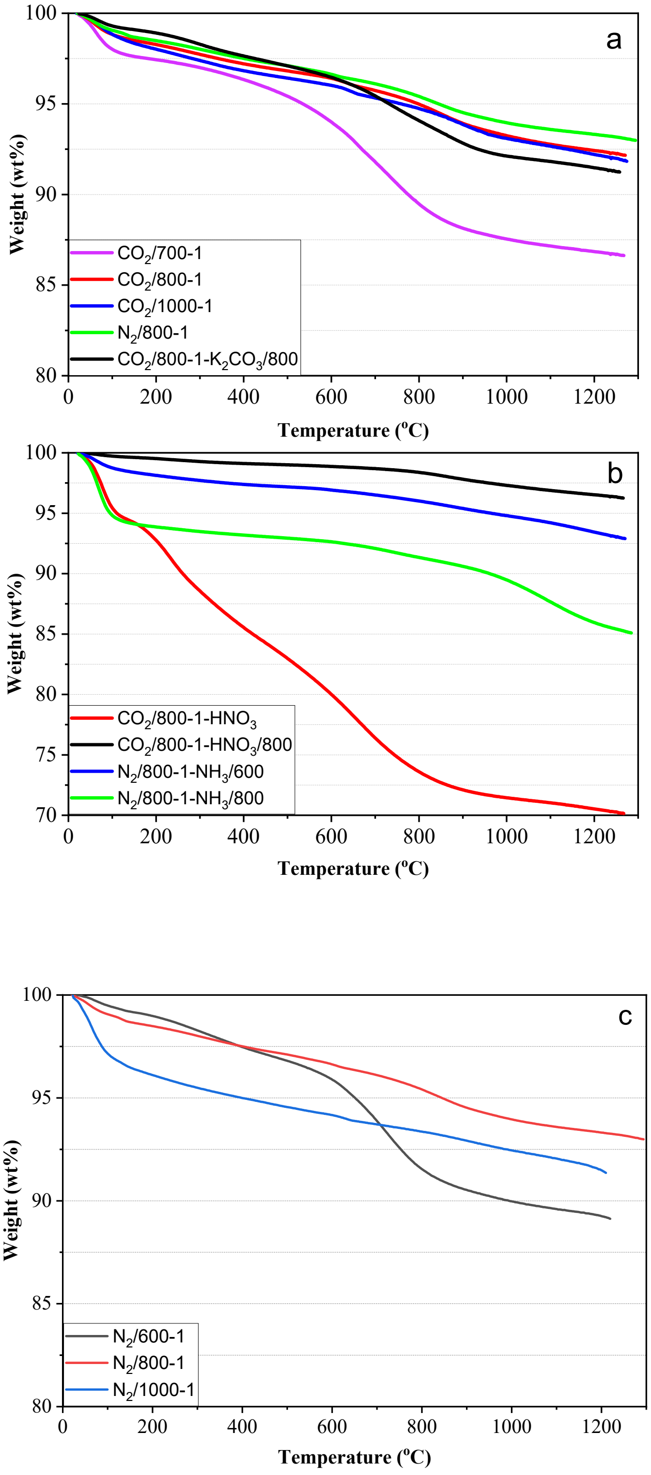

Figure S30 Comparison of thermogravimetric profiles for a) carbons CO_2/700-1, CO_2/800-1, CO_2/1000-1, N_2/800-1 and CO_2/800-1-K_2CO_3/800 b) carbons CO_2/800-1-HNO_3, CO_2/800-1-HNO_3/800, N_2/800-1-NH_3/600 and N_2/800-1-NH_3/800 and c) carbons N_2/600-1, N_2/800-1 and N_2/1000-1

6. Powder X-ray diffraction

a)

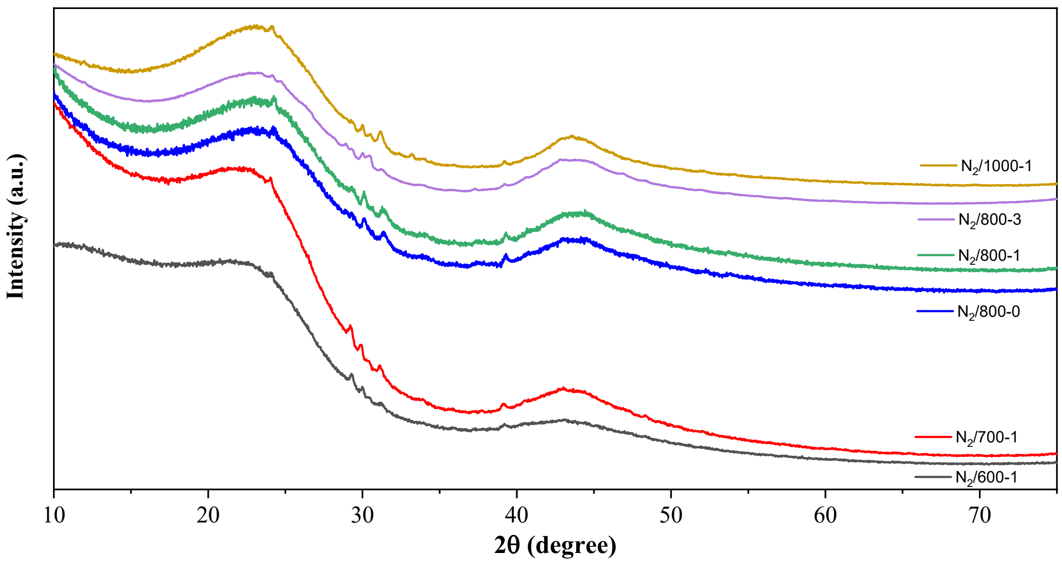

b)

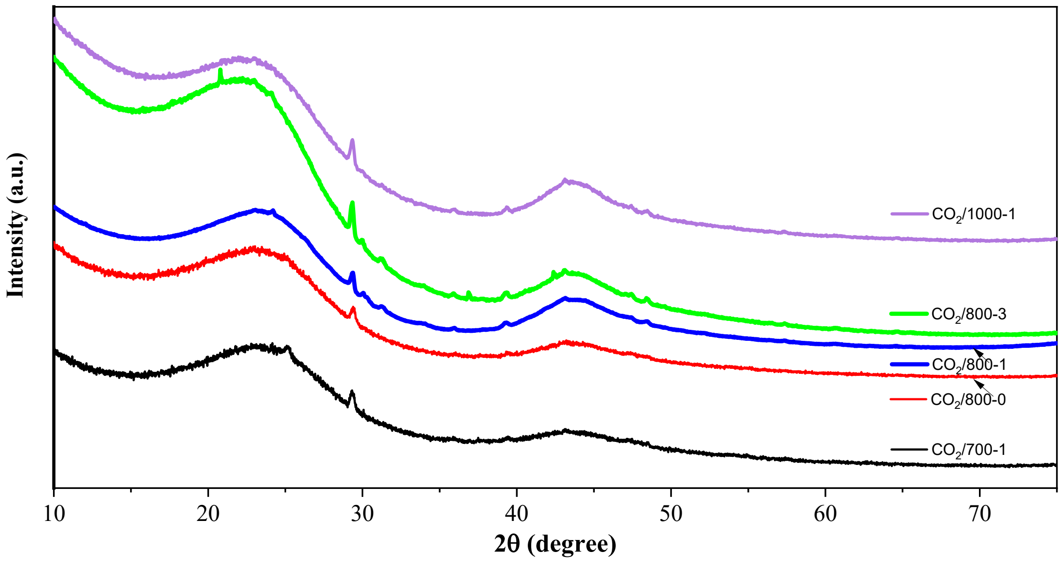

c)

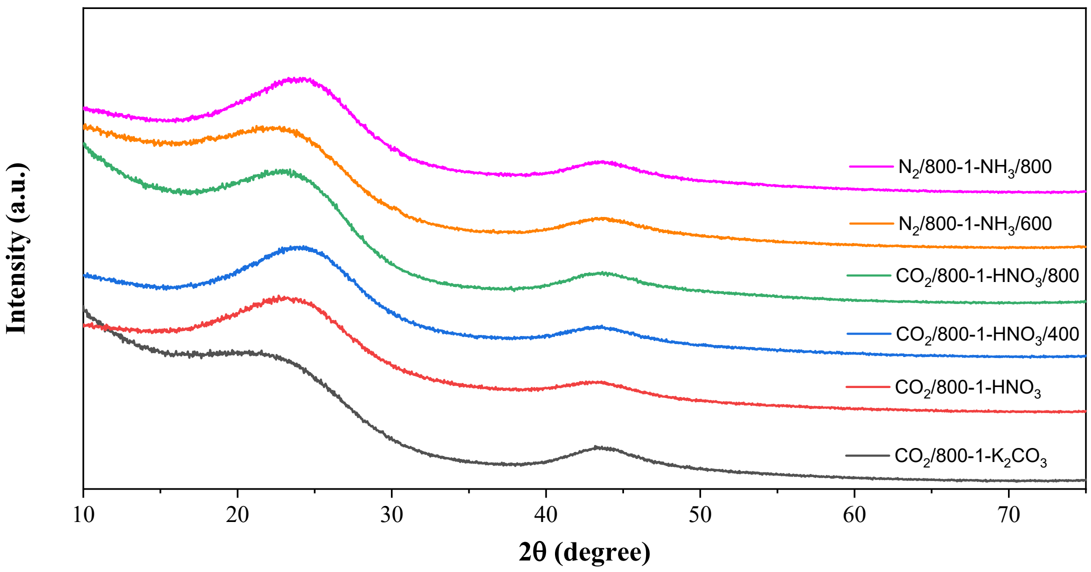

Figure S31 Comparison of powder X-ray diffraction profiles for a) Carbons prepared in a nitrogen atmosphere (Series 1), b) Carbons prepared in a carbon dioxide atmosphere (Series 2), and c) Functionalized carbons.

Table S6. Repeatability Studies for XRD Parameters (Å)

Series 1

Sample	N_2/800-3			
	Run 1	Run 2	Average	St dev
L_c	9.79	10.17	9.98	0.26
d_{002}	3.83	3.83	3.83	0.001
L_a	23.26	23.89	23.57	0.44

Series 2

Sample	CO_2/800-1			
	Run 1	Run 2	Average	St dev
L_c	9.91	10.36	10.14	0.32
d_{002}	3.86	3.85	3.86	0.005
L_a	23.15	23.92	23.53	0.54

Sample	CO_2/800-3			
	Run 1	Run 2	Average	St dev
L_c	9.28	9.37	9.32	0.06
d_{002}	3.86	3.86	3.86	0.00
L_a	24.65	23.97	24.31	0.48

7. Raman Spectroscopy

7.1 Repeatability of peak curve fitting

Table S7. Repeatability Studies for Curve Resolution of the Raman Spectra of Carbons

Series Sample	I_D/I_G	A_D/A_G	D Raman Peak $FWHM_D$	G Raman Peak $FWHM_G$	D Raman Peak	G Raman Peak	χ2	R^2
			cm^{-1}	cm^{-1}	cm^{-1}	cm^{-1}		
**Series 1**								
N_2/700-0 R1	0.828	1.440	163.8	63.8	1337.8	1597.5	87.2	0.993
N_2/700-0 R2	0.798	1.492	164.6	59.7	1337.2	1597.5	61.3	0.990
Average	0.813	1.466	164.2	61.7	1337.5	1597.5		
St Dev	0.021	0.037	0.6	2.9	0.5	0.03		
N_2/700-1 R1	0.847	1.534	163.3	61.1	1336.6	1598.5	66.0	0.992
N_2/700-1 R2	0.855	1.537	162.3	61.2	1336.0	1597.8	54.5	0.990
Average	0.851	1.535	162.8	61.1	1336.3	1598.2		
St Dev	0.006	0.002	0.7	0.1	0.4	0.5		
N_2/700-3 R1	0.903	1.543	160.2	63.5	1337.6	1597.5	63.0	0.992
N_2/700-3 R2	0.928	1.599	161.6	63.6	1337.3	1596.2	52.9	0.985
Average	0.916	1.571	160.9	63.6	1337.4	1596.9		
St Dev	0.018	0.039	1.0	0.04	0.3	0.9		
N_2/800-0 R1	0.941	1.670	155.0	59.2	1336.8	1599.5	60.5	0.991
N_2/800-0 R2	1.042	1.751	151.1	60.9	1338.1	1597.6	46.8	0.976
Average	0.992	1.711	153.1	60.1	1337.5	1598.6		
St Dev	0.071	0.057	2.7	1.2	0.9	1.4		
N_2/800-1 R1	0.972	1.617	156.1	63.6	1338.1	1599.6	34.8	0.992
N_2/800-1 R2	0.992	1.715	158.3	62.1	1339.0	1597.1	28.7	0.977
Average	0.982	1.666	157.2	62.8	1338.5	1598.3		
St Dev	0.014	0.069	1.6	1.0	0.6	1.8		
N_2/800-3 R1	1.014	1.711	152.1	61.1	1338.9	1599.6	43.8	0.991
N_2/800-3 R2	1.018	1.598	157.1	67.9	1341.4	1600.5	37.5	0.987
Average	1.016	1.654	154.6	64.5	1340.1	1600.0		
St Dev	0.003	0.080	3.5	4.8	1.8	0.7		
**Series 2**								
CO_2/700-1 R1	0.880	1.438	160.9	66.8	1339.5	1599.2	50.4	0.992

CO_2/700-1 R2	0.903	1.600	162.5	62.2	1335.9	1594.7	41.3	0.991
CO_2/700-1 R3	0.907	1.578	160.4	62.4	1336.5	1597.0	45.3	0.991
Average	0.897	1.539	161.3	63.8	1337.3	1597.0		
St Dev	0.014	0.088	1.1	2.6	2.0	2.3		
CO_2/700-3 R1	0.926	1.583	159.1	63.1	1337.5	1597.3	42.9	0.991
CO_2/700-3 R2	0.952	1.641	158.5	62.3	1336.5	1596.8	55.0	0.993
CO_2/700-3 R3	0.958	1.626	158.3	63.2	1335.6	1594.8	39.2	0.987
Average	0.945	1.617	158.6	62.8	1336.5	1596.3		
St Dev	0.017	0.030	0.4	0.5	1.0	1.3		
CO_2/800-1 R1	0.987	1.667	151.3	60.7	1337.5	1598.1	40.7	0.992
CO_2/800-1 R2	1.015	1.688	155.4	63.4	1338.1	1596.6	35.2	0.985
Average	1.001	1.677	153.4	62.0	1337.8	1597.4		
St Dev	0.020	0.015	2.9	1.9	0.4	1.1		
CO_2/800-3 R1	1.052	1.748	138.7	56.5	1337.2	1597.9	53.4	0.990
CO_2/800-3 R2	1.048	1.692	145.8	61.2	1336.6	1596.6	38.6	0.985
Average	1.050	1.720	142.3	58.9	1336.9	1597.3		
St Dev	0.002	0.040	5.0	3.3	0.4	0.9		

FWHM = Full Width Half Maximum, R1 = Run 1, R2 = Run 2, R3 = Run 3.

a)

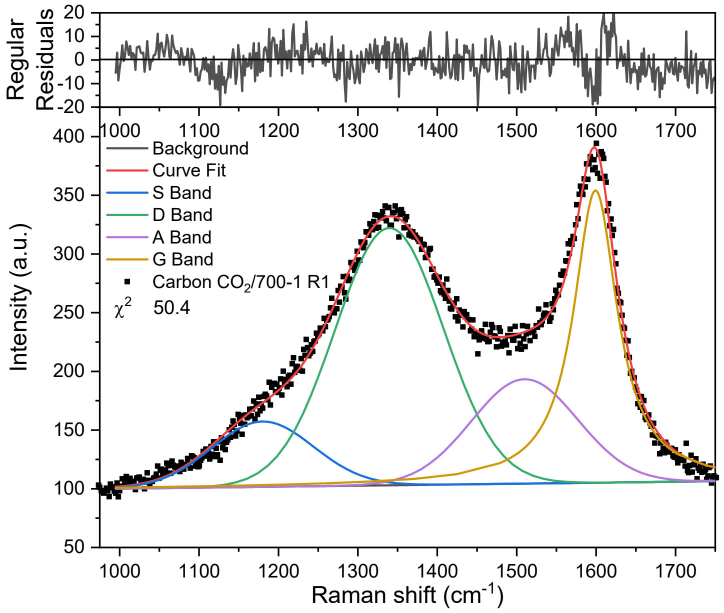

b)

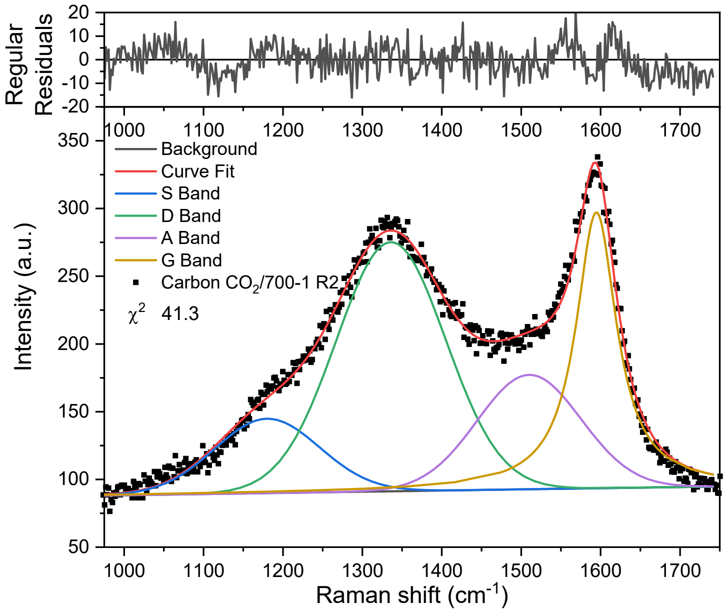

Regular Residuals
Intensity (a.u.)
Raman shift (cm^{-1})
Background
Curve Fit
S Band
D Band
A Band
G Band
Carbon CO_2/700-1 R2
χ^2 41.3

c)

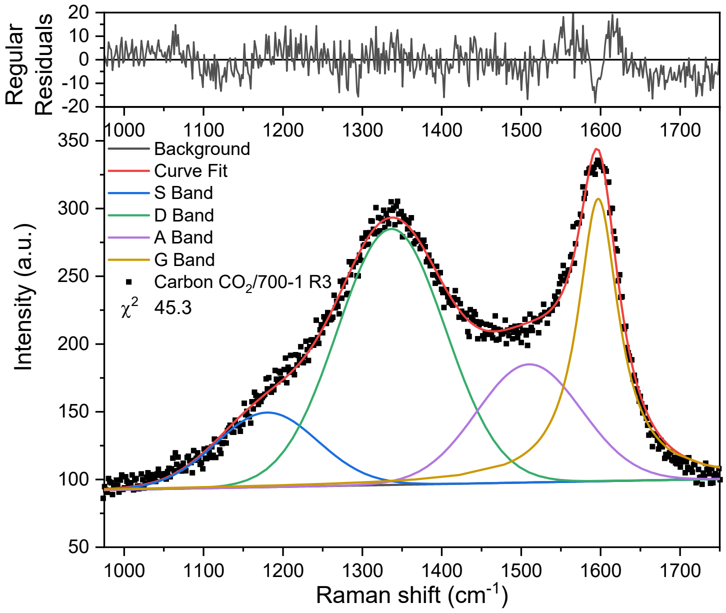

Regular Residuals
Intensity (a.u.)
Raman shift (cm^{-1})
Background
Curve Fit
S Band
D Band
A Band
G Band
Carbon CO_2/700-1 R3
χ^2 45.3

d)

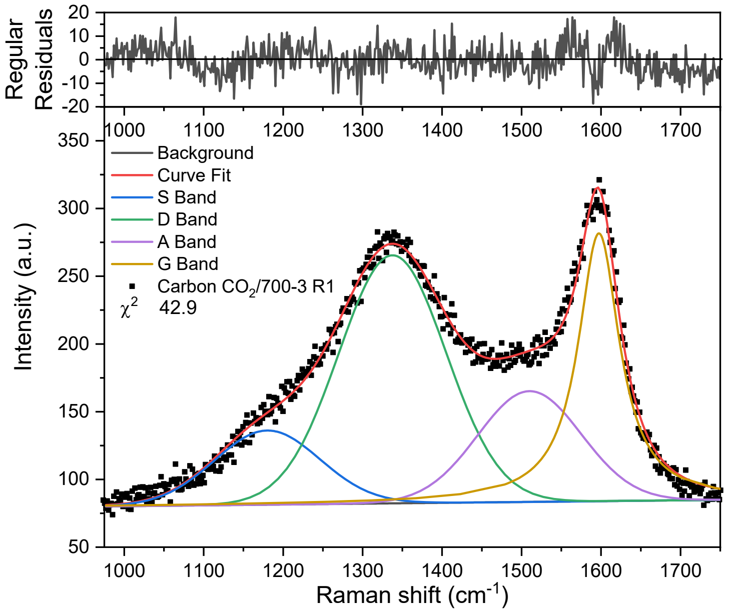
Regular Residuals
20
10
0
-10
-20
1000
1100
1200
1300
1400
1500
1600
1700
Background
Curve Fit
S Band
D Band
A Band
G Band
Carbon CO2/700-3 R1
χ2 42.9
Intensity (a.u.)
350
300
250
200
150
100
50
Raman shift (cm-1)

e)

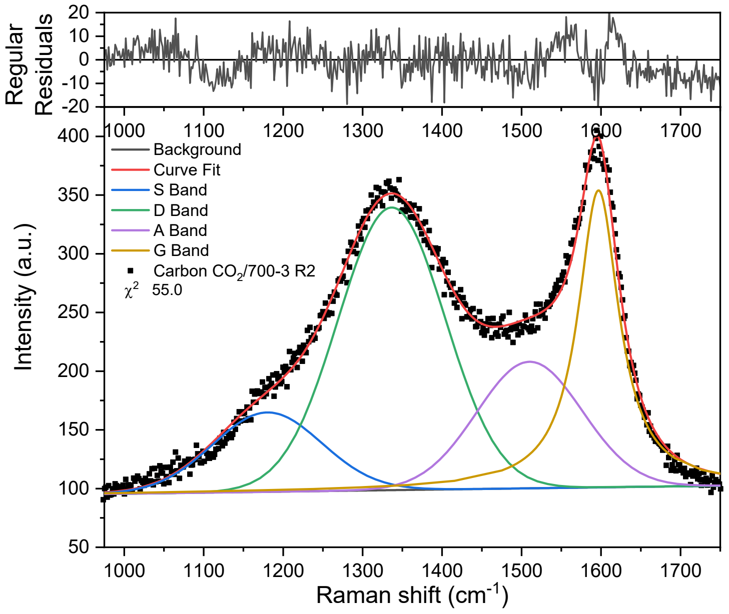
Regular Residuals
20
10
0
-10
-20
1000
1100
1200
1300
1400
1500
1600
1700
Background
Curve Fit
S Band
D Band
A Band
G Band
Carbon CO2/700-3 R2
χ2 55.0
Intensity (a.u.)
400
350
300
250
200
150
100
50
Raman shift (cm-1)

f)

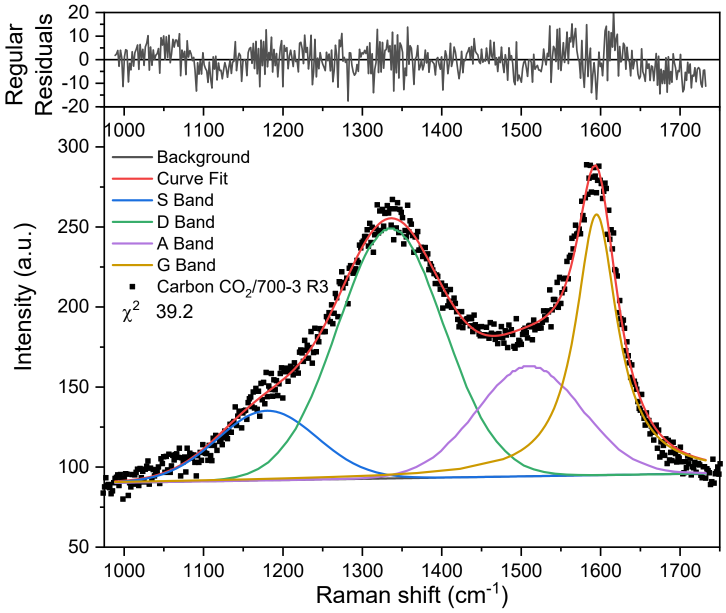

Figure S32. Repeatability of curve resolution of Raman spectra a) Carbon CO_2/700-1 Run 1, b) Carbon CO_2/700-1 Run 2, c) Carbon CO_2/700-1 Run 3, d) Carbon CO_2/700-3 Run 1, e) Carbon CO_2/700-3 Run 2 and f) Carbon CO_2/700-3 Run 3

7.2 Raman Spectra, Curve Fitting, and Residuals

a)

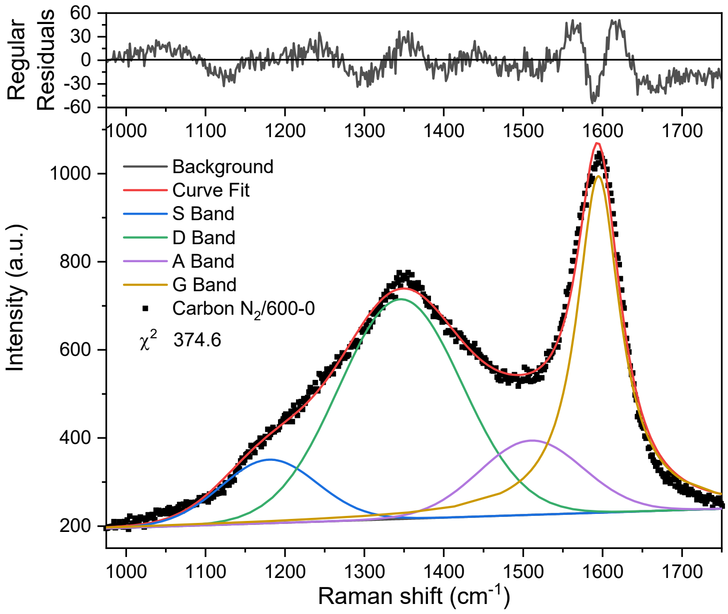

b)

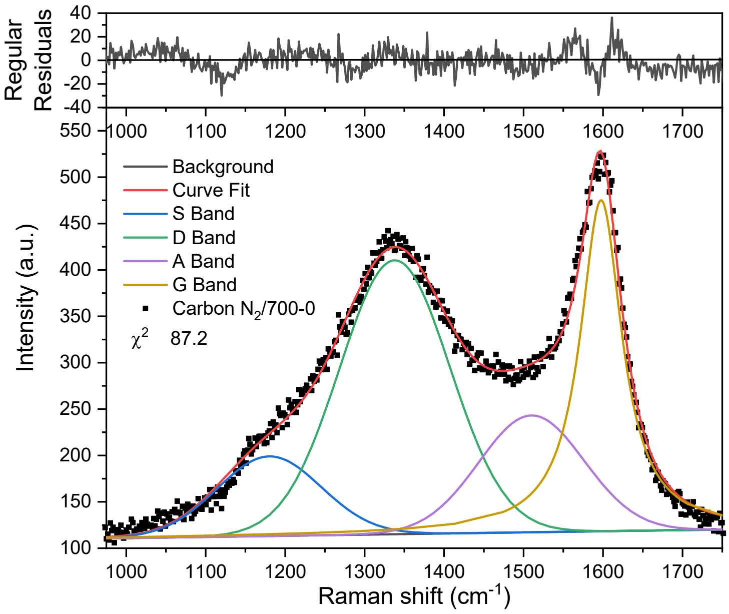

c)

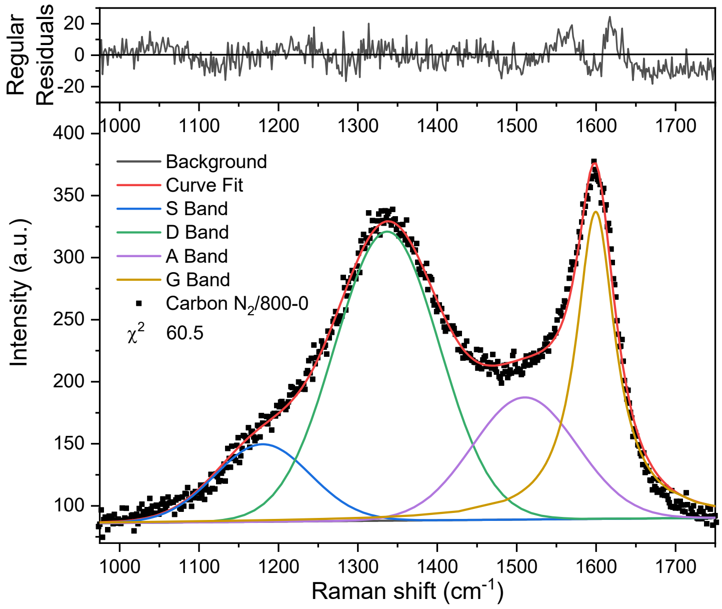

d)

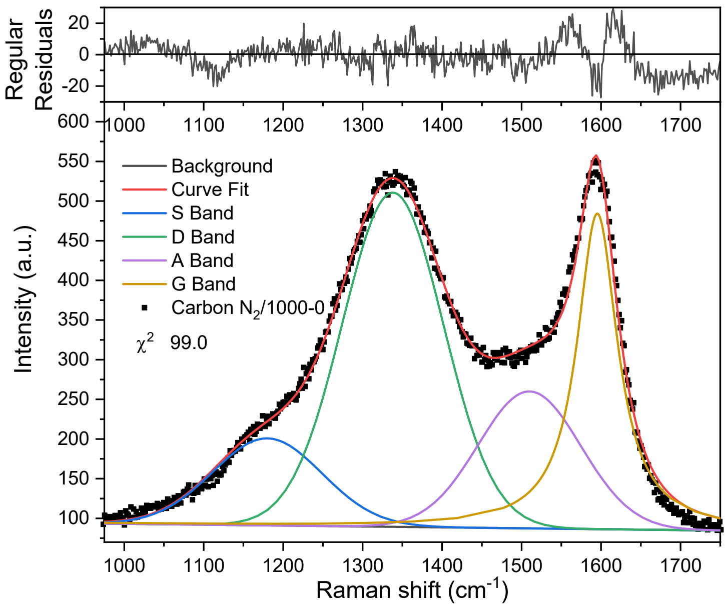

Figure S33. Raman spectra, peak fitting, and residuals for the Series 1 carbons prepared with a hold time of 0 h in a nitrogen atmosphere as a function of HTT a) Carbon N_2/600-0, b) Carbon N_2/700-0 c) Carbon N_2/800-0 and d) Carbon N_2/1000-0

a)

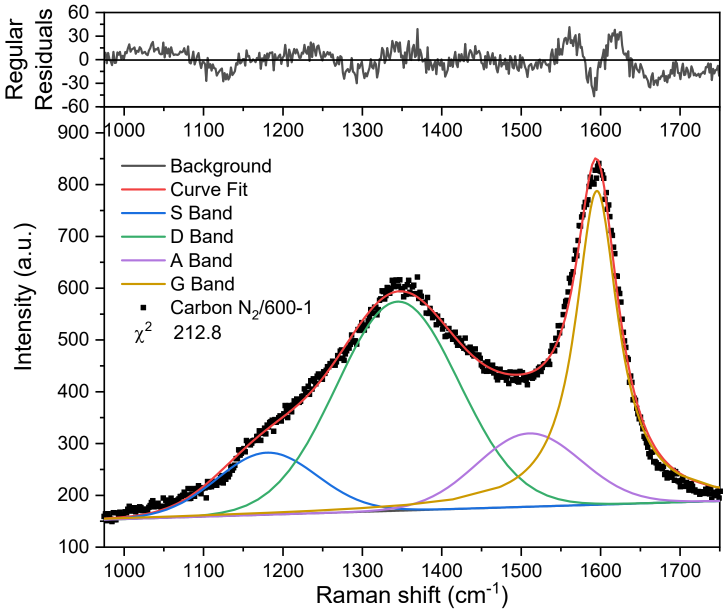
Regular Residuals
60
30
0
-30
-60
Intensity (a.u.)
Background
Curve Fit
S Band
D Band
A Band
G Band
Carbon N2/600-1
χ2 212.8
1000
1100
1200
1300
1400
1500
1600
1700
Raman shift (cm-1)

b)

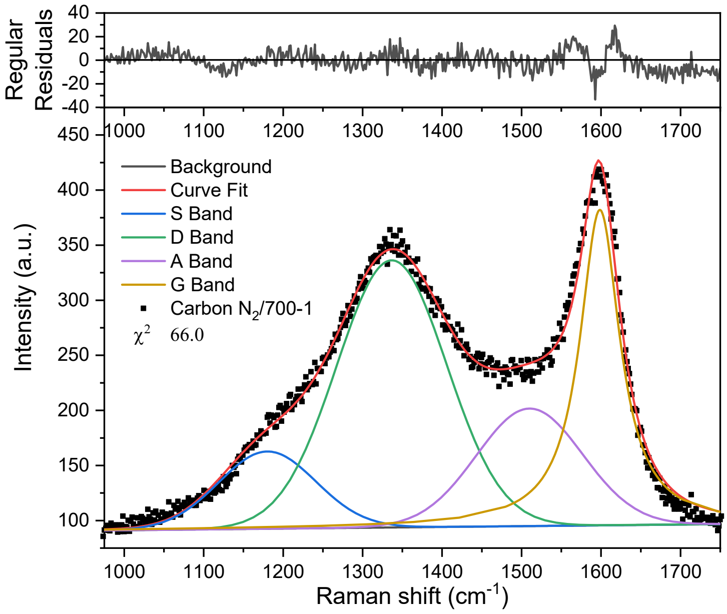
Regular Residuals
40
20
0
-20
-40
Intensity (a.u.)
Background
Curve Fit
S Band
D Band
A Band
G Band
Carbon N2/700-1
χ2 66.0
1000
1100
1200
1300
1400
1500
1600
1700
Raman shift (cm-1)

c)

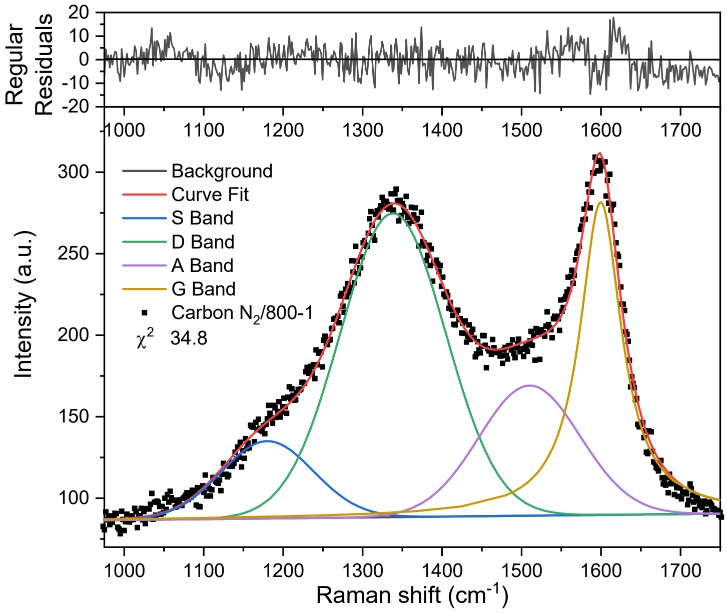

d)

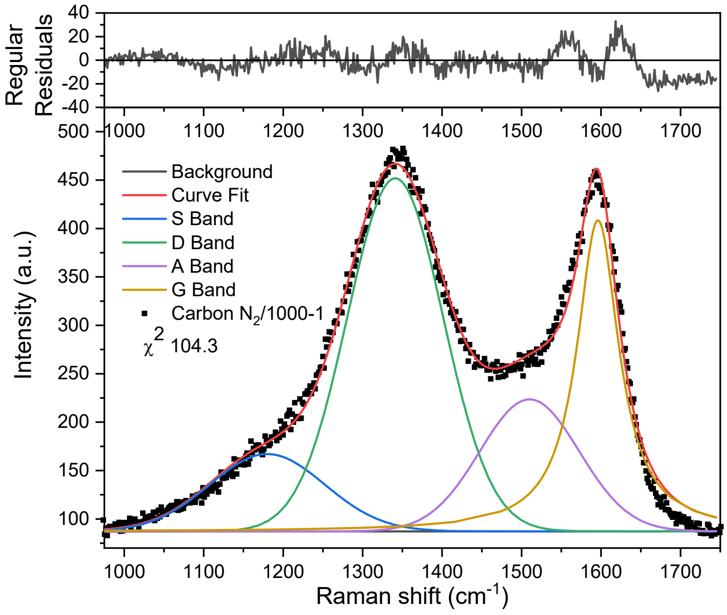

Figure S34. Raman spectra, peak fitting, and residuals for the Series 1 carbons prepared with a hold time of 1 h in a nitrogen atmosphere as a function of HTT a) Carbon N_2/600-1, b) Carbon N_2/700-1 c) Carbon N_2/800-1 and d) Carbon N_2/1000-1

a)

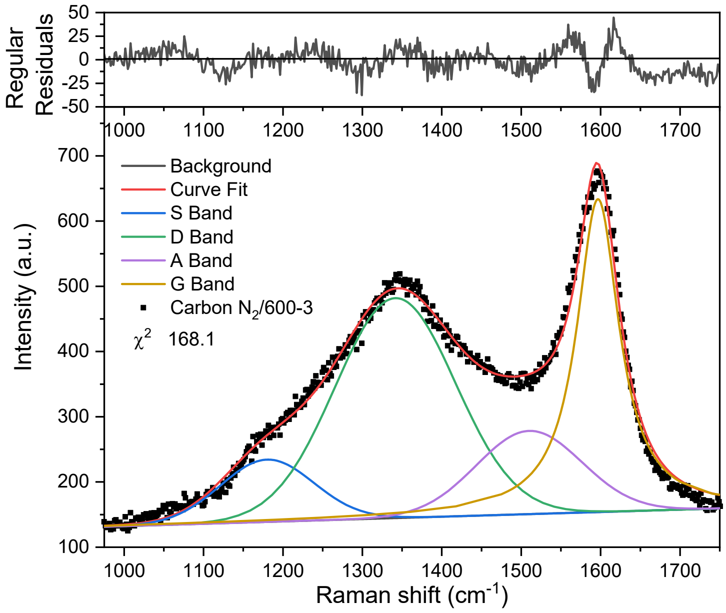
Regular Residuals
50
25
0
-25
-50
1000
1100
1200
1300
1400
1500
1600
1700
Intensity (a.u.)
700
600
500
400
300
200
100
Background
Curve Fit
S Band
D Band
A Band
G Band
Carbon N_2/600-3
χ^2 168.1
Raman shift (cm^{-1})

b)

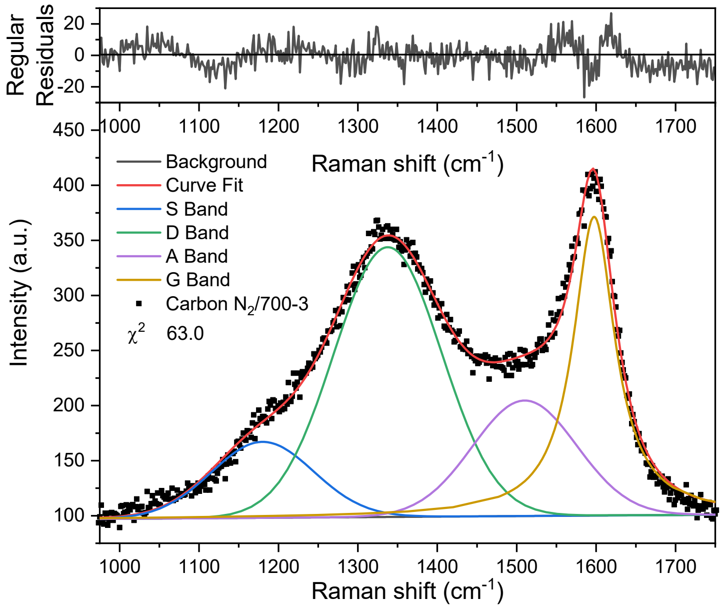
Regular Residuals
20
0
-20
1000
1100
1200
1300
1400
1500
1600
1700
Intensity (a.u.)
450
400
350
300
250
200
150
100
Background
Curve Fit
S Band
D Band
A Band
G Band
Carbon N_2/700-3
χ^2 63.0
Raman shift (cm^{-1})
Raman shift (cm^{-1})

c)

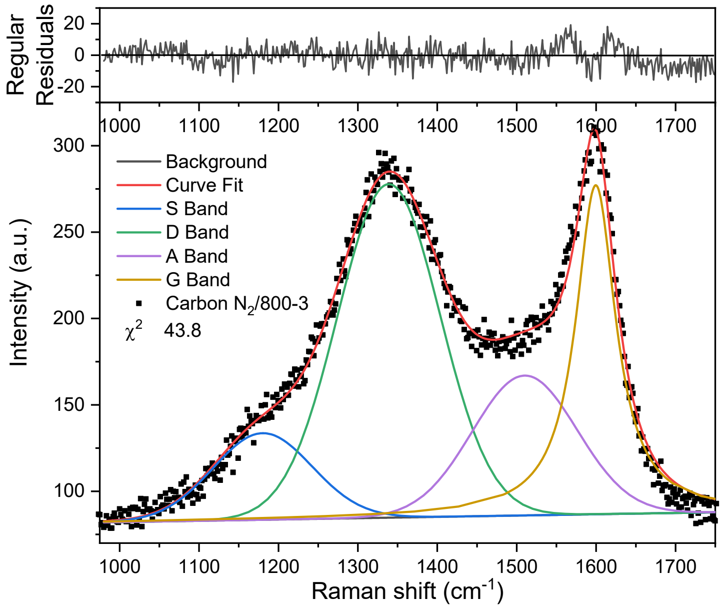

d)

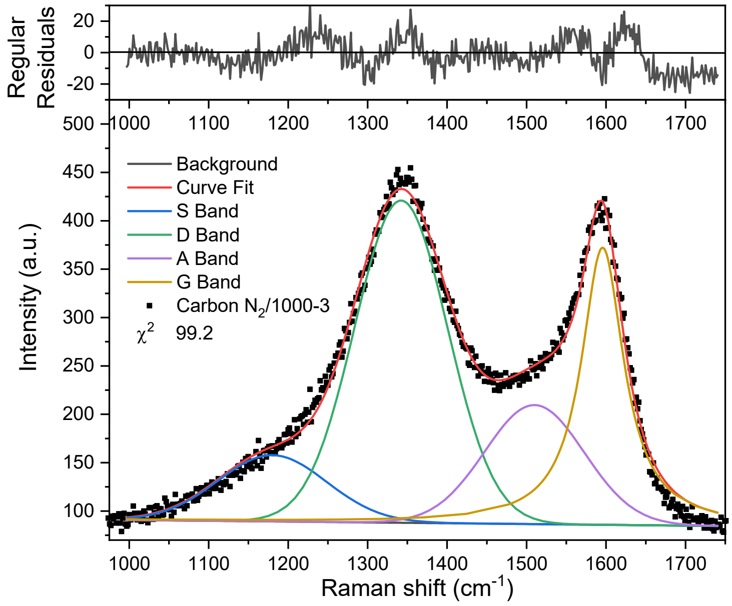

Figure S35. Raman spectra, peak fitting, and residuals for the Series 1 carbons prepared with a hold time of 3 h in a nitrogen atmosphere as a function of HTT a) Carbon N_2/600-3, b) Carbon N_2/700-3 c) Carbon N_2/800-3, and d) Carbon N_2/1000-3

a)

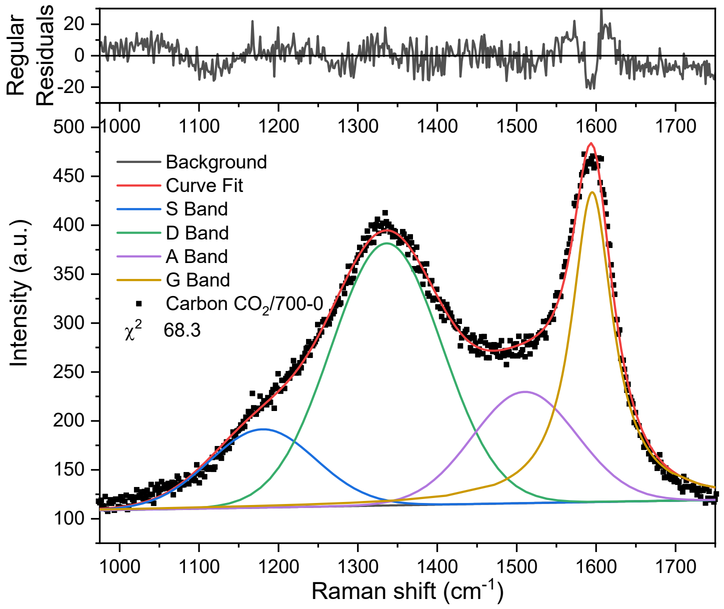

Regular Residuals
Intensity (a.u.)
Raman shift (cm-1)
Background
Curve Fit
S Band
D Band
A Band
G Band
Carbon CO2/700-0
χ2 68.3

b)

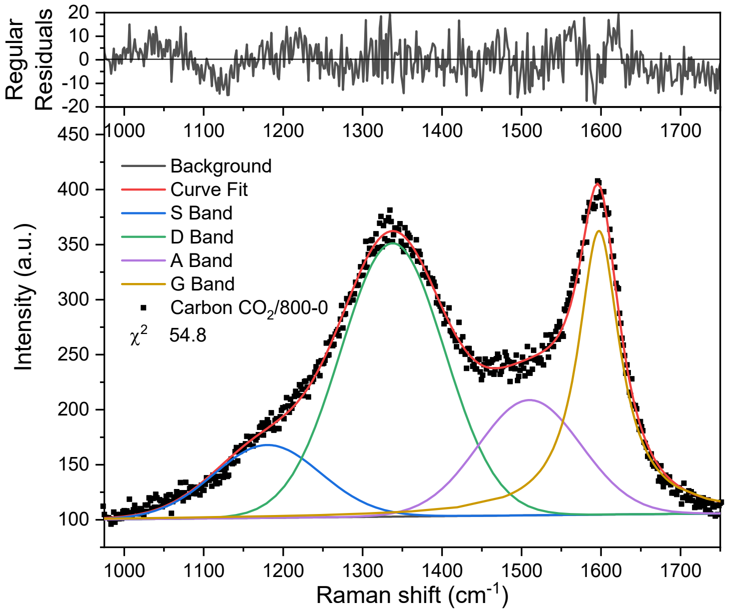

Regular Residuals
Intensity (a.u.)
Raman shift (cm-1)
Background
Curve Fit
S Band
D Band
A Band
G Band
Carbon CO2/800-0
χ2 54.8

c)

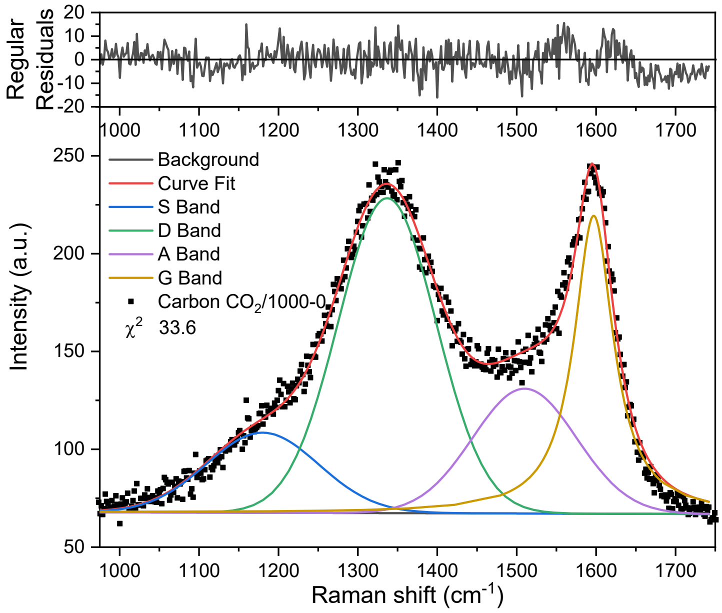

Figure S36. Raman spectra, peak fitting, and residuals for the Series 2 carbons with a hold time of 0 h prepared in a carbon dioxide atmosphere as a function of HTT a) Carbon CO_2/700-0, b) Carbon CO_2/800-0 and c) Carbon CO_2/1000-0.

a)

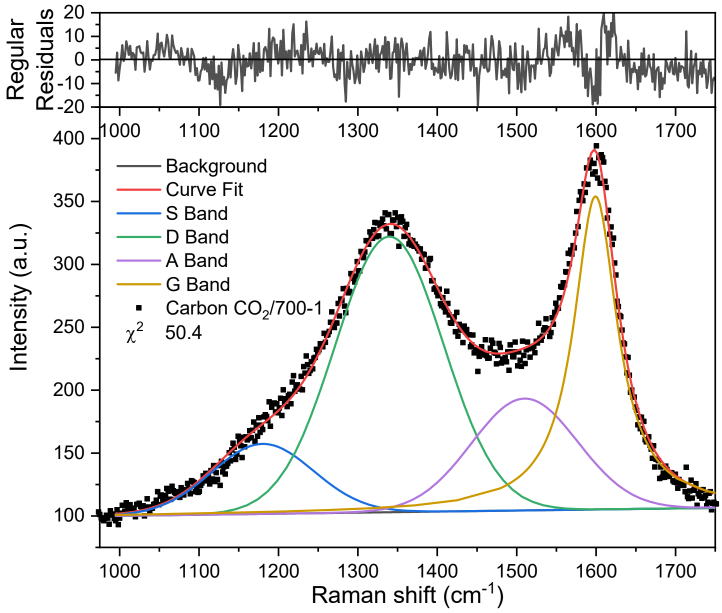

b)

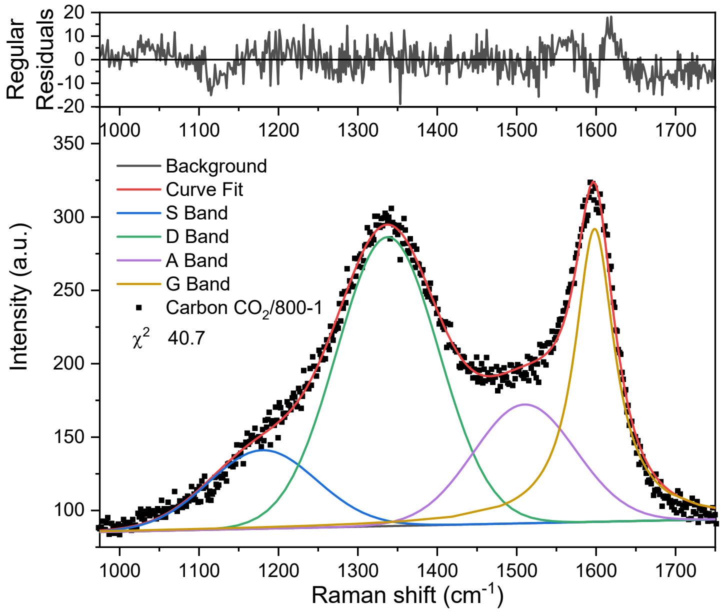

c)

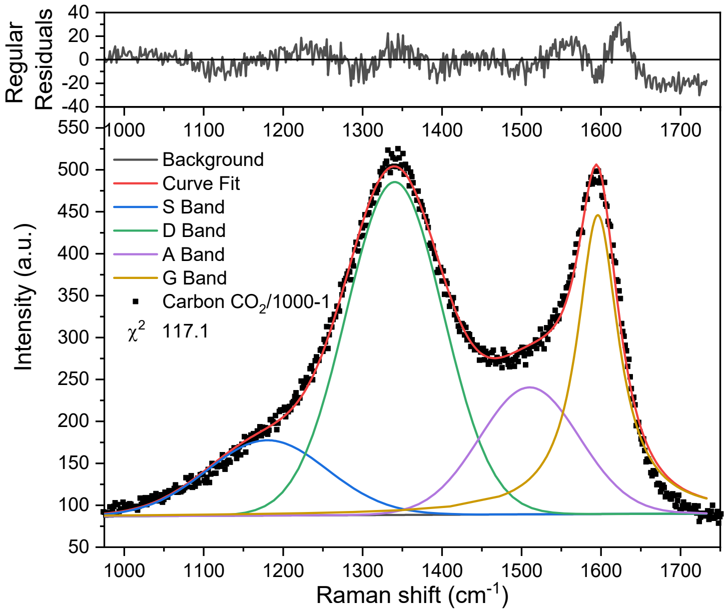

Figure S37. Raman spectra, peak fitting, and residuals for the Series 2 carbons with a hold time of 1 h prepared in a carbon dioxide atmosphere as a function of HTT a) Carbon CO_2/700-1, b) Carbon CO_2/800-1 and c) Carbon CO_2/1000-1.

a)

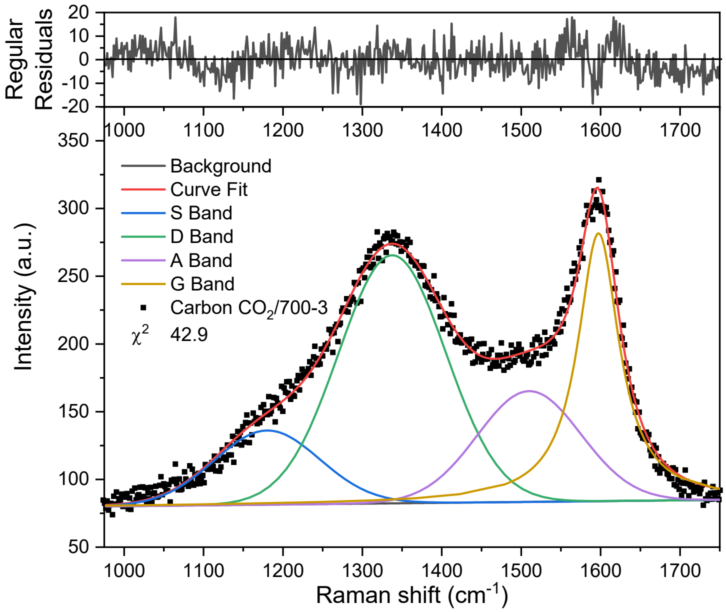

b)

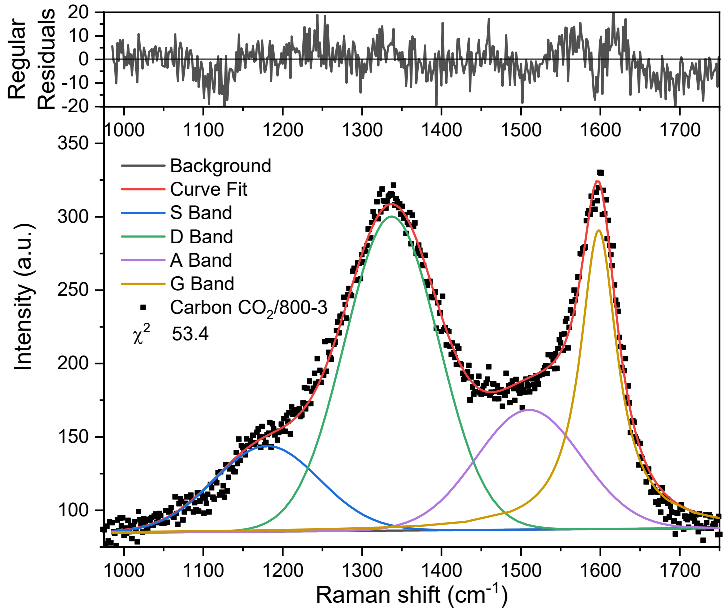

Figure S38. Raman spectra, peak fitting, and residuals for the Series 2 carbons with a hold time of 3 h prepared in a carbon dioxide atmosphere as a function of HTT a) Carbon CO_2/700-3 and b) Carbon CO_2/800-3.

a)

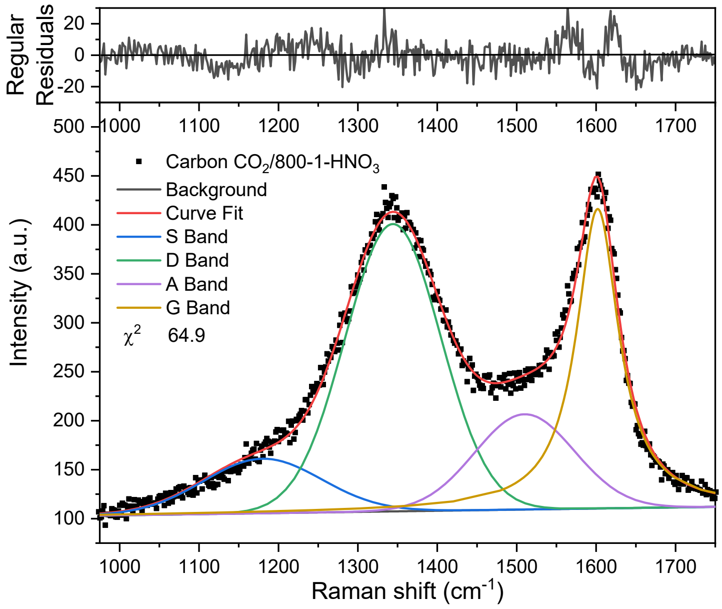
Regular Residuals
20
0
-20
Intensity (a.u.)
Carbon CO2/800-1-HNO3
Background
Curve Fit
S Band
D Band
A Band
G Band
χ2 64.9
Raman shift (cm-1)

b)

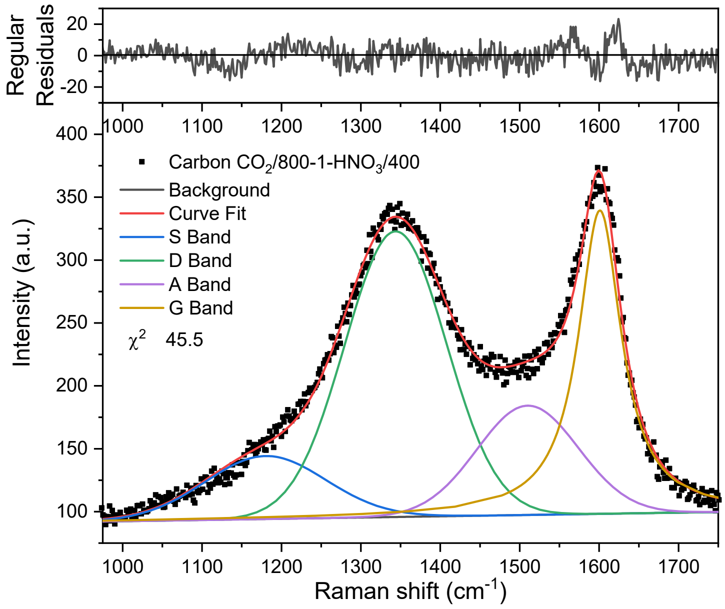
Regular Residuals
20
0
-20
Intensity (a.u.)
Carbon CO2/800-1-HNO3/400
Background
Curve Fit
S Band
D Band
A Band
G Band
χ2 45.5
Raman shift (cm-1)

c)

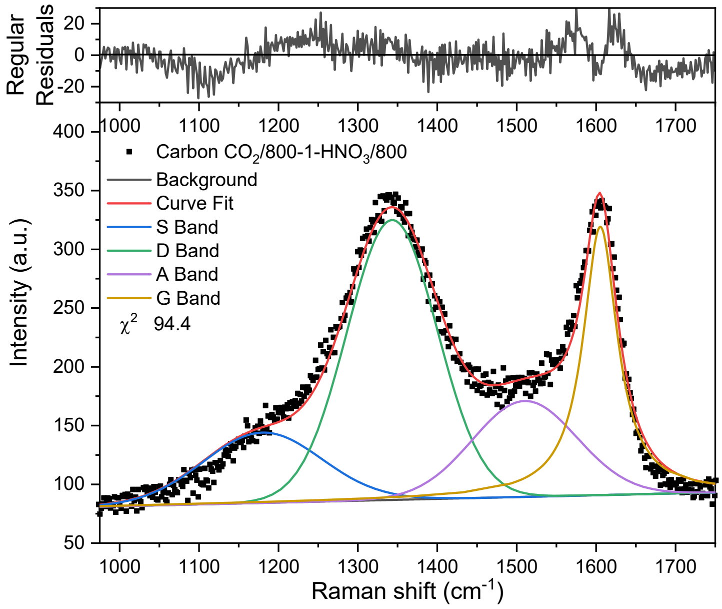

Regular Residuals
20
0
-20
1000 1100 1200 1300 1400 1500 1600 1700
Carbon CO_2/800-1-HNO_3/800
Background
Curve Fit
S Band
D Band
A Band
G Band
χ^2 94.4
Intensity (a.u.)
400 350 300 250 200 150 100 50
Raman shift (cm^{-1})

d)

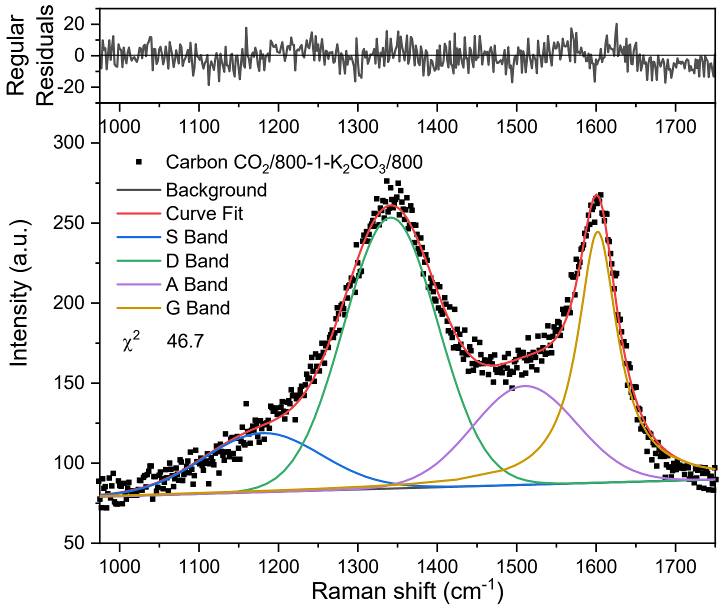

Regular Residuals
20
0
-20
1000 1100 1200 1300 1400 1500 1600 1700
Carbon CO_2/800-1-K_2CO_3/800
Background
Curve Fit
S Band
D Band
A Band
G Band
χ^2 46.7
Intensity (a.u.)
300 250 200 150 100 50
Raman shift (cm^{-1})

e)

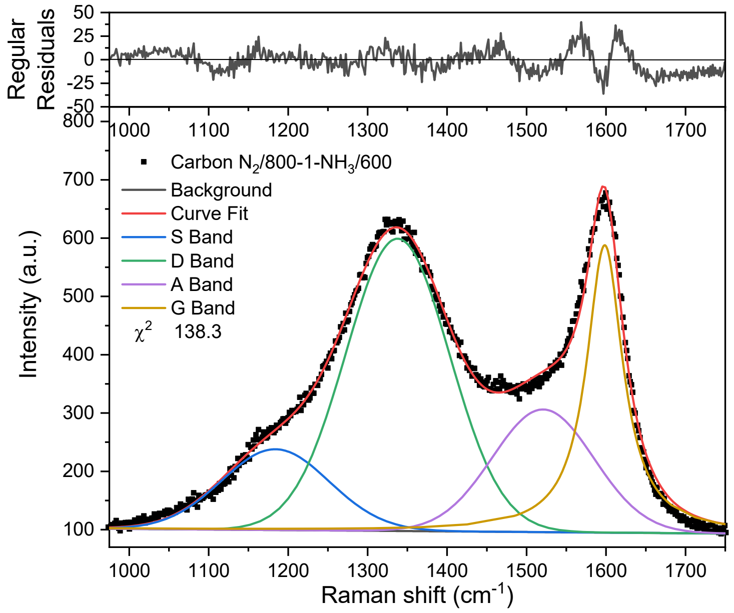

f)

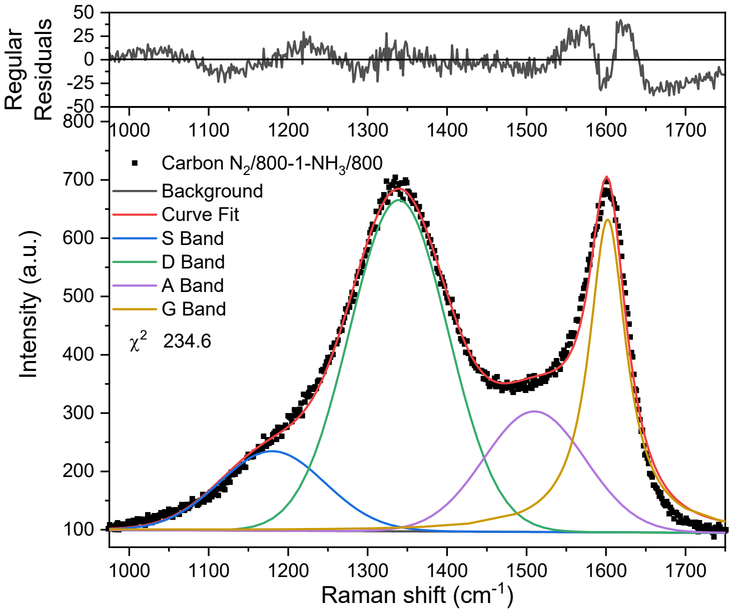

Figure S39. Raman spectra, peak fitting, and residuals for the functionalized carbons a) Carbon CO_2/800-1-HNO_3, b) Carbon CO_2/800-1-HNO_3/400, c) Carbon CO_2/800-1-HNO_3/800, d) Carbon CO_2/800-1-K_2CO_3/800, e) Carbon N_2/800-1-NH_3/600 and f) Carbon N_2/800-1-NH_3/800.

7.3 Comparisons of Raman, X-ray Diffraction, and Carbonization Temperature Data

a)

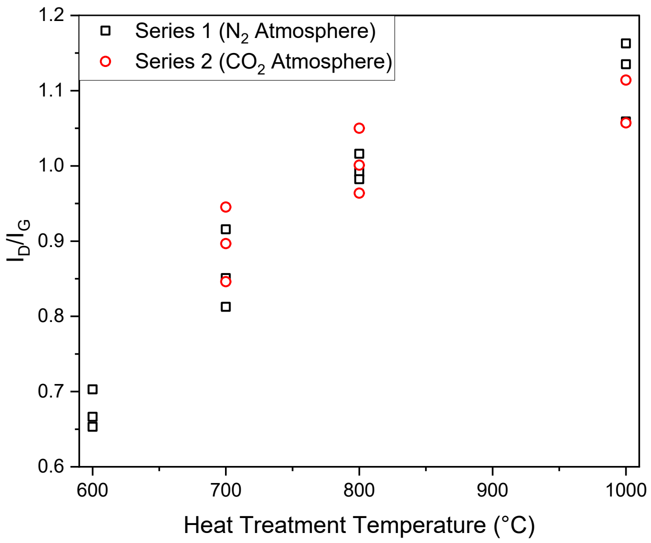

b)

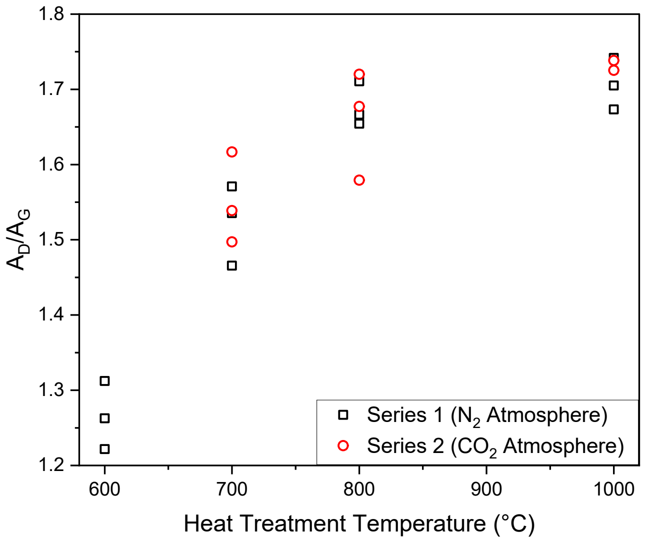

c)

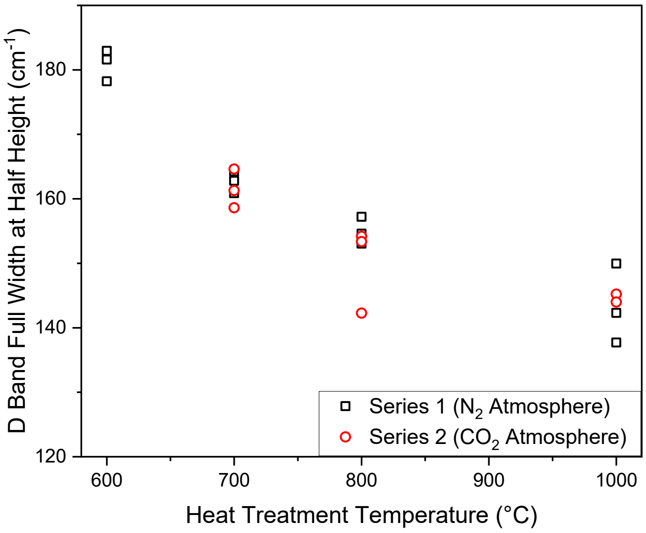

D Band Full Width at Half Height (cm^{-1})
180
160
140
120
600
700
800
900
1000
Heat Treatment Temperature (°C)
Series 1 (N_2 Atmosphere)
Series 2 (CO_2 Atmosphere)

d)

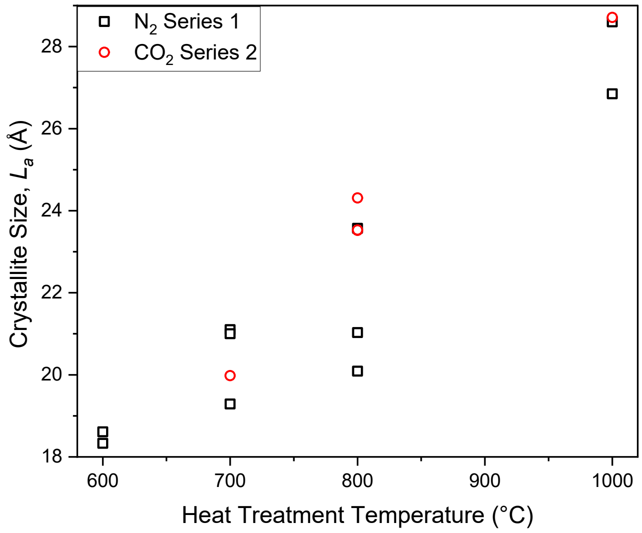

N_2 Series 1
CO_2 Series 2
Crystallite Size, L_a (Å)
28
26
24
22
20
18
600
700
800
900
1000
Heat Treatment Temperature (°C)

e)

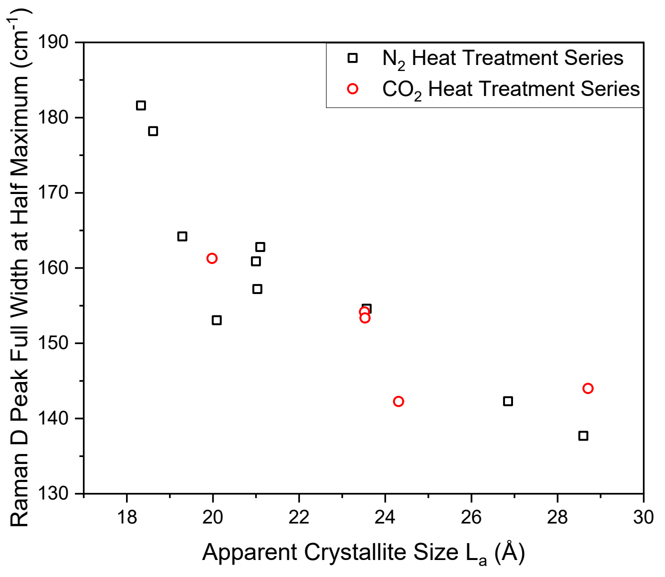

f)

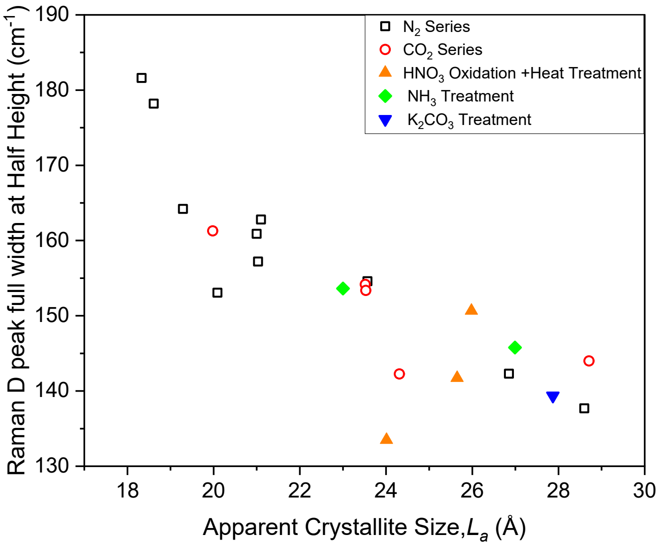

Figure S40. Comparison of the Raman D peak full width at half height, the apparent crystallite size (L_a), and heat treatment temperature (HTT) for carbons prepared under carbon dioxide and nitrogen atmospheres a) The variation of I_D/I_G with HTT, b) The variation of A_G/A_G with HTT, c)The variation of Raman D peak full width at half height with HTT, d) The variation of apparent crystallite size (L_a) with HTT, e) The variation of Raman D peak full width at half height with crystallite size (L_a) for carbonization series and f)) The variation of Raman D peak full width at half height with crystallite size (L_a) for carbonization under N_2 and CO_2 series and chemical treatment of carbons CO_2/800-1 and N_2/800-1.

8 Fourier Transform Infrared Spectroscopy

a)

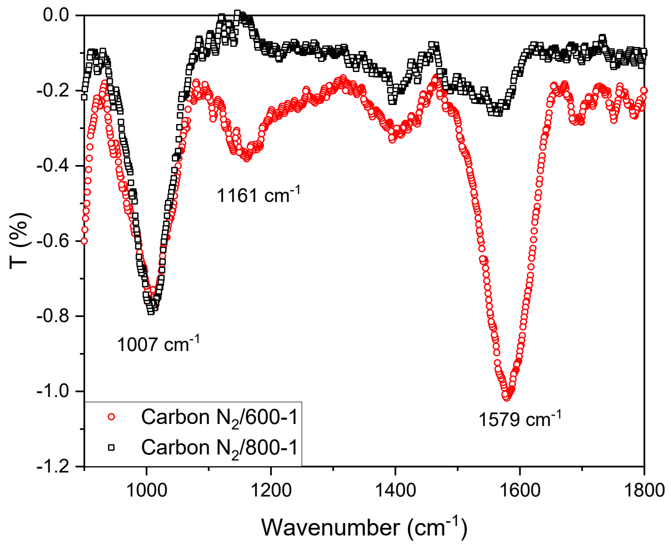

b)

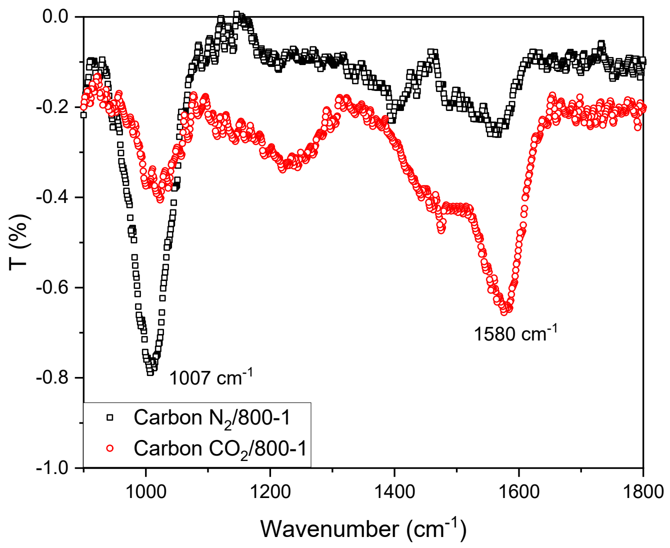

c)

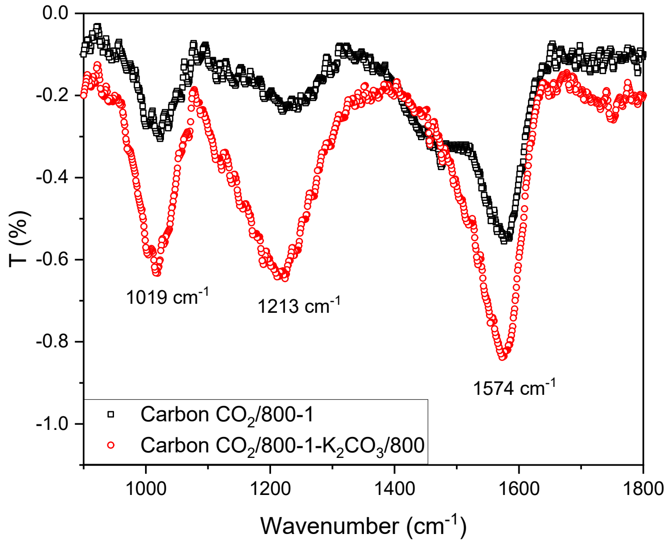
0.0
-0.2
-0.4
-0.6
-0.8
-1.0
T (%)
1019 cm-1
1213 cm-1
1574 cm-1
Carbon CO2/800-1
Carbon CO2/800-1-K2CO3/800
1000
1200
1400
1600
1800
Wavenumber (cm-1)

d)

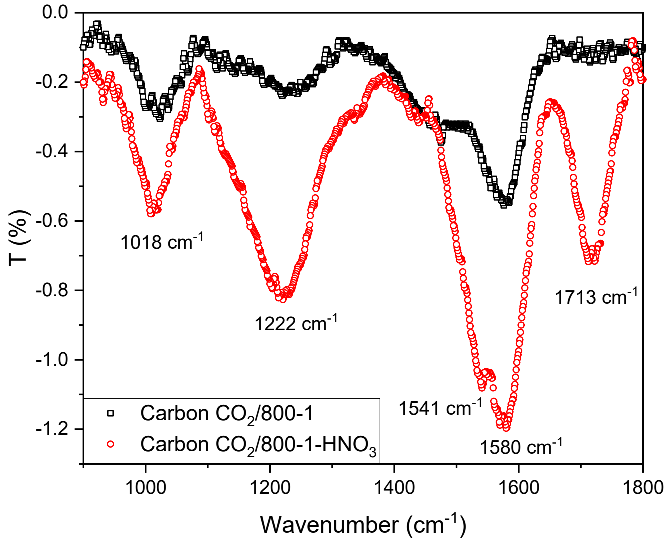
0.0
-0.2
-0.4
-0.6
-0.8
-1.0
-1.2
T (%)
1018 cm-1
1222 cm-1
1541 cm-1
1580 cm-1
1713 cm-1
Carbon CO2/800-1
Carbon CO2/800-1-HNO3
1000
1200
1400
1600
1800
Wavenumber (cm-1)

e)

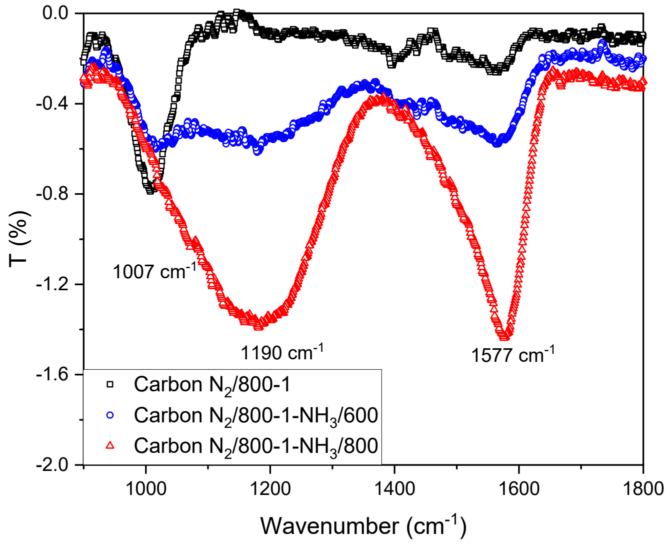

Figure S41 Comparison of infrared spectra of various carbons a) Carbons N_2/600-1 and N_2/800-1, b) Carbons N_2/800-1 and CO_2/800-1, c) Carbons CO_2/800-1 and CO_2/800-1-K_2CO_3/800, d) Carbons CO_2/800-1 and CO_2/800-1-HNO_3, e) Carbons N_2/800-1, N_2/800-1-NH_3/600 and N_2/800-1-NH_3/800. Spectra have been modified by background subtraction for comparison purposes.

9 Electron Microscopy

TEM-BF and TEM-DF images of carbon sample (CO_2/800-0) are illustrated in Figures S41a and b. SAED pattern (Figure 41c) fits a graphite hexagonal structure (a = 0.242 ± 0.005 nm, c = 0.660 ± 0.005 nm). These values are similar to the values for the structure of hexagonal graphite (a = 0.2461 nm, c = 0.6708 nm)[1]. TEM-DF image (Figure S41b) of (002) diffraction beam shows the contrast from crystalline graphite appearing bright in the image. The distance between carbon layers is shown in Figure S41d. This confirms the presence of small carbon nanostructures randomly distributed throughout the carbon.

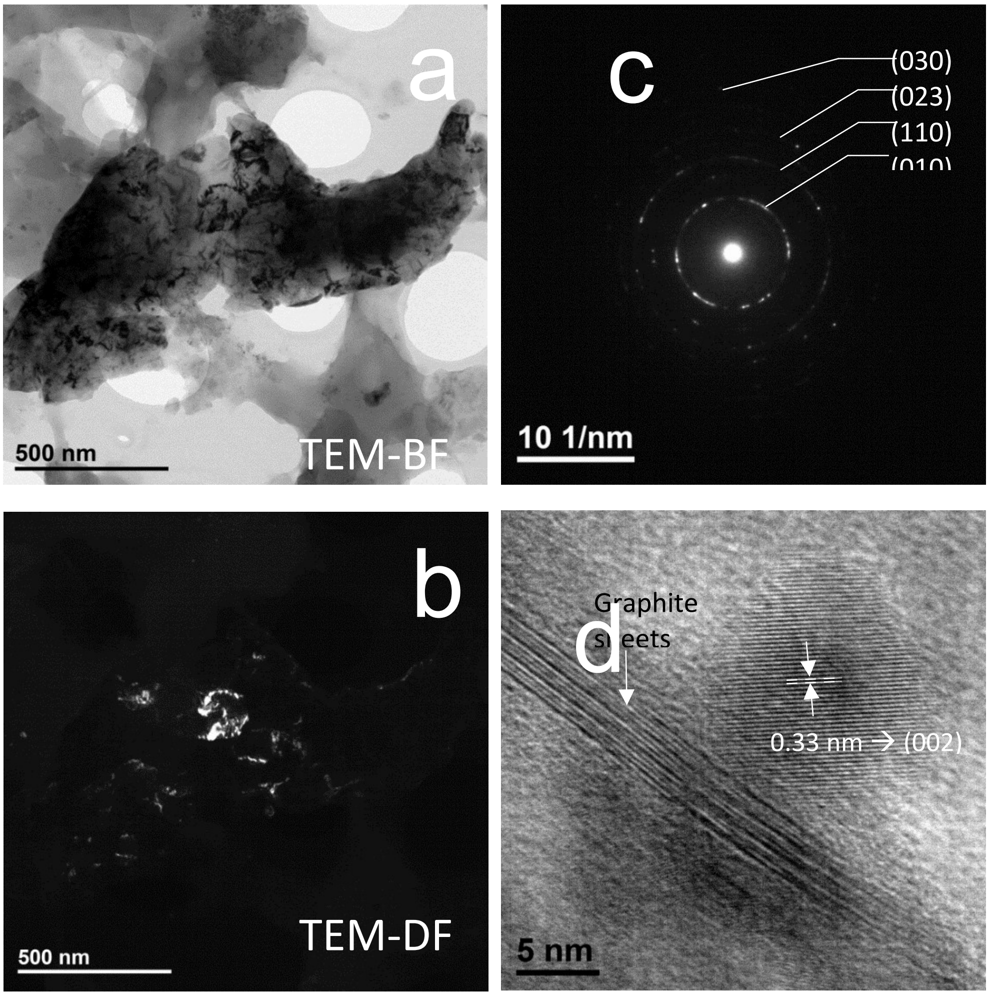

Figure S42. Electron micrographs (a) TEM-BF image, (b) TEM-DF image, (c) SAED pattern, and (d) HR-TEM of carbon CO_2/800-0.

10. Comparison of Characterization Data for the HNO_3 Functionalized Carbons

a)

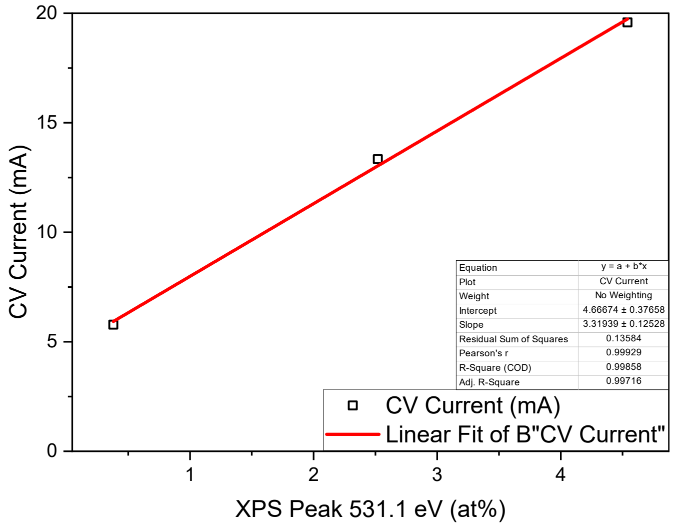

b)

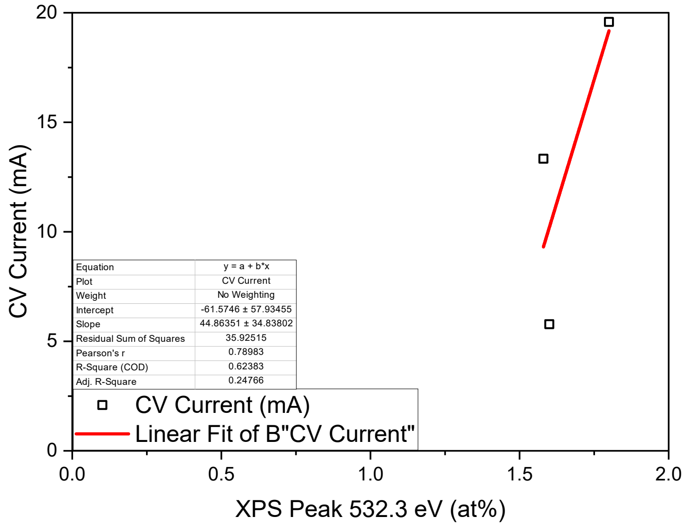

c)

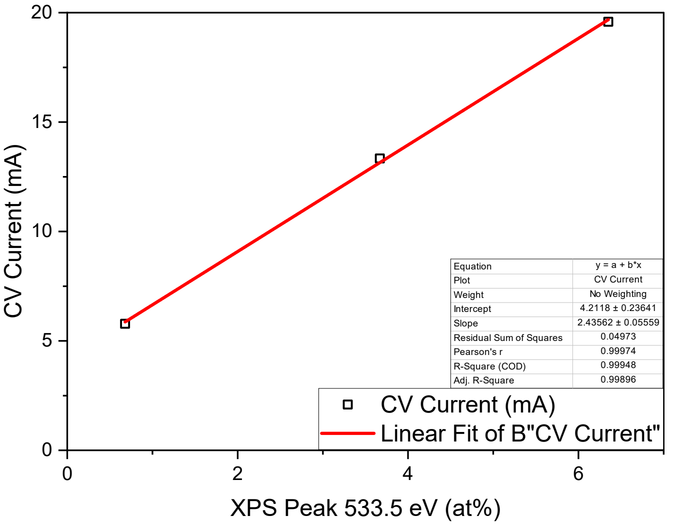

CV Current (mA)
XPS Peak 533.5 eV (at%)
Equation y = a + b*x
Plot CV Current
Weight No Weighting
Intercept 4.2118 ± 0.23641
Slope 2.43562 ± 0.05559
Residual Sum of Squares 0.04973
Pearson's r 0.99974
R-Square (COD) 0.99948
Adj. R-Square 0.99896
CV Current (mA)
Linear Fit of B"CV Current"

d)

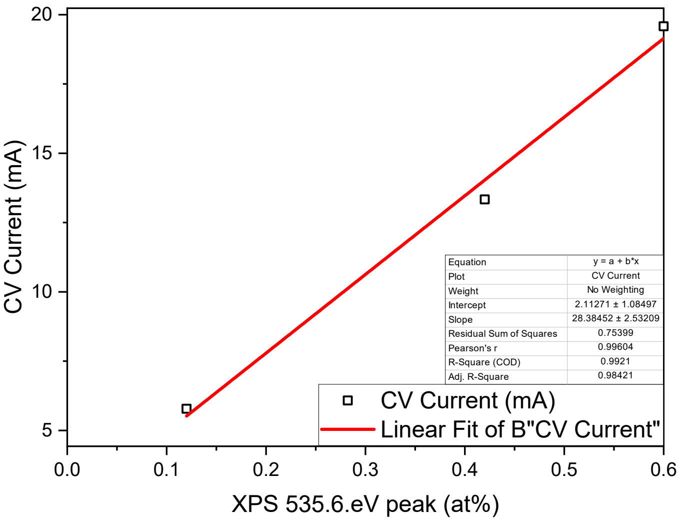

CV Current (mA)
XPS 535.6.eV peak (at%)
Equation y = a + b*x
Plot CV Current
Weight No Weighting
Intercept 2.11271 ± 1.08497
Slope 28.38452 ± 2.53209
Residual Sum of Squares 0.75399
Pearson's r 0.99604
R-Square (COD) 0.9921
Adj. R-Square 0.98421
CV Current (mA)
Linear Fit of B"CV Current"

e)

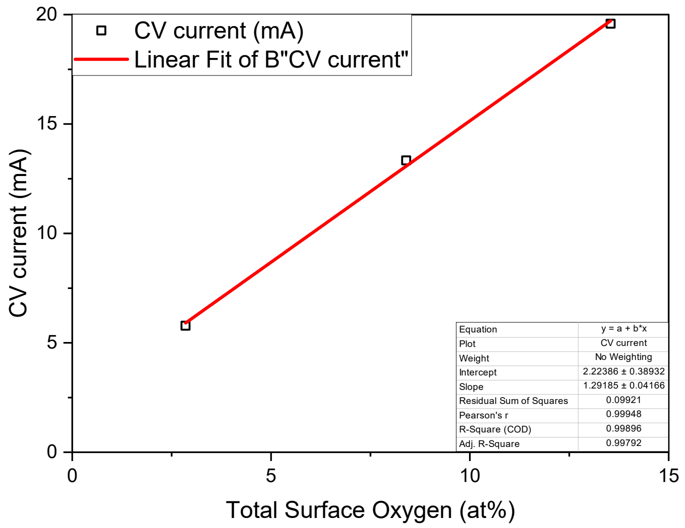

CV current (mA)
Linear Fit of B"CV current"
Equation y = a + b*x
Plot CV current
Weight No Weighting
Intercept 2.22386 ± 0.38932
Slope 1.29185 ± 0.04166
Residual Sum of Squares 0.09921
Pearson's r 0.99948
R-Square (COD) 0.99896
Adj. R-Square 0.99792
CV current (mA)
Total Surface Oxygen (at%)
0
5
10
15
20

f)

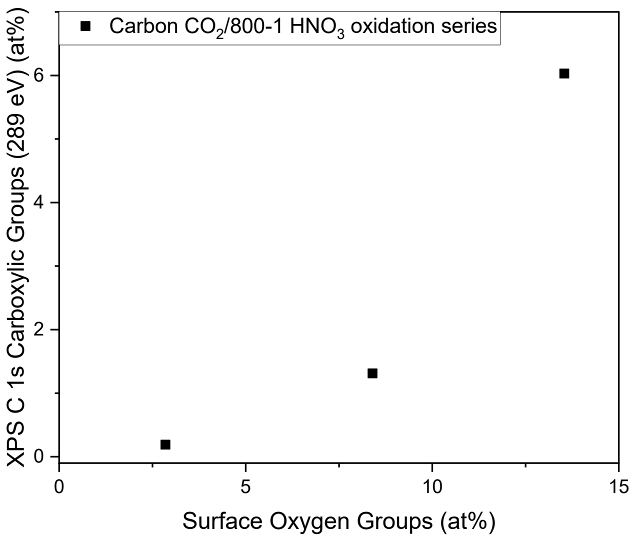

Carbon CO2/800-1 HNO3 oxidation series
XPS C 1s Carboxylic Groups (289 eV) (at%)
Surface Oxygen Groups (at%)
0
2
4
6
5
10
15

g)

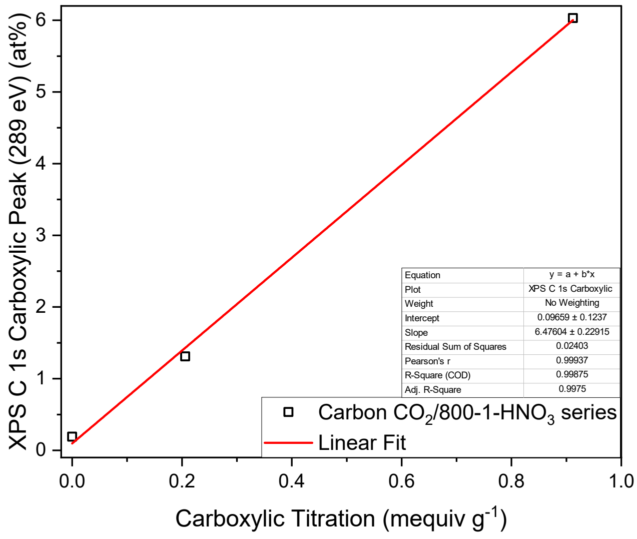

h)

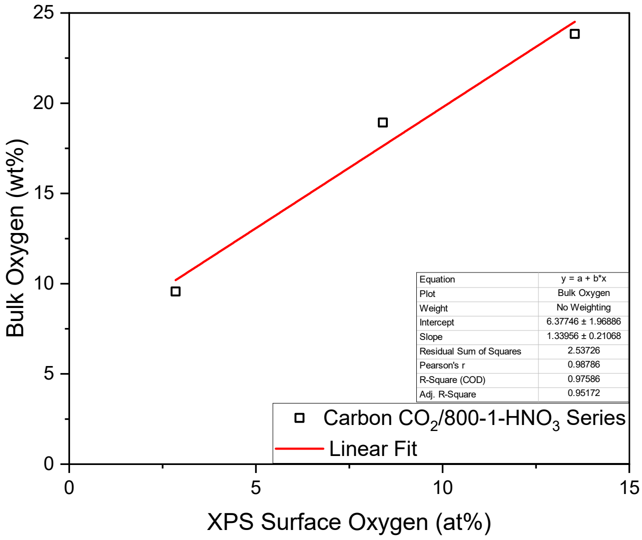

Figure S43. Comparison of characterization data for the HNO_3 functionalized carbons a) The variation of CV current with XPS 531.1 eV peak concentration, b) The variation of CV current with XPS 532.3 eV peak concentration, c) The variation of CV current with XPS 533.5 eV peak concentration, d) The variation of CV current with XPS 535.5 eV peak concentration, e) The variation of CV current with XPS surface oxygen concentration, f) The variation XPS C 1s carboxylic groups with XPS surface oxygen groups, g) The variation XPS C 1s carboxylic

groups with carboxylic groups obtained from titration measurements, h) The variation of bulk oxygen from chemical analysis with XPS surface oxygen group measurements.

REFERENCES

1. M. Inagaki, in *Handbook of Advanced Ceramics*, ed. S. Somiya, Academic Press, Oxford, 2 edition., 2013, ch. 2, pp. 25-60.
